# Web Evaluation at the US National Institutes of Health: Use of the American Customer Satisfaction Index Online Customer Survey

**DOI:** 10.2196/jmir.944

**Published:** 2008-02-15

**Authors:** Fred B Wood, Elliot R Siegel, Sue Feldman, Cynthia B Love, Dennis Rodrigues, Mark Malamud, Marie Lagana, Jennifer Crafts

**Affiliations:** ^6^Westat IncRockvilleMDUSA; ^5^Center for Information TechnologyNational Institutes of HealthUS Department of Health and Human ServicesBethesdaMDUSA; ^4^National HeartLungand Blood InstituteNational Institutes of HealthUS Department of Health and Human ServicesBethesdaMDUSA; ^3^Office of Communications and Public LiaisonOffice of the DirectorNational Institutes of HealthUS Department of Health and Human ServicesBethesdaMDUSA; ^2^National Cancer InstituteNational Institutes of HealthUS Department of Health and Human ServicesBethesdaMDUSA; ^1^National Library of MedicineNational Institutes of HealthUS Department of Health and Human ServicesBethesdaMDUSA

**Keywords:** Surveys, evaluation studies, satisfaction, Internet, World Wide Web, consumer health information

## Abstract

**Background:**

The National Institutes of Health (NIH), US Department of Health and Human Services (HHS), realized the need to better understand its Web users in order to help assure that websites are user friendly and well designed for effective information dissemination. A trans-NIH group proposed a trans-NIH project to implement an online customer survey, known as the American Customer Satisfaction Index (ACSI) survey, on a large number of NIH websites—the first “enterprise-wide” ACSI application, and probably the largest enterprise Web evaluation of any kind, in the US government. The proposal was funded by the NIH Evaluation Set-Aside Program for two years at a cost of US $1.5 million (US $1.275 million for survey licenses for 60 websites at US $18,000 per website; US $225,000 for a project evaluation contractor).

**Objective:**

The overall project objectives were to assess the value added to the participating NIH websites of using the ACSI online survey, identify any NIH-wide benefits (and limitations) of the ACSI, ascertain any new understanding about the NIH Web presence based on ACSI survey results, and evaluate the effectiveness of a trans-NIH approach to Web evaluation. This was not an experimental study and was not intended to evaluate the ACSI survey methodology, per se, or the impacts of its use on customer satisfaction with NIH websites.

**Methods:**

The evaluation methodology included baseline pre-project websites profiles; before and after email surveys of participating website teams; interviews with a representative cross-section of website staff; observations of debriefing meetings with website teams; observations at quarterly trans-NIH Web staff meetings and biweekly trans-NIH leadership team meetings; and review and analysis of secondary data.

**Results:**

Of the original 60 NIH websites signed up, 55 implemented the ACSI survey, 42 generated sufficient data for formal reporting of survey results for their sites, and 51 completed the final project survey. A broad cross-section of websites participated, and a majority reported significant benefits and new knowledge gained from the ACSI survey results. NIH websites as a group scored consistently higher on overall customer satisfaction relative to US government-wide and private sector benchmarks.

**Conclusions:**

Overall, the enterprise-wide experiment was successful. On the level of individual websites, the project confirmed the value of online customer surveys as a Web evaluation method. The evaluation results indicated that successful use of the ACSI, whether site-by-site or enterprise-wide, depends in large part on strong staff and management support and adequate funding and time for the use of such evaluative methods. In the age of Web-based e-government, a broad commitment to Web evaluation may well be needed. This commitment would help assure that the potential of the Web and other information technologies to improve customer and citizen satisfaction is fully realized.

## Introduction

At the US National Institutes of Health (NIH), as at many other biomedical institutions, World Wide Web–based information dissemination now dominates [1,2]. The use of the Internet and Web at NIH has grown dramatically over the last decade, to the point where all major NIH organizations have one or more websites. NIH has realized the necessity to better understand Web users in order to help assure that websites are user friendly and well designed for effective information dissemination.

### Multidimensional Approach

Over the last several years, various individual NIH organizations have experimented with several different methods of Web evaluation [3-5]. These methods have evolved into a so-called “multidimensional approach” to Web evaluation that acknowledges that no one evaluation method meets all needs. Methods may vary with the preferences and sophistication of individual website teams, complexity of websites, and stage of the website improvement cycle.

The multidimensional approach can be described as including methods in four categories: usability testing, user feedback, usage data, and website and Internet performance data. These methods are primarily based on feedback from both users and the systems that monitor Web servers and Internet performance [6].

Another way to describe the multidimensional approach divides evaluation methods into two groups: what users say about a website, and what experts say. Prior to the American Customer Satisfaction Index (ACSI) project reported here, NIH as a whole placed the greater emphasis on evaluating its website content by “what experts say,” ensuring quality information through writing and review of Web content by subject experts. This ACSI project is one step in giving Web teams at NIH another tool to learn more about “what users say.”

User opinions and behavior—what users say—are expressed through Web logs, surveys, focus groups, email, phone, personal contact, words used in search queries, Internet audience measurement, usability studies, and other methods [6,7]. NIH websites vary considerably in the budget, staff, and time they have to implement Web evaluation based on user input. For many of the websites participating in this study, this ACSI project was their first opportunity to get routine, structured, direct-from-the-user feedback.

Customer satisfaction surveys, like the ACSI, are one tool for listening to “what users say” to determine user perceptions of a website’s usefulness and performance. Perceptions are inherently subjective, but they do help Web managers understand another facet of user opinion. Other prior user-based evaluations at NIH have included search log analysis of user queries on a website or user queries on referring sites such as major Internet portals [8,9], analysis of email from users [10], and research on market share for online health information services [11].

The second group of Web evaluation methods, what experts say, is already heavily used in evaluating NIH Web content because of the inherent importance of providing accurate health information that can be accessed by many different audiences. NIH organizations have focused considerable efforts on ensuring that their websites convey the highest quality health information and reflect the latest findings from medical research.

Especially in health and medicine, subjectively perceived customer satisfaction can be only one measure of the value of a health information website. Some users might readily find a well-designed website with convincing graphics, testimonials, and popular appeal to be “highly satisfactory,” even if the site’s health information content is misleading, erroneous, or even harmful. NIH websites aim to be both well-designed and credible. Examples of NIH’s strengths in evaluating content include the efforts of websites to use strict guidelines for selecting and writing health content [12,13], evaluate content for readability and ethnic/cultural sensitivity [14], fund and implement research on Web design of health information for children, seniors [15], and others, and to secure external accreditation from organizations like Health On the Net (HON) Foundation [16].

In the age of Web-based e-government, a broad commitment to Web evaluation may well be needed. This commitment would help assure that the potential of the Web and other information technologies to improve customer and citizen satisfaction is fully realized.

### Customer Satisfaction

In parallel with the rise to dominance of the Internet and Web has been an increasing emphasis on “customer satisfaction” in the US government. Customer satisfaction is viewed as an important metric of the political goal of developing a more “customer-centric” government that is more responsive to citizen needs. These needs include a wide range of types of information from the government. In the case of NIH, citizens are seeking biomedical and health information on diverse diseases, conditions, health trends, research results, and the like.

There are many examples of requirements for the federal government to address customer needs and satisfaction. The Government Performance and Results Act of 1993 states the following: “The purposes of this act are to…improve Federal program effectiveness and public accountability by promoting a new focus on results, service quality, and *customer satisfaction*” [17] (italics added for emphasis).

The Office of Management and Budget (OMB) Circular A-130: Management of Federal Information Resources requires agencies to develop enterprise architecture that “will define principles and goals and set direction on such issues as the promotion of interoperability, open systems, public access, compliance with GPRA, *end user satisfaction*, and IT security” [18] (italics added for emphasis). The OMB Circular also requires demonstrating “a projected return on the investment that is clearly equal to or better than alternative uses of available public resources. The return may include improved mission performance in accordance with Government Performance and Results Act measures, reduced cost, increased quality, speed, or flexibility; as well as *increased customer and employee satisfaction* [18, 19] (italics added for emphasis).

In 2004, the Interagency Committee on Government Information wrote “Recommended Policies and Guidelines for Federal Public Websites” [20] at the request of OMB. The suggestions formed the basis for “Policies for Federal Agency Public Websites” [21] issued by OMB. The recommended policies document includes extensive implementation guidance, currently used by federal Web managers, and suggests the use of “customer satisfaction surveys.” Of key importance to the use of the ACSI at NIH is this provision:

**2e. Requirement: Organizations Must Measure Customer Satisfaction and Usability of Federal Public websites.** Organizations must evaluate customer satisfaction and usability of their websites and use the assessments to improve the websites. Federal public websites that reach the widest audiences—including agency websites and all second-level domain names registered in .gov, .mil, or .fed.us—must use a standard customer satisfaction survey.

Rationale: Organizations that create federal public websites, and the citizens they serve, want these websites to be as useful as possible. While Web content managers do their best to write and organize their websites to be effective, they need to test their websites to identify problem areas and then fix those problems. A common customer satisfaction survey will reduce costs government-wide and compare government websites with each other.

### Online User Survey

Within the multidimensional evaluative approach, the online user survey is the method that provides the most direct feedback from users. Online user surveys can generate data on the types of users coming to a website, user demographics, levels of user satisfaction with the website and the information provided, and intended use of the information obtained.

Various NIH organizations, and in particular the National Library of Medicine (NLM) and National Cancer Institute (NCI), have a long history with user surveys, dating from the pre-Web era. NLM, for example, transitioned from paper to online surveys in the early 1980s and then to Web-based surveys in the late 1990s. These were snapshot surveys—typically fielded for 2 or 3 weeks—and only provided a “snapshot” of the customer base and were implemented at most once a year [22,23, personal communication, Cindy Love, National Library of Medicine, April 30, 2007]. In addition, there were few standard methods or benchmarks for surveys of websites.

In comparison, the ACSI methodology offers several advantages: continuous data collection, randomized rolling sample, rigorous standardized survey methodology, standardized questions plus capability for optional custom questions, and extensive benchmarking of results.

The ACSI was first implemented in 1994 as an offline survey measuring customer satisfaction with businesses [24] and was adapted to the Internet in 2002 [25]. More than two dozen other federal websites began using the survey in 2002 [26].

During the late 1990s, the President’s Management Council, composed of the chief operating officers of each cabinet-level agency, responded to then Vice President Al Gore’s National Performance Review (also known as the National Partnership for Reinventing Government) initiative by considering ways to measure citizen satisfaction with government services. The Council members and other government leaders were interested in measuring government services using the same methods as the private sector and holding government programs to a level of customer responsiveness equal to or better than the private sector [27]. The Council, with the Government Services Administration (GSA) taking the lead, solicited proposals for a measurement tool that could be used across multiple agencies and provide benchmarking among agencies and between government and nongovernment providers of services or goods.

In 1999, using federal contract competition processes, GSA awarded the contract to Arthur Andersen LLC and the University of Michigan to provide the ACSI for wide adoption as a survey measure of offline government services [28]. The ACSI was already well established as a measure of customer satisfaction in nongovernment sectors, routinely publishing its results in the Wall Street Journal and other prominent publications. This was the first opportunity for government agencies to use the same yardstick. GSA successfully sought clearance under the Paperwork Reduction Act for blanket permission for any agency to use the survey.

The contracting function and survey clearance responsibilities were assumed by the Federal Consulting Group in January 2000. The ACSI serves a unique role as the most widely and easily available survey instrument for federal government. Early users of the offline ACSI included the agencies that have the greatest contact with citizens such as the Social Security Administration (retirement beneficiaries), the Internal Revenue Service (tax filers), the State Department (passport applicants), the Customs Service (international travelers), the Department of Veterans Affairs (compensation and medical care beneficiaries), and others. In October 2001, the ACSI also became available for online use through contract arrangements between ForeSee Results, Inc. and the Federal Consulting Group. The first online use was piloted by GSA for firstgov.gov (now USA.gov) and by NASA for NASA.gov. By mid-2002, the Federal Consulting Group obtained a generic clearance from the Office of Management and Budget for agencies that used the ForeSee Results Web metric tool and began to promote the use of the ACSI to federal Web managers. The ACSI continues to be used government-wide for both online and offline measures of customer satisfaction (personal communication by Bernie Lubran, ForeSee Results, Inc., May 1, 2007). Aggregate results for all government use of the ACSI, offline and online, are released every December by the University of Michigan [29-31].

NLM and NCI implemented the ACSI on several websites in 2003, taking advantage of the newly available contract providing the ACSI for measuring federal websites. In 2004, NLM and NCI staff shared their ACSI experience and survey results with the broader NIH Web community. This community, represented by a group known as the NIH Web Authors Group, was polled about their interest in participating in a trans-NIH project using the ACSI as a common online survey method.

The Web Authors Group members indicated strong interest, and as a result, a team of co-principal investigators self-organized to develop an evaluation plan and funding proposal. In mid-2004, a proposal was submitted to the NIH Evaluation Set-Aside Program and was approved for funding beginning in September 2004. The NIH Evaluation Branch [32] administers the Evaluation Set-Aside Program [33] that provides funds to evaluate programs and services at NIH. The US Department of Health and Human Services (HHS) “sets aside” funds each year for evaluation; institutes can then competitively apply for those funds. For NIH Web services, the Evaluation Branch funds several types of evaluation, depending on applications received. These have included feasibility studies, surveys (ACSI and others), usability, focus groups, user interviews, and measures of Internet connectivity.

The project was noteworthy because it was the first time that a broad cross-section of NIH organizations used the same method to evaluate websites. The implementation of website evaluations, as well as an external evaluation of the project, was designed and coordinated by a trans-NIH team of senior professionals. At peak participation, the project included 18 (of 27) NIH institutes and centers and 13 offices of the Office of the NIH Director, and 60 separate ACSI website licenses. See Table 1 for a list of participating NIH organizations and Multimedia Appendix 1 (Appendix A) for a list of specific websites.

**Table 1:** NIH organizations participating in the trans-NIH ACSI project (See Multimedia Appendix 1 [Appendix A] for a list of the specific websites participating in the project).

Institute/Center/Office		No. of ACSI Licenses
**Institute/Center**		
	National Cancer Institute	7
	National Eye Institute	1
	National Human Genome Research Institute	1
	National Heart, Lung, and Blood Institute^*^	6 (5)^†^
	National Institute of Allergy and Infectious Diseases	1
	National Institute of Arthritis and Musculoskeletal and Skin Diseases	1
	National Institute on Drug Abuse	2
	National Institute on Deafness and Other Communication Disorders	3
	National Institute of Dental and Craniofacial Research	1
	National Institute of General Medical Sciences	1
	National Institute of Mental Health	1
	National Library of Medicine	7
	Center for Information Technology^*^	7 (3)^†^
	National Center for Complementary and Alternative Medicine	1
	Fogarty International Center	1
	National Institute on Aging	1
	National Institute of Diabetes and Digestive and Kidney Diseases	1
	National Institute of Environmental Health Sciences	1
Total = 18		44 (39)^†^
**Offices Within the NIH Office of the Director (OD)**		
	Office of Animal Care and Use	1
	Office of Communications and Public Liaison	2
	Office of Extramural Research	2
	Office of Electronic Research and Reports Management	1
	Office of Human Resources	1
	Office of Research Services	1
	Office of Research Facilities	1
	Office of Rare Diseases	2
	Office of Intramural Research Continuing Medical Education	1
	Office of Dietary Supplements	1
	Office of Technology Transfer	1
	Office of Science Policy/Office of Science Education	2
	Office of Science Policy and Planning^*^	1
Total = 13		17

^*^These NIH institutes and centers reallocated licenses to other websites or absorbed some license months into existing active licenses.

^†^Number of ACSI licenses allocated, with actual number of licenses used in parentheses.

The trans-NIH ACSI evaluation project lasted for two years, from September 2004 until September 2006, with initial and supplemental funding totaling US $1.5 million from the NIH Evaluation Set-Aside Program. This funding was for outside contracting of the ACSI survey implementation, offered by ForeSee Results Inc. [34], through the Federal Consulting Group / US Department of the Treasury [35], and for an outside evaluation conducted by Westat, Inc. The ACSI survey licenses cost US $18,000 per website, for a total of US $1.275 million (the US $18,000 per site was considered competitive or less expensive for the value added compared to other survey options). The overall project evaluation by Westat, Inc. cost US $225,000. The contractors worked closely with the NIH co-principal investigators and leadership team and the participating NIH organizations.

This paper presents the results of the overall project evaluation that was concluded in fall 2006.

## Methods

The core purpose of the project evaluation was to assess the value of using the ACSI to the participating NIH organizations, identify any NIH-wide benefits of the ACSI, ascertain any additional or new understanding about the NIH Web presence resulting from the ACSI, and evaluate the process of implementing an enterprise-wide approach.

It is important to note that the purpose was not to evaluate the ACSI itself as a stand-alone online survey methodology and/or as compared to other Web evaluation methods. The emphasis in this study was on the process of trans-NIH collaboration on Web evaluation, which was and still is unprecedented in scale. The ability to do an experimental study was confounded in part because websites started and ended their participation at variable times and because many websites did not participate long enough to go through a complete redesign cycle. Also, the emphasis of the study was not to increase ACSI customer satisfaction scores per se but to increase the familiarity of Web teams with use of online surveys as part of website evaluation. Finally, as will be noted in the discussion, the actual change in measured ACSI satisfaction scores when available was, in most cases, not statistically significant. For all these reasons, this project is properly viewed as an observational process study and not an experimental study.

### The ACSI Methodology

The core ACSI methodology was developed by Professor Claes Fornell, Director of the National Quality Research Center, University of Michigan Business School, and is offered as an online service by ForeSee Results, Inc. of Ann Arbor, Michigan [36,37]. The ACSI method uses multiple regression analysis to link questions on key elements driving customer satisfaction with questions on overall customer satisfaction that are in turn linked to questions on future customer behavior. All standardized questions are framed using a 10-point Likert scale. The standardized questions cover the following areas: Elements that Drive Customer Satisfaction (ie, questions covering content, functionality, image, look and feel, navigation, search, privacy, and site performance); Composite Satisfaction (three questions); Future Behavior (ie, three questions covering likelihood to return, likelihood to recommend, likelihood to use as a primary resource).

Table 2 provides a complete list of the standardized ACSI questions. See Multimedia Appendix 2 for illustrations of the ACSI data reporting structure and analytical framework.

**Table 2:** Standardized questions used in the American Customer Satisfaction Index (ACSI) survey methodology

Category	Question^*^
	**Please rate the following on a 10-point Likert scale.**
	**1 = poor, 10 = excellent**
**Site Performance**	Speed of loading the page on this site? Consistency of speed on this site? Reliability of site performance on this site?
**Search**	Usefulness of search results on this site? Provides comprehensive search results on this site? Organization of search results on this site? Search features help you narrow the results on this site?
**Privacy**	Ability to limit sharing of your personal information on this site? Amount of personal information you are asked to submit on this site? Site’s commitment to protecting your personal information?
**Navigation**	Number of steps to get where you want on this site? Ability to find information you want on this site? Clarity of site map or directory? Ease of navigation on this site?
**Look and Feel**	Ease of reading this site? Clarity of site organization? Clean layout on this site?
**Functionality**	Usefulness of the information provided on this site? Convenience of the information on this site? Ability to accomplish what you wanted to on this site?
**Content**	Accuracy of information on this site? Quality of information on this site? Freshness of content on this site?
	**1 = very low, 10 = very high**
**Satisfaction**	What is your overall satisfaction with this site? How well does this site meet your expectations? How does this site compare to your idea of an ideal Website?
	**1 = very unlikely, 10 = very likely**
**Primary Resource**	How likely are you to use this site as your primary resource for health information?
**Recommend**	How likely are you to recommend this site to someone else?
**Likelihood to Return**	How likely are you to return to this site?

^*^These standardized questions are taken from the ACSI online customer survey as used in this study.

In addition to standardized questions, the ACSI methodology allows for the inclusion of questions customized to specific client needs. Custom questions can have flexible formats, ranging from multiple-choice to open-ended.

Typical custom questions used in the NIH project included topics such as frequency of visits (eg, daily, weekly, monthly, first time); customer role (consumer, health provider, researcher, etc); primary purpose for visiting the website; primary means of finding the site; type of information being sought; demographics (age, gender, race/ethnicity, etc); results of query or search; use of the information found; and open-ended questions focusing on a site’s strengths and weaknesses.

The ACSI survey used randomized selection with pop-up presentation of the survey. The sampling rate is set as a function of website traffic volume and estimated response rate, in order to obtain about 300 complete responses per 6-week reporting period. The typical response rate for participating NIH websites was about 5% (range of about 3% to 7%), and the sampling rate varied between a few percent (or less, the lowest being 0.1%) for the busiest sites to 100% for the low-traffic sites. The ACSI, like all online survey methods, can be problematic for very low traffic sites (see later discussion).

 The GSA selected the ACSI in 1999 through a competitive procurement process for use by any interested government agencies. The Federal Consulting Group of the US Department of the Treasury now coordinates the government’s contract with ForeSee Results and interagency agreements between the Federal Consulting Group and agencies using the survey. The Federal Consulting Group also secures multi-year approval from OMB for the use of the ACSI survey by any federal agency. Under the requirements of the Paperwork Reduction Act of 1995, OMB must approve each collection of information by a federal agency (including customer satisfaction surveys) before it can be implemented. As part of its approval of the ACSI, OMB also provides expedited clearance of custom questions that are submitted in conjunction with the ACSI. If the OMB clearance through the Federal Consulting Group were not in place, each agency would need to allow several months to obtain the same clearance for each survey. By handling contracts and coordinating OMB clearances, the Federal Consulting Group greatly streamlines the process of survey implementation for participating federal agencies such as NIH.

### Evaluation Methodology

The major evaluation component of the trans-NIH ACSI project was, in effect, an “evaluation of the evaluation,” with greatest emphasis on the overall impact and utility of the ACSI at the website, organizational, and trans-NIH levels. Of the total project contracting budget of US $1.5 million, about US $225,000 was allocated to evaluation.

The evaluation contractor, Westat, Inc., was engaged throughout the project, worked closely with the NIH leadership team, and attended quarterly trans-NIH meetings with staff from participating websites.

The major components of the project evaluation strategy included the following. At the outset of the study, baseline website profiles were completed for all sites participating in the evaluation. These profiles were established in order to provide a baseline understanding of each site. The profiles were based on self-reported measures by website teams and coding of site characteristics (including website purpose, users, traffic levels, etc).

At the beginning and end of the study, email surveys of participating website teams were conducted. A total of 51 websites completed both the before and after surveys. The response rate for the final Web team survey was 51 out of 55 that implemented the ACSI, or 93%. Also at the beginning and end, the evaluation contractor interviewed a representative cross-section of website staff. Staff from about one third of the websites were interviewed one or more times. Teams were selected for interviewing so as to be representative of website size, purpose, and experience using the ACSI.

During the course of the study, ForeSee Results debriefing meetings with website teams were observed by the evaluation contractor. ForeSee Results, the ACSI contractor, held quarterly meetings, mostly by teleconference, with participating Web teams to discuss survey results and analysis. The NIH evaluation contractor observed a cross-section of these meetings. The evaluation contractor also observed discussions at quarterly trans-NIH ACSI meetings. The trans-NIH leadership team convened quarterly meetings for participating NIH staff to discuss progress, interim results, and lessons learned. ForeSee Results, Westat, and the Federal Consulting Group typically attended these quarterly meetings and gave brief presentations, fielded questions, and engaged in discussion as appropriate. The evaluation contractor also observed discussions at biweekly meetings of the trans-NIH leadership team.

Finally, in addition to the primary data collection listed above, the evaluation contractor had the benefit of secondary data, including quarterly reports on government-wide and private sector ACSI customer satisfaction results. These data were used to track performance of NIH websites and benchmark them against government and private sector websites with similar functions. Multimedia Appendix 3 includes all ACSI quarterly reports on overall ACSI survey results, from inception through March 2007, for federal agencies participating in the e-government satisfaction index based on the ACSI.

The completeness and robustness of the overall project evaluation strategy is illustrated in Table 3 and by specific website in the matrix included in Multimedia Appendix 1 (Appendix A). Multimedia Appendix 1 (Appendix B) also includes copies of the initial and final website staff survey instruments and the initial and final website staff interview instruments.

**Table 3:** Evaluation methods and data sources for the trans-NIH ACSI project

Method/Data Source	Primary Content	Planned Coverage (actual n)
**Review of secondary data**		
Website review	Coding of a variety of website characteristics	All sites (61)
ForeSee pre-implementation worksheets	Coding of team’s responses to pre-implementation questions	All sites (48)
ACSI data for sites generating sufficient response for model data	Satisfaction results per quarter	All sites collecting data during evaluation period: Q4 2004 (8) through Q1 2006 (42)
ACSI site-level data aggregated to NIH level	Standard custom question results Secondary analysis results	All sites using standard custom question; all sites using similar questions (varied by type of analysis)
**Surveys**		
Initial survey	Site background Site evaluation before ACSI Reasons for joining the trans-NIH ACSI evaluation	All sites (57)
Final survey	Intermediate outcomes Longer term outcomes Trans-NIH benefits	All sites (51)
**Interviews**		
Initial in-depth interview (primary focus: processes)	Implementation process Receipt and use of ACSI results Trans-NIH benefits	Subset of sites (14 in 2005; 6 in 2006)
Final in-depth interview (primary focus: outcomes)	Intermediate outcomes Longer term outcomes	Subset of sites (20)
Final brief interview	Benefits of ACSI use without full activities and data for full model	Subset of sites with less ACSI experience (5)
**Observations**		**Coverage(number of meetings)**
Observation of implementation and feedback meetings	How teams: Implemented ACSI Received and reacted to feedback	Sample of meeting types – implementation, initial feedback, follow-up feedback (15)
Observation of trans-NIH meetings	Attendee questions and issues Discussion topics Case studies	All trans-NIH meetings (5)
Observation of leadership team meetings	Management of trans-NIH effort Perceptions about ACSI use across sites	Biweekly meetings (all meetings during evaluation period)

## Results

The results are presented in relation to the four evaluation objectives:

- Objective 1: Through the offer of an ACSI license at no cost to participants, were Web teams encouraged to use an online customer satisfaction survey?
- Objective 2: What was the perceived value to the Web teams of using the ACSI?
- Objective 3: Did broad ACSI use provide additional enterprise-wide NIH benefits?
- Objective 4: Did the trans-NIH ACSI project provide any additional understanding about how NIH websites are used and are meeting NIH communication needs?

### Web Team Participation Rates

Prior to the trans-ACSI project, only a handful of NIH Web teams were using online customer satisfaction surveys of any type. Three NIH organizations were using the ACSI survey method (for a total of seven websites). However a clear majority of NIH website representatives had indicated interest in using the ACSI, if funds permitted.

The central funding of the ACSI project allowed ACSI licenses to be offered to participating websites at no cost to them. The result was that all 60 of the website teams indicating preliminary interest signed up for the project. Of those original teams representing 60 websites, 55 sites actually implemented the ACSI, and 42 of those generated enough data to qualify for regular reporting of satisfaction scores (as of September 2006); 51 website teams completed the final survey; 5 of the original 60 withdrew for various reasons, such as inadequate Web traffic, changing priorities, or insufficient staff or management support. Low-traffic sites were the most likely to withdraw; these included Intranet sites and niche or specialty sites with very small target audiences or narrow topics.

The combination of the free ACSI license plus the significant support from the trans-NIH leadership team, the ACSI contractor, and the quarterly meetings were sufficient to increase NIH participation in the ACSI from seven websites to several times that.

### Perceived Added Value of the ACSI

A major goal was to evaluate the use and value of the ACSI to NIH website teams. Based on the responses of 51 website teams, the respondents overwhelmingly (78%) strongly or somewhat agreed that the custom questions were useful for evaluating the website. About three fifths of respondents strongly or somewhat agreed that the overall customer satisfaction score and the element scores were useful. Respondents rated future behavior scores somewhat less useful, by comparison. A majority of respondents (57%) indicated confidence that scores reflected a website’s strengths and weaknesses.

**Figure 1:**
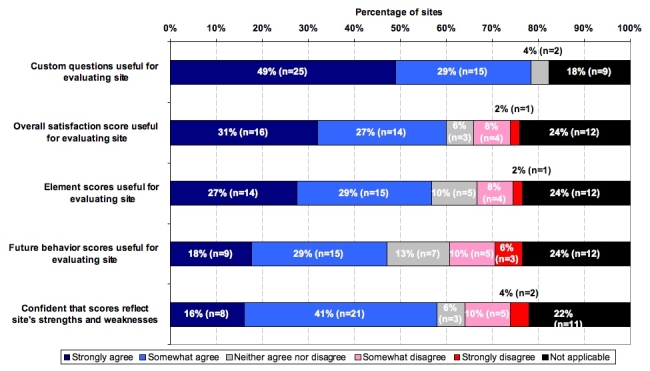
Usefulness of custom questions and ACSI survey scores as reported by participating NIH website teams (Method: Final Website staff survey, n=51)

The website teams were queried on their planned and actual use of the ACSI data. An overwhelming majority of respondents indicated that the ACSI data were more extensively used than planned to provide feedback to their NIH organization, to participate in customer satisfaction benchmarking, and/or to establish program priorities. Some responded that the ACSI data were shared with their website contractor, used to plan for use of additional evaluation methods, and/or used to promote the NIH organization or the website. For example, some NIH organizations used the positive results of their ACSI surveys to favorably promote their resources in annual reports [38], newsletters [39,40], congressional budget justifications [41], and reports to advisory groups [42,43]. A few used ACSI data to establish budget priorities, evaluate contractor performance, or publish or present a paper on the ACSI [44-49] (see also Multimedia Appendix 2 and Multimedia Appendix 4).

**Figure 2:**
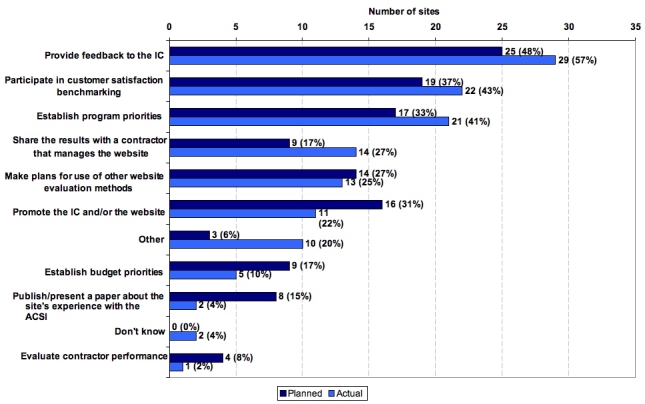
Use of ACSI survey data as reported by participating NIH website teams (Method: Initial Website staff survey, n=52, and final Website staff survey, n=51)

**Figure 3:**
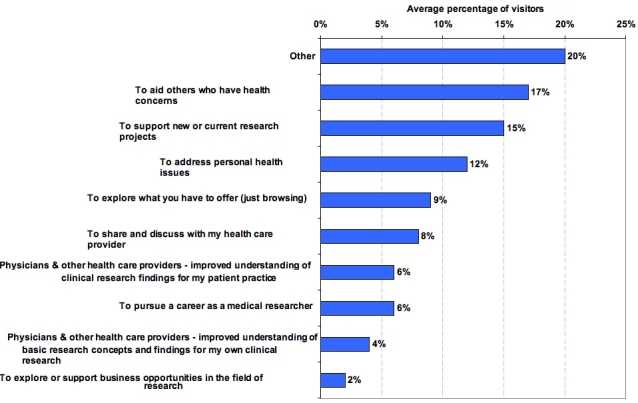
Intended use of information found on website as reported by site visitors (Note: Percentage of visitors indicating each intended use, averaged across all 35 reporting websites; percentages in this case add up to 100% because a standard question with the same response choices was used on all participating sites.) (Method: ForeSee Results Inc. standard custom question)

Website teams were asked what types of site improvements were planned based on what they learned from the ACSI data. The responses covered the breadth of possible website improvements. Almost half of the respondents cited site functionality and navigation. A third or more mentioned improved content, search, overall look and feel, and home page or subpage redesign. A handful mentioned site performance. For further details, see Multimedia Appendix 1 (pp. 3-20, Figure 3-13).

A clear majority (55%, 28/51 sites responding) indicated plans to use the ACSI data for their next website redesign; only a small minority (12%, 6/51) said they were not planning to use the ACSI data in the next redesign. However 25% were not sure (13/51); and one fifth said not applicable (7/51), which could imply that a site redesign was not anticipated.

Website teams were asked whether they were satisfied overall with the use of the ACSI to evaluate their website. The results indicate a roughly four to one balance of those agreeing versus disagreeing—67% (34/51) were strongly or somewhat satisfied, and 18% (9/51) were strongly or somewhat disagreed.

There is some indication that those website teams that actively used the ACSI data during the project were able to increase their overall ACSI customer satisfaction scores. For example, for the 12 websites that showed statistically significant changes in ACSI satisfaction scores, those sites that used the ACSI survey results for continuous website improvement and/or for evaluating effects of website changes tended to have higher satisfaction scores. Those sites that did not use the ACSI survey for those purposes tended to have lower satisfaction scores. These were the only conclusions that could credibly be drawn for the subset of websites with statistically significant changes in satisfaction scores. And those conclusions cannot be generalized to the entire group of participating websites given the absence of statistically significant data and the complexity of the survey and Web design processes.

The generally positive evaluative results need to be balanced by survey results that indicate significant constraints on the ability of Web teams to redesign their sites and to use and continue using the ACSI in the future. When asked about barriers to making changes to their website, almost half (47%, 24/51) of respondents mentioned staff time constraints, and about one quarter (27%, 14/51) noted financial resource constraints. About one fifth cited insufficient calendar time (16%, 8/51) or other reasons (12%, 6/51). Only 9 sites (18%) indicated that there were no barriers; 13 sites (25%) said that the question was not applicable, implying no plans to make major site changes.

### Benefits of Trans-NIH ACSI Use

Another major goal was to evaluate the importance of the trans-NIH ACSI project to NIH as a whole. Based on interviews with a cross-section of Web teams (see Multimedia Appendix 1 for the interview guide) and observations of quarterly meeting discussions, the project greatly increased the focus on measurement of customer satisfaction with NIH websites. The project also encouraged a user-centered approach to NIH website design, improvement, and evaluation. In addition, the project strengthened the network of NIH website professionals and provided opportunities to share experiences and lessons learned and offer staff mentoring.

These results were a direct consequence of making the ACSI licenses available to all participating websites (basically, virtually all interested NIH websites), the nature of the ACSI process, which includes online reporting and periodic analytic support sessions (from ForeSee Results Inc.), and the quarterly trans-NIH meetings. Attendance at the quarterly meetings, held on the NIH main campus, ranged from about 30 to 60 persons and averaged about 45. The majority of websites had one or more team members present at most meetings. The demonstrated level of interest was usually high. Only 3 of 51 teams reporting had not sent a team representative to attend any quarterly meetings. Seven teams reported attendance at all meetings.

The NIH-wide meetings were especially helpful in highlighting contributions and challenges of the ACSI, contributing to an increased awareness and understanding of Web evaluation at NIH, and providing a forum to share lessons learned and identify future directions and opportunities. Web teams shared case studies of specific website experiences with the ACSI, including the use of different types of custom questions. For further details, see Multimedia Appendix 1 (pp. 4-5, Figure 4-1).

The trans-NIH project identified key factors associated with the successful use of the ACSI and with difficulties implementing the ACSI. Factors associated with success included the timing of the surveys with the website redesign cycle—the ACSI survey results were quite useful when planning a website redesign or in evaluating a completed website design. Also important is supportive management that believes in the value of customer surveys and Web evaluation in general. Another success factor is sufficient financial resources (in this project, for staff and website development costs—the cost of the ACSI survey itself was paid through central NIH funds).

Factors related to ACSI implementation difficulties included low-traffic websites. Based on the NIH experience, websites with fewer users, roughly anything less than 50,000 unique visitors per month, need to be monitored carefully to assure that enough completed survey responses are generated in a reasonable period of time. Low-traffic sites tended to include niche or specialty sites as well as Intranet sites, for which very high sampling rates may be needed, thus necessitating the use of persistent cookies to block repeat surveys for the same visitor (see below). Intranet websites with few or no outside users were likely to be problematic. For this NIH project, the Intranet sites had both low traffic and low survey response rates, which means it takes a long time to generate sufficient survey responses. Another factor associated with difficulties is a skeptical staff and/or management attitude toward surveys or Web evaluation in general. Infrequently, a technical issue, such as manual software coding to install the survey pop-up code, contributed to problems. This was the exception, however. The typical experience was easy technical implementation with automated software download and installation.

Another benefit of the trans-NIH approach was the approval of use of persistent cookies on NIH websites. Persistent means that the cookie was left on beyond the time of the initial site visit. The cookies did not collect any personally identifiable information and were used simply to block repeat surveys to the same visitor in a specified period of time (eg, 60 or 90 days). OMB policy generally prohibits use of cookies on federal government websites in order to assure that websites are not used to track individual Web use or collect personally identifiable information [50]. It is difficult to get an exception. But cookies can be used if there is a “compelling need,” if privacy requirements are met, and if the cookie use has “personal approval by the head of the agency.” NIH applied to the Secretary of Health and Human Services, who granted permissions because of the scope of the project and possible burden on the consumer (websites users) from repeat surveys. The cookies were used solely to block site visitors from receiving multiple surveys, and did not contain any personally identifiable information. The cookies helped alleviate concerns about visitors getting “survey weary” or, on the other hand, about a few visitors biasing the results by submitting multiple responses.

### Additional Understanding about NIH Website Visitors

The use of a common survey method across a large number of NIH websites provided an opportunity to gain new insight or clarify earlier impressions about NIH Web visitors. The clear finding is that, overall, through its websites NIH serves multiple audiences with diverse information needs. Many NIH websites have significant percentages of health care provider, scientist, and consumer (including patients, families, and friends) visitors and provide information on a wide range of health, disease, treatment, research, and funding topics.

Based on responses to custom questions asked by 42 websites, students and patients each accounted for about one fifth of visitors, and health care professionals and scientists/researchers each accounted for about one seventh of visitors, on average. The general public (students, patients, families/friends, other) accounted for half to two thirds of visitors based on self-reported visitor roles. For further details, see Multimedia Appendix 1 (pp. 4-14, Figure 4-5).

Very few websites have earlier comparable survey data. For a handful of sites with earlier data, including MedlinePlus, TOXNET, Cancer.gov, and NHLBI, the results were reasonably consistent. The data from this trans-NIH study tended to confirm the trend over the last few years toward a large increase in consumer and general public use of NIH websites, in part due to greater emphasis by NIH on serving the general public’s health information needs as well as needs of health care providers, scientists, and researchers.

Responses to custom questions asked by 31 websites indicated that, on average, the majority of visitors to NIH websites found the information they wanted. In response to the question “Did you find what you were looking for?” visitors responded: yes, 63%; no, 11%; still looking, 26%; partially, 21%; not sure, 9%; not looking for anything specific, 8%.

There were 26 sites using custom questions asking “How did you hear about (or get to) this site?” Across these sites, a search engine was cited most often (42%), followed by a link from another site (17%), and then by a bookmark (16%). For further details, see Multimedia Appendix 1 (pp. 4-16, Figure 4-7).

The trans-NIH leadership team did mandate one common custom question for all participating websites: “How do you plan to use the information you find on this site today?” “You” in this context refers to the website user responding to the online survey. The ACSI contractor, ForeSee Results Inc., included this question on all custom surveys active in January 2006 (with the exception of sites that opted out); 35 sites included this trans-NIH question.

The results indicate a wide range of reported uses of information found on NIH websites. The response options selected indicate that while uses related to research and health practice are significant (about one quarter), there is an even greater emphasis on using information for personal health issues (about one third), whether for oneself or for family and friends. The one third combines the categories of aiding others who have health concerns, addressing personal health issues, and discussing personal health issues with a health care provider. This again reflects the shift in users since the advent of the Web, with a relatively large increase in patients and the public compared to the traditional (pre-Web) NIH core users from the research and health provider communities.

The use of the ACSI survey also provided a basis for benchmarking NIH websites against other federal government and private sector websites. The benchmarking is based on the combined responses to three ACSI standardized questions: “What is your overall satisfaction with this site?”; “How well does this site meet your expectations?”; “How does this site compare to your idea of an ideal Website?”

The customer satisfaction index can range from 0 to 100 based on a weighted average of responses to the three questions (which themselves use a 100-point Likert scale).

The NIH websites as a group scored consistently higher than the federal government and the private sector averages, based on 2006 quarter 4 data for US government websites [51] and 2006 annual data for private sector websites [52]. The average score of 81.3 for participating NIH websites compared very favorably with 73.9 for all federal e-government websites.

It should be noted that the NIH-wide customer satisfaction score varied during the course of the study depending on the number of sites participating and the relative performance of the sites included in the average. At the beginning and after the end of the study period, NIH scores were somewhat higher because some of the weaker performing websites had either not started up or had discontinued participation. The NIH-wide average quarterly score ranged from a high of 79 in 2004 quarter 4 and 81.3 in 2006 quarter 4 to a low of 75.1 in 2006 quarter 1, but in all quarters the NIH average was higher than the comparable federal e-government average score.

NIH average satisfaction scores also outpaced private sector scores. In the news/information sector in 2006 quarter 4, the average for all NIH was 81.6 compared to 72.9 for all e-government websites and 73.0 for all private sector websites using the ACSI. Leading individual websites in the news/information sector included the following, among NIH websites: MedlinePlus (NLM), 86; MedlinePlus in Spanish (NLM), 86; AIDSinfo (NLM), 84; NIDDK (National Institute of Diabetes and Digestive and Kidney Diseases), 84; and Cancer.gov in Spanish (NCI), 83. Among private sector news/information websites, the leaders were as follows: USATODAY.com, 74; CNN.com, 73; ABCNEWS.com, 73; MSNBC.com, 72; and NYTimes.com, 72.

In the portal sector, in 2006 quarter 4, the NIH average satisfaction score was 80.8, the all e-government score, 74.9, and the private sector, 76.0. Leading individual NIH websites in the portal sector included Cancer.gov (NCI), 83; NHLBI (National Heart, Lung, and Blood Institute), 83; Office of Science Education/Office of NIH Director, 82; and NIAMS (National Institute of Arthritis and Musculoskeletal and Skin Diseases), 80. Leading private sector portal websites included Yahoo.com, 76; MSN.com (Microsoft Corp.), 74; and AOL.com (Time Warner Inc.), 74.

While the numeric customer satisfaction scores varied somewhat during the project, the NIH websites as a group scored consistently higher than e-government and private sector averages. The leading NIH websites individually scored significantly higher than the leading private sector websites in their class.

Aside from these global comparisons, it was not possible to conduct drill-down quantitative analyses of impacts on satisfaction scores. This was because, in the first instance, only 12 of the 55 websites implementing the ACSI showed statistically significant changes in satisfaction from start to finish. Second, while the ACSI standardized question responses give some indication of the most highly leveraged Web design changes, no quantitative data were collected on the specific Web changes made, if any, and their relationship to changes in satisfaction. Thus, while qualitative data based on interviews and surveys of Web teams are reported in this paper, drill-down quantitative analyses could not be credibly and validly carried out and, in any event, were beyond the project scope.

## Discussion

This project was the first enterprise-wide ACSI application and probably the largest enterprise Web evaluation project to date in the US government. The project implemented the largest number of ACSI surveys (55) at any one government agency. Other agencies using the ACSI have multiple measures but in smaller numbers; for example, the Centers for Medicare and Medicaid Services are using 20, the US Department of State is using 15, the US Department of Agriculture uses 9, and the US Department of the Treasury uses 8 (personal communication, Ron Oberbillig, Federal Consulting Group, US Department of the Treasury, April 16, 2007).

The trans-NIH ACSI project met all of the original study and evaluation goals—a broad cross-section of NIH websites participated, the trans-NIH project leadership team drew from several NIH organizations and functioned very well for the 2-year project duration, NIH Web staff attendance at quarterly meetings was good to excellent, the project evaluation methodology was well designed and funded and fully implemented, and the evaluation itself was successful in identifying useful information on the site-specific and trans-NIH impacts of using the ACSI as well as assessing the success of the project as a whole.

Multimedia Appendix 5 is a PowerPoint presentation highlighting select evaluation and trans-NIH results, presented at the last trans-NIH meeting to be held as part of the project (October 2006). Multimedia Appendix 4 is a PowerPoint presentation discussing the enterprise-wide approach, presented at the Federal Consulting Group’s ACSI Web Survey Group quarterly meeting (March 2007).

A majority of participating website teams reported significant benefits and new knowledge from the ACSI survey results and from being involved in the overall project process. The more experienced and better funded so-called “power users” among the participating NIH websites were able to use the ACSI as a ready-to-use customer satisfaction metric that provided pre-approved OMB clearance (a major advantage in streamlining the start-up process) and as a tool for incorporating custom questions into the survey in order to identify specific website issues and problems. Power users also employed the ACSI results as a source of information about site visitor demographics and as a means to analyze the satisfaction levels and information retrieval results of visitor subgroups to identify needed site improvements. The power users utilized the ACSI as a source of information for planning any follow-up or parallel work involving additional evaluation methods and as an archive of survey data for future use and analysis in website redesign and information enhancements.

These power users were able to apply the ACSI survey results to benchmark their particular NIH websites against other government and private sector websites and to gain insights about and opportunities for improving their Web presence through site-specific feedback. The ACSI results allowed power users to respond more quickly and effectively to the ever-evolving and changing Web environment and to help determine the impact of website changes and evaluate whether Web-based information dissemination programs are performing significantly better or worse over a defined period of time.

As a group, the participating NIH websites performed very well overall against US government and private sector benchmarks. The power user NIH websites—again, typically the larger and more heavily used, staffed, and funded websites—tended to have higher satisfaction scores than other participating websites. These websites also were more likely to use several evaluation methods in order to triangulate results and obtain more complete inferences and interpretations. However, with all NIH websites included, the NIH-wide average satisfaction score exceeded the government-wide average from the beginning of the project until the end.

As a consequence, NIH as a whole, and some individual NIH organizations, received significant positive media coverage of their Web performance during the course of the project [53-57]. Also, NIH received the first ever e-government award from the Federal Consulting Group / US Department of the Treasury—the Customer Performance Achievement Award—conferred by the OMB Administrator for Electronic Government and Information Technology in recognition of the success of the trans-NIH ACSI project.

Websites varied in their ability to implement the ACSI and utilize results. The majority of participating websites were able to implement the ACSI and receive survey results, including satisfaction scores. Some sites were able to implement the ACSI but did not generate sufficient completed surveys to generate satisfaction scores due to low traffic on the website or because the ACSI was implemented too late in the study. However, these sites were able to obtain the results of their custom questions. The ACSI or any other online user survey does not work well with low-traffic websites. It simply takes too long to obtain a minimum sample for statistically significant results.

Due to the large number of websites involved, the trans-NIH project, out of necessity, implemented the ACSI in stages, determined in part by the degree of readiness of each website to participate. This generally meant that the more experienced better-staffed websites (including sites that had been pilot testing the ACSI) fully implemented the ACSI earlier and had more time to collect survey results. Other sites were not ready to implement the ACSI until late in the project. In addition, some sites that dropped out were replaced by others late in the project. The late starters in some cases did not have sufficient time to generate enough completed surveys.

Website teams that used the ACSI the longest tended to be satisfied with and find value in its use, especially for planning site changes and comparing versions of the website before and after revisions or redesigns. Teams with relatively later start dates and/or slow rates of collecting completed ACSI surveys were more likely to be dissatisfied with the ACSI because they did not have sufficient time or opportunity to receive and/or act on ACSI survey results.

Relative inexperience in using the survey may also have been related to perceived value because of the complexity of the survey results. The ACSI, unlike simpler survey methods, generates multidimensional results based on both standardized and custom questions. Segmentation of results, while analytically powerful, can also be daunting to the inexperienced.

In addition to time and experience, other key factors driving successful use of the ACSI or, by extension, other similar online survey methods, based on this project experience include staff and management buy-in, adequate resources, staff training and understanding, the website design cycle, and technical support.

Across all participating NIH websites, the Web teams derived substantially greater value from their custom question data and from segmentation data (breaking out results by specific types of visitors, information seeking goals, demographics, etc), than from the standardized ACSI questions. The custom question data provided many Web teams with valuable insight about visitor profiles and visit characteristics. For example, through cross-correlations between responses to custom and standardized questions, Web teams were able to identify visitor subgroups that were less satisfied and highlight needed website improvements. Many teams also took advantage of having a continuous source of customer feedback for tracking the visitor responses to website improvements implemented in response to ACSI data (as reflected in satisfaction scores).

The ACSI, like all online surveys in the Web environment, has relatively low response rates (typically about 5%, but ranging from 3% to 7%). The ACSI uses random intercepts and several cross-checks to help assure that nonresponse bias is minimized, but the latter is still a concern and warrants greater attention in the academic and survey research communities. NLM, NCI, and NHLBI, three of the participating NIH organizations, had used online surveys for several years prior to the ACSI. The prior surveys placed greater emphasis on the custom questions and less on standardized questions or benchmarking. Comparison of results about site visitors between the prior surveys and the ACSI results for several websites (eg, MedlinePlus, AIDSinfo, and TOXNET at NLM, and the NHLBI website) indicated that similar results were obtained between the earlier surveys and the ACSI surveys [22,23, personal communication, Cindy Love, April 30, 2007; personal communication, Mark Malamud, October 9, 2007]. This suggests that the ACSI survey results can be considered reasonably valid, and not unduly affected by non-response bias, unless there are undetected sources of non-response bias affecting all surveys over an extended time frame.

However, it is best not to rely too heavily on any one Web evaluation methodology. As noted earlier, a multidimensional approach is warranted and has been adopted by the more experienced better-funded NIH websites. The survey of NIH Web teams indicates that 21 of the participating teams practise, to varying degrees, a multidimensional approach. In addition to the ACSI, during the time of the trans-NIH project, 19 of the 21 websites also used Web log software, 18 used usability testing, 11 used expert or heuristic reviews, 4 used other types of surveys, 4 used focus groups, 3 used audience measurement and profiling, and 1 indicated other.

### Conclusions

The trans-NIH leadership team believed in the importance of Web evaluation going into the trans-NIH ACSI project and was motivated to make the ACSI available to a broad group of NIH websites. The hope was to significantly increase the use of online customer surveys, the ACSI being a particular variant of the general class, within the NIH Web community. Further, the hope was that the project would not only increase NIH staff understanding of the value of this and other forms of Web evaluation, but also strengthen the management and financial support for Web evaluation at NIH.

The project was successful in increasing the use of and interest in online surveys and enhancing the understanding of the strengths and limitations of such surveys. A majority of participating websites found considerable added value in the survey process and results. However, many of the Web teams gave a clear indication in the project evaluation survey that notwithstanding the benefits, it was uncertain or questionable whether they would be able to fund the modest (US $20,000 or so per year per website) cost of renewing the ACSI from their own funds if central NIH funds were no longer available. As it turned out, central funding was not continued beyond the 2-year project life of this trans-NIH project, and each participating NIH website had to make its own decision whether to continue, and, if so, find its own funding to do so. The result was that only about one quarter of the NIH websites renewed their ACSI license, and half of those renewals were the early experimenters who had been using the ACSI for the longest time.

For this trans-NIH project, the US $18,000 survey license fee per website was considered to be competitive with other online survey options in terms of cost and to offer a better value added per dollar when considering the other benefits of the ACSI. For those websites wishing to continue, the FCG and ForeSee Results offered an ACSI “lite” version at US $15,000 (compared to US $25,000 for full service), but even at that price point there were relatively few renewals.

The NIH was fortunate to have the support of the Evaluation Set-Aside Program for the trans-NIH ACSI project. Much was learned, and many websites received significant added value, in their own estimation. But this was an experiment, not an ongoing operational activity. Without central funding, only the more experienced better-resourced larger websites, for the most part, continued with the ACSI.

Thus, a final lesson learned from the trans-NIH ACSI project experiment is the tenuous nature of Web evaluation in the age of e-government, when OMB and departmental policies are placing ever greater emphasis on Web-based delivery of government information and services. A parallel commitment to adequate evaluation of those Web-based activities may well be needed in order to help assure that the potential of the Web and other information technologies to improve customer and citizen satisfaction is fully realized.

## Multimedia Appendix 1

Trans-National Institutes of Health (NIH) American Customer Satisfaction Index (ACSI) Web Site Evaluation. Final Report.

## Multimedia Appendix 2

Siegel ER, Wood FB. Customer satisfaction and performance metrics. Paper presented at: International Council for Scientific and Technical Information; January 22, 2007; London, UK.

## Multimedia Appendix 3

American Customer Satisfaction Index E-Government Satisfaction Index Quarterly Reports (Archived Links)

## Multimedia Appendix 4

Feldman S, Love CB. NIH’s enterprise approach to measuring customer satisfaction. Paper presented at: ACSI User Group Meeting; March 20, 2007; Washington DC.

## Multimedia Appendix 5

Crafts J. Summary briefing on the goals, methods, and results of the Trans-NIH evaluation. Report presented at: Trans-NIH ACSI Meeting, October 4, 2006, Bethesda, MA.

## Abbreviations

ACSI: American Customer Satisfaction Index
GSA: Government Services Administration
NCI: National Cancer Institute
NHLBI: National Heart Lung and Blood Institute
NIH: National Institutes of Health
NLM: National Library of Medicine
OMB: Office of Management and Budget

## References

[1] Lacroix Eve-Marie, Mehnert Robert (2002). The US National Library of Medicine in the 21st century: expanding collections, nontraditional formats, new audiences. Health Info Libr J.

[2] Hesse Bradford W, Nelson David E, Kreps Gary L, Croyle Robert T, Arora Neeraj K, Rimer Barbara K, Viswanath Kasisomayajula (2005). Trust and sources of health information: the impact of the Internet and its implications for health care providers: findings from the first Health Information National Trends Survey. Arch Intern Med.

[3] Bright Mary Anne, Fleisher Linda, Thomsen Chris, Morra Marion E, Marcus Al, Gehring Wendy (2005). Exploring e-Health usage and interest among cancer information service users: the need for personalized interactions and multiple channels remains. J Health Commun.

[4] Grama Lakshmi M, Beckwith Margaret, Bittinger Wayne, Blais Diana, Lollar Cindy, Middleswarth Anne, Noone Marianne, Price Deborah, Quint-Kasner Sharon, Shields Victoria, Wright Lawrence W (2005). The role of user input in shaping online information from the National Cancer Institute. J Med Internet Res.

[5] McCray A T, Dorfman E, Ripple A, Ide N C, Jha M, Katz D G, Loane R F, Tse T (2000). Usability issues in developing a Web-based consumer health site. Proc AMIA Symp.

[6] Wood FB, Siegel ER, LaCroix EM, Lyon BL, Benson DA, Cid V, Fariss S (2003). A practical approach to e-government Web evaluation. IT Professional.

[7] Sterne J (2002). Web Metrics: Proven Methods for Measuring Website Success.

[8] Bader Judith L, Theofanos Mary Frances (2003). Searching for cancer information on the internet: analyzing natural language search queries. J Med Internet Res.

[9] Redalen A, Miller N (2000). Evaluating website modifications at the National Library of Medicine through search log analysis. D-Lib Magazine.

[10] Miller Naomi (2007). Analysis of user messages to MedlinePlus.gov. J Med Libr Assoc.

[11] Wood Fred B, Benson Dennis, LaCroix Eve-Marie, Siegel Elliot R, Fariss Susan (2005). Use of Internet audience measurement data to gauge market share for online health information services. J Med Internet Res.

[12] National Library of Medicine MedlinePlus Quality Guidelines. 2006. NLM/NIH.

[13] Miller N, Lacroix E M, Backus J E (2000). MEDLINEplus: building and maintaining the National Library of Medicine's consumer health Web service. Bull Med Libr Assoc.

[14] Wilson F L, Baker L M, Brown-Syed C, Gollop C (2000). An analysis of the readability and cultural sensitivity of information on the National Cancer Institute's Web site: CancerNet. Oncol Nurs Forum.

[15] International Council on Active Aging (2005). NIHSeniorHealth.gov: empowering older adults with health information. J Act Aging.

[16] Boyer Celia, Geissbuhler Antoine (2005). A decade devoted to improving online health information quality. Stud Health Technol Inform.

[17] Office of Management and Budget Government Performance Results Act of 1993. whitehouse.gov.

[18] Office of Management and Budget Circular No. A-130 Revised. Management of Federal Information Resources. whitehouse.gov.

[19] Office of Management and Budget (2002). Part IX: Guidelines for Ensuring and Maximizing the Quality, Objectivity, Utility, and Integrity of Information Disseminated by Federal Agencies; Notice; Republication. Section 515 of the Treasury and General Government Appropriations Act for Fiscal Year 2001: Public Law 106-554. Federal Register.

[20] Web Managers Advisory Council, Interagency Committee on Government Information (2004) Recommended Policies and Guidelines for Federal Public Websites. Final Report of the Interagency Committee on Government Information. Submitted to The Office of Management and Budget June 9, 2004. usa.gov.

[21] Memorandum for the Heads of Executive Departments and Agencies (2004). December 17, 2004. Policies for Federal Agency Public Websites.

[22] National Library of Medicine’s MedlinePlus Survey Results 2001 NLM/NIH.

[23] MedlinePlus Survey Results 2003 NLM/NIH.

[24] Fornell CG, Johnson MD, Anderson EW, Cha J, Bryant BE (1996). The American Customer Satisfaction Index: nature, purpose and findings. J Mark.

[25] Stowers GNL (2004). Measuring the Performance of E-Government.

[26] Freed L (2003). American Customer Satisfaction Index E-Government Satisfaction Index.

[27] Yao ML (2000). The President’s Management Council: An Important Management Innovation.

[28] US General Services Administration Vice President Launches First Governmentwide Customer Satisfaction Survey. 1999 May 26. gsa.gov.

[29] Fornell C ACSI Commentary: Federal Government Scores. 2004 Dec 14. theacsi.org.

[30] Fornell C ACSI Commentary: Federal Government Scores. 2005 Dec 15. theacsi.org.

[31] Fornell C ACSI Commentary: Federal Government Scores. 2006 Dec 15. theacsi.org.

[32] Office of Portfolio Analysis and Strategic Initiatives Evaluation Branch. OPASI/NIH.

[33] Office of Portfolio Analysis and Strategic Initiatives Evaluation Branch. NIH Evaluation Set-Aside Program. OPASI/NIH.

[34] ForeSee Results home page ForeSee.

[35] US Department of the Treasury Federal Consulting Group home page. fcg.gov.

[36] Fornell C, Van Amburg D, Morgeson F (2005). The American Customer Satisfaction Index at Ten Years, ACSI 1994-2004. A Summary of Findings: Implications for the Economy, Stock Returns and Management.

[37] American Customer Satisfaction Index Methodology Report (2005).

[38] National Institutes of Health, National Library of Medicine (2005). Programs and Services FY 2005.

[39] National Cancer Institute (2005). NCI Web site rated best e-government main site. NCI Cancer Bull.

[40] National Institute of Diabetes and Digestive and Kidney Diseases (2005) NIDDK health information Web sites rate high in customer satisfaction. In: NIDDK Director's Update (June 15, 2005). NIDDK/NIH.

[41] National Library of Medicine FY 2006 Budget. NLM/NIH.

[42] Wood F, Lacroix EM (2004). NLM User Studies. In: Minutes of the Board of Regents February 10-11, 2004.

[43] National Institute of Dental and Craniofacial Research Satisfaction with NIDCR Web site compares favorably with other government and private sector sites. In: Director's Report to the National Advisory Dental and Craniofacial Research Council. June 2005. NODCR/NIH.

[44] Fariss S, Love CB (2004). Now you’ve built it…Does it work?. Paper presented at: E-Government Citizen Satisfaction Summit; September 21,.

[45] Feldman S (2004). Evaluation of NCI Website. Paper presented at: E-Government Citizen Satisfaction Summit; September 21,.

[46] Martin KL, Feldman S Who’s behind the screen & what are they looking for?. Paper presented at: National Cancer Institute HINTS Data Users Conference; January 20, 2005, St. Pete Beach, FL.

[47] Feldman S Demonstrating the value, impact and return on investment from evaluating Web-based services: An overview of strategies. Paper presented at: Digital Government Institute. Website Evaluation: Making Web Services Integral to Agency Mission; November 7-8, 2005; Washington, DC.

[48] Rodrigues D CSI survey results and how they are used. Paper presented at: Healthfinder Steering Committee Meeting; December 11, 2006; Rockville, MD.

[49] Cadden C How physicians are using the federal Treatment Guidelines: Data from AIDSinfo's online American Customer Satisfaction Index survey. Paper presented at: American Conference for the Treatment of HIV (ACTHIV); May 31 to June 3 2007; Dallas, TX.

[50] Office of Management and Budget Memorandum for the Heads of Executive Departments and Agencies. June 22, 2000. Privacy Policies and Data Collection on Federal Web sites. whitehouse.gov.

[51] Freed L American Customer Satisfaction Index E-Government Satisfaction Index. Ann Arbor, MI: ForeSee Results: 2006 Dec 15. http://www.fcg.gov/documents/acsi-results-12-2006.pdf.

[52] Freed L (2006). American Customer Satisfaction Annual E-Business Report.

[53] Chabrow E (2004). High marks for government jobs and health sites: the University of Michigan’s quarterly customer-satisfaction survey gives some government sites higher scores than commercial sites. InformationWeek.

[54] Michael S (2004). Consumers wowed by Web sites. FCW.com.

[55] Pulliam D Customer satisfaction with federal Web sites nudges up a notch. GovernmentExecutive.com. 2005 Jun 13.

[56] Perera D Health sites spur satisfaction. Government Health IT. 2005 Jun 14.

[57] NIH Web sites win customer satisfaction (2007). http://nihrecord.od.nih.gov/pdfs/2007/01122007_Record.pdf.

