# Supplementary material for: Web Evaluation at the US National Institutes of Health: Use of the American Customer Satisfaction Index Online Customer Survey
Source: J Med Internet Res. 2008 Feb 15;10(1):e4. doi: 10.2196/jmir.944 (PMC2483849; doi:10.2196/jmir.944)
Supplement: Supplementary file 1 [file jmir_v10i1e4_app1.pdf]

Multimedia Appendix 1 to
Wood FB, Siegel ER, Feldman S, Love CB, Rodrigues D, Malamud M, Lagana M, Crafts J
Web Evaluation at the US National Institutes of Health: Use of the American Customer Satisfaction Index Online Customer Survey
J Med Internet Res 2008;10(1):e4
<URL: http://www.jmir.org/2008/1/e4/>

Trans-National Institutes of Health (NIH) American Customer Satisfaction Index (ACSI) Web Site Evaluation

Final Report

September 2006

EB # 05-605-S-NLM

Prepared for:

NIH Leadership Team

Prepared by:

WESTAT
1650 Research Boulevard
Rockville, Maryland 20850-3195

WESTAT
AN EMPLOYEE-OWNED RESEARCH CORPORATION

TABLE OF CONTENTS

Chapter		Page
	EXECUTIVE SUMMARY	xi
	Background	xi
	Method	xii
	Results and Conclusions	xiii
	Site Level	xiii
	Trans-NIH Level	xv
	Recommendations	xvii
1	INTRODUCTION	1-1
	1.1 Project Background	1-1
	1.2 Scope of the Evaluation	1-3
	1.2.1 Time Frame for the Evaluation	1-5
	1.2.2 Participating Web Sites	1-7
	1.3 Scope of the Final Report	1-8
2	EVALUATION METHODS	2-1
	2.1 Data Collection Methods and Sources	2-1
	2.1.1 Review of Secondary Data	2-3
	2.1.2 Surveys	2-3
	2.1.3 Interviews	2-4
	2.1.4 Observations of Program Processes	2-5
3	SITE-LEVEL RESULTS	3-1
	3.1 How NIH Web Sites Have Used the ACSI	3-1
	3.1.1 Perceived Effectiveness of ACSI Start-up Activities	3-3
	3.1.2 Perceived Value of ACSI Feedback	3-4
	3.1.3 Perceived Value of Review Meetings and Satisfaction Research Analysts	3-6
	3.1.4 Understanding Segmentation, ACSI Results, and Use of Results for Planning	3-8
	3.1.5 Level of Web Site Teams’ Experience With the ACSI	3-9

TABLE OF CONTENTS (continued)

Chapter			Page
3.2	Intermediate Outcomes Associated With ACSI Use		3-11
	3.2.1	Perceived Benefits of ACSI Use	3-12
		3.2.1.1 Overall Satisfaction With ACSI Use	3-12
		3.2.1.2 Met and Unmet Expectations	3-12
		3.2.1.3 Usefulness of Custom Questions in Web Site Evaluation	3-14
		3.2.1.4 Perceived Usefulness of ACSI Score Data	3-16
		3.2.1.5 Satisfaction With Segmentation	3-17
	3.2.2	Reported Uses of ACSI Data	3-18
		3.2.2.1 Planned vs. Actual Use of ACSI Data	3-18
		3.2.2.2 Use of ACSI Data in Next Web Site Redesign	3-20
		3.2.2.3 Use of Data to Make Web Site Changes	3-21
		3.2.2.4 Barriers to Making Planned Web Site Changes	3-22
		3.2.2.5 Use of Benchmarking Data	3-23
	3.2.3	Impact of ACSI Use on Web Site Teams' Use of Other Site Evaluation Tools and Methods	3-24
3.3	Longer Term Outcomes		3-26
	3.3.1	ACSI Satisfaction Score Changes	3-27
		3.3.1.1 Score Changes Related to Implemented Site Changes	3-27
		3.3.1.2 Score Changes Related to Other ACSI Activities.	3-28
		3.3.1.3 Examples of Web Site Improvements That Led to Higher Satisfaction Scores	3-28
	3.3.2	Impact of ACSI Use on Management Approach	3-29
	3.3.3	Impact of Web Sites Using ACSI	3-30
	3.3.4	Desire of Participating Web Site Teams to Continue Using the ACSI	3-32
3.4	Web Sites That Were Less Successful in Using the ACSI		3-35
	3.4.1	Timely Collection of 300 Completed ACSI Surveys	3-38

TABLE OF CONTENTS (continued)

Chapter				Page
		3.4.2	Timing of License Period With Web Site Development/ Redesign Schedule	3-40
		3.4.3	Fit of Web Site Team and SRA	3-41
		3.4.4	"Niche" or Specialty Web Sites	3-42
		3.4.5	Lack of Support From IC Staff or Management	3-42
4	TRANS-NIH RESULTS			4-1
	4.1	Benefits and Drawbacks of the Trans-NIH Program		4-1
		4.1.1	Meeting Attendance	4-3
		4.1.2	Benefits of Attending Meetings	4-3
		4.1.3	Benefits of Participating in the Trans-NIH Project	4-3
		4.1.4	Benefits of Experienced/Knowledgeable Web Site Team Members and Teams	4-5
		4.1.5	Drawbacks of Participating in the Trans-NIH Project	4-5
	4.2	Identifying and Resolving Common Issues		4-6
		4.2.1	Search Element Scores	4-6
		4.2.2	Common Barriers to Improvement Across Sites	4-6
		4.2.3	Directing Visitors to Appropriate Information Sources	4-7
	4.3	Demonstrating the Contribution of Web Sites to NIH Missions		4-8
		4.3.1	Use of Custom Questions Across Sites	4-8
		4.3.2	Important Message About the Value of Evaluation	4-16
	4.4	Trans-NIH Benefits for the Sites		4-17
		4.4.1	Automatic OMB Clearance	4-17
		4.4.2	Collection of Site-Specific Qualitative Feedback	4-17
		4.4.3	Increased Interest in Customer-Centered Design	4-18
		4.4.4	Increased Interest in Web Analytics	4-18
		4.4.5	Other Uses of ACSI Data	4-18
	4.5	Impacts Across the U.S. Government		4-19
		4.5.1	Expanding Knowledge About Customer Satisfaction Measurement	4-19
		4.5.2	Benchmarking Across Agencies	4-20
		4.5.3	Best Practices	4-21
		4.5.4	Change in Persistent Cookie Policy	4-21

TABLE OF CONTENTS (continued)

Chapter		Page
5	CONCLUSIONS AND RECOMMENDATIONS	5-1
	5.1 Conclusions	5-1
	5.1.1 Trans-NIH Level	5-1
	5.1.2 Web Site Level	5-2
	5.2 Trans-NIH Level Recommendations	5-3
	5.2.1 Trans-NIH Continuation	5-3
	5.2.2 Alternatives to the Leadership Team Role in Guiding Teams Using the ACSI	5-5
	5.3 Web Site-Level Recommendations	5-6
	5.3.1 Web Sites Considering ACSI Use	5-6
	5.3.2 Web Sites Continuing to Use the ACSI	5-9

List of Appendixes

Appendix		
A	SITE TRACKING TABLE	A-1
B	SURVEY AND INTERVIEW INSTRUMENTS	B-1
	Initial Survey for Participants in the NIH	B-1
	Final Survey for Participants in the NIH	B-11
	Initial Interview Questions for Selected Participants (for Teams that Have Completed at Least the First ACSI Feedback Cycle)	B-18
	Final Interview for Selected Participants	B-21

List of Boxes

Box		Page
1	Overview of the ACSI Methodology	1-2
2	Overview of Key Results in Chapter 3	3-2
3	Summary of Key Results in Chapter 4	4-2

TABLE OF CONTENTS (continued)

List of Tables

Table		Page
1-1	Institutes, centers, and offices within the office of the NIH Director participating in the ACSI Trans-NIH Evaluation, with number of licenses issued to each	1-4
2-1	Methods used to evaluate the use of the ACSI at NIH	2-2
3-1	Web site teams' coded comments: Ways in which the ACSI exceeded and fell short of expectations	3-14
4-1	Teams' suggestions for additional standard custom questions	4-11
4-2	Example: ForeSee's recoding for trans-NIH analysis of custom questions (visitor's role)	4-13
4-3	Satisfaction scores for most frequently reported visitor roles	4-14

List of Figures

Figure		Page
1-1	Components of the ACSI	1-2
1-2	ACSI reporting and analysis process	1-6
3-1	Web site team rating of key start-up activities	3-3
3-2	Match between ACSI scores and web site team expectations	3-5
3-3	Web site team comments on value of ACSI findings	3-5
3-4	Site team satisfaction with feedback meetings and SRAs	3-7
3-5	Web site teams' perceived benefits of ACSI score data	3-8
3-6	Web site teams' understanding of ACSI results, segmentation, and planning	3-9
3-7	Reported activities broken out by license term start date	3-10
3-8	Web site teams' overall satisfaction with use of ACSI to evaluate site	3-12

TABLE OF CONTENTS (continued)

List of Figures (continued)

Figure		Page
3-9	Web site teams' judgments of whether ACSI use met their expectations.........	3-13
3-10	Usefulness of custom questions and ACSI scores..	3-15
3-11	Web site teams' satisfaction with segmentation..	3-17
3-12	Web site teams' planned vs. actual use of ACSI data......................................	3-18
3-13	Types of site improvements planned using ACSI data....................................	3-20
3-14	Web site teams' plans for using ACSI data for next redesign...........................	3-21
3-15	Barriers to making changes to site..	3-23
3-16	Reported uses of ACSI data for benchmarking...	3-24
3-17	Impact of ACSI on use of other evaluation methods.......................................	3-25
3-18	Reported evaluation methods used with ACSI (for subset of web site teams using ACSI in combination with other evaluation methods)................	3-26
3-19	Mean satisfaction score changes based on use of ACSI..................................	3-27
3-20	IC and ODO management support for ACSI use..	3-29
3-21	Trends in ACSI scores based on quarter of reporting start..............................	3-31
3-22	Use of ACSI if IC/ODO had to pay license fee...	3-32
3-23	Constraints affecting continued use of ACSI..	3-33
3-24	Satisfaction scores across time for subgroups of web sites based on traffic volume..	3-36
3-25	Satisfaction scores across time for subgroups of web sites based on FTEs......	3-36
3-26	Satisfaction scores across time for subgroups of web sites based on primary audience...	3-37
3-27	Satisfaction scores across time for subgroups based on visit frequency (first-time vs. repeat visitors)...	3-37

4-1	Benefits of trans-NIH meetings...	4-4
4-2	Standard custom question added to ACSI Survey in 2006..............................	4-9
4-3	ForeSee's standard custom question results: Intended uses of site information..	4-10
4-4	Frequency of site visits (across 41 sites)..	4-13
4-5	Role in which visitor comes to site (across 42 sites).......................................	4-14
4-6	Whether site visitors found the information they were looking for.................	4-15
4-7	How visitors were brought to site (across 26 sites)..	4-16
4-8	Comparison of NIH and e-Government satisfaction indexes during evaluation period..	4-20

EXECUTIVE SUMMARY

Background

In October 2004, the National Institutes of Health (NIH) funded an effort across Institutes, Centers, and Offices of the Office of the NIH Director (subsequently referred to as ICs and ODOs) to evaluate the use of the American Customer Satisfaction Index (ACSI) as a standard evaluation tool for 60 NIH web sites. The ACSI is an online customer satisfaction survey methodology widely used across government to meet the Federal directive to measure citizen satisfaction with e-government.

The NIH Office of Evaluation (now the Evaluation Branch within the Office of Portfolio Analysis and Strategic Initiatives) awarded funding of $1.22 million in 2004 to cover the 18-month trans-NIH evaluation project. A group of seven co-principal investigators (the NIH Leadership Team, or LT) representing NIH ICs and ODOs managed all components of the project. ForeSee Results supported ACSI implementation, and Westat served as the evaluation contractor for the LT. In 2005, supplemental funding of $250,000 extended the project for 6 months.

One goal of the project was to enhance web site evaluation practices for interested NIH web site teams by providing them with an ACSI license. Another goal was to provide additional benefits for NIH above and beyond those at the site level, through simultaneous use of the ACSI across participating teams.

The Westat evaluation focused on two levels of ACSI use. At the level of individual participating teams, the evaluation examined how these teams applied the ACSI as a web site performance metric and the ways these sites benefited. At the trans-NIH level, the evaluation looked at the value added by using the ACSI in a simultaneous and phased approach for web site evaluation across NIH. The project, with its number and diversity of web sites, represents the largest web evaluation effort of its kind for NIH and the Federal government.

By offering an ACSI license, NIH hoped to encourage web site teams to add customer satisfaction measurement to their current web site evaluation methods. For ICs and ODOs, use of a license addresses the requirement of all Federal agencies to measure citizen satisfaction with e-

government. Across the government as well as in the private sector, the ACSI has become the recognized methodology for web site performance measurement.

Method

Data for both levels of the evaluation were captured using a multi-method approach. Westat used the following methods and sources over the evaluation period (October 2004 to May 2006):

- Review of secondary data (participating web sites, worksheets the web site teams completed for ForeSee before implementation of the ACSI, and ACSI data at both the site and aggregate levels);
- Surveys of all participating teams at both the start and end of the evaluation period;
- In-depth interviews with selected teams at the project midpoint and near the end of the evaluation period; and
- Observations of three types of meetings (ForeSee feedback meetings, trans-NIH meetings, and LT meetings).

These methods and sources provided a comprehensive set of quantitative and qualitative data to address the following evaluation goals:

- For participating web sites:
  - Did the ACSI meet the needs and expectations of participating teams?
  - What were the benefits of using a continuous online customer satisfaction survey for site evaluation?
  - What value did the ACSI results provide? Were teams able to turn the results into action steps? What effects did any site changes have on subsequent ACSI scores?
- For NIH:
  - Was there added value when NIH web site teams simultaneously used a common evaluation tool?
  - What types of sites benefited the most from the ACSI license and why? What are the optimal contexts and practices for ACSI use at NIH?
  - Did the evaluation provide any additional understanding about the performance of NIH web sites?

Results and Conclusions

Site Level

The ACSI filled a very large gap for most NIH web site teams that did not have internal resources for evaluation and/or previous experience in measuring customer satisfaction. Two-thirds of the teams indicated overall satisfaction with their use of the ACSI for site evaluation. Dissatisfied teams were those that used the ACSI for a shorter period, did not receive results within the evaluation period, or were skeptical about the methodology.

At the site level, teams used the ACSI as:

- A ready-to-use customer satisfaction metric that provided pre-approved Office of Management and Budget (OMB) clearance;
- A tool for incorporating custom questions with expedited OMB approval in order to identify and qualify specific site issues and problems; and
- A source of information about audiences and web site areas to be used in planning any follow-up work involving additional evaluation methods.

The majority of teams indicated that they derived significant value from the data provided by the custom questions they added to their ACSI survey. However, many teams did not value the data provided by the ACSI model questions; they felt that these results did not relate specifically to their sites in the way that the custom questions did.

The custom questions provided highly valued information about audience segments and their level of satisfaction with the site, visit characteristics, desired site content, and site strengths and weaknesses. Teams put their ACSI data to a variety of uses, including planning site improvements, benchmarking, establishing program and work priorities, planning for additional follow-up evaluation activities, and providing feedback about site performance to their ICs and ODOs. One-half to two-thirds of teams concentrated their plans for improvements on the web site elements of Functionality, Navigation, and/or Search, elements which have tended to receive relatively lower Satisfaction Scores across many NIH sites. The majority of teams expected to use the ACSI results in their next web site redesign. Even teams that had not yet had the resources to act on their results said they were saving their custom question results for use at that time.

The following paragraphs describe the two factors that had the greatest impact on teams' perceptions of the value of the ACSI, as well as their opportunity to make full use of its capabilities.

- **Longevity.** The teams that had used the ACSI the longest tended to be most satisfied and find greatest value in its use, especially for planning site changes and comparing versions of the site before and after revisions. Teams with relatively later license term start dates and/or those that compiled too few completed ACSI surveys to receive a feedback report were disappointed because they did not have sufficient opportunity or data to act on ACSI findings. These teams indicated that they had expected to be able to obtain and use results to prepare reports and direct their web site revisions.

- **Timing of the license.** Teams that were actively involved in updating or redesigning their sites made optimal use of the custom question data and in-depth analyses to direct their plans for revising their sites and meeting audience needs. These teams tended to have resources ready to act on results and implement site changes. Timing the license to the period when team members most need information is especially critical for sites that have a long revision or redesign cycle.

Twenty-one web sites that used the ACSI data as the basis for site changes showed a mean increase per quarter in their reported Satisfaction Scores during the evaluation period. The 15 teams that did not use the ACSI data for making site changes had a slight mean decrease in Satisfaction Scores per quarter.

An important question addressed at the end of the evaluation period was whether teams would continue to use the ACSI if their ICs/ODOs had to pay the license fee. Twenty-seven of the 51 teams were not sure whether their ICs/ODOs would fund a continuation of their license after the term ended, based mainly on concerns about budget constraints across all of NIH. Many teams indicated that, in light of widespread recent budget cuts, they were not sure what resources would be available for web site work. Staff time constraints were also cited as a reason for uncertainty by approximately half of these teams. Seven teams indicated that their IC/ODO would fund a continuation, and 13 teams indicated that the value of the ACSI was not sufficient to continue using it if they had to pay the license fee. Of these 13, 10 were teams with less ACSI experience (because they had later license start dates or no experience with ACSI feedback); only 3 were teams that had used the ACSI for an extended period.

Trans-NIH Level

The ACSI provided web site teams with an expedient method to measure customer satisfaction under blanket OMB approval, which would not have been possible otherwise in most cases. It served as a vehicle for continuous qualitative data collection at what teams perceived to be a lower cost than short-term options such as a one-time user survey.

Use of the ACSI across sites provided NIH with additional benefits. The trans-NIH meetings established a network of NIH web site professionals for sharing experiences, knowledge, and tips about evaluation in general, and specifically about customer satisfaction measurement and ACSI use. The meetings brought to the surface issues that needed to be addressed across sites, such as the relatively lower Search Scores seen among NIH sites and methods to direct visitors to appropriate information sources.

Web site teams identified the following as benefits of having multiple teams using the ACSI:

- Informal mentoring within ICs and ODOs (i.e., individuals with experience using the ACSI helped others who started their ACSI process later);
- NIH web site teams became more interested in and knowledgeable about customer satisfaction measurement; and
- Teams focused on the importance of user-centered design in meeting user needs.

The following web site and context characteristics were associated with successful use of the ACSI:

- Timing the site revision or redesign stage with the license period;
- A high volume of web site traffic;
- Committed resources for site evaluation and revisions; and
- Supportive IC/ODO management and staff.

The following web site and context characteristics were associated with issues and difficulties in using the ACSI (some of these characteristics occurred in combination):

- Intranet or Internet sites were used primarily by an internal NIH audience.

- The site had a low volume of traffic (some of these were "small" sites in terms of content areas and/or number of pages).
- Manual page coding was required for implementing the ACSI survey on the site.
- Management and/or statistical staff were skeptical about the ACSI model and method.
- The site was a "niche" site for which audience expectations did not fit the site mission.

The NIH ACSI Customer Satisfaction Index consistently exceeded the e-Government ACSI Index during all quarters of the evaluation period. NIH sites continue to be significant contributors to the overall government customer satisfaction index; 42 NIH sites received scores in the first quarter of 2006. During the entire evaluation period, NIH web sites were consistently ranked among the top-performing Federal sites by ForeSee; of the 14 sites cited in this elite group during the same quarter of 2006, 11 were NIH sites. The quarterly NIH Customer Satisfaction Index has exceeded the e-Government Customer Satisfaction Index by a margin of 1.3 to 6.9 points during the evaluation period.

This evaluation showcases NIH's position in the forefront of Federal customer satisfaction evaluation. NIH's evaluation study serves as a model for a large-scale evaluation; it includes not just the systematic and simultaneous involvement of multiple web sites, but also many types of sites, in using a common customer satisfaction metric.

An additional benefit of using the ACSI methodology was that the data show that NIH sites support and contribute to the NIH missions. Results indicated that NIH sites serve a variety of audiences with diverse information needs. Visitors across NIH sites range from lay audiences (health consumers, students, etc.) to professional audiences (researchers, scientists, physicians), and many sites serve both internal NIH and external audiences. The following general findings arose from the evaluation:

- Patients and their friends and family are more likely than researchers to be satisfied with NIH sites.
- Sites on which more than 80 percent of visitors reported that they found the information they were looking for have a mean Satisfaction Score of 80 percent.

Recommendations

The following trans-NIH-level recommendations focus on ways to enhance the NIH-level benefits of ACSI use by increasing communication and sharing among teams.

- Provide additional structure to optimize support for the existing network of NIH ACSI users. During the current evaluation period, teams had the chance to interact during the periodic meetings scheduled by the LT. Additional structure could be provided through an NIH ACSI Users Group, by establishing a formal liaison with an NIH web metrics group, by adding members to the LT, or by changing the role of the LT to that of a steering committee directing a larger group of site representatives responsible for trans-NIH activities.

- Provide the means and support for more frequent and targeted contact and information sharing among teams using the ACSI. Support could be provided by distributing meeting notes to teams that miss trans-NIH meetings, providing a forum or workspace for sharing information and ideas, establishing a listserv, and/or forming web site workgroups to meet periodically to address common issues.

- Expand the use of standard questions or secondary analysis of ACSI results to evaluate web site performance across NIH.

- Conduct case study tests of new products that ForeSee offers to enhance web evaluation in concert with the Satisfaction Score results. Based on findings from the case studies, provide guidance to other teams.

- Encourage sharing of scarce resources across web sites and ICs/ODOs to address issues that are common to small groups of sites.

- To ease the restrictions on funding available for web site evaluation, consider encouraging the formation of partnerships among sites to pool limited resources. Teams with similar cross-cutting issues could jointly address those issues. These teams could review their results and responses to open-ended questions to identify cross-cutting audience issues. Alternatives for addressing the issues could be subjected to usability testing; the teams could all learn from the results and apply those general principles to make changes.

If the LT does not continue to direct collaboration activities, an alternative entity should take over that role. Options are for the ICs and ODOs to provide their own support, for a Users Group to work directly with web site teams and ForeSee, or for a designated NIH staff member to serve as a full-time director of the ACSI work across NIH.

The persistent cookie policy that NIH enacted to reduce respondent burden should be continued. Teams indicated that the policy change had a very positive effect on reducing visitor

frustration with repeated exposure to the ACSI survey invitation, especially for sites with a high percentage of internal NIH visitors.

Site-level recommendations focus on (1) ways for NIH to use ACSI experiences and patterns of success to make informed decisions about license use for additional sites and (2) ways to ensure that sites continuing their ACSI licenses set realistic goals for their next license term.

Chapter 5 of the report offers a checklist that ICs and ODOs can use to determine whether a web site has a good probability for success using the ACSI, based on site characteristics and context. The checklist addresses specific points about the adequacy of resources for implementing and using the ACSI, commitment of the IC/ODO management and staff to web site evaluation and change, and fit of web site characteristics to the ACSI methodology.

Use of the ACSI across 60 NIH web sites provided data to judge whether the ACSI serves all web sites equally well as an evaluation method. Based on the Westat evaluation, the ACSI is recommended for NIH web sites and teams that meet the following criteria:

- Teams that are committed to evaluation in general, and specifically to customer service measurement using the ACSI methodology;
- Teams that can adjust their site maintenance/revision cycle to the timing of the license;
- Teams that can commit to using the ACSI for more than a year (ideally 1½ to 2 years); and
- Sites that have high or very high traffic volume (at a level to provide 300 ACSI responses within 1 to 2 months, ideally).

Teams that do not fit these criteria should consider other methods for obtaining visitor input or should develop their own customer satisfaction surveys using some other sampling approach to yield a higher volume of data. For teams that had not had a previous opportunity to collect data about their sites directly from visitors, the custom questions allowed them to address specific site issues. However, ICs and ODOs for these sites should evaluate alternatives and determine whether the ACSI is a cost-effective way to collect these data (vs. using a contractor or internal resources to conduct a survey with similar questions).

Another site-level recommendation is to develop a guide or manual to facilitate use of the ACSI by new web sites. The manual should provide information about setting a realistic schedule and goals for using the ACSI to improve the site, making optimal use of custom questions and segmentation, obtaining assistance from a mentor, and translating ACSI results into plans and actions (an activity that was problematic for a number of teams).

For teams continuing to use ACSI licenses, Westat strongly recommends planning and scheduling site revision and redesign activities around the license period. Careful consideration should be given to timing the ACSI feedback cycle with other evaluation activities, to make optimal use of the ACSI data (e.g., for developing scenarios for user testing, for identifying areas to be addressed through heuristic review). In some cases, redesign efforts may be timed so that ACSI results can be used to compare "before" and "after" versions of the web site. Another recommendation for teams with continuing ACSI licenses is to seek out members of other site teams when in need of specific guidance (e.g., to plan and make web site changes based on ACSI data).

1. INTRODUCTION

1.1 Project Background

The Internet plays an increasingly important role for the Institutes, Centers, and Offices of the Office of the NIH Director (subsequently referred to as ICs and ODOs) of the National Institutes of Health (NIH). The ICs and ODOs make extensive use of the Internet to accomplish their missions, including disseminating information, providing access to research information, conducting business transactions related to the IC/ODO function, and supporting administrative processes for NIH programs. Many of the ICs and ODOs have multiple web sites to support their internal functions as well as their variety of individual programs.

Before 2004, web site evaluation across NIH was broadly focused on measures of web site usage (e.g., tracking reports). A minority of teams managing web sites were also using web evaluation methods such as usability testing and heuristic review. Only a few web site teams were actually measuring customer satisfaction; others relied on informal feedback channels and anecdotal reports to learn about audience reaction to their sites. Typical obstacles to broadening evaluation objectives and methods included time and resource constraints such as staff time required to develop and conduct an online survey, calendar time required for obtaining Office of Management and Budget (OMB) approval for collecting survey data, and lack of funding for web site evaluation.

In late 2003, three NIH ICs (the National Cancer Institute, the National Library of Medicine, and the National Institute of Allergy and Infectious Diseases) began using the American Customer Satisfaction Index (ACSI) to measure customer satisfaction for eight NIH sites.[1] The ACSI is an online customer survey methodology that includes both standardized and custom questions and provides the ability to benchmark the standardized question results against other government agencies and private sector companies. (For more information about the ACSI methodology, see Box 1.)

[1] These eight sites were: the National Cancer Institute main web site, the National Institute of Allergy and Infectious Diseases main web site, and the following five National Library of Medicine web sites: MedLine Plus English, MedLine Plus Español, PubMed, AIDSInfo, and TOXNET.

Box 1. Overview of the ACSI Methodology

The ACSI provides continuous real-time data on web site users' satisfaction with a web site.

The ACSI predictive model relates web site evaluation Element Scores (Content, Functionality, Look and feel, Navigation, Search, Site performance, and, in some contexts, Image) to a Composite Satisfaction Score and Future Behavior Scores (Likelihood to return and Recommend site to others). The model quantifies the potential impact that changes in each site element will have on a site's Satisfaction Score, plus the potential impact that a higher satisfaction score will have on user likelihood of returning to the site or recommending it to others. Web site teams can review these quantified impacts to help make decisions about where to allocate resources (with the aim of increasing the Satisfaction Score).

Figure 1-1 shows an example of how these scores are presented for a web site. In this case, the lowest Element Scores are Navigation (71) and Search (72), and improvements to the site Search have the potential to make the greatest impact (an increase of 2.2 points) on the Satisfaction Score.

The survey data are collected as follows. Pop-up surveys are presented to randomly selected web site visitors who view a predetermined number of pages of the site that have survey code on them. (The percentage of visitors sampled and the number of pages that must be viewed are set for each site.) In addition to the "ACSI model questions" on the survey that underlie these elements, each survey can include a number of custom questions that are tailored to collect quantitative and/or qualitative data about visitors and visit characteristics.

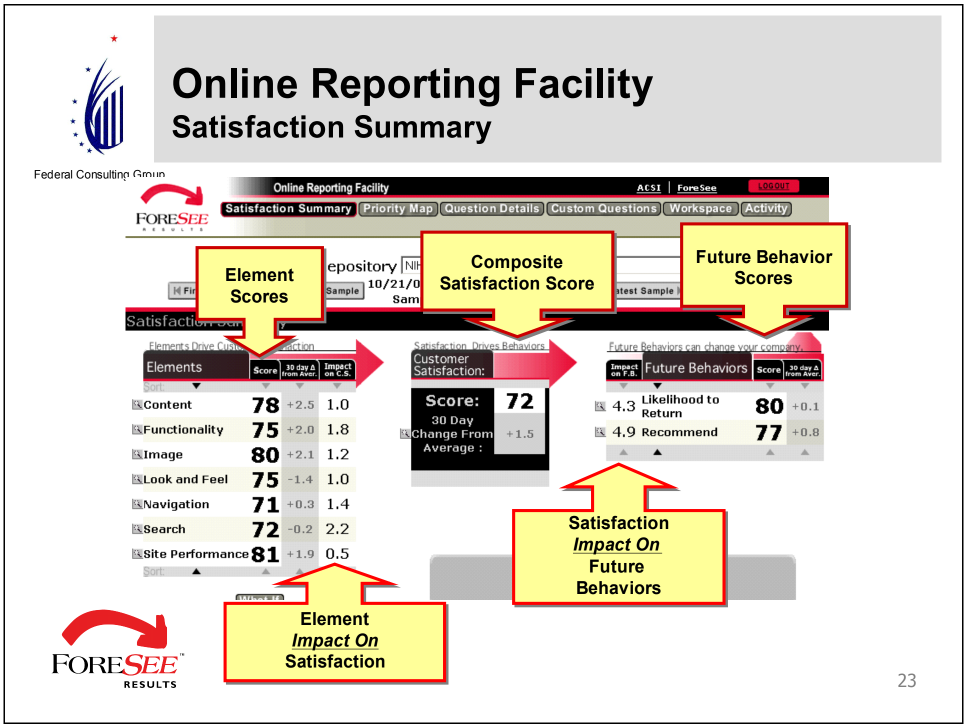

Figure 1-1. Components of the ACSI

Based on initial successes with the ACSI, a team representing NIH ICs and ODOs submitted a proposal to the NIH Office of Evaluation to extend ACSI use across NIH. ACSI licenses were offered, with the goal of encouraging sites across NIH to add customer satisfaction measurement to their current evaluation methods. For ICs and ODOs, use of a license addresses the requirement of all Federal agencies to measure citizen satisfaction with e-government. Across the government as well as in the private sector, the ACSI has become the recognized methodology for web site performance measurement.

The NIH Office of Evaluation (now the Evaluation Branch within the Office of Portfolio Analysis and Strategic Initiatives) awarded funding of $1.22 million to cover ACSI use for 60 web sites across the NIH ICs and ODOs. The award also included a component for evaluation; Westat was selected as the evaluation contractor. Table 1-1 shows the ICs and ODOs involved in the evaluation, along with the number of licenses issued to each. Eighteen ICs and 13 ODOs were involved during the evaluation period. Individual web sites that participated are listed in Appendix A.

1.2 Scope of the Evaluation

The evaluation focused on two levels of ACSI use. One level was the individual participating web site teams: how these teams applied the ACSI to evaluate their sites and the ways these sites benefited from ACSI use.

The second level of the evaluation was the trans-NIH level: the value of using the ACSI simultaneously across NIH sites. A team of seven NIH co-principal investigators, the NIH Leadership Team (LT), directed this trans-NIH effort. Members represented the Office of the Director (OD) as well as three ICs; team members are Dr. Siegel, Dr. Wood, and Ms. Love of the Library of Medicine; Mr. Rodrigues of the NIH Office of Communications and Public Liaison; Ms. Feldman of the National Cancer Institute; Mr. Malamud of the National Heart, Lung, and Blood Institute; and Ms. Lagana of the Center for Information Technology. At this trans-NIH level, the LT fulfilled the following roles:

- Administering the ACSI licenses for participating sites through liaison activities with the Federal Consulting Group and ForeSee Results, the contractor that administers the ACSI methodology for e-government applications;

Table 1-1. Institutes, centers, and offices within the office of the NIH Director participating in the ACSI Trans-NIH Evaluation, with number of licenses issued to each

Institutes and Centers		Offices Within the NIH Office of the Director (OD)	
**Name**	**No. of licenses**	**Name**	**No. of licenses**
National Cancer Institute	7	OD/Office of Animal Care and Use	1
National Eye Institute	1	OD/Office of Communications and Public Liaison	2
National Human Genome Research Institute	1		
National Heart, Lung, and Blood Institute*	(6, 5)	OD/Office of Extramural Research	2
National Institute of Allergy and Infectious Diseases	1	OD/Office of Electronic Research and Reports Management	1
National Institute of Arthritis and Musculoskeletal and Skin Diseases	1	OD/Office of Human Resources	1
		OD/Office of Research Services	1
National Institute on Drug Abuse	2	OD/Office of Research Facilities	1
National Institute on Deafness and Other Communication Disorders	3	OD/Office of Rare Diseases	2
		OD/Office of Intramural Research Continuing Medical Education	1
National Institute of Dental and Craniofacial Research	1		
		OD/Office of Dietary Supplements	1
National Institute of General Medical Sciences	1	OD/Office of Technology Transfer	1
		OD/Office of Science Policy/Office of Science Education	2
National Institute of Mental Health	1		
National Library of Medicine	7	OD/Office of Science Policy and Planning*	1
Center for Information Technology*	(7, 3)		
National Center for Complementary and Alternative Medicine	1		
Fogarty International Center	1		
National Institute on Aging	1		
National Institute of Diabetes and Digestive and Kidney Diseases	1		
National Institute of Environmental Health Sciences	1		
Totals (Initial number allocated, actual number used)	(44, 39)		17

Note: Appendix A lists the individual sites participating in the Trans-NIH evaluation.

*These ICs reallocated licenses to other sites or absorbed months into existing active licenses.

- Planning and conducting periodic meetings of the participating web site teams; and
- Providing oversight and review of the evaluation activities performed by the evaluation contractor (Westat).

The LT scheduled and invited all participating teams to periodic trans-NIH meetings. (The timing of these meetings typically coincided with the meeting that ForeSee Results holds to present quarterly e-government ACSI results to a government-wide audience.) At the trans-NIH meetings, ForeSee staff reported the progress of implementation across participating web sites, provided an overview of NIH web sites' performance relative to other government and private industry sites, and reported sites' scores. To address expected needs across the participating teams, the LT also allocated meeting time to discussion of issues relevant to ACSI use. Examples are issues related to search, use of persistent cookies to reduce visitor burden, and use of custom questions and segmentation. These NIH-wide topics were addressed through web site case studies and question and answer periods during the meetings.

The main objective of the evaluation at this second level was to assess whether broad ACSI use across individual web sites provided additional benefits for NIH. The basic question was whether the ACSI, widely used for web site evaluation by private sector companies and other government agencies, would be useful across NIH. A related objective was to determine whether the ACSI site-level data provided any additional insight into how NIH web sites are used and how they perform relative to NIH communication goals.

As described above, the evaluation project was limited to two distinct levels. The evaluation did not cover the methodology that the teams used for site evaluation. Evaluating the ACSI itself, including its structure, analytic model, and comparison with other methods of measuring customer satisfaction, was not within the scope of the evaluation.

1.2.1 Time Frame for the Evaluation

The project was originally funded for an 18-month period, based on a schedule that called for implementing the ACSI across participating web sites in a phased approach. The rationale for staggering the start dates over a 4- to 6-month period was to provide web site teams with ample time to complete start-up steps and to ensure an even workload for Foresee and its Satisfaction Research Analysts (SRAs), who were responsible for preparing the sites for implementation.

The expected reporting and analysis cycle for the NIH web sites was based on the ForeSee implementation and reporting cycle shown in Figure 1-2. A measurement and revision cycle starts with implementation steps (e.g., a planning meeting to set ACSI survey parameters, development and testing of survey code). After a survey goes live, a site moves to the reporting cycle, which includes components of collecting data and monitoring responses, reviewing results informally online and in more formal reports with the ForeSee SRA, and participating in quarterly review meetings. Web site teams may or may not take subsequent steps, based on the results and any constraints of the situation (e.g., staff time to review and act on results, funding for web site revision activities). Teams may initiate activities to plan, redesign, and/or re-launch a site and then reassess whether these activities have resulted in any changes in ACSI scores.

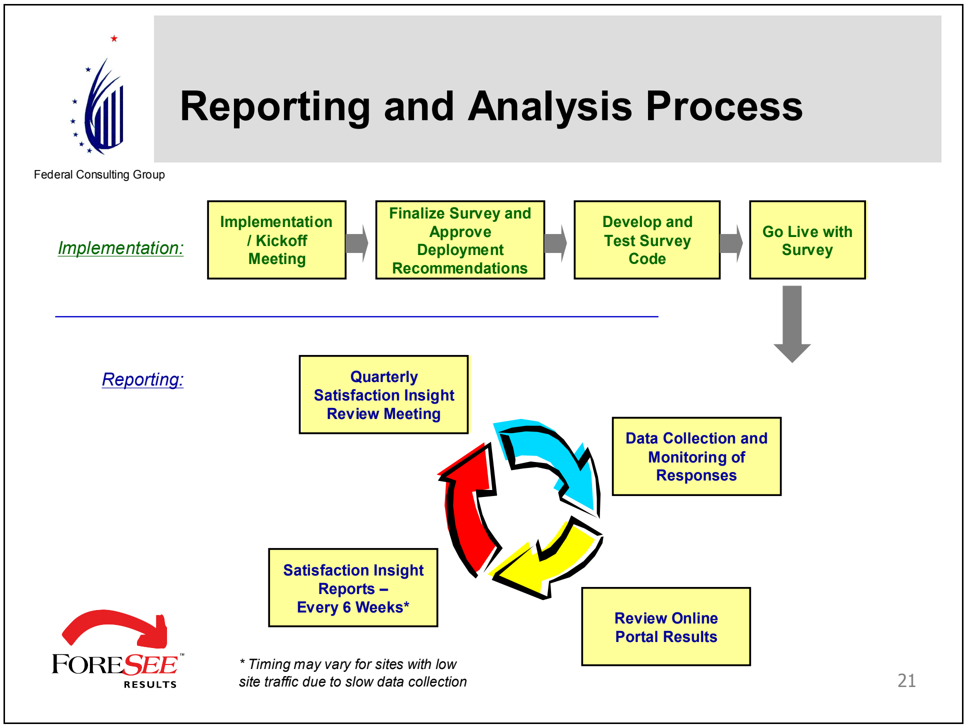

Figure 1-2. ACSI reporting and analysis process

At the start of the project, there was a general expectation that the 18-month time frame would provide sufficient time for teams to begin and complete an improvement cycle. However, this did not prove to be the case, as many sites implemented the ACSI later than planned. Delays were related to the development stage of the web site, teams choosing to address site issues before implementing the ACSI, teams timing the license to seasonal site use patterns, and other factors.

The LT received supplemental funding of $250,000 from the Office of Evaluation in July 2005. The funding extended the licenses and evaluation for 6 months. For the majority of web sites, the licenses were extended through June 2006. For 18 sites that started their licenses later, the term end dates were extended 1 to 3 months. This extension allowed more sites to collect data simultaneously.

1.2.2 Participating Web Sites

The following are general characteristics of the participating web sites:

- General IC home pages and/or portals supporting programs for public access to NIH;
- Sites supporting access to and use of research data for disseminating research results;
- Sites for disseminating general medical information for programs meeting congressional requirements to provide state-of-the-art information on health issues and their treatment;
- Sites for transacting extramural business such as grant applications or promoting access to clinical trials to support research programs; and
- Sites for intramural administrative processes or that provide the infrastructure for NIH programs.

At the project start, 60 ACSI licenses were allocated among the 18 ICs and 13 ODOs that indicated interest. Several of the ICs/ODOs had sites that were originally expected to use a license but elected not to do so. In such cases, the licenses were either transferred to another site or the unused months were added on to the licenses of other active sites. Licenses for 55 sites were still active in early 2006. Subsequently, 2 sites stopped collecting ACSI data before the end of the evaluation period. Appendix A provides the reasons why sites changed or transferred licenses. By the first quarter (Q1) of 2006, 42 sites were generating ACSI score data and contributing to the NIH Customer Satisfaction Index.

1.3 Scope of the Final Report

This evaluation report covers project activities between October 2004 and May 2006. The report integrates and presents background, process, and outcome data for all participating web sites, with

an emphasis on outcome data. Due to the general delay in start times for many sites, there are some limitations on the outcome data that could be collected and reported. Most sites did not progress as far through an improvement cycle as expected within the extended time frame of the evaluation. In addition, some sites that received feedback and identified improvements did not have sufficient time to implement site changes, due to funding cycles, budget considerations, and other factors.

By the end of the evaluation period (May 2006), 12 sites had not collected their first sample of 300 completed ACSI surveys. Therefore, these sites did not receive a results report based on the full ACSI model (Element Scores, Composite Satisfaction Score, and Future Behavior Scores). ForeSee provided approximately half of these sites with a preliminary report of their custom question results, so these sites received at least limited customer feedback.

2. EVALUATION METHODS

The evaluation required a multi-method approach to capture the variety of data types for the two levels of the evaluation and the characteristics of participating web sites and teams. The main types of data collected are described below:

- **Background data:** characteristics about the participating sites and teams before use of the ACSI.
- **Process data:** descriptions of how the ACSI was implemented across the sites, and the teams' experiences with implementing and using the ACSI.
- **Intermediate outcome data:** the teams' perceived benefits of ACSI use, perceived value of ACSI results, satisfaction with the ACSI for site evaluation, and use of ACSI data.
- **Longer-term outcome data:** impacts of site revisions, impacts of sites using ACSI, teams' desire to continue ACSI use, value added by involvement in the collaboration, contributions to NIH missions, trans-NIH benefits of the collaboration, and Federal-level impacts.

2.1 Data Collection Methods and Sources

The methods and data sources are listed in Table 2-1, with a brief description of the primary content that each source provided. The plan developed to guide the evaluation called for each data source to cover all or a subset of the sites and meetings. The last column of Table 2-1 indicates the planned coverage for the source and the number of sites or meetings for which data were actually collected.

The strategies used to collect the data and the timing of the data collection activities are described in the following sections. Copies of the email survey instruments and the interview protocols are included in Appendix B.

Table 2-1. Methods used to evaluate the use of the ACSI at NIH

Method/Data Source	Primary Content	Planned Coverage (Actual n)
**Review of secondary data**		
Web site review	Coding of a variety of website characteristics	All sites (61)
ForeSee pre-implementation worksheets	Coding of team's responses to pre-implementation questions	All sites (48)
ACSI data for sites generating sufficient response for model data	Satisfaction results per quarter	All sites collecting data during evaluation period: Q4 2004 (8) through Q1 2006 (42)
ACSI site-level data aggregated to NIH level	Standard custom question results<br>Secondary analysis results	All sites using standard custom question; all sites using similar questions (varied by type of analysis)
**Surveys**		
Initial survey	Site background<br>Site evaluation before ACSI<br>Reasons for joining the trans-NIH ACSI evaluation	All sites (57)
Final survey	Intermediate outcomes<br>Longer term outcomes<br>Trans-NIH benefits	All sites (51)
**Interviews**		
Initial in-depth interview (Primary focus: processes)	Implementation process<br>Receipt and use of ACSI results<br>Trans-NIH benefits	Subset of sites (14 in 2005; 6 in 2006)
Final in-depth interview (Primary focus: outcomes)	Intermediate outcomes<br>Longer term outcomes	Subset of sites (20)
Final brief interview	Benefits of ACSI use without full activities and data for full model	Subset of sites with less ACSI experience (5)
**Observations**		**Coverage (Number of meetings)**
Observation of implementation and feedback meetings	How teams:<br>Implemented ACSI<br>Received and reacted to feedback	Sample of meeting types – implementation, initial feedback, follow-up feedback (15 meetings)
Observation of trans-NIH meetings	Attendee questions and issues<br>Discussion topics<br>Case studies	All trans-NIH meetings (5 meetings)
Observation of Leadership Team meetings	Management of trans-NIH effort<br>Perceptions about ACSI use across sites	Bi-weekly meetings (all meetings during evaluation period)

2.1.1 Review of Secondary Data

The review of web sites and pre-implementation worksheets provided data for use in categorizing the sites. These characteristics were examined in order to identify characteristics that related to patterns of success in using the ACSI.

These sources were reviewed between January and March 2005; late starting sites were reviewed in April 2006. The review is described below:

- **Web sites.** Contractor staff reviewed the sites and reached consensus on coding of specific characteristics including site structure, types of transactions supported, intended audiences, presence of a web site mission statement, and whether the web site URL was part of a larger web site.

- **Pre-implementation worksheets.** Most web site teams (some in conjunction with the ForeSee SRA) completed these forms as an initial planning step before implementing the ACSI survey. The form captured web site teams' expectations for using the ACSI, the business objectives of the site, and evaluation practices prior to using the ACSI.

The ACSI provided quantitative data on a quarterly basis to assess the level of customer satisfaction for individual sites. ForeSee also analyzed the quantitative data to investigate questions about the performance of sites across NIH and their role in meeting NIH missions.

These data were obtained from ForeSee near the end of the evaluation period in May 2006:

- **ACSI score data.** ForeSee provided Composite Satisfaction Scores for all sites that received these scores for Q4 2004 through Q1 2006.

- **Secondary analysis results.** ForeSee analyzed results for one standard custom question that addressed how visitors planned to use information they found on an NIH site. ForeSee aggregated data across sites for four additional custom questions that were similar across sites (visit frequency, visitor role, whether visitors found the information they looked for, and reason for visiting the NIH site).

2.1.2 Surveys

All survey data were collected via email invitation and reminders. The initial survey focused on web site background and site evaluation practices prior to using the ACSI. The initial survey data were used to create a baseline of site and team characteristics. The final survey focused on outcomes, and

captured a variety of measures used to quantify how site teams used the ACSI, the benefits of using the ACSI, team members' judgments about its value, and the benefits of participation in the trans-NIH meetings.

The initial survey was administered to all teams during August 2005. The final survey was administered to a split sample because some sites had later license start dates and some did not progress into improvement cycles as expected. The first set of 32 surveys was sent in March 2006 to teams that had received a feedback report and/or had an initial results review meeting with ForeSee by Q4 2005. These teams were judged to have had enough time using the ACSI to provide judgments about their experiences. To close out the data collection and qualify the outcomes for teams with less ACSI experience, the remaining 23 sites with licenses received a survey in early May 2006. The survey was modified slightly to provide teams with response options to indicate the ACSI activities they had not experienced.

2.1.3 Interviews

The objectives for the in-depth interviews were to collect additional qualitative information about teams' ACSI experiences and to add further explanation to the survey results.

Near the end of the evaluation period, when it became clear that some of the teams would not accumulate enough ACSI responses to receive an initial feedback report and meeting, the LT decided to conduct a very brief interview with a sample of these teams. The objectives for these interviews were to learn what factors accounted for sites' slow collection rates and/or delay in start time, as well as to collect qualitative information about their limited experiences with the ACSI.

The 14 teams with the most experience using the ACSI were interviewed during the summer of 2005 and again in March 2006.

Six additional teams were selected in April 2006 to add breadth to the group of sites already included for in-depth evaluation. These teams were interviewed once, and they responded to questions on both the initial and final interview protocols. The teams were chosen to ensure diversity in the following areas:

- A range of implementation dates (from January to August 2005), so that teams had different periods of use and experiences to relate;

- ICs beyond those represented by the 14 sites previously interviewed;
- A variety of responses to process and outcome items on the final survey;
- A range of levels of site traffic volume; and
- A range of site characteristics.

In May 2006, the LT members realized that a group of sites would not obtain the required number of surveys (approximately 300) to receive their first sample with satisfaction scores and other ACSI analyses. In order to capture information about this group regarding their experience with the ACSI, Westat contacted five teams to participate in a final short interview in late May 2006. These teams were selected from the respondents to the last survey. These teams were also selected to represent the range of starting dates, various ICs/ODOs, and a variety of responses on the final survey.

The interviews covered the following topics and were administered to the following numbers of teams:

- **Initial in-depth interview (n = 20 teams).** The protocol focused on teams' views about the ACSI implementation, the data review processes, use of the feedback, and benefits of involvement in the trans-NIH project.
- **Final in-depth interview (n = 20 teams).** The protocol assessed teams' understanding of and satisfaction with the ACSI results, custom questions, and segmentation; impact on evaluation practices; and lessons learned from the trans-NIH project.
- **Final short interview (n = 5 teams).** The protocol included specific questions about why sites did not progress at a rate that allowed them to take full advantage of the ACSI methodology and what benefits they were able to achieve from its limited use.

2.1.4 Observations of Program Processes

Westat staff listened to conference calls and attended meetings over the course of the evaluation period to track use of the ACSI at the team level and trans-NIH activities. The following three types of meetings were observed:

- **ForeSee and team meetings.** The ForeSee SRAs scheduled and conducted these meetings with teams to plan for implementation, provide instruction in how to use the online reporting facility, and present and discuss quarterly results.

- **Trans-NIH meetings.** The LT scheduled and invited all participating teams to attend quarterly meetings. These provided updates on progress in implementing the ACSI across sites, feedback about ACSI scores for NIH web sites, and sharing of information about ACSI use across sites.

- **Bi-weekly LT meetings.** Throughout the evaluation period, the LT held these meetings to direct the trans-NIH project and evaluation. ForeSee and Westat participated in these conference calls.

3. SITE-LEVEL RESULTS

This chapter addresses results at the level of the participating websites; Chapter 4 presents trans-NIH-level results. An overview of the results presented in this chapter is provided in Box 2. Results in this chapter are organized into the following sections:

- How web sites have used the ACSI;
- Intermediate outcomes associated with ACSI use;
- Longer term outcomes of ACSI use; and
- Web site characteristics associated with issues in using the ACSI.

The last section discusses contexts for which there are some cautions for ACSI use. Several ways of categorizing the many sites are also presented; these categories will also serve as the basis for identifying which NIH sites are likely to derive the most benefits from ACSI use in the future, to make more informed decisions about allocation and use of ACSI licenses at NIH.

The research questions in the first section are addressed with the in-depth interview data (n = 20). Results for most of the remaining research questions are based on data collected through the final survey (n =51). The data source is identified for each result and noted in each of the figures.

3.1 How NIH Web Sites Have Used the ACSI

One of the critical objectives of the initial interview questions was to identify factors that account for "successful" use of the ACSI. Success was defined loosely as positive perceptions on the part of the participating web site teams. The next sections summarize teams' success with key activities associated with ACSI start-up, data interpretation, and action planning.

An improvement cycle includes conducting activities required to add the ACSI survey to a site, interpreting results, refining custom questions and segmenting data, using results to plan actions, executing changes, and monitoring subsequent round(s) of results. Teams' perceptions of the ease with which they completed these activities influenced their overall level of satisfaction with the ACSI.

Box 2. Overview of Key Results in Chapter 3

1. The majority of web site teams were able to implement the ACSI and receive results for their sites. Issues surfaced in cases where:
   - Adding code to web site pages was a labor-intensive process;
   - Internal IC/ODO staff or management were skeptical about the ACSI methodology; or
   - ACSI data accumulated slowly (e.g., for intranet sites or sites with low traffic volume).
2. Across all sites, teams derived the most value from their custom question and segmentation data rather than from their ACSI model data.
   - Data provided valuable insight about audience profiles and visit characteristics.
   - Teams took advantage of having a continuous feedback source for identifying site problems and audience information needs.
   - Teams used their custom question data to plan a variety of types of site improvements to address areas identified as important for improving customer satisfaction.
3. Timing of the license was a key factor in perceived value of the ACSI.
   - Teams that were actively involved in updating or redesigning their sites used the custom questions and segmentation analyses to address needs. These teams tended to have their resources ready to act on results and implement site changes.
   - Teams that did not currently have the staff time to devote to reviewing results indicated that they were saving their qualitative data for use in planning their next redesign.
4. Longevity was a key factor in making optimal use of the ACSI for web site evaluation.
   - Teams that used the ACSI the longest tended to be satisfied with and find value in its use, especially for planning site changes and comparing versions of the site before and after revisions.
   - Teams for sites with relatively later license term start dates and/or slow rates of collecting ACSI surveys tended to be dissatisfied with the ACSI because they did not have sufficient time or opportunity to receive and/or act on ACSI results.
5. Web site teams expressed some dissatisfaction with the process of using the ACSI in cases where:
   - There was turnover of the Satisfaction Research Analysts (SRAs) assigned from ForeSee.
   - Teams perceived that ACSI Satisfaction Scores did not truly reflect site quality (e.g., sites for which visitors look for content that does not fit within the site mission).
   - Staff time constraints were a barrier to attending to or acting on the perceived large volume of ACSI data. Some of these teams would prefer to use an online survey on a more intermittent basis.
6. Web site teams that used the ACSI data as the basis for site changes (21 sites) or as part of their continuous improvement cycle (15 sites) showed a mean score increase per quarter in their reported Satisfaction Score. The group of sites that did not use the ACSI data in these ways had a slight mean score decrease per quarter.
7. Approximately half of the web site teams (27) were not sure whether their ICs/ODOs would pay to continue their license after the term end, based on concerns about budget constraints across all of NIH. Seven teams indicated that their IC/ODO would fund a continuation, and 13 teams indicated that the value of the ACSI was not sufficient to continue using it if they had to pay the license fee.

3.1.1 Perceived Effectiveness of ACSI Start-up Activities

Representatives of the site teams were asked to describe their start-up activities of setting parameters (loyalty factor, sampling rate), developing custom questions, and adding code to the identified pages/sections of the site. Coding of the qualitative responses captured the positive or negative nature of these perceptions.

Results shown in Figure 3-1 indicate that teams were most positive about developing their custom questions. Eighteen of 20 teams indicated that this activity proceeded smoothly, without problems. Most teams indicated that their SRAs provided on-target guidance for developing their questions. In some cases, teams were able to build upon survey questions they used for their previous in-house surveys and/or they received assistance from other NIH staff who had experience with the ACSI. In addition, ForeSee provided the LT and participating site teams with a list of custom questions developed by other teams across the Federal government. Teams found the list to be extremely useful in developing their own custom questions.

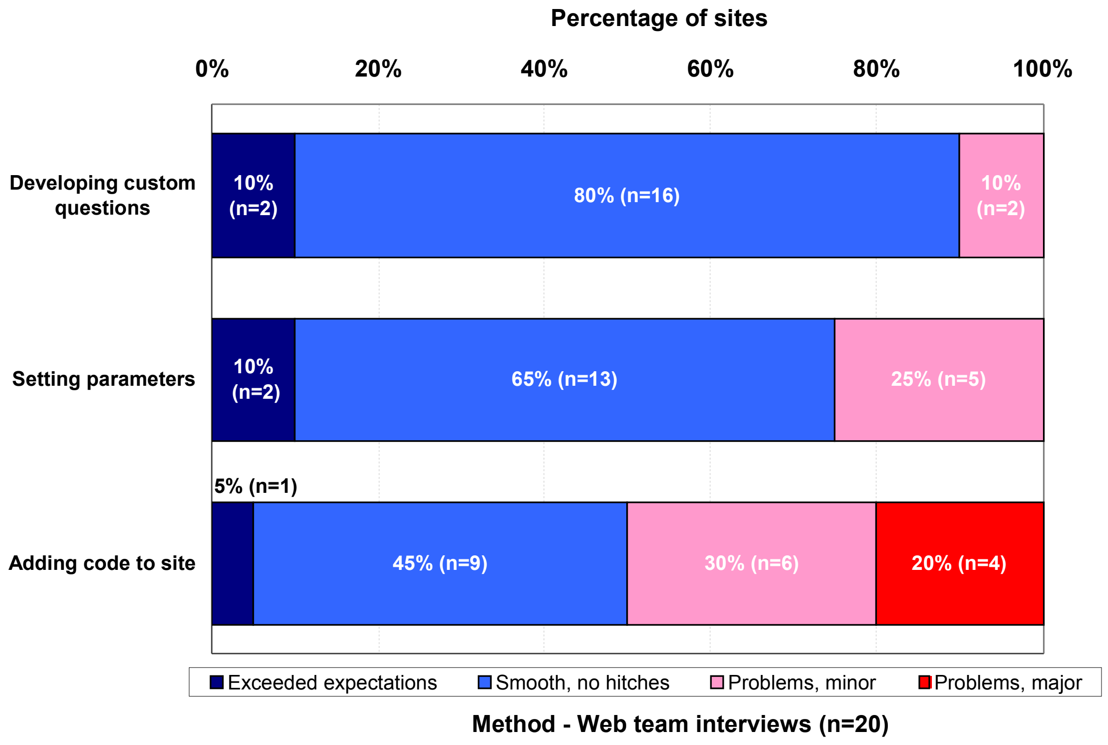

Figure 3-1. Web site team rating of key start-up activities

The start-up activity that was problematic for 10 of the 20 teams was adding the required ACSI code to the site. The teams that had the most problems were ones that had to add code to individual pages, rather than through more systematic global changes, such as a content management tool. Five teams had minor difficulties setting parameters. These sites initially set a sampling rate or loyalty factor that the team thought was too low (e.g., asking visitors to respond to the ACSI survey when they had seen relatively few pages); this required making a series of adjustments.

3.1.2 Perceived Value of ACSI Feedback

Once web sites met the threshold of 300 completed ACSI surveys (required to obtain the first "sample" of data and for access to the online reporting facility), their SRAs generated initial feedback reports. Typically, SRAs scheduled a conference call with the site teams to jointly review the initial results, plan how to tailor the custom questions (if appropriate for the context), and specify audience segments of interest for further in-depth tracking and analysis. In some cases, the initial meeting was conducted more informally and/or delayed to suit the team's purposes.

Teams were asked whether their ACSI scores matched their expectations. The results shown in Figure 3-2 indicate that scores were higher than expected for 8 of 20 teams. Scores were lower than expected for just two teams; these teams had made site changes before using the ACSI and were surprised that the initial scores were not higher. The remaining 10 teams were split between having no initial expectations about their scores and achieving scores within their expected range.

Web site teams were asked to describe what insights the ACSI feedback provided about their sites and how useful the data proved to be. Figure 3-3 shows the results of coding those comments on each of four dimensions (produced new findings, confirmed other findings, produced counterintuitive results and produced uninterpretable results). For the majority of the 20 sites, the results revealed new information about characteristics of visits and visitors and/or confirmed what teams knew about their web sites from separate evaluation sources such as their previous in-house surveys and web site traffic reports.

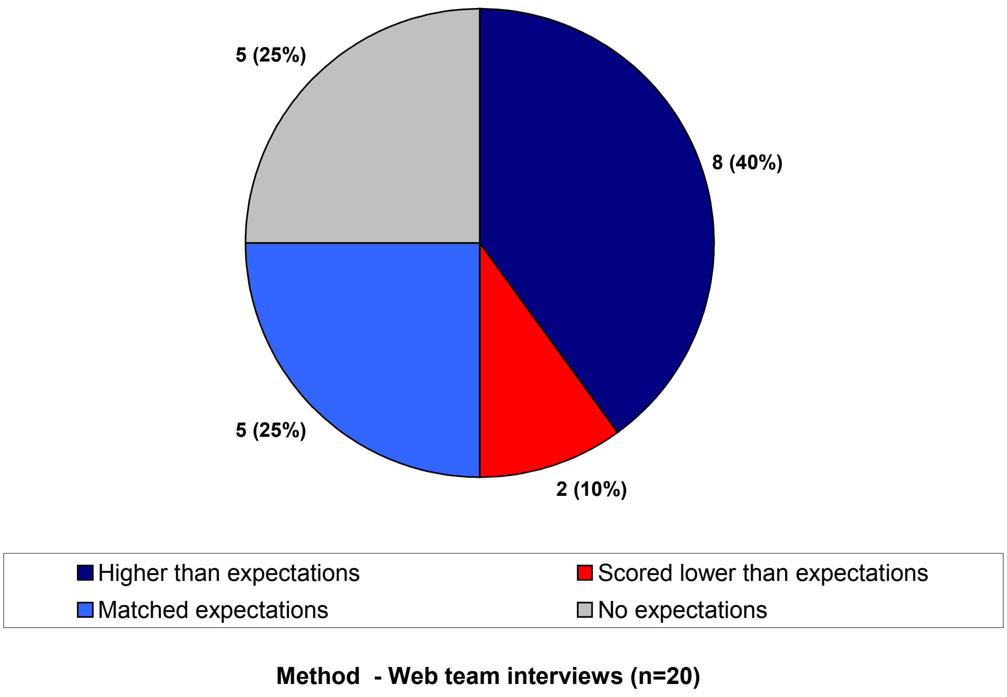

Figure 3-2. Match between ACSI scores and web site team expectations

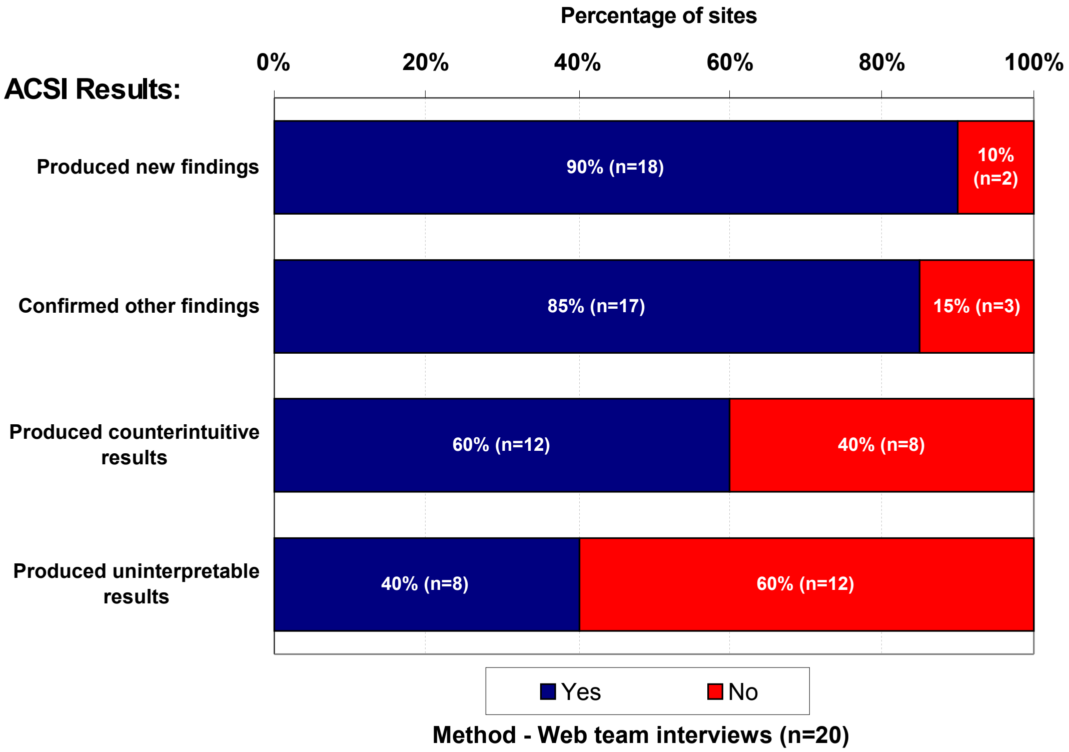

Figure 3-3. Web site team comments on value of ACSI findings

Web site teams also reported some difficulty understanding results. Examples of results teams considered to be counterintuitive were low Search Scores after making the switch to a Google-type search, a low Satisfaction score paired with very high Future Behavior Scores (Likelihood to Return and Primary Resource), and a low primary resource score for the principal target audience. As an example of what teams considered to be uninterpretable results, several teams described open-ended question responses that did not clearly indicate the section of the web site to which the comments applied. Teams could not directly take action on these comments, but in some cases they were able to adjust the wording of their custom questions to collect more specific information in subsequent samples. Two of the three teams that reported both no new findings and no confirmation of other findings were also not interested in continuing to use an ACSI license (addressed below in the section on longer term outcomes).

3.1.3 Perceived Value of Review Meetings and Satisfaction Research Analysts

Another focus of the in-depth interviews was the web site team's perceptions of the value of the ForeSee feedback meetings. Teams were asked to describe the benefits of the meetings (e.g., whether the meetings addressed their issues, prepared them for next steps to take with custom questions and segmentation) and working with the SRAs. Results related to satisfaction with the meetings and the SRAs are given in Figure 3-4. The majority of teams were positive about the initial meetings, indicating that these early meetings were very useful in learning about the data.

The findings show that half of the teams were positive about their working relationships with SRAs and half were negative. Extremes were shown by two teams that reported that their SRAs were very responsive and helpful and by two teams that were very negative about experiences with their SRAs. Half of the teams mentioned minor or major issues with SRA performance: SRA turnover and inconsistent levels of performance between SRAs after turnover. Teams mentioned the time required for a replacement SRA to become familiar enough with their site context to provide guidance and direction. In some cases, the teams felt better served by an original SRA; in others, a more experienced replacement performed better than an original SRA.

The ForeSee support model allows for SRAs to conduct quarterly meetings with the teams after the initial feedback meeting. This pattern varied considerably across sites. The general pattern was that meeting frequency decreased over time. In many cases, this occurred because teams became more comfortable with interpreting their ACSI data. In addition, meetings were held less frequently in cases

where results changed little from quarter to quarter, the teams relied on the online reporting facility to obtain results more informally, and/or there was SRA turnover.

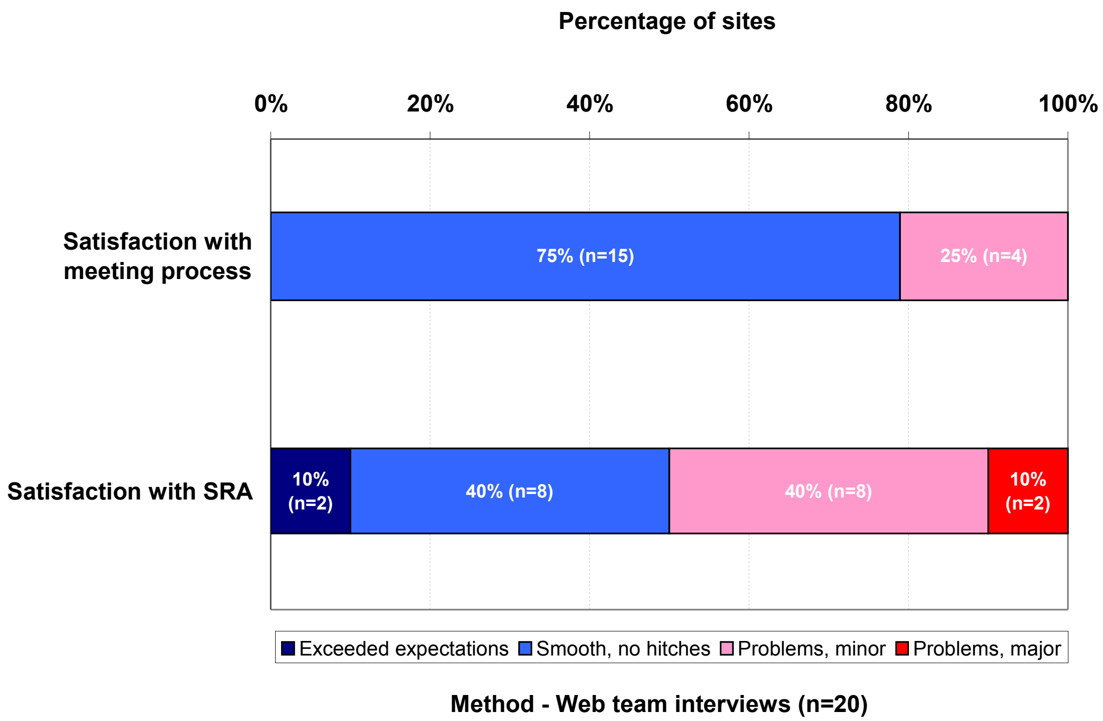

Figure 3-4. Site team satisfaction with feedback meetings and SRAs

The types of insights that the ACSI results provided about the sites are summarized in Figure 3-5. Across interviewed teams, the main benefit of the ACSI data was identifying search problems. Some teams analyzed the terms visitors used when searching. Many teams mentioned successful wording of open-ended custom questions to elicit specific information related to both (1) existing site content that visitors look for but cannot find (indicating navigation problems) and (2) nonexistent content that visitors try to find. The latter helped teams to address whether to add content to their sites or post a statement on the web site that the content is not available on the site. In some cases, teams responded by providing links to the desired content on other sites.

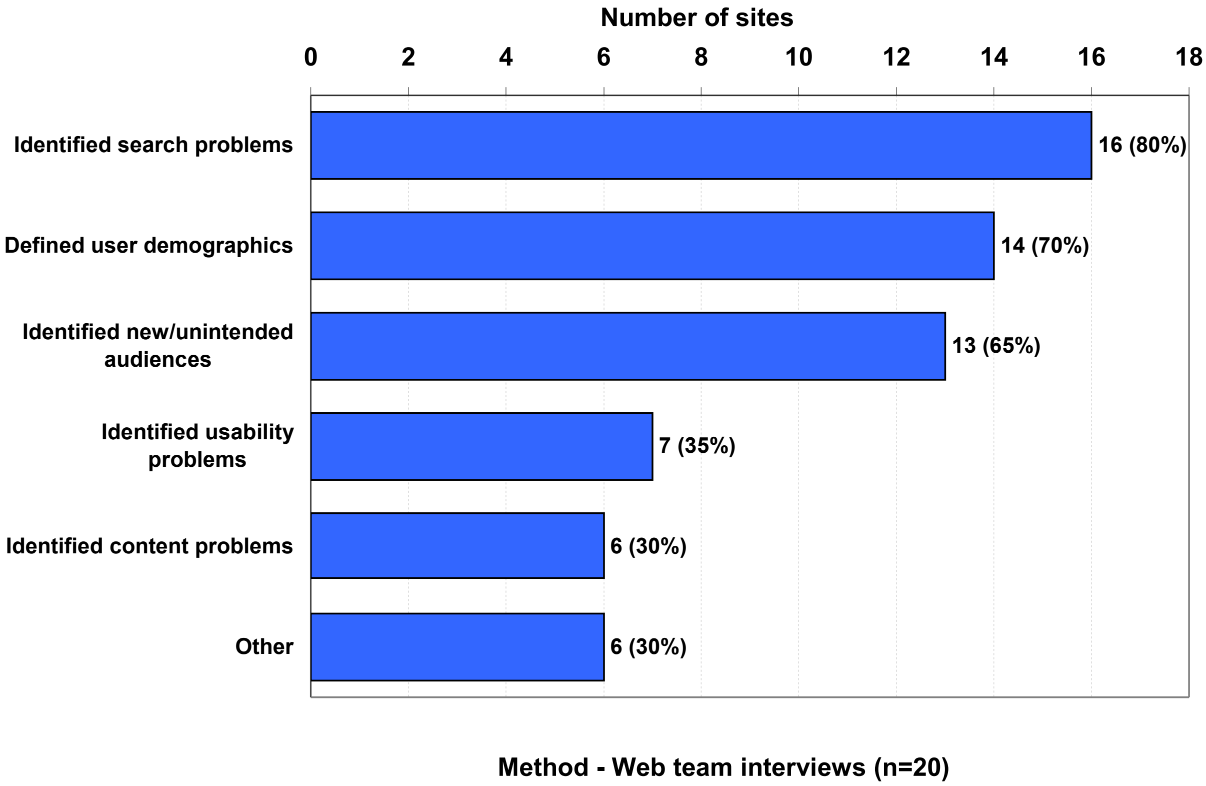

Figure 3-5. Web site teams' perceived benefits of ACSI score data

Additional benefits for 14 and 13 sites, respectively, were defining demographics of audience groups and learning characteristics of unintended audiences. Teams mentioned that their expectations about their intended target audiences were typically confirmed, but that, in some cases, the findings pointed out misperceptions about these important audiences (e.g., their level of satisfaction, site sections these audiences were not using as expected). Other teams mentioned using the open-ended data to generate ideas for the next redesign, identify errors to be corrected on their site, and identify underused features.

3.1.4 Understanding Segmentation, ACSI Results, and Use of Results for Planning

The final survey included items to assess web site teams' overall understanding of how to interpret ACSI findings, conduct more in-depth analysis of ACSI data, and use the data as the basis for planning site improvements. Ratings in Figure 3-6 show that more than 60 percent of the teams

understood (based on "Strongly agree" and "Agree" responses) how to use segmentation, interpret results, and use results to plan site revisions (34, 37, and 32 of the 51 teams, respectively). Responses by two teams indicated that they experienced some difficulty understanding how to use segmentation. Interview comments indicated that learning how to take full advantage of segmentation required some investment of time and effort. In several cases, very experienced SRAs helped to guide teams to identify appropriate segments for further analysis.

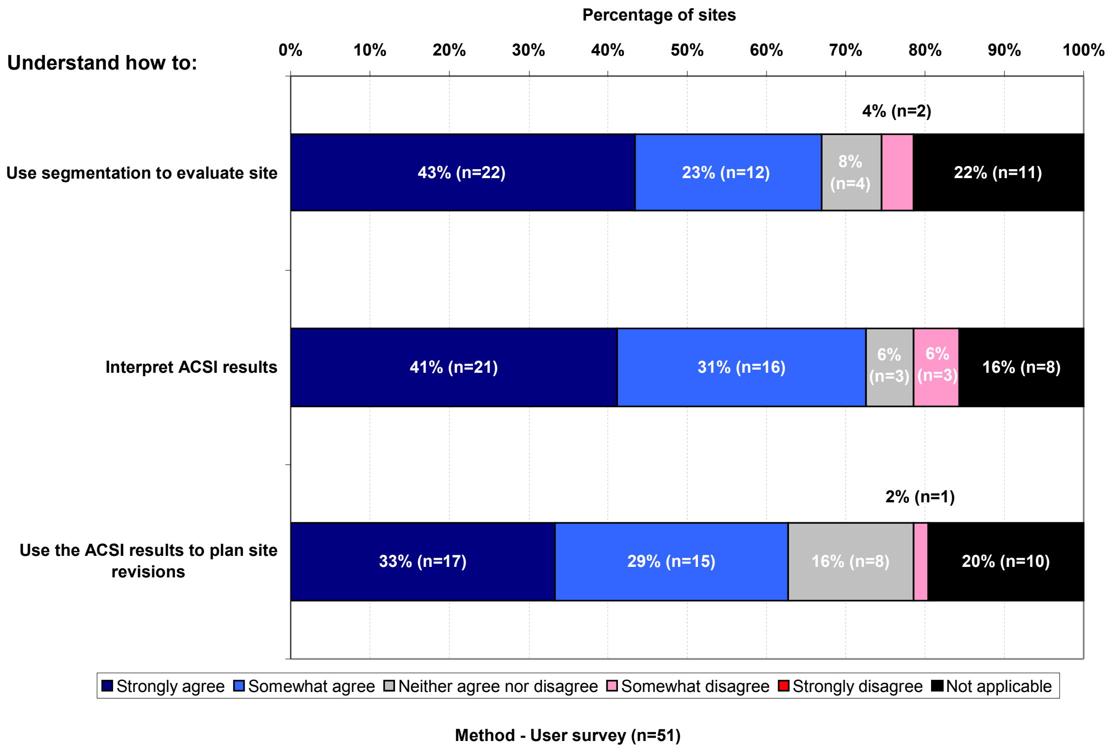

Figure 3-6. Web site teams' understanding of ACSI results, segmentation, and planning

3.1.5 Level of Web Site Teams' Experience With the ACSI

An item on the final survey was included to measure web site teams' level of experience with using the ACSI, apart from the actual time that their licenses were active. Teams were asked to indicate which data review and interpretation, planning, and change activities they had completed from a list of activities. Activities ranged from those typically done after reaching the target of 300 completed surveys (such as participating in an initial review meeting and modifying custom questions to investigate initial issues that surface) to those done by teams that fully integrated the ACSI tool into their web site

management practices (e.g., used ACSI feedback in a continuous improvement cycle). Teams' reported activities are presented in Figure 3-7. Teams were grouped to allow further examination of the relationship between activities and the approximate length of time teams have actively used their license. The three groups are: 14 teams that used the ACSI prior to the start of the trans-NIH project or implemented in the first group during December 2004, 18 teams that implemented between January and August of 2005, and the remaining 19 teams that started licenses relatively late or collected data slowly.

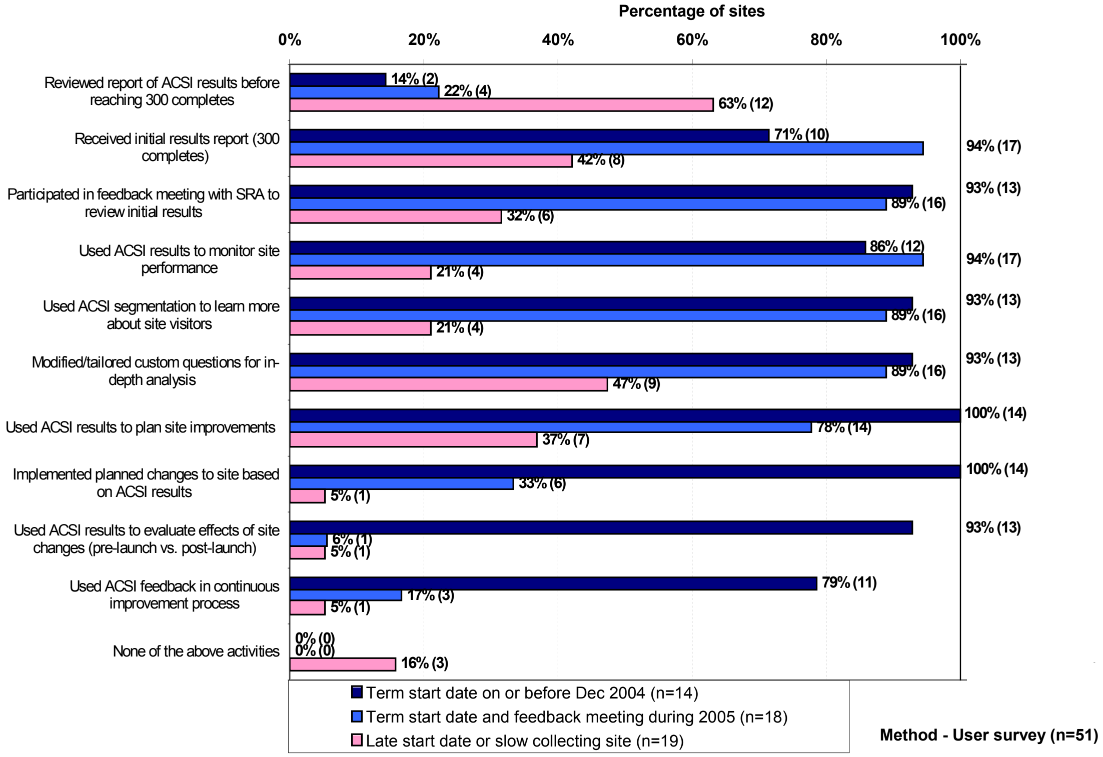

Figure 3-7. Reported activities broken out by license term start date

The expectation was that all 32 teams in the first two groups had received an initial results report and had a feedback meeting. Four of the five teams that indicated they did not receive an initial results report were among the eight teams that used the ACSI prior to the start of the trans-NIH effort. These teams did not follow the same pattern of activities during their initial steps. One of the three teams that did not participate in a feedback meeting had used the ACSI solely for making plans for changes and then making some changes (based on qualitative feedback via custom questions); the low level of involvement was attributed to management's skepticism of the ACSI model and response rates.

An examination of Figure 3-7 shows two main patterns or levels of ACSI use. One pattern is represented by most sites (approximately 75% to 100%) in the first two groups. These teams had reviewed their data, used the custom questions and segmentation analyses to deepen their understanding of their data, then used these data as the basis for monitoring site performance and planning site revisions.

The second pattern of activities is represented by teams that had, in addition to the activities described above, incorporated the ACSI more fully into their web site evaluation practices. These teams had actually based changes on results, compared subsequent results to evaluate the impact of those changes, and used ACSI results as part of a continuous improvement effort. While fewer than 33 percent (for each of these three activities) of the 18 sites that implemented the ACSI during 2005 had conducted these activities, more than 75 percent of the sites in the first group had.

Figure 3-7 also shows clearly that sites that started late and/or collected ACSI surveys slowly had not conducted many activities. If sites started late, they needed to have high traffic volume to reach the threshold for a report. Many simply did not have sufficient time to generate the level of response to participate fully in ACSI activities.

The activities that sites had completed are related to several longer term outcome measures discussed in section 3.3 Longer Term Outcomes.

3.2 Intermediate Outcomes Associated With ACSI Use

The original evaluation plan included addressing both intermediate outcomes and longer term outcomes. At the start of the trans-NIH project, the length of an improvement cycle was expected to be less than a year (whether that improvement cycle focused on interim changes to the web site or specific pages or even a full redesign). However, because not all sites followed this pattern, a longer period was needed to address the research questions related to outcomes. With supplemental funding, site licenses were extended to allow sites more time to use the ACSI. However, as described above, some sites still did not have adequate time to receive data, make and implement action plans, and monitor effects of any changes. This section of the chapter focuses on intermediate outcome measures, including perceived benefits of ACSI use, acceptance and perceived usefulness of the results, the teams' level of understanding of the activities required to implement and use the ACSI, and teams' satisfaction with the ACSI.

3.2.1 Perceived Benefits of ACSI Use

General results reported here are based on the final survey data for 51 web sites. The interview data provide additional explanation for the responses where noted.

3.2.1.1 Overall Satisfaction With ACSI Use

Web site teams were asked to indicate their level of agreement/disagreement for the overall evaluation item "Our team is satisfied with using the ACSI to help evaluate our site." As shown in Figure 3-8, the majority of teams—more than 70 percent—were positive or neutral about their experiences with the ACSI. Six of the nine teams that were negative about their overall experience represented "late starter" and/or "slow collector" sites. Five teams opted out of responding because of lack of experience.

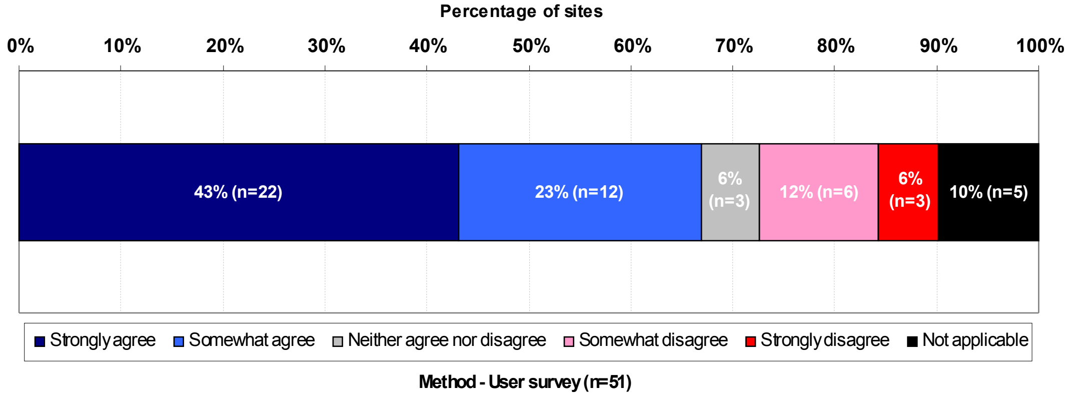

Figure 3-8. Web site teams' overall satisfaction with use of ACSI to evaluate site

3.2.1.2 Met and Unmet Expectations

The in-depth interview provided the opportunity to learn more about the range of benefits achieved and challenges faced by web site teams. Teams described both the ways in which their expectations for ACSI use were exceeded and the ways in which ACSI experiences fell short of their

expectations. At a general level, coding of these data indicated that expectations were met or exceeded for 13 of 20 teams (see Figure 3-9). None of the sites reported experiencing major problems.

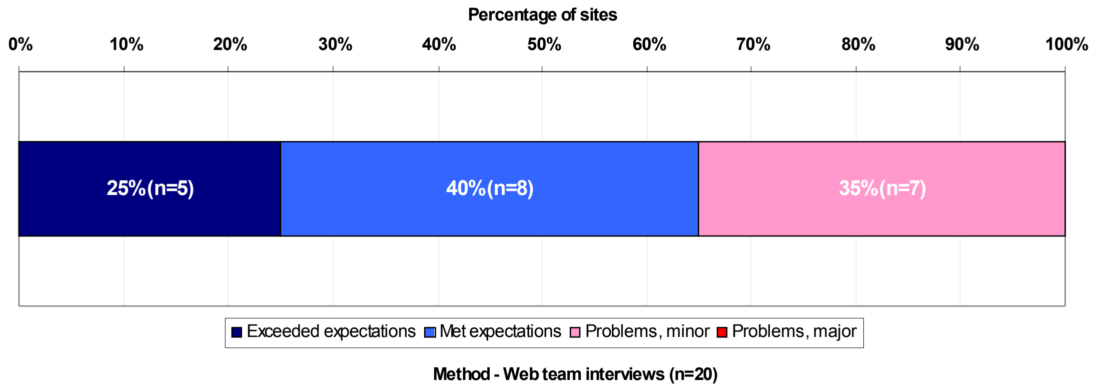

Figure 3-9. Web site teams' judgments of whether ACSI use met their expectations

Web site teams' comments about specific expectations exceeded and not met were coded into categories. Table 3-1 lists these coded comments and the numbers of teams that mentioned them. Comments indicate that teams took advantage of the expected strengths of the ACSI; it provided them with a continuous measure of customer satisfaction, a profile of audience segments, analysis techniques to obtain more detail about the data, and a means of identifying site problems and issues that warrant further investigation. Consistent with findings cited above, some teams' expectations for SRA performance were not met, several did not achieve the scores they hoped for initially or as a result of site changes, several had concerns about the ACSI sampling and response rates, and a few mentioned difficulties related to interpreting results (in some cases due to time constraints). One-quarter of these teams reported having two to four SRAs during their time of using the ACSI. A representative from one of the five teams that expressed disappointment that their investment in making site changes did not pay off in a marked satisfaction score increase said, "Our statisticians don't think that one point change is significant."

Table 3-1. Web site teams' coded comments: Ways in which the ACSI exceeded and fell short of expectations

Coded Comment	Number of Teams
**Expectations exceeded**	
Identification/confirmation of important factors about site audiences	14
Identification of site component/feature/content to change	8
Identification of important factors about site structure/content	6
Mechanism for continuing customer satisfaction measurement already approved by OMB	5
Segmentation	3
Initial scores higher than expected	3
Allowed pre- vs. post-comparison of site versions	3
SRA support	1
Provided good public relations for focus on customer satisfaction	1
**Fell short of expectations**	
SRA support did not meet needs	7
SRA turnover	5
Site changes did not produce expected/desired Satisfaction Score change	5
Response rates were of concern	4
Scores not what team expected	3
Time constraints affected interpretation of data	3
Sampling approach was of concern	2
Scores were skewed based on audience opinions about site content	2
Start-up was difficult	1
Interpreting results was difficult	1
Results did not help to inform site revisions/management	1
Reporting interface was inflexible	1

3.2.1.3 Usefulness of Custom Questions in Web Site Evaluation

An integral part of ACSI use for web site teams was working with their set of custom questions to learn more about visits and visitors. In the interviews, teams were asked to describe how they used the custom questions. They mentioned doing any or all of the following: dropping questions when they no longer yielded new or useful information, revising/tightening the wording of questions to get more specific information where it was difficult to interpret, adding new questions to address issues that had surfaced in previous feedback reports, or quickly posting time-sensitive questions to investigate effects of news items or national/international events on site use.

A final survey item asked teams to provide their level of agreement or disagreement with the statement "Our team has found the custom questions to be useful in evaluating our site." The top bar in

Figure 3-10 shows that 40 of the 42 site teams that had the opportunity to work with their custom questions were positive about their usefulness for site evaluation. During the in-depth interviews, many teams expressed the view that the custom questions provided more value than the model questions. Teams said they paid more attention to the custom question data than the model question data; they felt that these results related more specifically to their site than did the responses to the model questions. For example, a member of one team said “I believe too many of the standard ACSI questions are not appropriate (or not well-worded) for government sites and do not yield useful information.” Teams liked the flexibility and creativity provided through use of both closed-ended and open-ended questions. Teams cited many benefits of using custom questions, including learning about the following:

- Demographic characteristics and satisfaction levels of key audience segments;
- What visitors could not find on the site when searching;
- Content areas to consider adding, based on what visitors were hoping to find;
- Content areas that visitors want but that are not within the scope or mission of the site, indicating a need to add explicit links to external sites that provide that content; and
- Their site’s areas of strength and weakness.

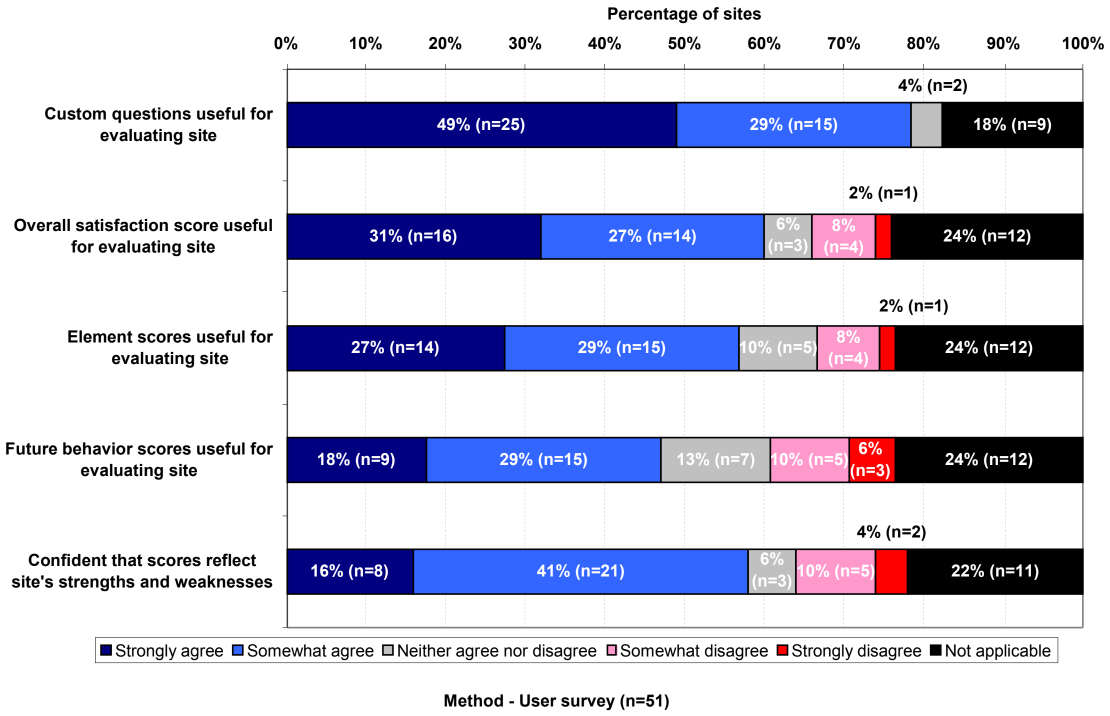

Figure 3-10. Usefulness of custom questions and ACSI scores

Web site teams talked about the benefits provided specifically by the open-ended question data. They mentioned using qualitative response data in the following ways:

- Sending applicable responses to content teams to make them more aware of what visitors are looking for when they come to the site. The content experts are then able to consider information needs of target and unintended audiences and make informed decisions about whether to expand their sites to fill those needs.
- Providing lists of qualitative responses to usability staff or contractors. Input can be used in developing scenarios/tasks for investigating navigation issues for each key audience segment.
- Comparing descriptions of content searched for but not found to mappings of site content. These comparisons clarify whether visitors cannot find something or the content searched for does not exist.

In an in-depth interview, one team did question the value of the custom questions and wondered how much weight to give the results. The observation was that a review of the feedback revealed flaws in how the questions were worded. Approximately one quarter of the teams said that the qualitative responses provided opposing viewpoints rather than clear direction on what site improvements were needed.

3.2.1.4 Perceived Usefulness of ACSI Score Data

ACSI reports provide web site teams with overall Satisfaction Scores, scores on the individual elements such as Search and Navigation, and predicted Future Behavior Scores. One of the important research questions was to examine teams' perceptions of these scores: Are the scores meaningful? Do these scores help teams in evaluating their sites? The final survey included several items addressing teams' judgments about how useful the Satisfaction, Element, and Future Behavior Scores were, and whether the teams were confident that these scores reflected their sites' strengths and weaknesses.

Figure 3-10 also presents these results. The majority of teams were positive about the use of Satisfaction and Element Scores for evaluation purposes (30 and 29 teams, respectively). Fewer teams were positive about the use of the Future Behavior Scores; 15 of the 51 total surveyed were either neutral or negative about these. In the interviews, several teams expressed some frustration about having higher Future Behavior Scores than Satisfaction Scores; this made them doubt or pay less attention to these

scores. For example: “It is not useful for me to know [the percentage who] are likely to return and [the percentage who] are likely to recommend when I know my site is the only source of [information], so users have no choice.” While 29 of the teams indicated that they had confidence that the scores reflected site strengths and weaknesses, the pattern of “Strongly agree” and “Somewhat agree” responses indicates that teams’ opinions were more similar to their opinions about Future Behavior Scores.

3.2.1.5 Satisfaction With Segmentation

The in-depth interview provided an opportunity to hear how web site teams used this capability to learn more about specific audiences, how visitors used the site, and how satisfied they were with their visits. Figure 3-11 shows that 80% of the site teams were very satisfied or satisfied with segmentation. One respondent stated that segmentation was “one of the brightest aspects” of ACSI use for the team. In total, 17 of the 20 teams were neutral or positive about segmentation, and none expressed a negative opinion. Three sites had not used the segmentation capability or had not yet progressed to the point with their segmentation activities to provide a judgment.

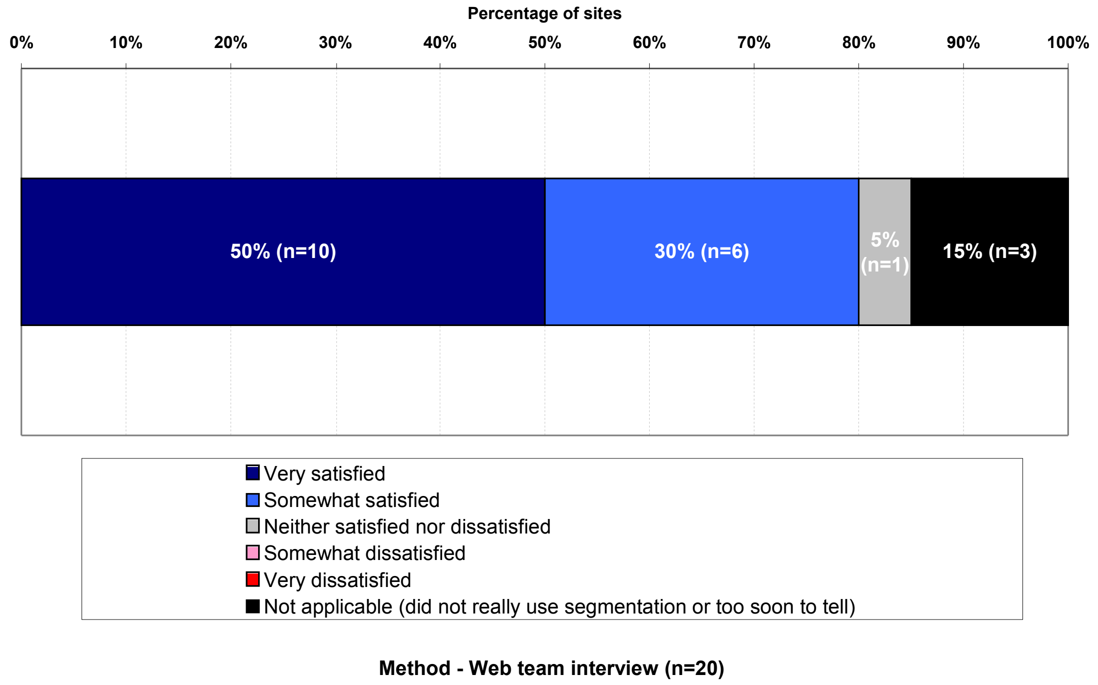

Figure 3-11. Web site teams’ satisfaction with segmentation

3.2.2 Reported Uses of ACSI Data

3.2.2.1 Planned vs. Actual Use of ACSI Data

As web site teams gained experience in interpreting ACSI results, they may have learned additional ways to use their data or found that they could not use their data as expected. A final survey item asked "How is your team using the ACSI survey data for your site?" The initial survey included essentially the same item and response choices, except that teams were asked how they planned to use the ACSI results. Figure 3-12 compares the frequencies of responses for teams' planned vs. actual use of the ACSI.

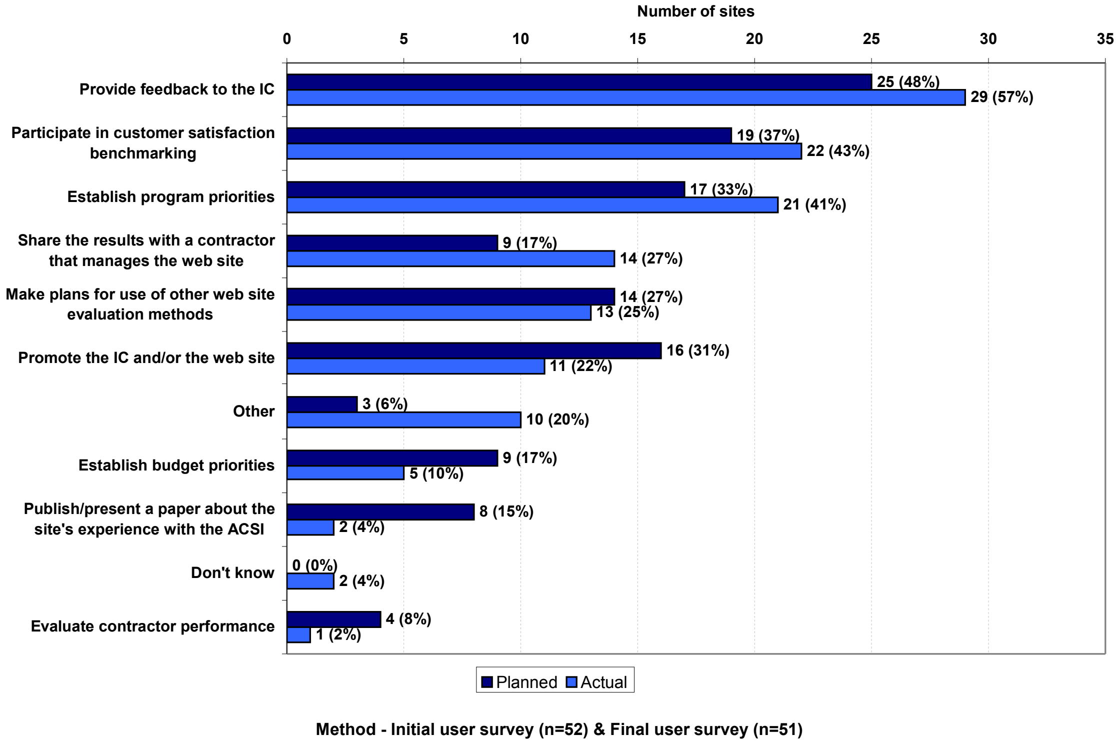

Figure 3-12. Web site teams' planned vs. actual use of ACSI data

More teams actually used the ACSI for the most frequent activities than had planned to do so. These activities included providing feedback to the IC/ODO, participating in customer satisfaction benchmarking (which was a required activity for participating in the trans-NIH effort), establishing program priorities, and sharing results with a contractor that managed the site. These results show that teams derived benefits from the ACSI that they had not anticipated, and many of these were the most popular uses.

Ten teams reported "other" ways that they were actually using data; these responses included the following uses:

- Using the demographic data for overall program evaluation, beyond just evaluation of the web site;
- Evaluating the need for, informing decisions about, and/or evaluating improvements after site redesign; and
- Sharing results with other ICs/ODOs that contribute content to the site.

A critical activity that required time and effort from web site teams was to move from interpreting ACSI feedback to actually making decisions and planning how to act on the data. In some cases, additional steps (e.g., collecting data with other custom questions, conducting additional segmentation analyses to examine the data at a more specific level, looking at other data sources to corroborate information) were required in order to qualify and quantify problems. When the issues were fully understood, then teams identified priorities and made decisions. In some cases, teams decided to continue to monitor an issue before making a decision or that the issue was not worth further attention.

One final survey item specifically addressed this planning component of ACSI use. Figure 3-13 shows the types of site improvements that teams reported planning on the basis of their ACSI data. Three of the four most commonly reported improvement plans were for Functionality, Navigation, and Search, three elements in the "Top priority" area of the ForeSee Priority Map for many sites.

This finding indicates that approximately one-third to one-half of the teams have attended to the ACSI elements with lower scores and higher impact on customer satisfaction. Many sites have also attended to their content, the site's overall look and feel, and redesign of their homepage and/or subordinate pages. ACSI data had been used to plan improvements in site performance for four sites; six other teams responded that they had not used the ACSI in planning site changes. One team reported using the data to plan priorities for site features and another team shared its data with a contractor to plan for usability testing.

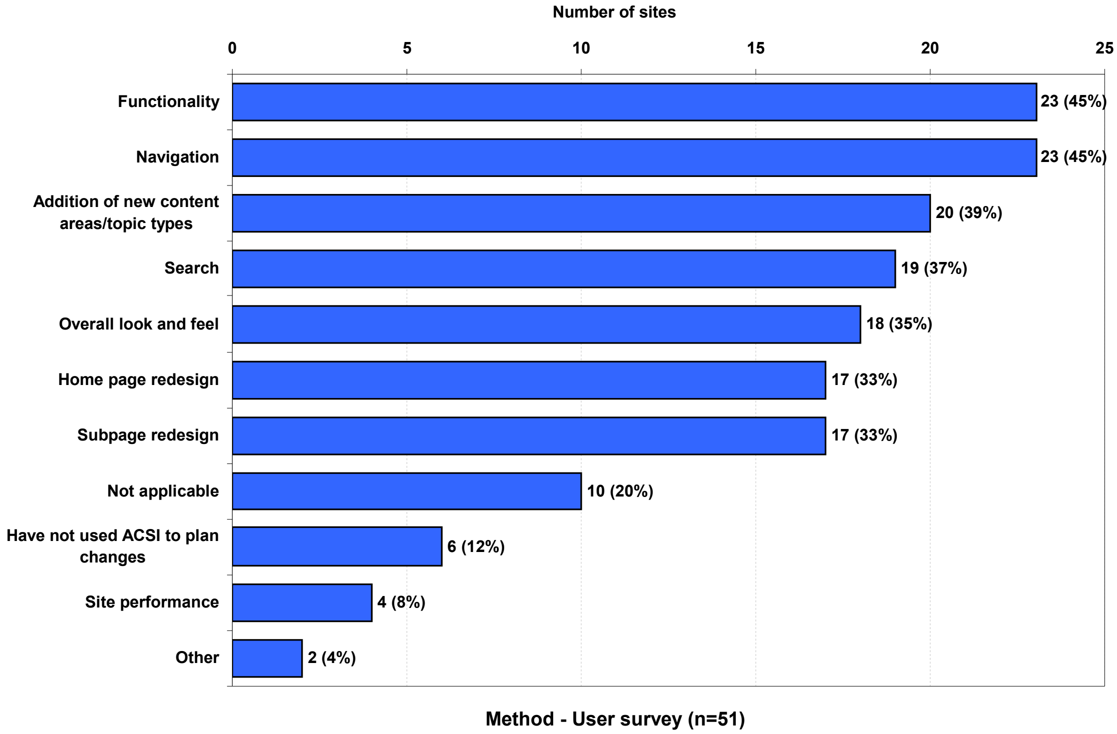

Figure 3-13. Types of site improvements planned using ACSI data

3.2.2.2 Use of ACSI Data in Next Web Site Redesign

At the time the final survey was administered, some of the web site teams had made minor site changes or implemented a more involved site redesign. All teams were asked about their plans for using their data in their next redesign, whether that was in the short-term or long-term plans. Figure 3-14 shows that more than half of the 51 teams plan to use the data in their next redesign. Only 3 teams said no; 13 were not sure, and 7 did not make a judgment because they had not seen their ACSI data yet. In the in-depth interviews, many teams specifically mentioned using their custom question responses, especially responses to the open-ended questions, to generate ideas for making site changes. Several teams said that even if they did not currently have time to review their data, they would save it for when they did have time to review the details. Teams that had not attended closely to the data said that even after their licenses run out, they would rely on the data they had already amassed rather than collect additional data.

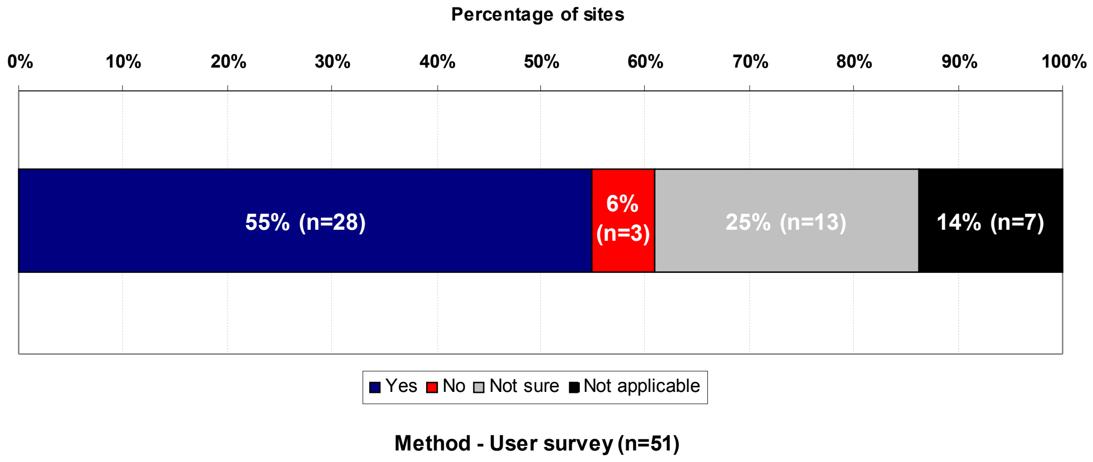

Figure 3-14. Web site teams' plans for using ACSI data for next redesign

3.2.2.3 Use of Data to Make Web Site Changes

Interviewed web site teams were asked to describe the types of changes they made to their sites, based on ACSI data. The following are examples of site changes that teams attributed to their ACSI use:

- Changing search results pages to offer alternative next steps for visitors who receive no results or unsatisfactory results;
- Redesigning a home page based on an audience or hybrid structure rather than content areas;
- Redesigning pages to highlight content that is typically used by audience groups;
- Redesigning a major content section and re-branding the pages to more fully integrate them into the site; and
- Adding explicit instruction on the home page about where (external websites) to find desired content that is <u>not</u> part of the site.

3.2.2.4 Barriers to Making Planned Web Site Changes

As shown in Figure 3-7, 35 of 51 surveyed web site teams used ACSI data to plan site changes, but only 21 of the 51 sites reported actually making those planned changes. A multiple choice

item was included on the survey to assess what types of barriers prevented teams from actually implementing some or all of the changes they would like to make. Results shown in Figure 3-15 indicate the following:

- Staff time was the main type of constraint cited. Although 24 teams reported a shortage of staff time to attend to the data, several teams indicated in the interviews that they have responded to this need by making arrangements to hire or make staff available for web site evaluation activities.

- Fourteen teams reported not making changes because of financial constraints. During the interviews, several of these teams clarified that their web sites had no budget to devote to changes.

- Thirteen teams did not make a judgment about barriers because they had not yet used ACSI results to make site changes.

- Nine teams reported that they faced no barriers in implementing their planned changes.

- Eight teams reported that they had not had sufficient calendar time to implement their planned changes. In some ICs, teams have to request funds and/or make a strong case for site revisions; in others, they must complete a budget cycle before they can implement their plans. In these cases, the required additional time extends the improvement cycle based on factors external to the team and site.

- The "other" types of barriers reported by one or more of the six teams include lack of new content, technical limitations on how the site can function, management opinion about the site, the content development and approval process, and a lack of clarity about what changes are indicated by the ACSI survey results.

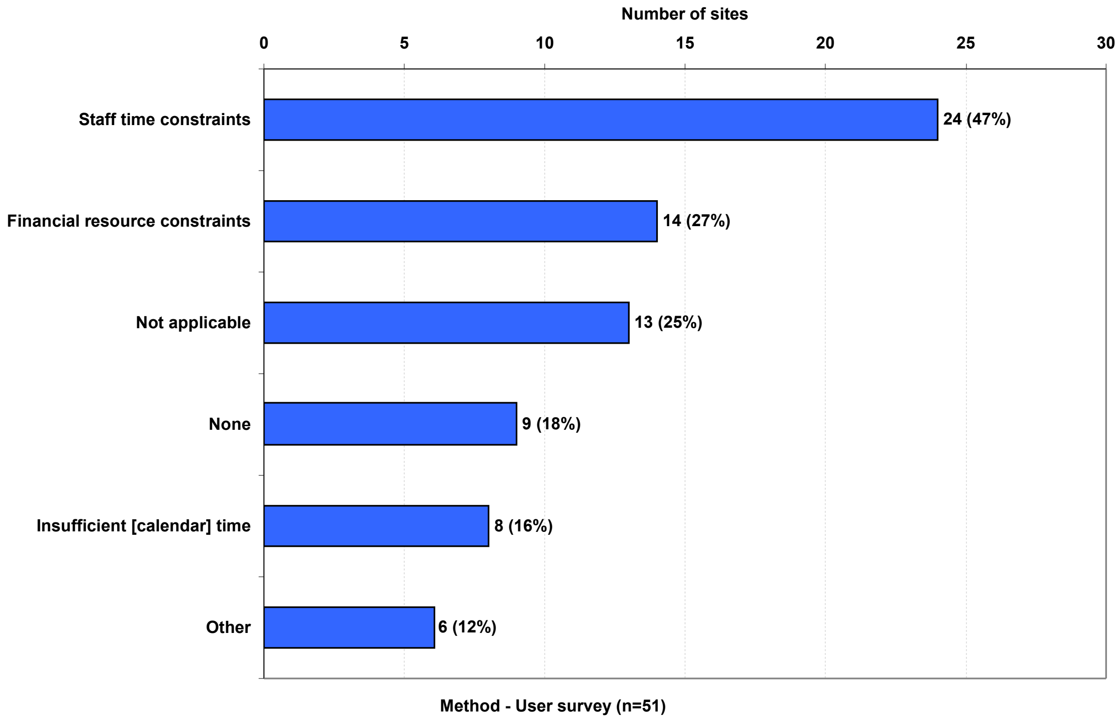

Figure 3-15. Barriers to making changes to site

3.2.2.5 Use of Benchmarking Data

The ways in which teams reported using ACSI scores for benchmarking are shown in Figure 3-16. Although ForeSee provided benchmarking data to sites that surpassed the threshold of 300 completed ACSI surveys, it was up to the teams to decide whether or not to actually use those data. The most frequently reported comparison was the overall index for NIH sites. In the interviews, a number of teams said that they did not have directly comparable sites, especially those that considered themselves to have niche or specialty sites. Those that thought similar sites existed elsewhere in government did say they compared scores. The question was not applicable for 11 teams that had not received ACSI data. Ten teams not using their data for benchmarking were either using the license on a specific subset of pages (limited application) or were those teams that reported not having time to look at their data. Only 3 teams reported making comparisons with private sector sites; most teams mentioned that parallels did not exist in the private sector.

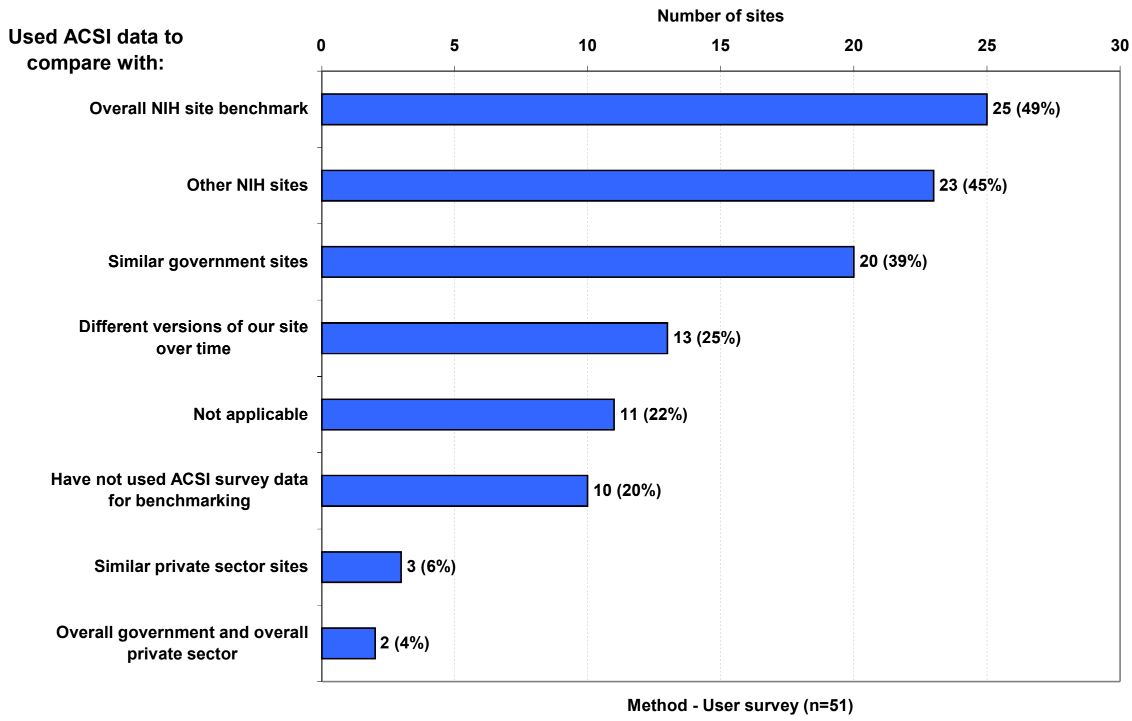

Figure 3-16. Reported uses of ACSI data for benchmarking

During the interviews, teams elaborated on how they took advantage of having other scores available. Several teams mentioned that they did the following:

- Looked at high-scoring sites to get ideas for design and function, as input to making plans for a redesign;
- Looked for consistent high scores within their IC/ODO;
- Ensured that the site was achieving comparable overall satisfaction scores with other government sites within the same ForeSee general classification; and
- Compared their site with sites for other agencies that have similar functions.

3.2.3 Impact of ACSI Use on Web Site Teams' Use of Other Site Evaluation Tools and Methods

Another important intermediate outcome was the degree to which using the ACSI affected web site teams' views of using other web site evaluation tools and methods. Survey respondents were asked to indicate whether they had substituted the ACSI for some other evaluation method(s), whether

they used the ACSI to strengthen their use of evaluation methods (i.e., existing methods), or whether the ACSI had not affected use of other evaluation methods. More than half of the teams (29) responded that the ACSI had no impact on the team's current evaluation practices (Figure 3-17), and 21 of the teams said that ACSI use strengthened their current evaluation practices.

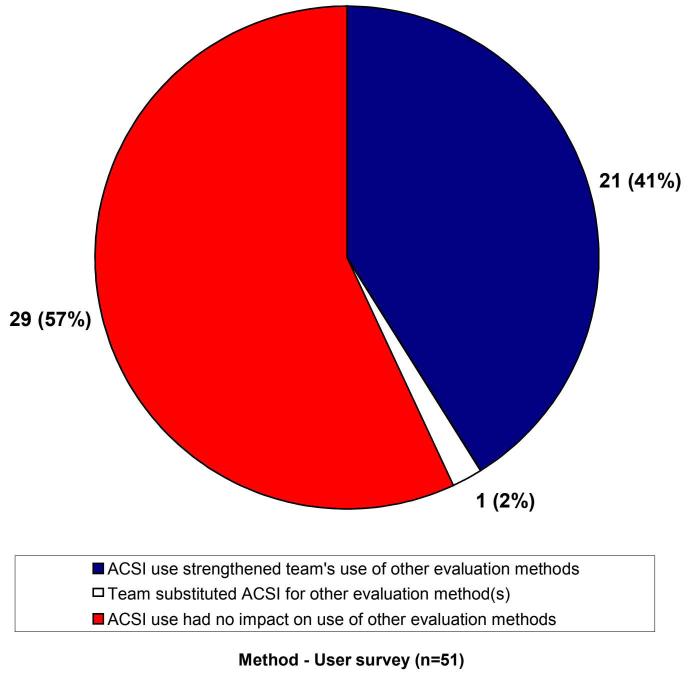

Figure 3-17. Impact of ACSI on use of other evaluation methods

The 21 web site teams that added the ACSI to their evaluation methods were also asked to indicate the other methods they currently used. As shown in Figure 3-18, all but two of these 21 teams also reviewed results provided by web log software; 18 were using usability testing as well. Whereas more than half (11) of the sites had used expert or heuristic review, relatively few (4) had used other types of surveys or ways to learn about their audiences. Four sites had used the focus group method, and one had used search engine reports (an "other" response) to evaluate how the site search is used.

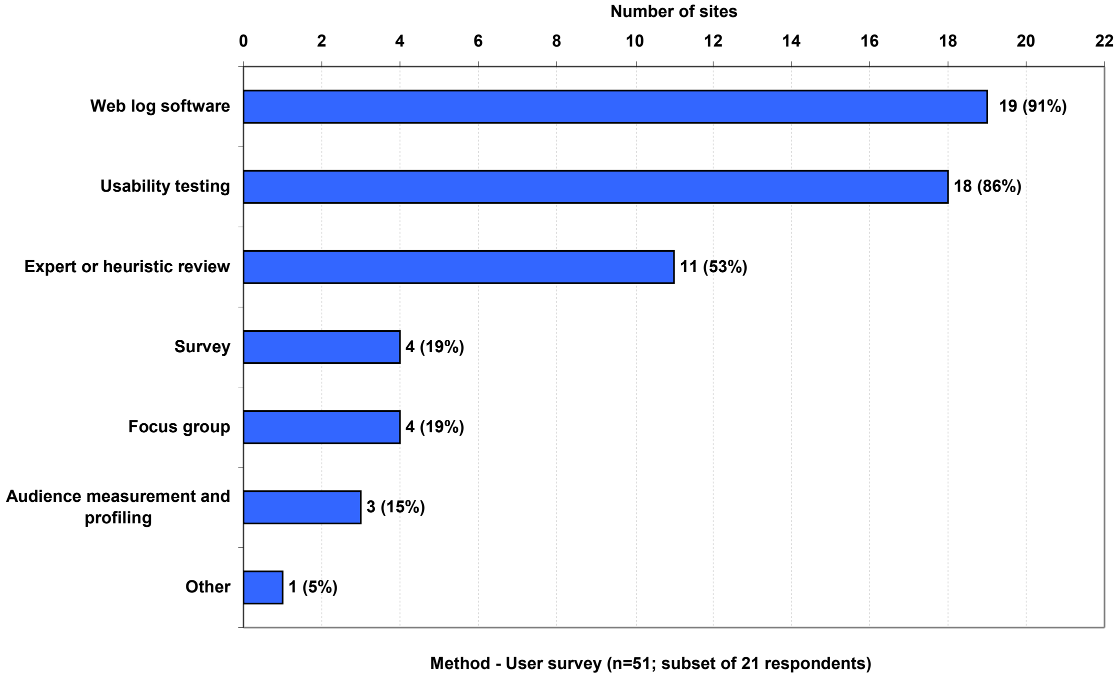

Figure 3-18. Reported evaluation methods used with ACSI (for subset of web site teams using ACSI in combination with other evaluation methods)

3.3 Longer Term Outcomes

Because there was wide variation across sites in how the ACSI was used and how long it was used, sites achieved different benefits from use of the ACSI. Changes to ACSI scores were considered to be among the long-term outcome measures for web sites. Some teams focused on tracking score changes after making site changes. However, partly because of the length of quality improvement cycles, some teams may not have intended or expected to show improvements in longer-term outcomes (such as higher customer Satisfaction Scores) by the end of the evaluation period. Instead, they focused on the benefits of ACSI use that are not represented in score changes. ForeSee states that sites with low scores have a greater potential for showing improvement when they make changes. However, sites with good scores are also expected to benefit from using the ACSI without changes to their scores per se.

3.3.1 ACSI Satisfaction Score Changes

3.3.1.1 Score Changes Related to Implemented Site Changes

One of the important questions addressed by the trans-NIH level of the evaluation was whether site changes that teams implemented were associated with any changes in ACSI scores. Results shown on the left of Figure 3-19 indicate that sites that were changed based on ACSI data had a positive mean score change (mean of 0.79 points), whereas sites that did not implement changes showed a mean score decline.

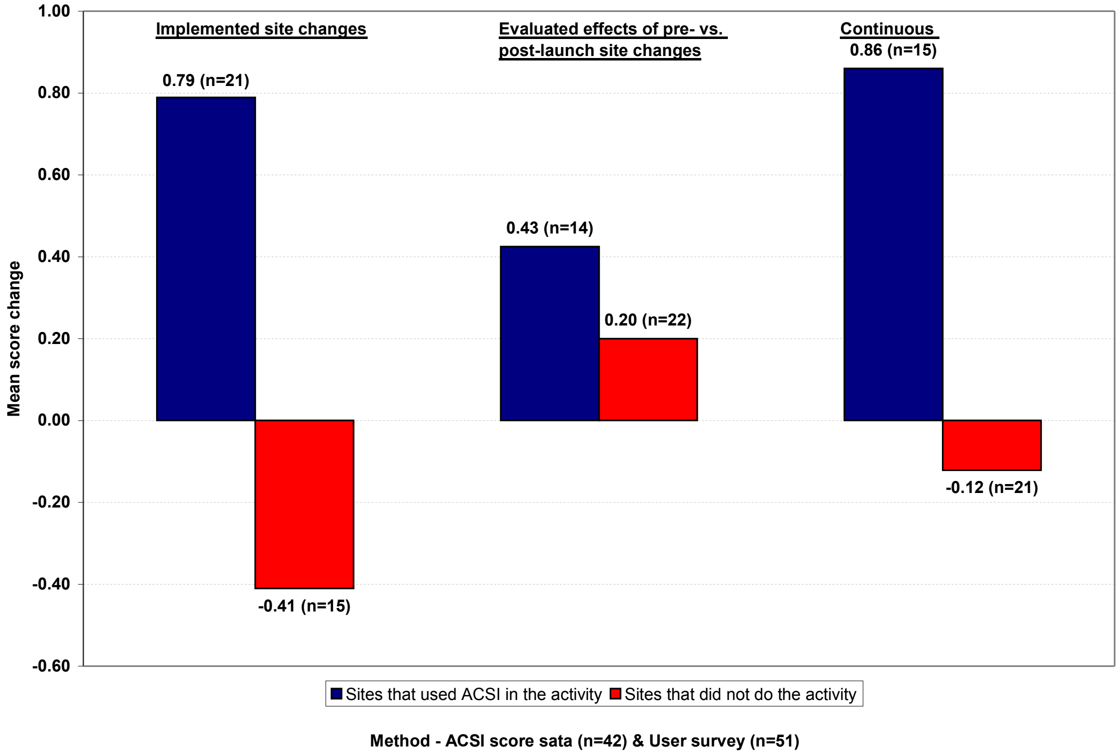

Figure 3-19. Mean satisfaction score changes based on use of ACSI

To investigate whether teams' web site revisions led to changes in ACSI satisfaction scores, Westat first computed mean score change per quarter for the 36 sites that had both responded to the final survey and posted at least two quarters of ACSI scores between Q4 2004 and Q1 2006. (The number of quarters used to derive the mean was based on the number of quarters for which each site received scores. The number of quarters that sites posted scores for varied from 2 to 6. Using the mean score change per

quarter controlled for sites having different numbers of quarters of reported ACSI scores.) The 36 sites were then divided into two groups. The first consisted of the 21 sites which had been revised based on ACSI results; the second consisted of 15 sites that had not been revised. Mean scores were computed for each of these two groups and compared, as shown in Figure 3-19. (Because of the small number of sites involved, t-tests were not conducted to test the significance of the difference between means.)

3.3.1.2 Score Changes Related to Other ACSI Activities

Also shown in Figure 3-19 are results of similar analyses to compare mean score changes for the same time period of Q4 2004 through Q1 2006. (As above, the number of quarters used to derive the mean was based on the number of quarters for which each site received scores.) Mean scores changes for the two groups of web sites were compared for the following activities:

- Use of the ACSI for evaluating the effects of pre- vs. post-launch site changes. Here, the mean (of the mean score changes per quarter) for the 14 teams that used the ACSI to monitor score change for site revisions was compared to the similar mean for the 22 teams that did not. Both groups had mean score increases, but the increase was larger for the teams that used the ACSI to evaluate pre- vs. post-launch changes.
- Use of ACSI feedback in a continuous improvement process. The mean for the 15 teams that used the ACSI for continuous improvement was positive, whereas the mean was negative for the 21 teams that did not.

Taken together, these results show a trend toward positive score outcomes for teams using the ACSI as part of their evaluation practices. In concert with the results presented in Figure 3-7, the time element should be considered as well. If teams that have used the ACSI for a short period continue to use it and apply it effectively to implement site changes, they may also realize the positive score trend.

3.3.1.3 Examples of Web Site Improvements That Led to Higher Satisfaction Scores

Previous sections presented the relationship between web site changes and satisfaction scores. In general, interviewed teams indicated that they moved to a more audience-centered design, worked with presentation of their search results, provided explicit instruction about what is/is not on their site, and, where possible, provided additional content for audiences that figure prominently in their audience profile.

To provide more detail about what types of changes are associated with improved satisfaction scores, the following list describes changes to several sites that showed the largest gains between their first “reporting” quarter (the first quarter for which their score was computed by ForeSee) and Q1 2006.

- One team changed the structure of its home page to help audiences identify the most appropriate links to follow.
- One team strengthened its search results page by adding a “Recommended Links” and a “Can’t Find It” box, with links to other NIH search engines.
- One team modified its search results page to offer users who got no results the options of trying another NIH site resource or trying hints for using other keywords.
- One team integrated a major content section (targeted at the primary audience) fully into the site and re-branded pages.

3.3.2 Impact of ACSI Use on Management Approach

Interviewed web site teams were asked to characterize the type of support their IC/ODO management provided for their use of the ACSI; these responses were coded into categories of Yes, Somewhat supportive, No, and Don’t know. Results shown in Figure 3-20 indicate that 60 percent of the teams (12) thought their ACSI use was supported, 5 teams thought that their management support was mixed, and 2 said there definitely was no support. One team was not sure if the IC/ODO management knew they were using the ACSI and therefore did not know whether there was support for its use.

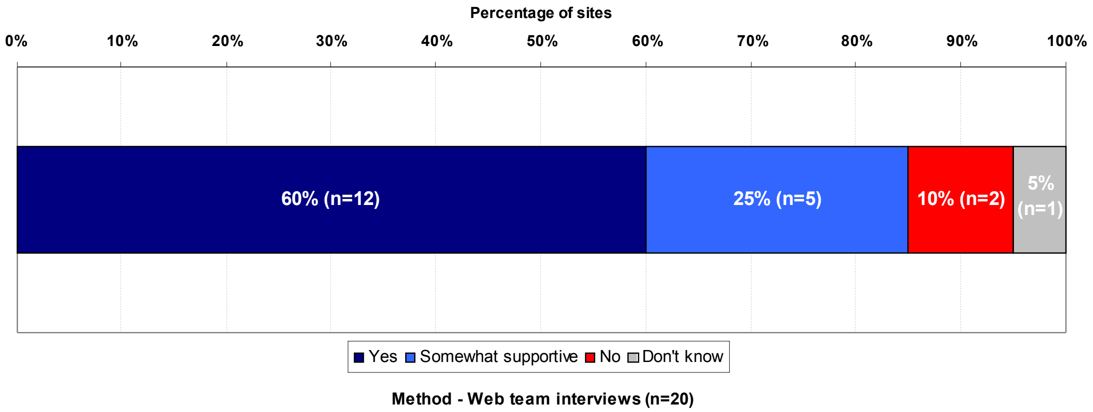

Figure 3-20. IC and ODO management support for ACSI use

One web site team reported that the management was hesitant to accept the results because of skepticism about the sample: “My management is unconvinced that the ForeSee results are representative of our actual user base. As a result, we essentially don’t use the ACSI except for anecdotal evidence from the open-text questions, when looking to prioritize future feature additions.”

Teams were asked to describe how their management indicated their support for ACSI use. Approximately one-third of the teams mentioned that management showed support by reporting and publicizing their scores and using them for public relations purposes (e.g., news items on the sites, facts in newsletter articles) and/or by giving them recognition when sites registered high scores or score improvements. Two teams said that management showed support by providing more resources for web analytics work.

3.3.3 Impact of Web Sites Using ACSI

Web site teams that used the ACSI for long periods reported a high level of satisfaction with it and derived benefits from its use (i.e., incorporated it into their evaluation “toolbox”). However, several interviewed teams indicated that they had reached a lull in their improvement cycles. They had no immediate plans to make site changes, their scores were relatively stable, and they had little or no staff time to attend to site issues. From their viewpoint, the ACSI would have higher utility if they could use it on an interval or non-continuous schedule (e.g., for 3 months to get an update to previous measures or for several months before and after a re-launch). These teams would prefer to time ACSI use to impending site revisions, when staff could devote time to monitor before and after measures of customer satisfaction.

Westat mapped mean scores for the web sites based on the quarter in which they began reporting scores to address the question of whether sites’ ACSI scores showed the following:

- Increases over time as teams had an opportunity to make changes based on customer satisfaction data or
- Leveling off over time as teams used the ACSI for longer periods.

Four subgroups were defined among the 42 sites that received ACSI scores as of Q1 2006:

- Eight sites that had scores for Q4 2004 (prior to the start of the NIH effort);
- Fifteen sites that received scores starting in the first two quarters of 2005;
- Eleven sites that received scores starting in Q3 2005; and
- Sites that received scores starting in Q4 2005 (4) and Q1 2006 (8).

As Figure 3-21 shows, the eight web sites using the ACSI the longest had a very stable mean ACSI Satisfaction Score. The ICs for these sites had the resources to pay for their sites' ACSI licenses before the start of the trans-NIH effort. Sites that started reporting scores at the beginning of 2005 had a slight mean Satisfaction Score increase over time. Sites that started in Q3 2005 actually started out with a 2-point higher mean and continued that same separation. The very last set of sites had a very low initial mean due to one very low-scoring site. With the addition of four more sites and an improvement in that site's initial low score, the mean Satisfaction Score increased for Q1 2006. ForeSee considers that stable or slightly increasing scores indicate that sites are keeping up with user expectations over time. If further work is done with the NIH sites, it will be important to map the mean scores for future quarters for evidence of any marked trends.

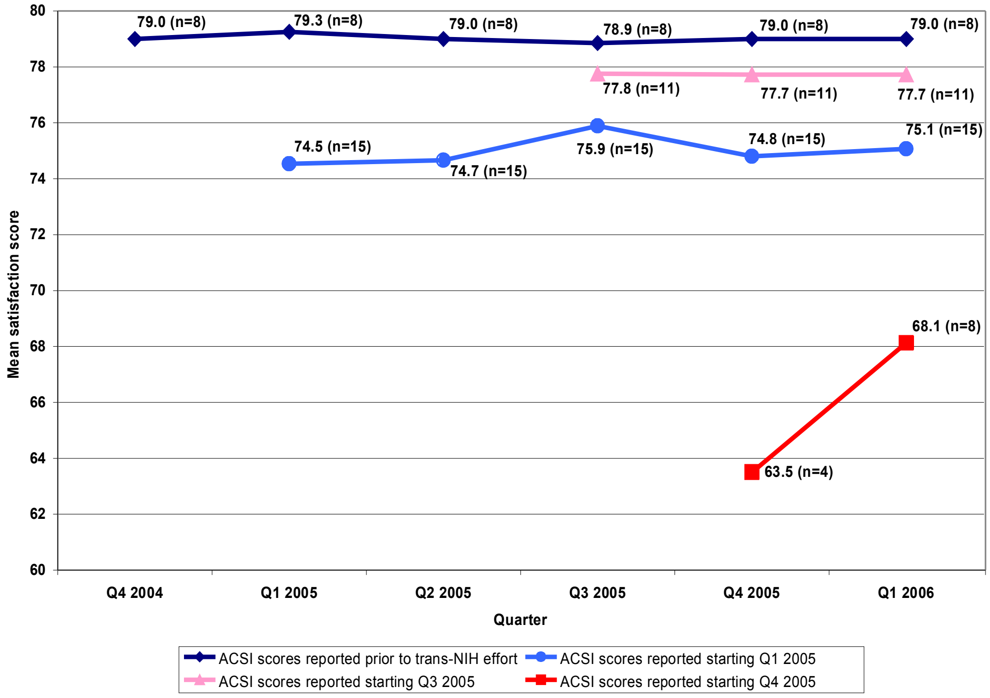

Figure 3-21. Trends in ACSI scores based on quarter of reporting start

3.3.4 Desire of Participating Web Site Teams to Continue Using the ACSI

From the start of the trans-NIH project, the license fees for the participating web sites were fully covered by evaluation set-aside funds. The ICs and ODOs received the benefits of ACSI services without paying a license fee. An item on the final survey asked web site teams to indicate whether they would continue to use the ACSI if the license fee had to be paid from their IC/ODO budget. Response choices were as follows:

a) Yes, on a continuous annual basis
b) Yes, but on a periodic or as-needed basis
c) Not sure
d) No, the ACSI is not of sufficient value to us to continue to use it

The results shown in Figure 3-22 break the 51 web site teams into two groups: teams that started their license term and had their initial feedback meeting by the end of 2005 and the remaining sites that had a late term start date or a slow ACSI collection rate. The survey item for the sites in the second group provided an estimate of the yearly cost of a license ($20,000 - $25,000).

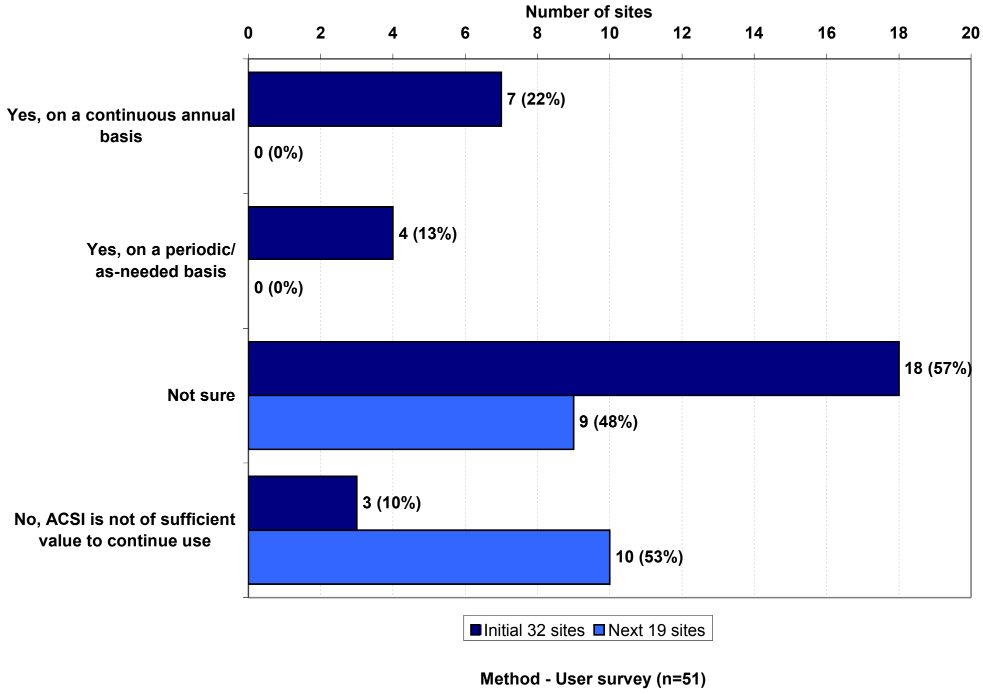

Figure 3-22. Use of ACSI if IC/ODO had to pay license fee

The following findings pertain to the teams using the ACSI the longest:

- Seven teams would continue the current use, expecting or knowing that the IC would pay the license fee. For five of these sites, the license fee was paid from the IC budget before it was covered by the evaluation set-aside funding.

- Three teams responded that the ACSI was not of sufficient value to continue using it if their IC/ODO had to pay the license fee. However, in the follow-up interview, one of these three teams qualified the survey response by saying that they were "on the fence" about continuing use; they considered themselves to be a specialty site and said that sampling issues were a big concern.

- Four of the teams would prefer an arrangement other than a continuous yearly license.

- The 18 teams that responded "Not sure," as well as the 4 teams that would prefer periodic use, were also asked to indicate whether their responses were related to budget constraints, staff constraints, or some other reason (and to specify the reason). Results are shown in Figure 3-23.

    - "Budget constraints" was the reason cited by all teams that indicated some uncertainty about continuing <u>and</u> by 1 to 3 of the 4 teams that would prefer some other period than a one year continuous license. In the interviews, the across-the-board budget concerns of all NIH ICs were mentioned.

    - Teams that were very short on funding for any type of web site work were fairly confident that the IC/ODO would not be able to cover a license fee of approximately $25,000. Teams that knew their IC management supported the ACSI were more confident that funding could be an option.

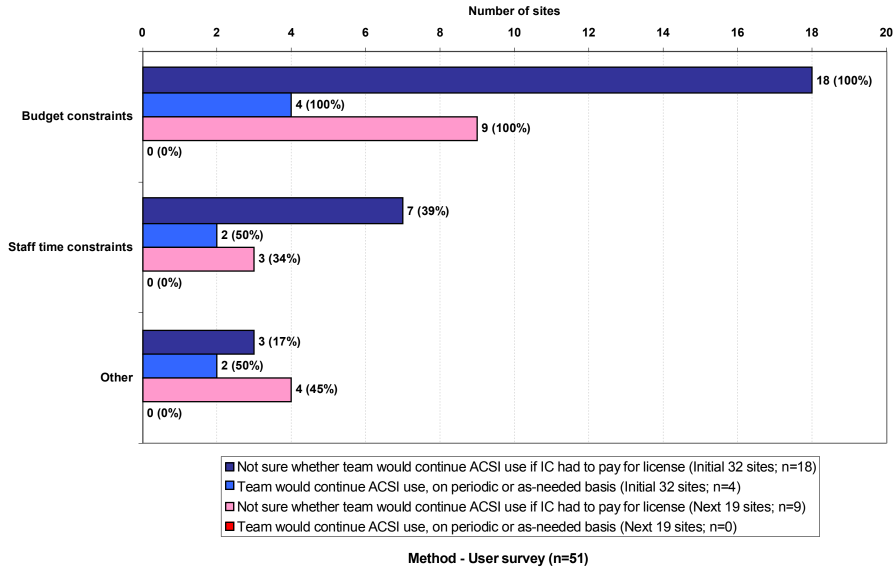

Figure 3-23. Constraints affecting continued use of ACSI

For the 19 teams with the least ACSI experience, the results were much different. None of the teams indicated that they would continue if the license fee had to be paid by the IC; their responses were evenly split, as follows:

- Ten of the sites responded that the ACSI was not of sufficient value to continue a license.
- Nine reported that they were not sure about continuing the license.
- The nine teams that responded "Not sure" were also asked to indicate what constraints were in effect. The pattern here was parallel to that of the sites that used the ACSI for a longer period. These results are also shown in Figure 3-23.
  - Budget constraints were a concern for all nine sites.
  - Three of the teams also cited staff time constraints.
  - The teams also mentioned the following additional constraints:

    "Not sure what actionable evidence there is in ACSI."

    "We haven't received any data, so are unsure of its value."

    "[I'm] not the one who makes budget decisions."

    "Staff concerns vis-à-vis validity of the data and, possibly, response rates."

As mentioned previously, some teams did not have the staff time to devote to reviewing the data they already had. These teams were not sure if it would be worth continuing to collect data unless they could make more staff time available to attend to the feedback. The following are examples of these teams' comments:

- "Now that we have a year's worth of data we are considering stopping the survey. We need to do something with all this data. We hope to start a fresh redesign process this fall and will use ACSI data to make changes then."
- "The most difficult challenge is finding the time to review and digest the wealth of information provided by the survey. We know the open-ended questions provide a gold mine of information but we simply do not have the time for thoughtful, focused review or interpretation. We could really use an executive summary (top bullet points) of the information so that we could more easily assimilate it all."

Four teams mentioned "other" types of constraints that influenced their judgment about continuing use:

- Given a limited budget, usability testing is more likely to be funded than an ACSI license.
- Users are expressing annoyance about getting the pop-up survey (although the use of persistent cookies helped to reduce user annoyance).

3.4 Web Sites That Were Less Successful in Using the ACSI

An important objective of this evaluation was to look for web site characteristics or patterns of site characteristics for which there are issues or cautions in using the ACSI. Given the range of NIH sites involved, and the differences among teams in experiences using the ACSI, there was an expectation that there would be a good fit for many, but not all, sites. The qualitative data collected during both the initial and final interviews with 20 teams have provided the basis for the patterns that have emerged. The following main characteristics are discussed in this section:

- Ability to obtain 300 completed ACSI surveys within a reasonable time;
- Timing of the license period;
- Fit between the web site team and the SRA;
- "Niche" or /specialty sites; and
- Lack of buy-in or support from IC/ODO staff or management.

Westat also looked at web site background variables as a basis for investigating meaningful associations between site characteristics and patterns/differences in Satisfaction Scores over time. These scores were graphed for the following characteristics; figure numbers are provided in parentheses:

- Site traffic volume (Figure 3-24):
  - Low = less than 100,000 average monthly visits;
  - Moderate = 100,000 to 999,999 average monthly visits;
  - High = 1 million to 5 million average monthly visits; and
  - Very high = more than 5 million average monthly visits.

- Full-Time Employees (FTEs, a measure of the level of support for sites) (Figure 3-25)
- Primary audience (based on ForeSee categorization of sites; categories were collapsed into primarily general public, primarily researcher/scientist, or primarily government) (Figure 3-26)
- Primary visitor type (based on ForeSee categorization of sites as primarily used by first-time or repeat visitors) (Figure 3-27)

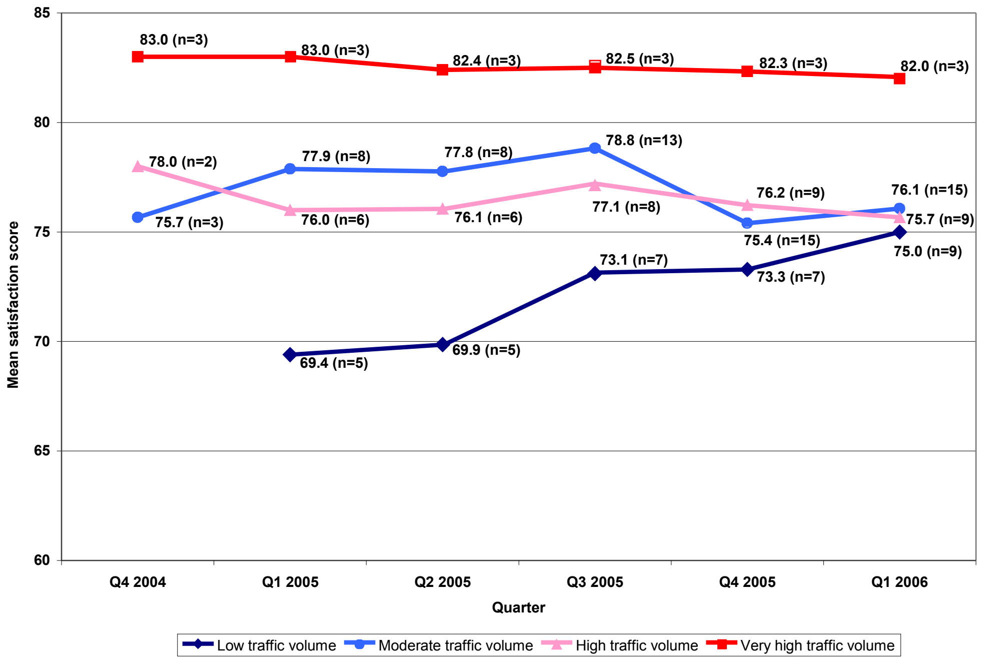

Figure 3-24. Satisfaction scores across time for subgroups of web sites based on traffic volume

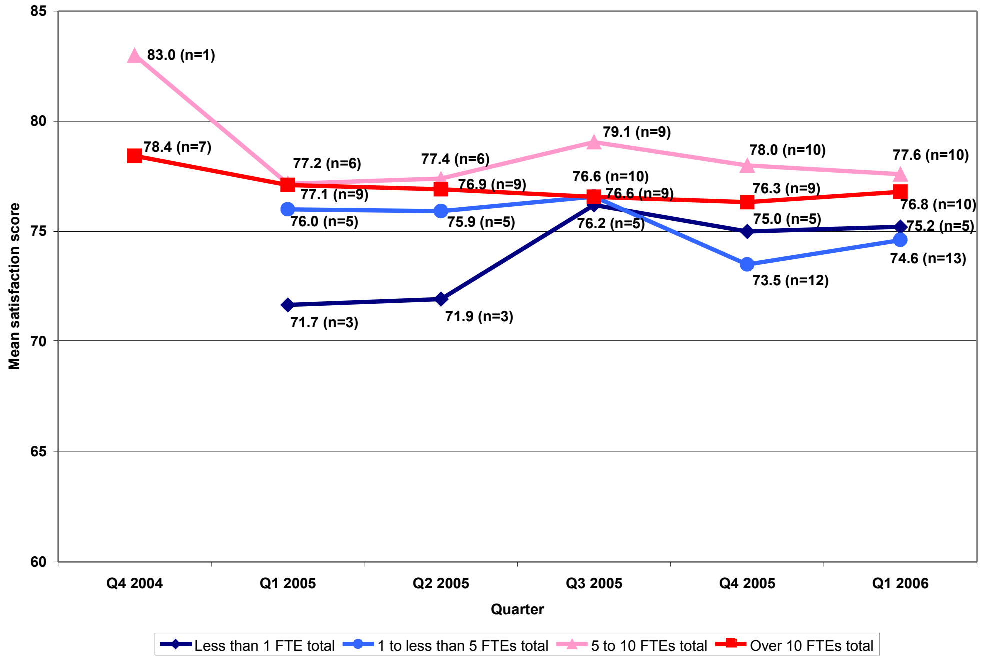

Figure 3-25. Satisfaction scores across time for subgroups of web sites based on FTEs

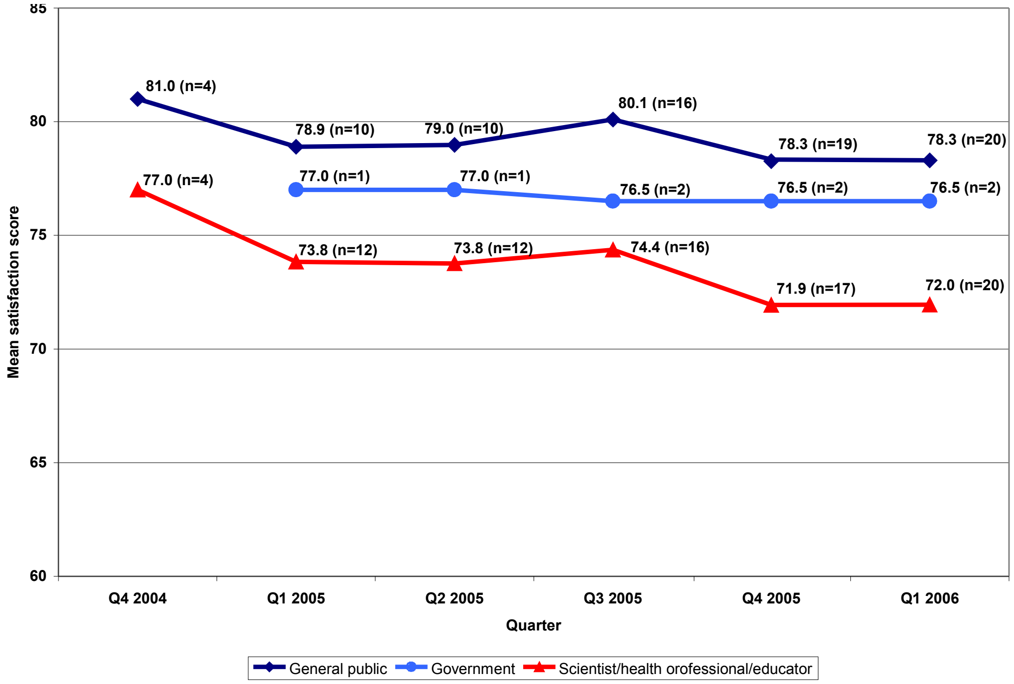

Figure 3-26. Satisfaction scores across time for subgroups of web sites based on primary audience

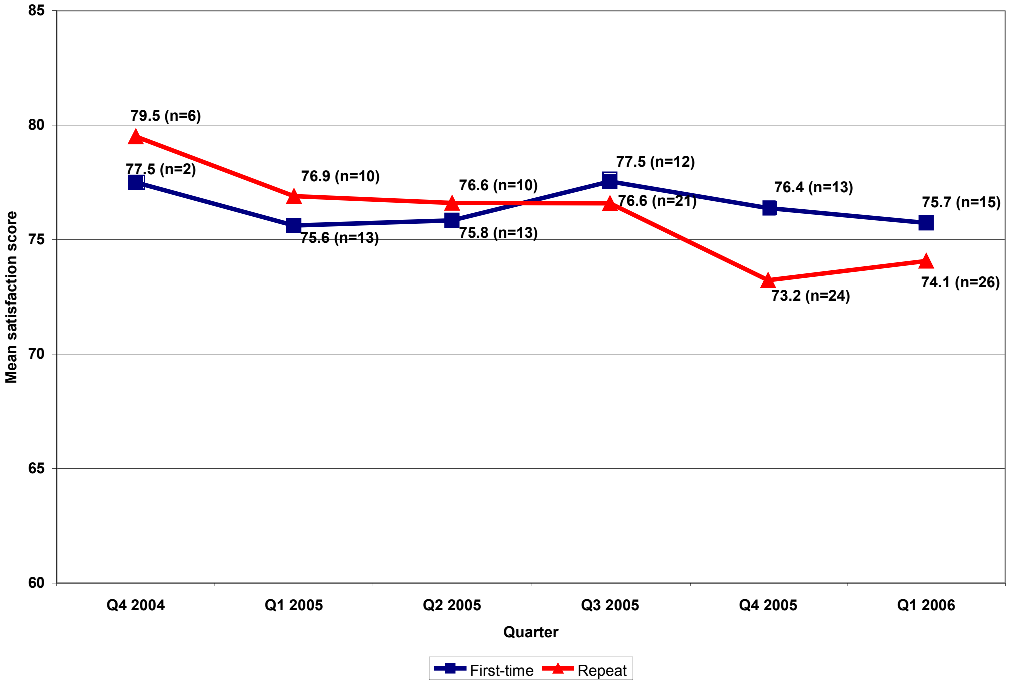

Figure 3-27. Satisfaction scores across time for subgroups based on visit frequency (first-time vs. repeat visitors)

The results shown in Figure 3-24 indicate that web sites with the very highest traffic volume are also those that have the highest mean Satisfaction Scores over time. Means for the last few quarters

for each traffic volume group can be compared with more confidence than means for the first few quarters; these means are less influenced by the addition of new sites for each quarter. The mean Satisfaction Scores for the group of low traffic volume sites increased due to both score increases for the 5 sites that received these scores in Q1 2005 and high initial scores for the 4 sites that started receiving scores in Q4 2005 and Q1 2006.

Results in Figure 3-25 indicate that web sites staffed by the fewest FTEs generally score lower than all other sites. The mean Satisfaction Score for one site with 1 to 5 FTEs accounted for the drop in overall mean for that group in Q4 2005 and Q1 2006. Means for the other three groups were more stable across quarters; these findings can be interpreted with more confidence.

Figure 3-26 shows Satisfaction Scores over time according to primary audience. Scientists and researchers were generally less satisfied with web sites than members of the general public. The scores for the most recent quarters appear to be relatively stable because almost the same numbers of web sites are included in the means. The higher scores for Q4 2004 are due to the smaller numbers of web sites underlying the means.

Visit frequency results in Figure 3-27 indicate a very gradual decline over time in mean Satisfaction Scores for both first-time and repeat visitors. (Repeat visitors are coded here as a combination of frequent and infrequent visitors.)

3.4.1 Timely Collection of 300 Completed ACSI Surveys

Web site population size and composition. The web site population size and audience composition are several site characteristics that accounted for difficulty reaching the target of 300 completed surveys within a "reasonable" time. With a license period of 1 year, there is an expectation that a site will collect enough completed ACSI surveys within 3 to 6 months (which translates to 3 to 6 per day) to move to the next stage of receiving and reviewing feedback. The site characteristics associated with lower numbers of completed surveys are described below.

- **Low traffic volume.** Sites that have fewer than 25,000 to 30,000 average monthly visits may not support the full use of the ACSI model. Sites that have a low traffic volume may collect completed ACSI surveys slowly, even if they have a very high or 100 percent sampling rate and/or a large percentage of repeat visitors.
- **Slow ACSI survey collection rate.** Sites that have some combination of factors such as a low sampling rate, a high percentage of repeat visitors who elect not to take the

survey, and a 60- or 90-day cookie policy may collect only one or two complete surveys every few days.

- **Intranet site.** Intranet sites tend to have lower traffic volume and also have a high percentage of repeat visitors who opt out of taking the survey. Sites that only the NIH community may access are also subject to these same constraints. Interview data also indicated that these audiences are more likely to be irritated by the pop-up survey (which caused one team to conclude that the ACSI visitor profile may misrepresent their user population).

Characterization of sites that started late or collected data slowly. At the end of March 2006, 13 sites had not yet reached the target of 300 completed ACSI surveys. Four of those started in the second half of 2005, but the others were collecting at a very slow rate over the course of approximately a year. Though eight of these sites received a report of their custom question results from ForeSee, they did miss out on analyses provided by the full ACSI model (which requires approximately 300 completed surveys). The question of data reliability when data are collected over such a long interval also suggests that sites that accumulate data below a rate of 3 to 6 per day should not use the ACSI.

Brief interviews were conducted with representatives of five web site teams selected from the 23 sites that had not had an initial feedback meeting by December 2005. These teams were asked to clarify the factors that accounted for a delayed license start and/or a slow ACSI collection rate. The findings are summarized below:

- Of the two sites that started late, one delayed the start due to “other site priorities” and the other delayed due to the reorganization of the IC.
- One site that both started late and collected data very slowly could not meet its intended start date because the single web staff person was out of the office for an extended period. In addition, external site access had to be restricted during much of the license period. (With only internal NIH staff visitors for this period, the traffic volume was substantially reduced.)
- Two sites collected data very slowly because they have internal NIH audiences and a high percentage of repeat users. Anecdotal evidence indicated that the site users were tired of the ACSI pop-up survey.

Teams were also asked what benefits they had achieved from their relatively limited use of the ACSI and in what ways their expectations for ACSI use were and were not met. These teams said they did not achieve the expected benefits of ACSI use. One of these five teams had just received an initial report but did not have the opportunity to thoroughly review or act on the results. The other teams were very disappointed that they had no data reports to show for time spent on start-up efforts. They had expected to be able to use customer satisfaction data for reporting purposes or for assistance with redesign decisions. Three teams questioned the validity of their custom question data because these results were

provided by only a small number of respondents and were somewhat contradictory. Several teams reported receiving complaints about the survey from internal staff.

ACSI coding requirements. Web site teams that had an efficient method for adding code for the pop-up survey to all planned pages reported that this process went relatively smoothly. In several cases, teams took much longer to start using the ACSI than expected. For these web sites, the teams had to apply the code manually to all pages.

3.4.2 Timing of License Period With Web Site Development/Redesign Schedule

An important factor that surfaced during the final interviews was the degree to which the ACSI license period was synchronized with teams' other web site evaluation activities and what stage of development/redesign the teams were in when they started to use the license.

Several teams were able to shift their resources and plan their activities around the license period. Others started to use the license after making some degree of site change. These teams were looking for some type of verification that they scored well as a result of the changes they had already implemented. Although teams were urged to start their term at the time originally planned (in order to have all sites collecting ACSI data concurrently), several sites did delay ACSI implementation to coincide with site changes already planned or to allow for a pre-/post-launch comparison.

Timing of license use was related to the teams' satisfaction with ACSI data. Teams perceived the ACSI to be of value when the license period enabled them to compare pre- vs. post-launch scores for their web sites. Leading up to a revision, teams had a heightened interest in identifying issues (e.g., navigation, content), collecting qualitative data through open-ended custom questions, and comparing pre- vs. post-launch scores and qualitative responses over time. A member of one team that did not synchronize the free license period with its site schedule commented, "We would also like to have ACSI data after our site redesign is complete (it's in the planning stages now), but that will be after the trans-NIH contract is done."

Teams whose license terms were out of synch with their other evaluation methods reported that they paid more attention to these other data sources. For example, a team started the license period after redesigning their web site based on usability testing. The team indicated that the ACSI results did not yield new information about visitors; the team paid more attention to specific usability testing results because these provided more guidance about what to change on the site.

Teams that were not planning to make site revisions in the near future (because of budget constraints or other factors) reported that they were collecting a lot of data. Although these teams may not have given much attention to data review, they were storing the data for use in a future redesign. These teams specifically mentioned saving the responses to open-ended custom questions for later use.

3.4.3 Fit of Web Site Team and SRA

As mentioned in section 3.1.3 Perceived Value of Review Meetings and Satisfaction Research Analysts, some web site teams were very pleased about the working relationship with their SRAs. Others had the opposite experience. The factors associated with negative experiences were high SRA turnover or working with inexperienced SRAs during initial stages when more help was needed to fine-tune custom questions and explore ways of conducting segmentation.

In addition, some teams seemed to expect that their SRA would identify ways to improve the problem areas of their sites: "ACSI has helped us document the need to improve our site (and the need to spend staff time and dollars), identify our users, and identify which dimensions of the site are most in need of improvement. ACSI had been less useful in helping us determine why certain elements of the site aren't working and how to improve them. More help from ForeSee on how best to use ACSI data would be useful." These teams may have misunderstood the role of the SRAs or not been informed that SRAs would not suggest specific site changes.

3.4.4 "Niche" or Specialty Web Sites

"Niche" web sites are sites that by intent have a limited audience or a limited scope. Members of teams managing these sites suggested that, because of their unique function and mission, the sites do not fit the ACSI model as well as more general information dissemination sites. Several of these teams commented that the model questions did not seem very applicable for their sites. They relied heavily on the custom questions instead and did not place much value on the scores generated by the model (Satisfaction Scores, Element Scores, Future Behavior Scores).

Several of these niche sites had lower than expected scores from their main audiences. The teams suspected that scores were artificially low and did not truly reflect site quality. They investigated the underlying reasons using segmentation analyses and determined that their audiences had content expectations that diverged widely from content dictated by the site missions. For example, one team stated that one of the site's main visitor audiences does not agree with the site message content (education

from a scientific viewpoint, not prevention or abstinence). Another example is a site that has a narrow mission to provide information about NIH's role in funding research; site visitors expected to find current news events and ethical viewpoints.

3.4.5 Lack of Support From IC Staff or Management

In several cases, web site teams said they placed no stock in the model-based data and scores because their management initially distrusted the methodology based on their views of convenience sampling and expected response rates. Several teams indicated that their statistical staff (as internal stakeholders) were reluctant to accept the ACSI score data because of skepticism about the method that generated the scores. In these cases, statisticians initially voiced reluctance about the approach (including the sampling method, the lack of variability in Satisfaction Scores across sites, the low response rates, the point at which the survey was offered to visitors, the 10-point response scale, the large number of items, and the phrasing of the items). As one team member stated, "The response rate to the survey does not instill confidence in the results. The lack of significant change in scores post-design underscores this." Another team representative said, "The ACSI in general has a horrible response rate" and "The overall score is meaningless." Teams said that once these objections were made public, they were not able to overcome them over the course of the license term.

4. TRANS-NIH RESULTS

This chapter provides results for the second major evaluation focus, the trans-NIH program. A general summary of the results presented in this chapter is provided in Box 3. The objective of the evaluation at the trans-NIH level was to assess the value of using a common metric for customer satisfaction measurement across multiple NIH web sites.

The chapter is divided into the following five results areas:

- Benefits and drawbacks of the trans-NIH program;
- Identification of common site issues and ways to resolve them;
- Demonstration of web sites' contributions to NIH missions;
- Site-specific benefits of the trans-NIH program; and
- Impacts of the trans-NIH program across the U.S. government.

Discussions throughout these sections are based mainly on the qualitative responses teams provided during the in-depth interviews and on observations at trans-NIH meetings. In addition, the first section presents and notes several findings from the final survey.

4.1 Benefits and Drawbacks of the Trans-NIH Program

In exchange for the use of a 1-year ACSI license to measure customer satisfaction, NIH web site teams were expected to attend trans-NIH meetings and participate in the data collection activities conducted as part of the evaluation.

This section focuses on web site teams' perceptions of the value of participating in the simultaneous and phased use of the ACSI. The final survey also included several items that elicited information about meeting attendance and benefits of attending meetings.

Box 3. Summary of Key Results in Chapter 4

1. Overall, NIH web sites meet audience information needs. NIH sites serve a broad range of audiences with diverse information needs. Use of the ACSI allowed NIH to use site-level data to address how well its web sites meet customer information needs, how it is meeting its information dissemination goals, and the satisfaction of various groups of customers with NIH sites.

2. The trans-NIH program provided additional NIH enterprise-wide benefits. Web site teams that attended trans-NIH meetings reported that major benefits were interacting with other NIH teams and ACSI users, learning from the case studies, and learning about factors that accounted for the high scores of other NIH sites. Another benefit of the trans-NIH program was the informal mentoring that took place among teams within several ICs/ODOs.

3. At the trans-NIH level, teams whose web sites had similar features (e.g., audiences, content) could collaboratively explore solutions to their common problems. Common site issues addressed at trans-NIH meetings included low Search Scores, barriers to making site improvements, and ways to direct visitors to more appropriate information sources.

4. Funding and carrying out this large-scale voluntary program across ICs and ODOs shows NIH-level support for web site evaluation. Teams recognized the value of automatic OMB clearance for their customer satisfaction survey, which provided major savings in terms of budget and, in some cases, staff time. The continuous feedback mechanism provided by the custom questions was also considered a large benefit. In the qualitative interviews, teams mentioned that they became more interested in evaluation and customer-centered design as a result of learning more about both their intended and unintended audiences.

5. NIH funding for this evaluation also shows the agency's commitment to meeting the Federal directive to measure customer satisfaction with e-government. During the evaluation period, NIH web sites scored consistently higher than the overall government index.

6. NIH will be able to use the information about site characteristics and contexts for which the ACSI is best suited (see Chapter 3) to guide future decisions about purchasing ACSI licenses and allocating them within ICs/ODOs. In addition, case studies of teams that had successful experiences with the ACSI (whether success was defined as learning about their audiences or increasing Satisfaction Scores) can be shared as best practices for ACSI use.

4.1.1 Meeting Attendance

The LT scheduled and held quarterly meetings to update teams about participating web sites' status and NIH scores relative to various indexes, including the e-government and private sector indexes.

Also, teams that had used the ACSI for longer periods or had successfully implemented site changes shared their experiences. Meeting attendance was evidence of interest in learning from the experiences and successes of other teams. On the final survey, teams indicated whether they had been to none, some, or all 5 of the trans-NIH meetings. Of the 51 teams:

- Only three had not sent a team representative to attend any meetings;
- Forty-one reported that a team representative had been to some of the meetings; and
- Seven teams reported attendance at all of the meetings.

4.1.2 Benefits of Attending Meetings

Web site teams indicated that they benefited in a number of ways from meeting attendance; these data are presented in Figure 4-1. The top four benefits cited were hearing the Satisfaction Scores of other NIH sites, meeting and interacting with their counterparts from other sites, learning more about data interpretation and analysis methods, learning more about the ACSI methodology, and sharing information about their ACSI experiences with other site teams. These responses make a strong case that the trans-NIH project contributed to the development of an information-sharing culture at NIH. It is expected that these relationships will continue to facilitate the exchange of knowledge and practices across NIH web site teams.

4.1.3 Benefits of Participating in the Trans-NIH Project

In the in-depth interviews, web site teams were asked to elaborate on the specific benefits of using the ACSI in a simultaneous process with other NIH teams, what contributed to the success of the trans-NIH project, and what could be improved to make the trans-NIH effort more effective for the teams. The comments of the 20 teams that participated in in-depth interviews were coded into categories. Three-quarters of the teams mentioned that they learned a lot from sharing information among teams. Approximately half of the teams specifically stated that the case studies gave them insights into ways to use the custom questions and segmentation analyses effectively, as well as what types of changes were being made to address site issues.

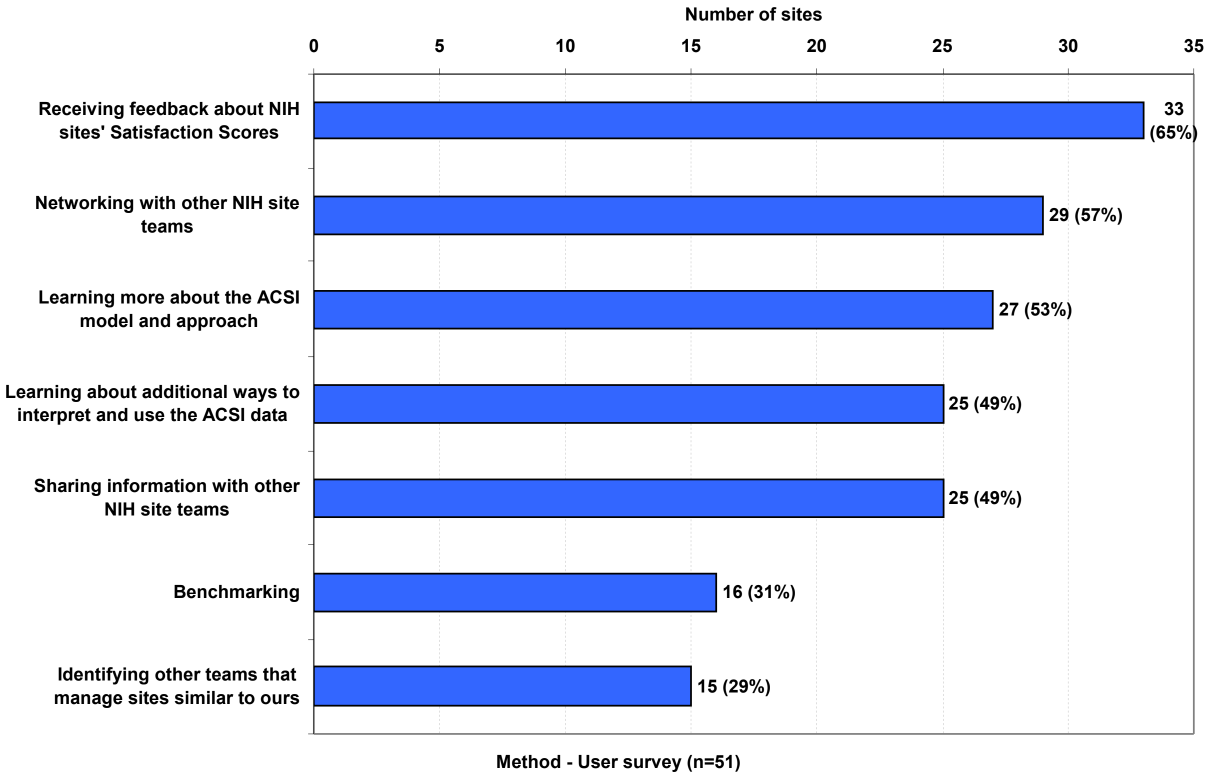

Figure 4-1. Benefits of trans-NIH meetings

The following are specific examples of the types of information teams mentioned hearing at meetings:

- Suggestions for additional ways to use score comparisons beyond the overall site level. (For example, comparing Satisfaction Scores for audience segments to those of other sites with similar audiences.)

- Which NIH sites achieved high Satisfaction Scores. This information enabled other teams to examine the sites for factors contributing to the high scores. Using NIH web sites to identify practices for site design and structure was helpful, because very few teams felt they had counterparts in the private sector. One team member took an opposing view about looking at high-scoring sites to generate change/redesign ideas: "High scores don't mean sites don't have problems."

Teams offered the following suggestions for improving the trans-NIH effort:

- Devote more time to addressing the needs of smaller web sites.
- Try to arrange for an alternate type of licensing agreement for those NIH sites that do not benefit from the yearly license term (or that want to be able to "turn on and off" the ACSI).
- Help teams to identify the sites against which they should be benchmarking.
- Make teams aware that there may be a lag time before site changes can be implemented because of a need to request resources.

4.1.4 Benefits of Experienced/Knowledgeable Web Site Team Members and Teams

An advantage of the phased adoption of the ACSI across web sites was that teams benefited from the experience of members who had been involved with another IC/ODO site's earlier implementation. These individuals functioned as mentors, speeding the pace of learning how to tailor custom questions, use segmentation to address issues, and plan site changes where warranted.

The phased approach also meant that the sites that started after Q4 2004 benefited from the experience of the initial implementers. Although the eight sites that used the ACSI before the trans-NIH project had some early problems, sites that implemented their licenses later reported that start-up activities proceeded relatively more smoothly. As SRAs gained experience working with Federal sites, they were able to extend this guidance to other NIH sites.

4.1.5 Drawbacks of Participating in the Trans-NIH Project

Several teams mentioned that there were drawbacks to participating in the trans-NIH project. The expectation that teams with ACSI licenses would participate in trans-NIH meetings and evaluation activities put additional demands on their time. Several teams missed meetings because they could not easily participate in activities on the NIH campus.

4.2 Identifying and Resolving Common Issues

As some of the teams received feedback and gained insights into site issues, they compared results and approaches with other teams. This section describes issues identified across participating sites that were addressed and/or resolved at the trans-NIH level.

4.2.1 Search Element Scores

At an early trans-NIH meeting, discussion focused on the finding that most NIH sites received relatively lower scores for the Search element. Search Scores typically were displayed in the "Top Priority" quadrant across NIH sites. This quadrant represents the intersection of a relatively lower score and a higher impact number, meaning that the overall Satisfaction Score has the potential for significant improvement if the team improves the way the Search function operates on the web site.

Discussion centered on how NIH sites were implementing Google Search. After the common Search issue was identified and discussed, a subset of teams and members of the Web Authors' Group continued to meet to discuss how they were testing the search capability and adapting the standard capability for use on their sites.

4.2.2 Common Barriers to Improvement Across Sites

As noted in Chapter 3, web sites face financial and staffing constraints when planning to implement changes. A number of teams indicated that they had no specific funding for site changes. One such team described its limited resource situation to the community of ACSI users and, by doing so, formed new contacts and relationships. These new relationships led to what the team described as "free help" from an NIH usability expert and several other resources for identifying key changes. These changes affected how the team managed its site; they now consider the user in deciding how best to present clear information. One team member observed: "You can reduce the number of questions coming in to your staff by putting well organized, useful information out there on the web – information hoarding is no longer acceptable."

4.2.3 Directing Visitors to Appropriate Information Sources

Discussion at several of the trans-NIH meetings focused on an issue that many teams face: visitors looking for information that does not exist on the web site because it is not within the scope of the site. As one team member observed, "It's hard to get people to the right place. People get to a site and should be elsewhere at NIH."

Some teams had mission-related or ethical reasons for not adding the types of content that visitors search for. Some teams decided to add content, based on consultation with their content experts. In other cases where it was possible to identify NIH or other sites that contain the desired content, teams are now linking to those sites; this is done by either adding text to explain where to access the content or making changes to search results pages. These types of solutions could theoretically lead to higher Satisfaction Scores for both first-time and repeat users.

Teams mentioned additional issues that cut across web sites and could be addressed in a trans-NIH forum. These are described below.

- **Limitations on evaluation funding.** Many teams questioned whether funds will be available for additional evaluation activities and for site change and redesign work, given budgets constraints across NIH ICs and ODOs. Although some teams are adding staff to handle data-related site work, many others simply do not have the staff time to review ACSI results. If only limited funds are available, teams may have to prioritize evaluation and redesign activities, delay them, or not conduct them at all. These common problems could be addressed across NIH, by bringing together teams that have the same resource constraints to explore approaches and generate guidelines. For example, it may be possible to pool ideas and resources for a heuristic review of multiple sites or user testing to address issues of specific audiences that cut across sites.

- **Representation of first-time vs. repeat visitors.** Several teams voiced a concern or suspicion that their ACSI results may be overrepresenting the percentage of first-time visitors. These teams were more concerned about meeting the needs of repeat visitors —a segment considered to be the main target audience(s). They think these core audiences may be skipping the survey, and as a consequence their opinions about the site are not reflected in the results.

- **Range of case studies.** Team members of several "small" sites would like to see case studies from other similar-sized sites so that they can communicate about common issues that are more applicable for them. These individuals think that the issues related to large sites have received more attention at the trans-NIH level but that small and niche sites deserve the same level of consideration.

- **Visitor burden and failure to differentiate experiences on other sites.** Several teams expressed the concern that visitors have become increasingly annoyed by the pop-up survey, because many Federal agencies in addition to NIH are using the ACSI. The issue (referred to as the "visitor annoyance factor" by one team) has the potential to lower the response rates across sites. Teams have also voiced the concern that the survey responses may actually reflect perceptions about other sites visited during an online session; visitors may not correctly differentiate which pages belong to a site. Some teams expressed a desire to make their pop-up survey look a little different, as a way to improve response.

4.3 Demonstrating the Contribution of Web Sites to NIH Missions

According to the mission statement on NIH's main web site, one of the ways NIH pursues its main research goals is by providing direction to programs that collect, disseminate, and exchange information about medicine and health. The role of the Internet in promoting information dissemination has increased across NIH as sites expand their reach to more audiences and add more topic areas. The web presence continues to increase in importance to both NIH and its customers as ICs and ODOs move towards accomplishing some or all of their core business functions via the Internet (e.g., grant application processes, clinical trials matching processes, job application processes).

4.3.1 Use of Custom Questions Across Sites

The ACSI methodology provided two options for using custom questions to look at the degree to which NIH web sites meet NIH information dissemination goals: (1) directly –using the <u>same</u> custom question(s) across participating sites and (2) indirectly – through secondary analysis of data collected using <u>similar</u> custom questions across participating sites. The LT and ForeSee Results staff discussed these options in late 2005. Future trans-NIH analysis of custom questions could be used to address questions such as these:

- Who is making use of NIH web sites and why?
- How are NIH web sites helping to advance NIH's mission?
- Is the quality and quantity of content uniform across NIH, or are there significant gaps or redundancies?

Use of a standard custom question. The LT decided to start with one standard custom question—a question with the same stem wording and response options. Results from this trial question would be reviewed and used to decide whether to expand to additional standard questions. The question chosen for the trial was how NIH web site visitors plan to use the information they obtain during a visit. The question wording and options were discussed, reviewed, and then finalized in December 2005; Figure 4-2 shows for the final version of the item. ForeSee added the item to the pop-up surveys across sites in January 2006. Not all sites participated; sites opted out of adding the question to their ACSI survey if it was not applicable to the site's purpose or mission.

How do you plan to use the information you find on this site today?

To share and discuss with my health care provider
To address personal health issues
To aid others who have health concerns
To pursue a career as a medical researcher
To support new or current research projects
To explore or support business opportunities in the field of biomedical research
To explore what you have to offer (just browsing)
Physicians and other health care providers – improved understanding of basic research concepts and findings for my own clinical research
Physicians and other health care providers – improved understanding of clinical research findings for my patient practice
Other

Figure 4-2. Standard custom question added to ACSI Survey in 2006

ForeSee reported results for the standard question at the spring 2006 trans-NIH meeting. Figure 4-3 provides ForeSee's distribution of the means for individual sites' planned information uses. As teams described in detail, the most frequent average response across sites was the "Other" option. Two of the top four planned information uses reflect responses from members of the general public: "To aid others who have health concerns" and "To address personal health issues" (average of 17% and 12%, respectively). In the second position was the response choice "To support new or current research projects"; this response represents a scientific/professional audience. Rounding out the top six positions were the "just browsing" option (9%) and the "To share and discuss with my health care provider" option (8%), which also most likely indicate responses from the general public (or personal rather than business-based uses of the information).

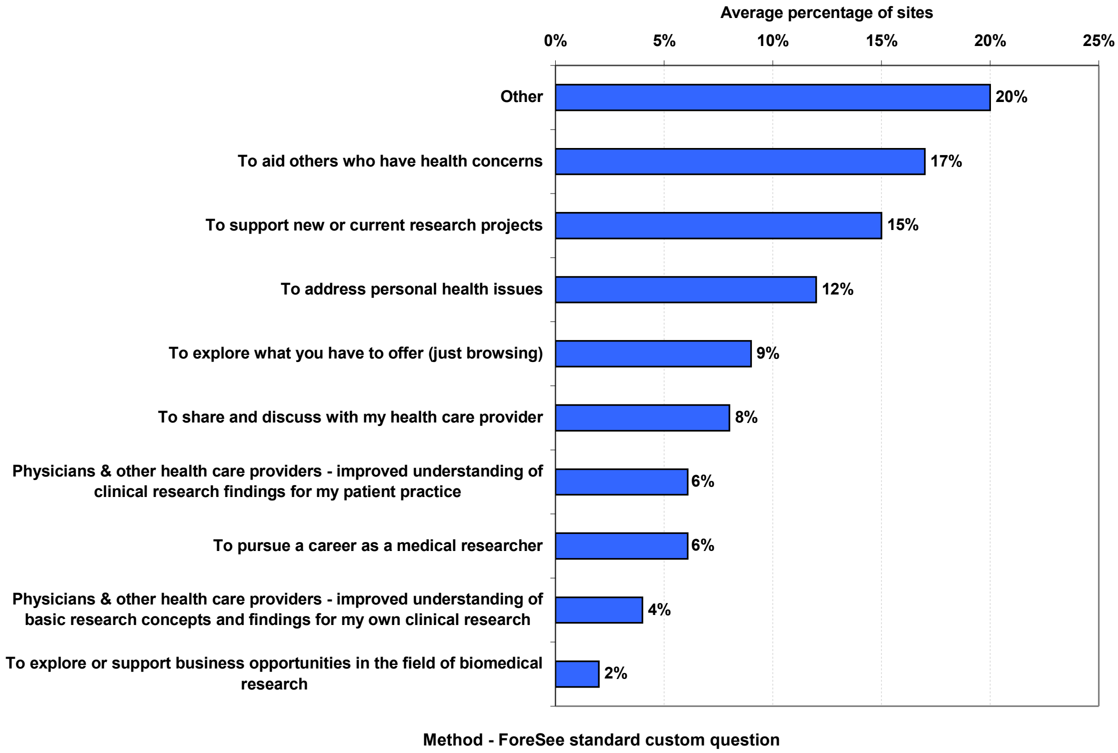

Figure 4-3. ForeSee's standard custom question results: Intended uses of site information

The response options chosen least frequently across sites were those most likely to be chosen by health care practitioners, scientists, researchers, and students. These options, which are shown at the bottom of Figure 4-3, were selected by between 2 percent and 6 percent of visitors.

Taken together, these results indicate that the NIH sites in general are serving a variety of audiences. There is also a heavy emphasis on using information for personal health issues, whether for oneself or for family and friends.

As part of the final interviews (administered in March to April 2006), teams were asked to relate their experiences with the standard custom question. At that time, teams generally did not have enough experience with the standard question to judge the value of using it. Of the 20 teams interviewed, 6 stated that they did not have any experience with the new question. Eight teams indicated that they had collected some responses but did not have enough data to judge the question's value for their site. Six teams reported a negative reaction to adding the standard question. Some of these teams reacted negatively based on their judgment that there were already too many custom questions. The others said

that they had had to rotate out a similar question specifically tailored for their site in order to use the standard version.

Of the teams that had looked at the data, 13 indicated that the "Other" category had received the highest frequency of response. They generally felt that they had lost detail by not being able to collect more in-depth information about visitors' specific plans for using the site information. (These sites had not included a follow-up question to elicit open-ended responses from those who selected the "Other" response option.) Although the standard question provided less specific data, eight of the teams acknowledged that it provided useful data for the trans-NIH level of the evaluation.

Teams were also asked for their ideas about additional questions that might be used across NIH sites in the future. Table 4-1 presents the ideas teams generated. Although approximately half of the teams did not make any suggestions, the suggestions of the other teams overlapped. The suggestions focus on two main areas: (1) visitors' awareness of and perceptions about NIH and (2) site experiences (e.g., whether visitors' information needs are being met at the NIH level).

Table 4-1. Teams' suggestions for additional standard custom questions

**Level of awareness/knowledge about NIH; perceptions of NIH**
Do you know what NIH is?<br>(to gauge visitor's awareness and what he/she learned about NIH from visiting the site)
Did you know what NIH was before coming to this site today?
How do you [the public] perceive the work that NIH does?<br>-- Is NIH making good use of taxpayer dollars (vs. the NIH's mission taken care of by the private sector)<br>-- Would you like to see NIH's funding go up or down?<br>-- Is the percentage of tax dollars that NIH receives for research an appropriate amount?
Would you want to have a portal that links to all NIH sites? (as an alternative to the current structure of many separate IC/ODO sites)
Have you used other NIH sites?
**NIH site experience**
What type of information are you looking for?
Did you find what you were looking for?
How do you prefer to get research/health information?

Secondary analysis of existing custom question data. The indirect approach to addressing how well web sites meet the NIH goals was based on analysis of data collected through the sites' existing custom questions. Custom questions are intended to collect data about a core set of key visitor and site-related variables. In many cases, teams initially drew from a core set of typical questions, then tailored the

question stem and response options to fit the characteristics of their site and expected audiences. Over time, teams also tweaked these questions, either to get more specific information or to correct a shortcoming that became apparent only when they tried to interpret the data (e.g., vague responses to open-ended questions indicated a need to request a referent or more specificity in responses). Therefore, because question stem wording and response options varied across sites, it was not possible to simply merge the data collected by individual sites.

A ForeSee staff member worked with the LT to develop a list of custom questions used commonly across sites, with a count of how many sites were using each one. The LT decided to analyze several of the most frequently used questions that were of primary importance for the trans-NIH level of the evaluation. Despite the slight differences in question wording and response options, the secondary analysis addressed the following site-related variables:

1. Frequency with which the visitor uses the site;
2. The visitor's role (e.g., general public, health care provider);
3. Whether the visitor found the information he/she looked for on the site; and
4. How the visitor heard about the site.

ForeSee conducted the analysis for each of these variables by building a crosswalk between the specific response options offered by the sites and the more general response categories defined to support the secondary analysis. An example of the crosswalk for role of visitor is shown in Table 4-2. The data for individual sites were recoded in this way for analysis at the NIH level.

For the first three of the four variables listed above, ForeSee calculated the mean satisfaction score for the subgroups. The results are summarized below, along with graphs provided by ForeSee.

Frequency With Which the Visitor Uses the Site. As shown in Figure 4-4, across the 41 sites using this question, 54 percent of visitors were first-time visitors; the remaining 46 percent were evenly split between frequent (at least once a week) visitors and infrequent visitors. ForeSee calculated that:

- Sites with more than 50 percent first-time visitors have a mean Satisfaction Score of 75.7 and
- Sites with more than 35 percent frequent visitors have a mean Satisfaction Score of 77.5.

Table 4-2. Example: ForeSee's recoding for trans-NIH analysis of custom questions (visitor's role)

Site's Response Choices	Grouped for Trans-NIH Analysis
General public<br>Citizen<br>General health consumer	General public
K-12 student<br>College student<br>Graduate student	Student
Teacher	Educator
Doctor<br>Nurse<br>Health practitioner	Health care provider
[IC] scientist<br>Outside scientist<br>Researcher<br>Grantee<br>Prospective grantee	Scientist/researcher
[IC] employee<br>NIH staff	NIH staff
Other	Other

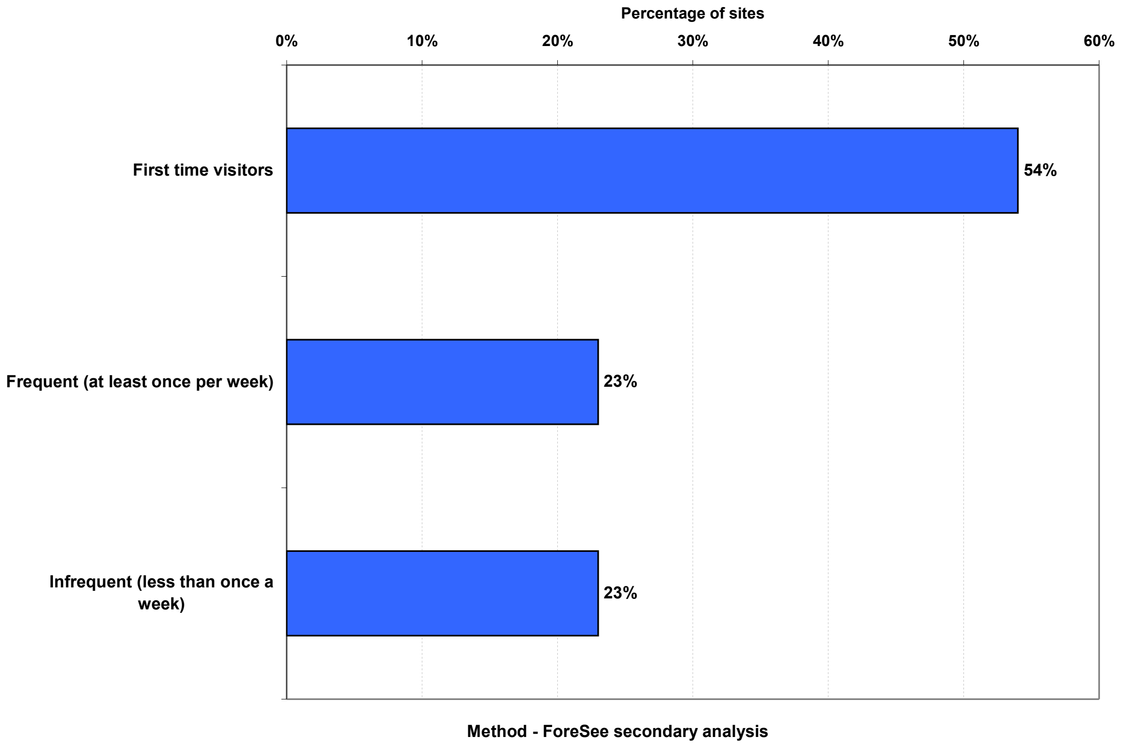

Figure 4-4. Frequency of site visits (across 41 sites)

Role of Site Visitor. Figure 4-5 shows the frequencies for various roles of NIH site visitors, ordered from highest to lowest mean percentage across 42 sites using the role question. Across all sites, students, patients, and healthcare professionals represent the largest percentages of visitors. ForeSee also calculated the satisfaction levels for the largest group visiting each of these 42 sites; these satisfaction results are listed in Table 4-3 below, ordered from highest to lowest satisfaction score (with the number of sites with that largest audience segment):

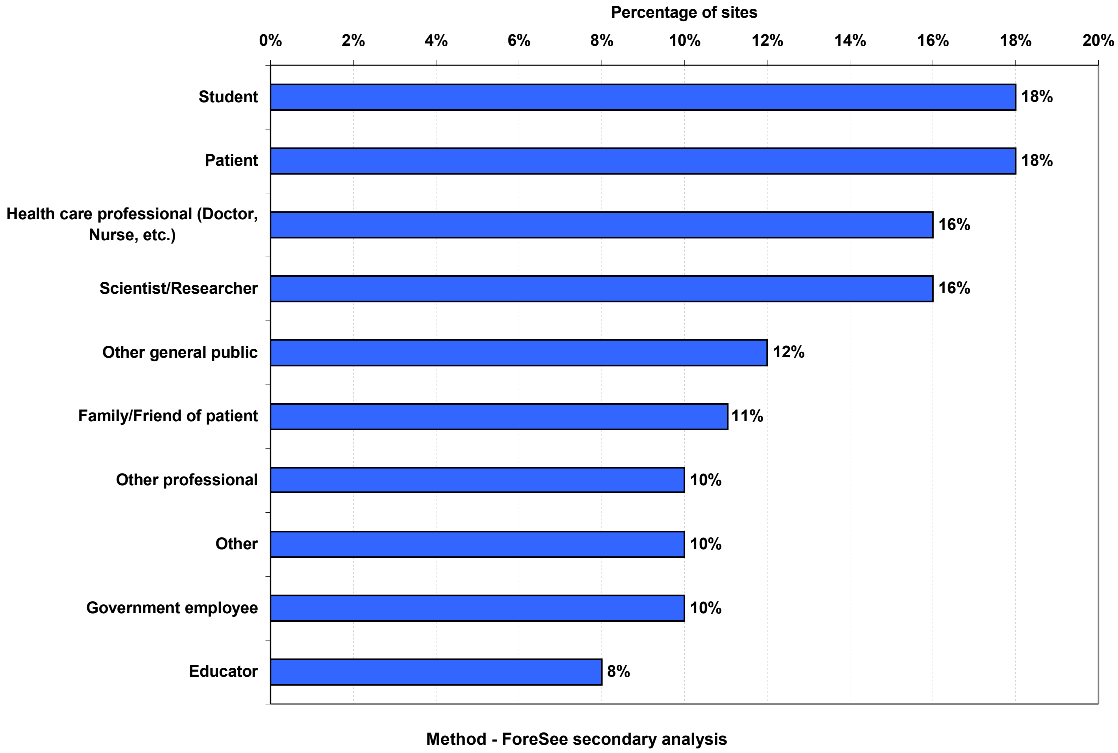

(Note: Percentages do not add up to 100% because of response choice differences between sites asking this question.)

Figure 4-5. Role in which visitor comes to site (across 42 sites)

Table 4-3. Satisfaction scores for most frequently reported visitor roles

Visitor Roles	Overall Satisfaction Score	Number of Sites
Other general public	79.3	4
Patient	79.0	8
Health care professional	76.6	5
Student	75.6	5
Other professional	70.9	5
Scientist/researcher	69.7	10

Whether Visitor Found Information He/She Wanted. Figure 4-6 shows results across 31 sites that used the question "Did you find what you were looking for?" The majority of visitors across sites (63%) responded that they found what they looked for. ForeSee calculated that:

- Sites with more than 80 percent of visitors who responded "Yes" (found what they were looking for) had a mean satisfaction score of 80.0 and
- Sites with fewer than 50 percent of visitors who responded "Yes" (found what they were looking for) had a mean satisfaction score of 71.6.

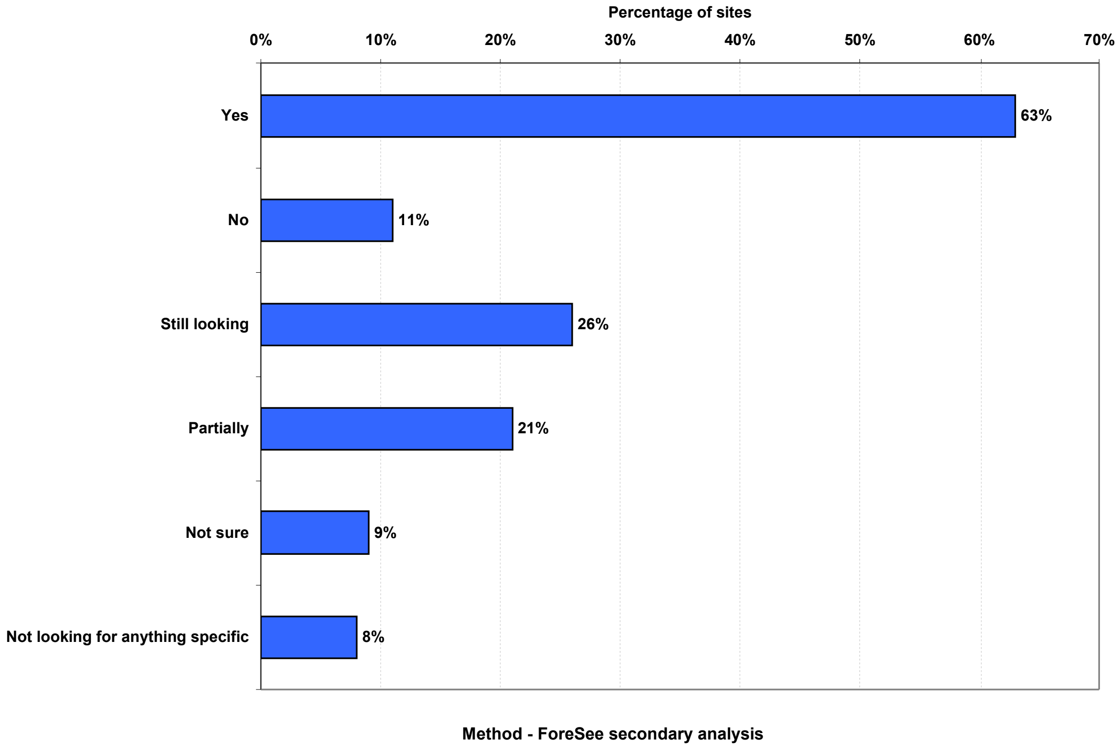

(Note: Percentages do not add up to 100% because of response choice differences between sites asking this question.)

Figure 4-6. Whether site visitors found the information they were looking for (across 42 sites)

How Visitor Heard About Site. Figure 4-7 shows frequencies for the ways visitors were brought to the NIH sites, for the 26 sites that used this question (or its variant, "How did you hear about this site?") The largest percentage (42%) got to NIH sites via a search engine. The next two top ways visitors got to the sites were through links from other (non-NIH) sites and bookmarks.

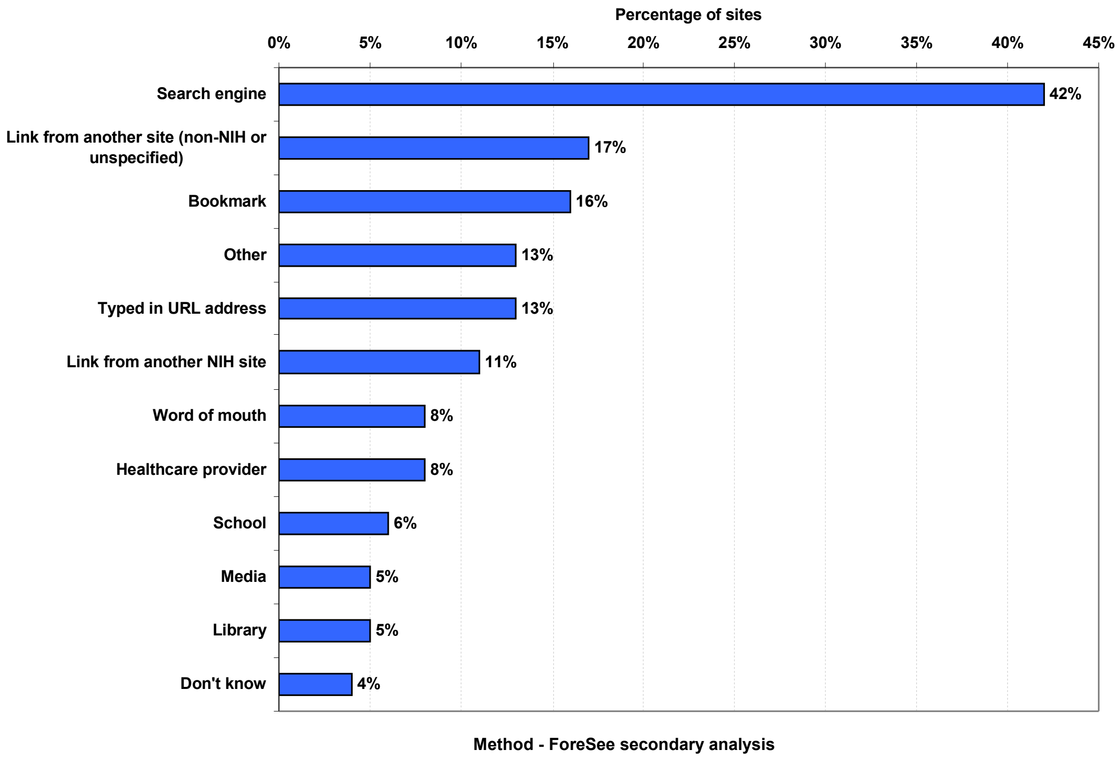

(Note: Percentages do not add up to 100% because of response choice differences between sites asking this question.)

Figure 4-7. How visitors were brought to site (across 26 sites)

Taking the secondary analysis results for all four questions together, these results indicate that NIH sites were serving a very broad variety of audiences with a great diversity of information needs. Overall, the sites are effectively meeting those needs, with some segments (e.g., the general public, those who found the information they looked for, frequent visitors) being more satisfied than others.

4.3.2 Important Message About the Value of Evaluation

The previous section showed how the ACSI customer satisfaction data were used to determine how well the NIH web sites support the overall NIH information dissemination objectives. The high NIH site satisfaction scores reflect well on NIH and indicate that NIH web sites are meeting the information needs of the public. There is an additional benefit gained from the secondary analysis results in that the evaluation data reinforce the importance of evaluation to management of ICs and ODOs as well as to the participating teams. As described below in the section entitled "Benchmarking Across

Agencies," the high Satisfaction Scores reflect well on the NIH and how NIH meets the information needs of the public.

4.4 Trans-NIH Benefits for the Sites

4.4.1 Automatic OMB Clearance

Web sites using the ACSI through the trans-NIH project were able to measure customer satisfaction under the blanket OMB clearance for customer satisfaction research. Thus, they saved both the calendar time and the staff time usually required for submitting an OMB package. Several teams mentioned during the final interview that this was an important factor in their decision to participate.

Given that Federal web sites need to meet an OMB directive to measure customer satisfaction, the free license greatly facilitated NIH sites' ability to meet their measurement goals. Several teams flatly stated that their ICs/ODOs would not have been able to afford a license. The free license provided their only opportunity to pursue customer satisfaction research.

4.4.2 Collection of Site-Specific Qualitative Feedback

As mentioned in Chapter 3, some teams reported that members of their IC/ODO staff (typically in management and/or statistician roles) did not place much stock in the model questions (e.g., "The model questions are not specific enough to our web site"). These teams, in addition to almost all of the others, said that qualitative data collected using the custom questions were invaluable. Teams stated that the custom questions provided more specific customer-based information than the web sites can collect on their own (e.g., through email messages to the site webmaster or through telephone inquiries).

Because each participating site was one of the many NIH sites using the ACSI, it was possible for teams to identify other sites with similar audience characteristics (e.g., percentage of first-time or repeat users, similar core users). Through their participation, teams were able to make comparisons that would not have been possible if they had used the ACSI on their own.

4.4.3 Increased Interest in Customer-Centered Design

The segmentation results and reporting of customer satisfaction by audience segment increased team interest in and focus on customer-centered design. Teams indicated that once they became better educated about their audiences, they were in a better position to serve them. Developing a profile of the audience segments and their satisfaction levels establishes a very important baseline for teams; it serves as the basis against which teams can measure future performance of the site.

4.4.4 Increased Interest in Web Analytics

Based on discussions with teams during the final interviews, one of the benefits of participation in the trans-NIH effort was an increased or renewed interest in conducting site evaluation and collecting customer satisfaction feedback. The use of the ACSI also heightened teams' awareness of the value of quantitative data in web site measurement. In addition, quantitative data provide a sound basis for establishing priorities. Approximately half of the teams used the ACSI in combination with other evaluation tools (as described in Chapter 3).

4.4.5 Other Uses of ACSI Data

Teams were asked during the final interview if they had used the ACSI data in any additional ways (beyond site evaluation). One or more teams mentioned each of the following as additional uses of ACSI data:

- To set priorities for work;
- To obtain assistance/guidance from experts (in site design, site testing, etc.);
- For team bonding (e.g., when scores are high or show improvement);
- To add interesting facts about the site to presentations, reports, etc.;
- To internally promote the important role of the site as a communication tool;
- To suggest scenarios for usability testing;
- To incorporate the data into evaluation of a program as a whole;

- To follow up with those who provide contact information in their responses to open-ended custom questions (which also allows that site to further its mission of helping customers who ask for assistance); and

- To make decisions about content development.

4.5 Impacts Across the U.S. Government

The results of the evaluation demonstrate that the experiences of the NIH web site teams are contributing greatly on a number of levels. This section addresses how the trans-NIH project has provided benefits across the U.S. government.

This evaluation showcases NIH's position in the forefront of Federal customer satisfaction evaluation. NIH's evaluation study can serve as a model of a large-scale evaluation; it includes not just the systematic and simultaneous involvement of multiple sites, but also many types of sites, in using a common customer satisfaction metric. This approach has extended evaluation beyond the level of individual sites to a broad scale, enabling identification of patterns of success. This information will prove increasingly useful in light of the need to establish priorities and make decisions based on budget and staff constraints. ICs/ODOs must deal with how to use funding and staff resources efficiently while also maximizing the use and effectiveness of their web resources.

4.5.1 Expanding Knowledge About Customer Satisfaction Measurement

The NIH experience may benefit other entities within the U.S. Department of Health and Human Services as they strive to meet the customer satisfaction measurement directive and establish best practices in customer-driven management of web sites. Other Federal agencies have steadily adopted ACSI use since September 2003. As of the first quarter of 2006, ForeSee reported that 91 Federal sites were administering the ACSI. Approximately half of these were NIH sites, so NIH accounts for a significant component of the base of knowledge about ACSI use. The increased interest in web analytics shown by NIH web site teams parallels the interest across Federal agencies.

4.5.2 Benchmarking Across Agencies

NIH web sites are significant contributors to the overall government index; the Q1 2006 NIH Customer Satisfaction Index was 75.1, which compares favorably to the e-Government Customer Satisfaction Index of 73.5. Figure 4-8 compares the NIH and e-Government Indexes over the period of the trans-NIH project. For Q1 2006, ForeSee also reported that NIH's mean Future Behavior Scores were comparable to or exceed their counterparts for e-Government and government-wide benchmarks.

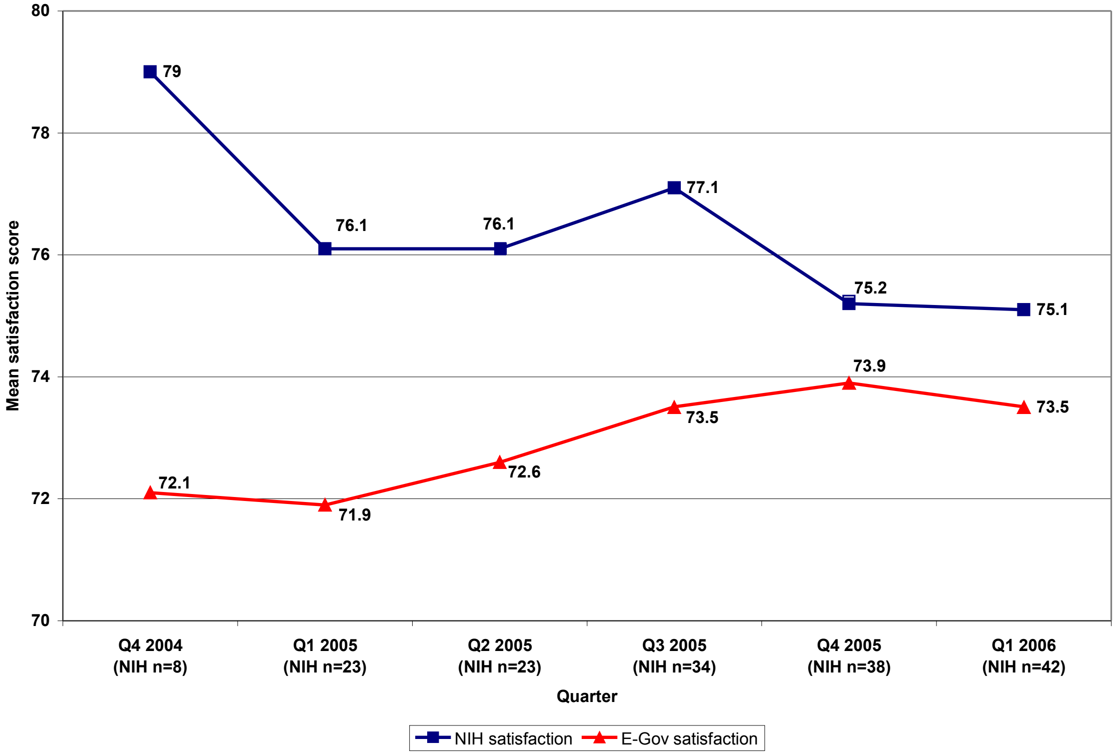

Figure 4-8. Comparison of NIH and e-Government satisfaction indexes during evaluation period

Since the first eight NIH web sites started using the ACSI, ForeSee consistently reported the high ranking of NIH sites among the top-performing Federal sites – scoring 80 or above in overall customer satisfaction. Of the 14 sites cited in this elite group in Q1 2006, 11 were NIH sites.

4.5.3 Best Practices

As a result of the work the NIH web sites have conducted to meet audience information needs and to improve their satisfaction scores, NIH can offer best and promising practices in web design, content delivery, and web-based business processes. The NIH sites that have most successfully used the ACSI can serve as case studies for other Federal web sites. These sites have followed the steps of an improvement cycle to first identify audience segments, then characterize the demographics of those audiences, their patterns of site use, and their satisfaction levels. These sites have identified site issues to be pursued and established priorities based on elements for which improvements are projected to lead to higher Satisfaction Scores. Another evaluation method (typically usability testing and/or heuristic review) is then used to identify specific changes to be made. In some cases, these changes are minimal; in others, they qualify as a full-scale redesign effort. The sites then use the ACSI to track any subsequent changes in customer satisfaction levels, overall and for specific audiences whose needs were addressed through changes.

The NIH evaluation with a variety of site types contributes systematic research results that have been lacking to this point. Identification of site characteristics typically associated with successful use of the ACSI provides guidelines for other Federal agencies faced with making strategic decisions about sites that are likely to benefit most from use of the ACSI. Especially where resources are limited, agencies will be better prepared to establish priorities for use of those resources. Realistic timelines for optimal use of customer satisfaction measurement can also be established if financial resources are not sufficient to cover ongoing and continuous customer satisfaction measurement.

4.5.4 Change in Persistent Cookie Policy

The technology that supports the ACSI survey allows use of a persistent cookie to identify visitors who have already been given the opportunity to complete a survey. The persistent cookie reduces response burden by preventing visitors from being asked to complete a survey multiple times within a specified time period. NIH requested and received special permission to use persistent cookies, representing a departure for Federal web sites. The cookie is used only for logging visit information; it does not collect personal information about site visitors. Individual teams decided whether to use 30-day, 60-day, or 90-day intervals for their persistent cookie, depending on their site use and traffic volume. With the change to a persistent cookie, the sampling rate for many sites was also increased. Visitors may

be presented with the pop-up survey more frequently than the set period if they delete their persistent cookies. In trans-NIH meetings and the in-depth interviews, teams all related that the policy change had a very positive effect in reducing site visitors' frustration at being repeatedly exposed to the survey invitation. This was especially true for sites that have a high percentage of internal NIH users.

5. CONCLUSIONS AND RECOMMENDATIONS

This chapter presents conclusions and recommendations related to both the web site team and trans-NIH levels of the evaluation. These recommendations cover suggested best practices to ensure that teams take full advantage of the capabilities of the ACSI as a site evaluation tool and suggested ways to expand upon the current benefits of the trans-NIH effort.

Trans-NIH-level recommendations are provided in the following areas:

- Continuation of the trans-NIH effort, with the LT leading the effort and with funding provided in part or full by the Evaluation Branch of the Office of Portfolio Analysis and Strategic Initiatives, and
- Alternative (informal) structures for sites continuing to use the ACSI in the event that ICs/ODOs provide partial or full funding for ACSI use.

Site-level recommendations are provided in the following areas:

- NIH web site teams considering whether to use the ACSI and
- NIH web site teams continuing to use the ACSI.

5.1 Conclusions

5.1.1 Trans-NIH Level

Trans-NIH use of the ACSI provided additional value for NIH by expanding and strengthening the evaluation practices of NIH web site teams and highlighting the overall high performance of these sites in meeting the information needs of a wide variety of audiences. Funding and carrying out this program across sites also shows NIH-level support both for the Federal directive to measure customer satisfaction with e-government and for web site evaluation practices.

As a result of the evaluation, NIH has identified the web site types and contexts to which the ACSI is best suited. In addition, teams have provided best practices for its use so that more informed decisions can be made about how to allocate site licenses within ICs and ODOs. The case studies

presented at trans-NIH meetings identified best practices for implementation and use of the ACSI at NIH. Experienced teams learned how to use the custom questions and segmentation to their advantage.

At the trans-NIH level, teams were able to explore solutions to issues that were common across web sites with similar audiences, content, etc. An important example was the special permission granted to NIH sites to use persistent cookies to reduce visitor burden.

Use of the ACSI also allowed NIH to identify how well its web sites meet customer information needs, how NIH is meeting its information dissemination goals, and customer satisfaction with NIH sites among visitors in various roles. Scientists and researchers were generally less satisfied with web sites than members of the general public. Sites with more than 80 percent of visitors who responded that they found the information they looked for on the site had a mean satisfaction score of 80.

5.1.2 Web Site Level

Most of the teams that were able to use the ACSI to collect data and receive feedback were satisfied with and found significant value in the ACSI. The greatest benefits and most positive perceptions of the ASCI's value were reported by teams that synchronized the license term to a stage in their site development/redevelopment cycle where they needed the types of information provided by the ACSI.

Teams that were least satisfied and did not value the ACSI were those that had not received a report, either because of a late license start or a slow ACSI survey collection rate. Some dissatisfaction was also expressed by teams that experienced SRA turnover or did not have an adequate staffing level to attend to the volume of ACSI data.

At the web site level, teams used the ACSI in the following ways:

- As a ready-to-use customer satisfaction metric pre-approved by OMB;
- As a tool to identify a site's strengths and weaknesses;
- As a mechanism for administering and tailoring custom questions as needed to identify and provide qualitative information about specific site issues and problems; and

- As a source of information about critical audiences and site areas to be addressed in any follow-up activities to inform site revisions.

During the interviews, teams indicated the following preferences:

- A short-term ASCI survey with an "on and off switch" that could be adjusted to fit the site's schedule and staff availability;
- A very easy implementation process;
- A means to ask their own questions;
- Experienced ForeSee analysts;
- Evaluation tools available at no cost;
- A cookie policy that reduces respondent burden; and/or
- A way to make their survey look unique to their site rather than similar to those offered on all other NIH sites.

5.2 Trans-NIH Level Recommendations

This section includes a range of suggestions for improving upon the current benefits of trans-NIH use of the ACSI. Observations of trans-NIH meetings, Westat experience, and data collected through qualitative interviews are the basis for these recommendations.

5.2.1 Trans-NIH Continuation

- If the LT receives additional set-aside funding and decides to continue the trans-NIH effort, consider adding one or several more standard custom questions to assess the degree to which NIH is meeting its communication objectives. This is important so that NIH can identify strengths (what sites are doing well) and weaknesses (underserved audiences and unmet information needs). Critical questions about visitors and information needs can be prioritized based on a review of the NIH mission statement.
- Provide a stable benchmark for the web sites that commit to continuous use of the ACSI. Designate a set of sites that will form the index of NIH customer satisfaction, to provide a more suitable indicator of NIH performance over time. The index will be less susceptible to changes that merely reflect the number of sites – and which sites – are included each quarter.

- Explore the possibility of forming a subgroup of teams that have a shared interest in benchmarking. The subgroup could pursue several issues related to internal NIH benchmarking and benchmarking in other contexts. The subgroup could first set criteria (such as web site processes, profiles of audience types or primary audience, site structure, traffic volume level, types of transactions provided by the web site) for identifying "similar" sites. Then, the subgroup could apply these criteria to identify comparison sites from among private sector sites, other NIH sites, and other government sites.

- Consider benchmarking on the basis of how NIH sites are using the ACSI, as suggested by several teams. Teams measuring satisfaction with the whole site could compare their sites to others using the ACSI in the same way. Teams measuring satisfaction at the level of a specific site section could make comparisons to other sites with that focus.

The LT or another group providing guidance could also judge the value of new products that ForeSee plans to offer (e.g., a daily report package, a page-level satisfaction measure, a comment card with a satisfaction measure). This evaluation could easily be done on a small scale, by selecting one or more sites willing to serve as test cases. The selection of test sites from among interested sites could be based on identifying which site type(s) is most likely to benefit from the services and can provide the necessary funding, then designating one (or more) as the test site(s). Recommendations based on those experiences could be provided to other sites that may benefit from the new products as well.

Web sites teams that have similar cross-cutting issues, such as how best to present grant opportunity information to researchers, could jointly address those issues. These teams could review their results and open-ended question responses to identify cross-cutting audience issues. To ease the restrictions on funding available for web site evaluation, teams could be encouraged to form partnerships to pool and stretch those limited resources. Results would provide benefits for more sites at the same time and reduce the need for all sites to do their own follow-up steps. Alternatives for addressing the identified issues could be subjected to usability testing; the teams could all learn from the results and apply those findings and principles to make site changes.

Another important trans-NIH issue is the concern of some web site teams that their ACSI data will be affected if more Federal sites use the ACSI. Some teams expressed a suspicion that their survey responses may represent visitors' broader experience across many sites rather than their opinions about the site on which they were presented with the pop-up survey. One or several sites could take the lead to investigate this hypothesis. The site(s) could provide an instructional message stating that the visitor can minimize the pop-up survey until he/she has used the site enough to feel comfortable responding. The team could investigate the effects of this message by comparing the response rate and

data quality (e.g., for open-ended responses) before and after implementing this technique. These results could then be shared and used to determine whether to extend the approach to other sites.

Information about and access to any central resources might be made available, where possible, to web site teams with very few or no resources to plan or act on their ACSI results. The LT can contribute to building the web evaluation community by disseminating information about and encouraging participation in any forums or meetings of Federal web groups. These venues provide teams with valuable insights from others in similar circumstances and facilitate potentially beneficial contacts.

5.2.2 Alternatives to the Leadership Team Role in Guiding Teams Using the ACSI

There is some uncertainty about what will happen after the funding provided by set-aside funds terminates for most web sites at the end of June 2006. If no central source of evaluation funding carries these sites forward, there will also be some uncertainty about the continuing role of the LT. In that situation, several alternative structures could provide support for teams continuing use of the ACSI.

If the ICs and ODOs assume the costs of continuing licenses, they might also take the role of encouraging the sites to take full advantage of the ACSI. Several ICs/ODOs have multiple sites using the ACSI; these ICs/ODOs could provide some centralized support within their own IC/ODO and also to ICs/ODOs that have only one site using the ACSI.

A second option is to form an NIH ACSI Users Group, possibly as a subgroup of the NIH Web Authors Group, to take on the role of fostering communications among teams. The group's responsibilities could extend to the collaboration activities currently performed by the LT and also cover liaison activities with ForeSee.

A third alternative is to designate an NIH staff member to serve as the full-time NIH ACSI program director and liaison with ForeSee. This staff member could perform a variety of activities to further ACSI use at NIH, including assisting/mentoring new sites during start-up, providing help for major activities such as segmentation (by providing several examples) and action planning, providing access to additional resources (e.g., evaluation methods assistance), monitoring the status of participating sites, and monitoring trans-NIH evaluation plans and results. A funding source to cover such a position would have to be addressed.

5.3 Web Site-Level Recommendations

Data collected through qualitative interviews and meeting observations provide the basis for these web site-level recommendations. Many of these recommendations involve identifying sites that might benefit from using the ACSI; these approaches may also help to identify criteria for selecting sites to continue their ACSI licenses if funding levels are not sufficient to cover all interested sites.

5.3.1 Web Sites Considering ACSI Use

Patterns of ACSI use by currently participating web sites should be examined to identify additional NIH sites that may benefit from using the ACSI and which sites might face some challenges in implementing and using it. The following points are offered as a checklist for ICs/ODOs and web site teams considering whether the context is favorable for a license.

"Yes" responses to all of the following questions indicate that a site is prepared to take on an online survey and that it fits a profile for likely success. However, if some of the following questions are answered "No," a team may still opt to proceed with a site license, find value in the ACSI, and achieve some degree of success using it. Even sites that have fewer FTEs for site support or lower traffic volume have shown increases in Satisfaction Scores over time. Knowing that other NIH sites have addressed a problem may help a team to change its approach to minimize the potential occurrence of a similar issue.

1. Does the web site have resources to devote to implementing and using the ACSI?
   - Do the staff members who will be required to interact with ForeSee have time to review data, learn how to use the custom questions and segmentation to delve into the data, and plan follow-up steps?
   - Does the IC/ODO have funding (or alternative funding sources) for making changes that the team identifies as critical for improving customer satisfaction?

2. Are the IC/ODO management and the web site team ready and willing to begin and follow through on making site changes?

- Does the team have a high level of interest in taking on evaluation and customer satisfaction work?
- Are the management and web site team willing to work within the basic ACSI timeline of 1 to 2 years? Are they aware of and willing to accommodate the factors that can stretch the timeline?
- Does the management structure support and facilitate an interest in evaluation?
- Is the team preparing for a site redesign or re-launch?

3. To what degree does the site fit the ACSI model and method assumptions?

- Does the site have the level of traffic volume to complete an ACSI feedback cycle within a "reasonable" time?
  - The monthly visitor traffic should ideally provide the first 300 ACSI responses within 1 to 2 months.
  - Teams wishing to use the ACSI as an expedited method for obtaining customer feedback (using custom questions but not the full model) should decide whether the ACSI is a cost-effective method in comparison to other options (e.g., an outside contractor or internal resources to conduct a one-time survey).
- Is the site a public site? (Use of the ACSI for intranet sites is strongly discouraged because of their typical site traffic volume and a high percentage of repeat visitors, who may develop "survey fatigue.")
- Is the site structured in a way that allows for easy adoption of code? (If not, is the team willing to spend the time to do that step manually?)
- Does the length of any previous design/redesign cycle(s) fit with the plan for the ACSI license term?

For teams that decide to use the ACSI, early assistance with ACSI implementation activities will work to the team's advantage. The experiences of teams using the ACSI, especially those with the longest running licenses, provide the basis for documenting best practices. A best practices guide or set of FAQs should cover the critical steps of implementation, data interpretation, custom question fine-tuning, segmentation and in-depth analysis, and action planning. The guide should explicitly describe the team's role and provide the following information.

- **Custom question guidance.** ForeSee recommends modifying some custom questions and adding other question types as a team learns about its audience base and visit characteristics. A best practices guide should provide some general guidelines about

the kinds of custom questions to be used and when, and how to tailor them to collect meaningful data on identified issues. Case studies of different NIH sites could also be included to provide realistic detail and meaning to the guidelines.

- **Segmentation guidance.** Through the SRAs, ForeSee recommends types of segmentation analyses expected to address issues or further quantify and explain results. Teams new to the ACSI would benefit from NIH site examples and the types of segmentations found to be most useful for different types of site issues.

- **Realistic expectations for the role of ForeSee analysts.** Some teams expected SRAs to identify not only high priorities for revision but also specific site revisions. Because this is a high need area but outside the SRAs' purview, teams could share their experiences making changes that result in more favorable feedback.

- **Realistic expectations for the timetable.** Based on experiences of teams that have the most experience, a range of number of months could be provided for each of the key activities (e.g., X months to conduct in-depth analysis of the data; Y months to follow up findings with other techniques such as expert review and usability testing with key groups; Z months for planning). Additional time requirements could be noted for contexts where stakeholders express reluctance about the method or where extra time is needed to request funding for site revisions or a redesign.

- **Acting on ACSI results.** Teams that had experience with user-centered design methods and/or a relationship with usability staff/experts seemed comfortable using their ACSI data to plan next steps. However, other teams indicated a strong need for assistance in translating results into actions. Case studies would help new teams learn how to generate ideas for site changes and incorporate user-centered design concepts. In addition, the guide could provide links to NIH resources such as methods advice/assistance to help in planning improvements.

- **Advantages of access to a "mentor".** ICs and ODOs with multiple participating sites have benefited greatly from the experience of a staff member who worked with the ACSI at an earlier time. A more formal basis for assigning mentors to new sites would be fruitful; mentors would especially need to help teams integrate ACSI results with any data from other sources and address how to make site revisions. If new sites will not have access to an internal NIH mentor, perhaps an experienced SRA could be requested from ForeSee. Several teams have identified specific SRAs who have a proven track record for efficiently getting teams through the implementation, segmentation, and interpretation stages.

- **Importance of using a mix of evaluation methods.** Teams need to be made aware that the ACSI should be one, but not the only, method used for web site evaluation. To ensure comprehensive site evaluation, teams should also consider combining and sequencing customer satisfaction measurement with other methods such as usability testing, heuristic review, web log review, analysis of public inquiries, and search log analysis.

5.3.2 Web Sites Continuing to Use the ACSI

Web site teams renewing their licenses should review the role of the ACSI as a web site evaluation tool and set realistic objectives for their next term of use. The current stage of their web site within the general "evaluation – revision or redesign – development" life cycle should be considered when setting these objectives. Teams should also review the following points about the ACSI.

- The ACSI is meant to be one tool – rather than the only tool – in the "evaluation toolbox." ForeSee recommends using the ACSI as a method to better focus the application of additional site evaluation methods that typically follow the diagnosis of site problems/issues.

- The ACSI provides a measure of the degree to which the site is connecting with visitor audiences. Satisfaction measures provide a different view of web site performance than (and a complement to) the site usage reports that most teams review to monitor how their sites are used on a monthly and yearly basis.

- Teams are most likely to capitalize on the strengths of the ACSI when the license period coincides with a significant level of effort to revise/update the site or launch a redesign.

- Teams' experiences indicate that ACSI results may not directly suggest what changes are required to address audience needs and improve satisfaction levels. The ACSI may not be as useful as other methods for specifying exactly how to make a change.

- A best practice for using the ACSI in web site management is to evaluate both the need for and success of design changes, which requires establishing a baseline for comparison and then monitoring against it after making changes.

- The ACSI pinpoints priorities for improvement in overall satisfaction as the basis for allocating resources.

In general, a recommended course for teams needing information is to seek out members of other web site teams. (As described above, a formal or informal mentoring process could help to designate contacts for assistance.) For example, a team may need guidance on how to allocate resources and take appropriate steps when the ForeSee priority map indicates that navigation is a top priority. Learning what other teams have done in similar circumstances would facilitate the process of generating ideas and devising practical solutions.

APPENDIX A

SITE TRACKING TABLE

APPENDIX A. SITE TRACKING TABLE

IC or OD Abbreviation	Site Name	URL	Included in Interim Report	PI Work-sheet	Site Look-up	Initial Survey	Initial Inter-view	Final Survey	Final Inter-view
CIT	iSDP*	http://isdp.cit.nih.gov	Yes	Yes	Yes	Yes		Yes	Yes*
NLM	AIDSinfo	aidsinfo.nih.gov	Yes		Yes	Yes	Yes	Yes	Yes
NHLBI	Aim for a Healthy Weight	http://www.nhlbi.nih.gov/health/public/heart/obesity/lose_wt/index.htm	Yes	Yes	Yes	Yes		Yes	Yes
CIT	Cit.nih.gov	http://CIT.nih.gov	Yes	Yes	Yes	Yes		Yes	Yes*
NCI	Clinical Trials Search Form – Results	http://www.cancer.gov/clinicaltrials	Yes	Yes	Yes	Yes		Yes	
NCI	Clinical Trials Search Form – No Results	http://www.cancer.gov/clinicaltrials			Yes	Yes		Yes	
OD/OIR CME	CME (1 site)	www.cme.nih.gov	Yes	Yes	Yes			Yes	Yes*
OD/ORD	Office of Rare Diseases Home Page	http://rarediseases.info.nih.gov/.	Yes	Yes	Yes	Yes			
NHLBI	Disease & Conditions Index	http://www.nhlbi.nih.gov/health/dci/	Yes	Yes	Yes	Yes		Yes	
FIC	Fogarty International Center	www.fic.nih.gov	Yes	Yes	Yes	Yes		Yes	
OD/OER	Funding Opportunities (The NIH Guide for Grants and Contracts)	http://grants1.nih.gov/	Yes	Yes	Yes	Yes		Yes	Yes
NHGRI	Genome.gov	www.genome.gov	Yes	Yes	Yes			Yes	Yes
OD/OHR	Jobs @ NIH	www.jobs.nih.gov	Yes	Yes	Yes	Yes	Yes	Yes	Yes
NLM	MedLinePlus	www.medlineplus.gov	Yes	Yes	Yes	Yes	Yes	Yes	Yes
NLM	MedLinePlus en Espanol	medlineplus.gov/spanish	Yes	Yes	Yes	Yes	Yes	Yes	Yes
NCCAM	National Center for Complementary and Alternative Medicine Web Site	www.nccam.nih.gov	Yes		Yes	Yes		Yes	
NIA	National Institute of Aging	nia.nih.gov	Yes	Yes	Yes	Yes		Yes	Yes
NIGMS	National Institute of General Medical Sciences – Laying the foundation for disease diagnosis, treatment, and prevention	http://www.nigms.nih.gov	Yes	Yes	Yes	Yes	Yes	Yes	Yes
**IC or OD**	**Site Name**	**URL**	**Included**	**PI**	**Site**	**Initial**	**Initial**	**Final**	**Final**

Abbreviation			in Interim Report	Work-sheet	Look-up	Survey	Inter-view	Survey	Inter-view
NCI	NCI Web Site	http://www.cancer.gov	Yes		Yes	Yes	Yes	Yes	Yes
NEI	NEI Web Site	www.nei.nih.gov	Yes	Yes	Yes	Yes		Yes	
NHLBI	NHLBI Public Site	http:/www.nhlbi.nih.gov	Yes	Yes	Yes	Yes		Yes	
NIAID	NIAID Web Site	www.niaid.nih.gov	Yes		Yes	Yes	Yes	Yes	Yes
NIAMS	NIAMS Public Web Site	http://www.niams.nih.gov/index.htm	Yes	Yes	Yes	Yes		Yes	
NIDCD	NIDCD Web Site	www.nidcd.nih.gov	Yes	Yes	Yes	Yes		Yes	
NIDCR	NIDCR Web Site	www.nidcr.nih.gov	Yes	Yes	Yes	Yes		Yes	
NIDDK	NIDDK Main Site	http://www.niddk.nih.gov	Yes	Yes	Yes	Yes		Yes	
OD/OSP/OSE	NIH Curriculum Supplements	http://science.education.nih.gov/supplements	Yes	Yes	Yes	Yes		Yes	Yes*
OD/OCPL	NIH Health Information	health.nih.gov	Yes	Yes	Yes	Yes		Yes	
OD/OCPL	NIH Home Page	www.nih.gov	Yes	Yes	Yes	Yes		Yes	Yes
OD/OTT	NIH Office of Technology Transfer	http://ott.od.nih.gov	Yes	Yes	Yes	Yes		Yes	
NIDCD	NIH Stem Cell Information	http://stemcells.nih.gov	Yes	Yes	Yes	Yes		Yes	Yes
NLM	NIHSeniorHealth	http://nihseniorhealth.gov	Yes		Yes	Yes		Yes	
NIMH	NIMH Web Site	www.nimh.nih.gov	Yes	Yes	Yes	Yes		Yes	Yes*
NLM	NLM Main	www.nlm.nih.gov	Yes	Yes	Yes	Yes	Yes	Yes	Yes
OD/OACU	OACU Web Site	http://oacu.od.nih.gov/	Yes	Yes	Yes	Yes		Yes	
OD/ODS	Office of Dietary Supplements	ods.od.nih.gov	Yes	Yes	Yes	Yes			
NCI	Office of Liaison Activities, NCI Listens and Learns	http://ncilistens.cancer.gov	Yes	Yes	Yes	Yes		Yes	
OD/ORS	Office of Research Services – Main Page	http://www.ors.od.nih.gov/	Yes	Yes	Yes	Yes		Yes	
OD/ORF	ORF Internet	http://orf.od.nih.gov	Yes		Yes	Yes		Yes	
OD/OSP/OSE	OSE Web Site	http://science.education.nih.gov	Yes	Yes	Yes	Yes	Yes	Yes	Yes
NLM	PubMed	www.ncbi.nlm.nih.gov/entrez/query.fcgi	Yes		Yes	Yes	Yes	Yes	Yes
NCI	Surveillance, Epidemiology, and End Results (SEER)	http://seer.cancer.gov/	Yes	Yes	Yes	Yes	Yes	Yes	Yes
NIDA	teens.drugabuse.gov	teens.drugabuse.gov	Yes	Yes	Yes	Yes	Yes	Yes	Yes
NLM	TOXNET	toxnet.nlm.nih.gov	Yes		Yes	Yes	Yes	Yes	Yes

Trans-NIH ACSI Evaluation:
Tracking May 26, 2006

IC or OD Abbrev	Site Name	URL	Included in Interim Report	PI Work-sheet	Site Look-up	Initial Survey	Initial Inter-view	Final Survey	Final Inter-view
NIDA	www.drugabuse.gov	www.drugabuse.gov	Yes	Yes	Yes	Yes	Yes	Yes	Yes
NIEHS	NIEHS Home Page	http://www.niehs.nih.gov/	Yes	Yes	Yes	Yes		Yes	
NCI	Division of Cancer Control & Population Sciences	http://dccps.nci.nih.gov	Yes		Yes	Yes		Yes	
OD/OERRM	Electronic Research Association	http://era.nih.gov/			Yes			Yes	
OD/OSPP	GPRA Site	http://nihperformance.nih.gov			Yes	Yes			
**Intranet Sites**									
NHLBI	NHLBI Intranet	http://insider.nhlbi.nih.gov	Yes	Yes	Yes	Yes		Yes	
NIDCD	NIDCD Intranet	http://intranet.nidcd.nih.gov/	Yes	Yes	Yes	Yes		Yes	
CIT	NIH Portal	my.nih.gov	Yes		Yes	Yes		Yes	
OD/OER	Grants Intranet	http://odoerdb2-1.od.nih.gov/gmac/home.html		Yes	Yes				
	(+ DIR Intranet)	*(see below – Deactivated)*	--	--	--	--			
	(+ Extramural Financial Data Branch)	*(see below – Deactivated))*	--	--	--	--			
**Deactivated Sites**								**Reason**	
CIT	antivirus.nih.gov	http://antivirus.nih.gov	Yes	Yes	Yes	Yes		Rolled up license to one CIT license; surrendered others	
CIT	CIT Data Center	http:datacenter.cit.nih.gov	Yes	Yes	Yes	Yes			
CIT	CIT Security Page	http://cit.nih.gov/security.html	Yes	Yes	Yes	Yes			
CIT	Networking and Telecommunications	http://cit.nih.gov/nw-tc.html	Yes	Yes	Yes	Yes			
NHLBI	DIR Intranet	http://dir-intranet.nhlbi.nih.gov/	Yes	Yes	Yes	Yes		Assigned to Roll-up report	
NCI	Extramural Financial Data Branch (Intranet)	http://camp.nci.nih.gov/admin/oem/efdb/	Yes	Yes	Yes	Yes		Re-assigned within IC	
Recently Deactivated									
OD/ORD	CHID Homepage	http://chid.nih.gov	Yes	Yes	Yes	Yes			Site ending
NHLBI	NHLBI DIR Internet	http://dir.nhlbi.nih.gov/	Yes	Yes	Yes	Yes		Yes	Low ACSI response
		**Totals**	**57**	**48**	**61**	**57**	**14**	**51**	**20 (+5*)**

*Five short interviews were conducted with teams representing sites that started license late in 2005 or collected completed surveys at a relatively slow rate. These interviews were conducted with an abbreviated protocol.

Trans-NIH ACSI Evaluation:
Tracking May 26, 2006

IC or OD Abbreviation	Site Name	URL	Included in Interim Report	PI Work-sheet	Site Look-up	Initial Survey	Initial Inter-view	Final Survey	Final Inter-view
**Never Activated Sites**									
CC	NIH Clinical Center	Clinicalcenter.nih.gov						Site decided not to use code	
NEI	NEI Clinical Studies Database	www.nei.nih.gov/neitrials/index.aspx						Site decided not to use code	
OD/OER	OER Extramural Intranet	http://odoerdb2-1.od.nih.gov/oer/oer.htm						Re-assigned within IC	
OD/OER	Funding Opportunities (The NIH Guide for Grants and Contracts)	http://grants1.nih.gov/grants/guide/index.html						Folded into grants.nih.gov site	
NCI	Trial Check Search Form							Replaced by NCI Clinical Trials Search – No results	

APPENDIX B

SURVEY AND INTERVIEW INSTRUMENTS

Initial Survey for All Participating Sites
Final Survey for All Participating Sites
Initial Interview for Selected Sites
Final Interview for Selected Sites

INITIAL SURVEY FOR PARTICIPANTS IN THE NIH

American Customer Satisfaction Index (ACSI) Evaluation

Instructions:

If you are completing an *electronic copy* of this survey:

- o Click on a shaded area to type an X or a text response. The text boxes will expand to the size you need.

If you are completing a *hard copy* of this survey:

- o Mark an X in a box to indicate your response.
- o Write your text into the boxes; continue on the back of the page if needed.

Section A. Website Background

The following items are designed to help us learn some basic background about the [PREFILL WEBSITE URL] website hosted by your agency. Click on the box to mark you answer electronically, or place an X in the box if you are completing this in hardcopy form.

1. This site is a(n):

☐Intranet site
☐Public site

2. Is use of your site restricted by password to a particular audience?

☐Yes [GO TO 2a]
☐No [GO TO 3]

2a. Which audience?

☐Registered users
☐NIH employees
☐Other (*specify*):

3. **Now we'd like to learn about your *current* site visitors.**

3a. What percentage of your site visitors visit your site only once?

_____Percent

☐Don't know

3b. What percentage of your site visitors are repeat visitors?

_____Percent

☐Don't know

3c. What is the source of your information about site visitors?

☐We don't collect

☐Quantitative data (*specify*):

☐Qualitative data (*specify*):

☐ACSI results

☐Other (*specify*):

4. **Approximately how many years has your site been up?**

☐Not yet launched; still in development
☐Less than 1 year
☐1 – 2 years
☐More than 2 years but less than 5 years
☐5 – 10 years
☐More than 10 years
☐Don't know

5. **When was the *current version* of your site launched?**
_____ (month)_____ (year)

☐Don't know

6. Which of the following user group(s) is your website *intended for?* *(Mark all that apply)*

☐General public
☐Patient
☐Family member, friend, or coworker of patient
☐Healthcare provider (e.g., nurse, physician, etc.)
☐Scientist/Researcher
☐Healthcare Administrator
☐Librarian or Information Professional
☐Journalist/Reporter
☐Student
☐Educator
☐Advocacy group member
☐NIH Institute/Center/Office Staff
☐Other *(specify)*: ____________
☐Don't know

7. Which of the following user groups are *actually using* your website? *(Mark all that apply)*

☐General public
☐Patient
☐Family member, friend, or coworker of patient
☐Healthcare provider (e.g., nurse, physician, etc.)
☐Scientist/Researcher
☐Healthcare Administrator
☐Librarian or Information Professional
☐Journalist/Reporter
☐Student
☐Educator
☐Advocacy group member
☐NIH Institute/Center/Office Staff
☐Other *(specify)*: ____________

☐Don't know [GO TO 8]

7a. What is the source of your information about site users? (*Please specify*):

☐We don't collect this information

☐Qualitative data (*specify*):

☐Quantitative data (*specify*):

☐ACSI results

☐Other (*specify*):

8. Please briefly describe how the website fits with your IC's or Office's mission.

8a. If your site is primarily used for information dissemination purposes, describe briefly how the website fits into your agency's overall communication strategy.

9. How does your IC or Office *primarily* promote the website to target audiences?

10. Are there other ways that your IC or Office promotes the website?

☐ Yes [GO TO 10a]
☐ No [GO TO 11]
☐ Don't know

10a. Please list the other ways that your IC or Office promotes the website.

11. Beyond your promotion of the website, are there other ways that people learn about your website?

☐Yes [GO TO 11a]
☐No [GO TO 12]
☐Don't know

11a. Briefly describe these other ways that people learn about your website.

12. Do other websites serve similar purposes to your website?

☐Yes [GO TO 12a]
☐No [GO TO SECTION B]
☐Don't know

12a. List up to three names of those other websites. Please include NIH and non-NIH websites.

Non-NIH websites:
1. ___________________
2. ___________________
3. ___________________

Other NIH websites:
1. ___________________
2. ___________________

3. ____________________

Section B. Management of Site Before ACSI

The next few items will help us understand how you monitored and managed your website(s) prior to implementation of the ACSI.

1. **Please tell us about the *kinds* of evaluation methods you used to collect information about your website(s) prior to using the ACSI.**

☐None[GO TO 4]

☐Expert or heuristic review
☐Usability testing
☐Focus group
☐Survey
☐Web log software (e.g. Web Trends)
☐Analysis of incoming emails
☐Analysis of incoming phone calls
☐Other (*specify*):

1a. Have you customized a measurement tool or approach for evaluating your website?

☐YES [GO TO 1b]
☐NO [GO TO 2]

1b. Please describe that tool/approach.

2. How did you use the data you collected?

3. Prior to the ACSI, what other types of information did your team feel it needed to manage the website?

[IF YOU ANSWERED 3, SKIP 4 AND GO TO 5]

4. Was there information that your team felt it needed to manage the website?

❑YES [GO TO 4a]
❑NO [GO TO 5]

4a. Please describe those types of information.

5. The following table is designed to help us learn about changes made to your site *prior to* implementing the ACSI.

Mark all changes that apply.

Include changes made since your last major redesign (see Section A, Item #5).

Do not include <u>routine updates</u> you do to keep content current.

For each "Yes" answer, indicate the degree and approximate date of change during the time your team has managed the site.

❑**Check here if your site has not had a redesign [GO TO SECTION C]**

Type of Change	Were any changes made? (Y/N)	Degree of change (Low/Moderate/High)	Approximate date of change (MM/YYYY)
5a. Addition of new topic areas or content types	☐Yes<br>☐No	☐Low<br>☐Moderate<br>☐High	Date(s) __/____<br><br>☐Don't know
5b. Functionality<br>(e.g., services provided, convenience of the services, visitors' ability to accomplish what they wanted)	☐Yes<br>☐No	☐Low<br>☐Moderate<br>☐High	Date(s) __/____<br><br>☐Don't know
5c. Overall look and feel	☐Yes<br>☐No	☐Low<br>☐Moderate<br>☐High	Date(s) __/____<br><br>☐Don't know
5d. Homepage redesign	☐Yes<br>☐No	☐Low<br>☐Moderate<br>☐High	Date(s) __/____<br><br>☐Don't know
5e. Subpage redesign	☐Yes<br>☐No	☐Low<br>☐Moderate<br>☐High	Date(s) __/____<br><br>☐Don't know
5f. Navigation	☐Yes<br>☐No	☐Low<br>☐Moderate<br>☐High	Date(s) __/____<br><br>☐Don't know
5g. Search	☐Yes<br>☐No	☐Low<br>☐Moderate<br>☐High	Date(s) __/____<br><br>☐Don't know
5h. Site Performance<br>(e.g., speed of loading the page, reliability of site performance, consistency of speed)	☐Yes<br>☐No	☐Low<br>☐Moderate<br>☐High	Date(s) __/____<br><br>☐Don't know
5i. Other (specify):	☐Yes<br>☐No	☐Low<br>☐Moderate<br>☐High	Date(s) __/____<br><br>☐Don't know

Section C. Joining the Trans-NIH ACSI Evaluation

The next items address your IC's or Office's reasons for participating in the trans-NIH ACSI website evaluation.

1. **Which of the following reasons for participating in the trans-NIH ACSI website evaluation apply to your site? (*Mark all that apply*)**

- ☐ Increase levels of customer satisfaction
- ☐ Use the benchmarking feature of the ACSI
- ☐ Gain experience in web evaluation methods
- ☐ Identify strength(s) of the site by examining ACSI component-level scores
- ☐ Identify weakness(es) of site by examining ACSI component-level scores
- ☐ Meet an IC directive to evaluate the site
- ☐ Take the opportunity to receive ACSI evaluation services for free
- ☐ Take the opportunity to incorporate custom questions into the ACSI
- ☐ Take the opportunity to participate in a trans-NIH collaborative effort to share web evaluation learning and experience
- ☐ Other (*specify*):
- ☐ Don't know

2. **How confident is your team that you will be able to use the ACSI results to improve the site for users?**

- ☐ Not at all confident
- ☐ Slightly confident
- ☐ Moderately confident
- ☐ Very confident
- ☐ Don't know

3. **What information (either related or unrelated to the ACSI) would be most valuable to your team for improving the site?**

4. **By using the ACSI custom questions, what types of information did/does your team hope to obtain? *(Mark all that apply)***

- ☐ We're not using ACSI custom questions

- ☐ Demographic information about the user groups
- ☐ Users' information needs
- ☐ Users' opinions about their experiences using the site
- ☐ Problems/issues that users identify by using the site
- ☐ User suggestions for improving the site
- ☐ User suggestions for adding content
- ☐ Site sections used
- ☐ How users found their way to the site
- ☐ Other (*specify*): ______

5. **How do you plan to use the ACSI results for your site? (*Mark all that apply*)**

- ☐ Provide feedback to the IC
- ☐ Promote the IC and/or the website
- ☐ Establish program priorities
- ☐ Participate in customer satisfaction benchmarking
- ☐ Implement changes to the site
- ☐ Share the results to a contractor that manages the site
- ☐ Use the results to evaluate contractor performance
- ☐ Establish budget priorities
- ☐ Publish/present a paper about the site's experience with the ACSI
- ☐ Make plans for use of other website evaluation methods
- ☐ Other (*specify*): ______
- ☐ Don't know

6. **If you have any further comments you wish to make, please provide those here:**

______

Thank you for participating!

Please do <u>one</u> of the following to submit your responses to Westat:

Email an electronic copy to NIHACSIeval@westat.com

OR

Fax a copy to Terry Koenig at (301) 294-3928

FINAL SURVEY FOR PARTICIPANTS IN THE NIH

American Customer Satisfaction Index (ACSI) Evaluation

Instructions:

If you are completing an *electronic copy* of this survey and emailing it to us:

- Click on a shaded area to type an X or a text response. The text boxes will expand to the size you need.

If you are completing a *hard copy* of this survey and faxing it to us:

- Mark an X in a box to indicate your response.
- Write your text into the boxes; continue on the back of the page if needed. (Be sure to fax to us the back of any pages you write on.)

Section A. Intermediate and Longer Term Outcomes

Items in this section address your team's experiences with using the ACSI to evaluate <website url>, the URL hosted by your IC or office.

1. Which of the following activities has your team done? *(Mark all that apply)*

☐ Reviewed a report of ACSI results before reaching target of 300 completed surveys
☐ Received initial results report (based on first 300 completes) from ForeSee
☐ Participated in feedback meeting with ForeSee SRA to review initial ACSI results
☐ Used ACSI results to monitor our site performance
☐ Used ACSI results to plan site improvements
☐ Implemented planned changes to site based on ACSI results
☐ Used ACSI results to evaluate effects of site changes (pre-launch vs. post-launch)
☐ Used ACSI segmentation (analyses to break results down by site visit and site visitor characteristics) to learn more about site visitors
☐ Modified/tailored custom questions to obtain more in-depth analysis results
☐ Used ACSI feedback in continuous improvement process: Assess site and identify problems → modify site → reassess site and identify problems → modify site, etc.

☐ None of the above

Please indicate the degree to which you agree or disagree with the following statements.

2. Our team understands how to interpret the ACSI results.

☐Strongly agree
☐Somewhat agree
☐Neither agree nor disagree
☐Somewhat disagree
☐Strongly disagree

3. Our team understands how to use the ACSI results to plan site revisions.

☐Strongly agree
☐Somewhat agree
☐Neither agree nor disagree
☐Somewhat disagree
☐Strongly disagree

4. Our team understands how to use segmentation (analyses to break results down by site visit and site visitor characteristics) to evaluate our site.

☐Strongly agree
☐Somewhat agree
☐Neither agree nor disagree
☐Somewhat disagree
☐Strongly disagree

5. Our team has found the overall ACSI satisfaction score to be useful in evaluating our site.

☐Strongly agree
☐Somewhat agree
☐Neither agree nor disagree
☐Somewhat disagree
☐Strongly disagree

6. Our team has found the ACSI element scores (content, functionality, search, etc.) to be useful in evaluating our site.

☐Strongly agree
☐Somewhat agree
☐Neither agree nor disagree
☐Somewhat disagree
☐Strongly disagree

7. **Our team has found the ACSI <u>future behaviors scores</u> (primary resource, recommend, return) to be useful in evaluating our site.**

☐Strongly agree
☐Somewhat agree
☐Neither agree nor disagree
☐Somewhat disagree
☐Strongly disagree

8. **Our team is confident that the ACSI scores reflect our site's strengths and weaknesses.**

☐Strongly agree
☐Somewhat agree
☐Neither agree nor disagree
☐Somewhat disagree
☐Strongly disagree

9. **Our team has found the custom questions to be useful in evaluating our site.**

☐Strongly agree
☐Somewhat agree
☐Neither agree nor disagree
☐Somewhat disagree
☐Strongly disagree

☐We do not use custom questions on our site

10. **How is your team using the ACSI survey data for your site? (*Mark all that apply*)**
To:
☐Provide feedback about the site performance to the IC
☐Promote the IC and/or the website
☐Establish program priorities
☐Participate in customer satisfaction benchmarking
☐Share the results with a contractor that manages the site
☐Evaluate contractor performance
☐Establish budget priorities
☐Publish/present a paper about the site's experience with the ACSI
☐Make plans for use of other website evaluation methods
☐Other *(specify):*

☐Don't know

11. **For which of the following types of site improvements has your team used ACSI survey data to plan changes? *(Mark all that apply)***

☐ Addition of new content areas or topic types
☐ Functionality (e.g. services provided, convenience of the services, visitors' ability to accomplish what they wanted)
☐ Overall look and feel
☐ Homepage redesign
☐ Subpage redesign
☐ Navigation
☐ Search
☐ Site Performance (e.g. speed of loading page, reliability of site performance)
☐ Other (*specify*): _____

☐ Our team has not used the ACSI survey data to plan changes to the site

12. **Will your team use ACSI survey data to plan the next (short term or long term) redesign effort?**

☐ Yes
☐ No

☐ Not sure

13. **How has your team used the ACSI survey data to benchmark your site? *(Mark all that apply)***

To compare:
☐ Different versions of our site over time
☐ Our site with other NIH sites
☐ Our site with the overall NIH site benchmark
☐ Our site with similar government sites
☐ Our site with similar private sector sites
☐ Other (*specify*): _____

☐ Our team has not used the ACSI survey data for benchmarking

Please indicate the degree to which you agree or disagree with the following statement.

14. **Our team is satisfied with using the ACSI to help evaluate our site.**

☐ Strongly agree
☐ Somewhat agree
☐ Neither agree nor disagree
☐ Somewhat disagree
☐ Strongly disagree

15. Has your use of the ACSI impacted use of other evaluation methods?

- ☐ No [GO TO 16]
- ☐ Yes; the team substituted the ACSI for other evaluation method(s) [GO TO 16]
- ☐ Yes; use of the ACSI strengthened the team's use of other evaluation methods

15a. Besides the ACSI, what other evaluation method(s) has your team used? *(Mark all that apply)*

- ☐ Expert or heuristic review
- ☐ Usability testing
- ☐ Focus group
- ☐ Survey
- ☐ Web log software
- ☐ Audience measurement and profiling
- ☐ Other (*specify*): _____

16. Would your team use the ACSI if the license fee had to be paid out of your own IC's budget?

- ☐ Yes; on a continuous annual basis [GO TO Section B]
- ☐ Yes; but on a periodic or as-needed basis [GO TO 16a]

- ☐ Not sure [GO TO 16a]

- ☐ No; the ACSI is not of sufficient value to us to continue to use it [GO TO Section B]

16a. Please indicate why you are not sure or would change to periodic use *(Mark all that apply)*

- ☐ Budget constraints
- ☐ Staff time constraints
- ☐ Other (*specify*): _____

<u>Section B. Evaluation of Trans-NIH Processes</u>

The next few items address your participation in the Trans-NIH ACSI project.

1. There have been five Trans-NIH meetings about the ACSI. How many of these meetings have you or a team member attended?

- ☐ None of them [GO TO Section C]
- ☐ Some of them
- ☐ All of them

2. **What were the benefits of attending the Trans-NIH meeting(s)?** ***(Mark all that apply)***

☐Receiving feedback about NIH sites' satisfaction scores
☐Sharing information with other NIH site teams
☐Networking with other NIH site teams
☐Benchmarking
☐Identifying other teams that manage sites similar to ours
☐Learning more about the ACSI model and approach
☐Learning about additional ways to interpret and use the ACSI data
☐Other (*specify*): _____

Section C. Website and Team Background

The following items are included to collect some additional background information about your team and site.

1. **What is the average number of monthly visits to your site?**
______

2. **Please indicate how many FTEs provide the following types of support for your web site:**

	**Types of Site Support**		
	**Content**	**Technical**	**Total FTEs**
Federal FTEs	_____	_____	_____
Contractor FTEs	_____	_____	_____
Other (specify):	_____	_____	_____
**TOTAL FTEs**	_____	_____	_____

3. **ForeSee uses the following four categories to classify website types. Which do you consider your site to be?**

☐E-commerce
☐Information/News
☐Portal/Department main website
☐Recruitment/Careers

☐Our site does not fit these categories; our site type is (*specify*): _____

4. **Which of the following barriers prevent your team from making planned changes to your site? *(Mark all that apply)***

☐ Insufficient time to implement the planned changes
☐ Staff time constraints
☐ Financial resource constraints
☐ Other (*specify*): _____

☐ None; our team is able to implement planned changes to our site

5. **Please provide any further comments you wish to make:**

Thank you for participating!

Please do one of the following to submit your responses to Westat:

Email an electronic copy to NIHACSIeval@westat.com

OR

Fax a copy to Daniel Sangria at (301) 610-4950

INITIAL INTERVIEW QUESTIONS FOR SELECTED PARTICIPANTS

(for teams that have completed at least the first ACSI feedback cycle)

Website Team Background

1. I'd like to start by getting some basic information about your website team. Please describe for me how your team is structured and how it functions.

Implementation Process

2. How well did the process of implementing the ACSI meet your expectations?
   Which expectations were well met?
   Which expectations were not met well?

3. How was the sampling rate determined?
   Did you change this at any time?

4. How did you identify/select the loyalty factor?
   Did you change this at any time?

5. How did you identify/select the custom questions for your site?
   What custom questions of your own did you create?

Data Collection Process

6. How well did the process of collecting the ACSI data meet your expectations?
   Which expectations were well met?
   Which expectations were not met well?

ACSI Results/Feedback

The Overall and Component Scores

7. To what degree did your site's scores match the team's expectations?

   What did the ACSI feedback point out that your team did not know about your site?
   - How did your team treat those data?

   What did the ACSI feedback confirm for your team about the site?
   - … that you'd already suspected but had no data about?
   - … that you'd learned from other evaluation methods?

Were there counterintuitive results?
- How did your team treat those data?

Were there results that could not be interpreted?
- What did your team do about those results?

Custom Questions

8. To what degree did the custom question data provide the information your team expected?
   Which data were most useful?
   Which data were not useful?

Meetings

9. How well did the ForeSee feedback meetings meet your teams' expectations?
   Which expectations were well met?
   Which expectations were not met well?
   What did your team learn from the meetings?
   How useful have these meetings been?

10. How satisfied is your team with the feedback meeting process?
    What factors have contributed to the success of the meetings?
    What would contribute to improvement?

<u>Use of ACSI Feedback</u>

11. How has your team used the ACSI data?
    How has the ACSI data been most helpful?
    How has your team used the benchmarking data?
    Has your team shared the ACSI findings with your IC management?
    - What was the impact of your team's use of the ACSI on your IC?

12. Based on the ACSI feedback, what plans did your team make?
    […about managing the site, planning revisions, obtaining resources, etc.]
    What changes has your team planned to make to your site?
    What changes has your team actually made to your site?
    How has use of the ACSI affected your team's plans to conduct other website evaluation activities?

13. How successful has your team been at implementing plans so far?
    How successful was your team at translating ACSI findings into site improvements?

Trans-NIH Effort

14. Apart from use of the ACSI, what benefits has your team experienced so far from its involvement with the other NIH website teams?

15. What drawbacks have there been to your team's involvement with other NIH website teams?

16. For your team, what are the main "Lessons Learned" from involvement in the trans-NIH implementation of the ACSI?
    What factors have contributed to its success?
    What would contribute to improvement?

FINAL INTERVIEW FOR SELECTED PARTICIPANTS

Process and Outcomes

1. How has your experience using the ACSI met expectations?

   In what ways has using the ACSI not met your expectations?

2. What insights did the ACSI survey data provide about your site?

   How has your view of your site visitors changed since using the ACSI?
   What value has been added by getting market segmentation data?

3. What factors have contributed to success of each of the following activities?

   a. Interpreting ACSI survey data
   b. Planning how to use the data to make site improvements
   c. Implementing site revisions [if relevant]
   d. Tracking ACSI score changes after making revisions to the site [if relevant]

4. What difficulties, if any, did your team have with each of the following activities?

   a. Interpreting ACSI survey data
   b. Planning how to use the data to make site improvements
   c. Implementing site revisions [if relevant]
   d. Tracking ACSI score changes after making revisions to the site [if relevant]

   What would contribute to improvement in these areas?

5. How satisfied is your team with use of the custom questions?

   What benefits did you get from using custom questions?
   What would contribute to improvement?

6. How satisfied is your team with using segmentation to get more in-depth analysis of your data?

   What benefits did you get from doing segmentations of your data?
   What would contribute to improvement?

7. Recently, the first "standard" version of a custom question was developed for trans-NIH use. [The question added to the ACSI is: "What do you plan to do with the information you found today on this site?"] The purpose of fielding "standard" custom questions is to provide comparable data across NIH sites for demographic and site experience questions. What has been your experience with the new question so far? [Some sites may not a) have data yet or b) have interpreted data yet.]

   How will your team use the information provided by the trans-NIH custom question?
   What additional trans-NIH questions would your team find useful?

8. How has your team benefited most from the benchmarking data?

   What factors account for successful use of these data?
   What factors would improve your use of these data?

9. How has your team's approach to site management changed as a result of using the ACSI?

10. How has use of the ACSI changed your team's views about evaluation of your site?

    What impact has your team's use of the ACSI had on:
    - Plans for using other evaluation methods?
    - Actual use of other evaluation methods?

    What other specific evaluation activities, if any, is your team planning?
    What other types of information does your team expect to collect to follow up on ACSI results?

11. Supplemental funding awarded to the NIH Leadership Team allowed NIH sites using the ACSI to receive additional months on their licenses. Did your team consider stopping use of the ACSI at the end of the original period (not taking additional months)?

    If so, why?
    Did your IC (higher management) consider stopping the ACSI license for your site? If so, why?

12. How has your IC higher management reacted to use of the ACSI for your site?

    Does your IC higher management understand and support use of the ACSI? Why or why not?

    How has use of the ACSI changed your IC's views about using the ACSI as a site evaluation tool?

13. Has your team used the ACSI survey results in other ways besides evaluating your site?

If so, how?

- o What unanticipated uses has your team found for ACSI survey results?

If not, why not?

14. In what ways has the ACSI been most helpful?

15. For your team, what are the main “Lessons Learned” from involvement in the trans-NIH implementation of the ACSI?

What factors have contributed to the success of the trans-NIH effort?

What would contribute to improvement of the trans-NIH effort?
