# Supplementary material for: Web Evaluation at the US National Institutes of Health: Use of the American Customer Satisfaction Index Online Customer Survey
Source: J Med Internet Res. 2008 Feb 15;10(1):e4. doi: 10.2196/jmir.944 (PMC2483849; doi:10.2196/jmir.944)
Supplement: Supplementary file 2 [file jmir_v10i1e4_app2.ppt]

## Slide 1
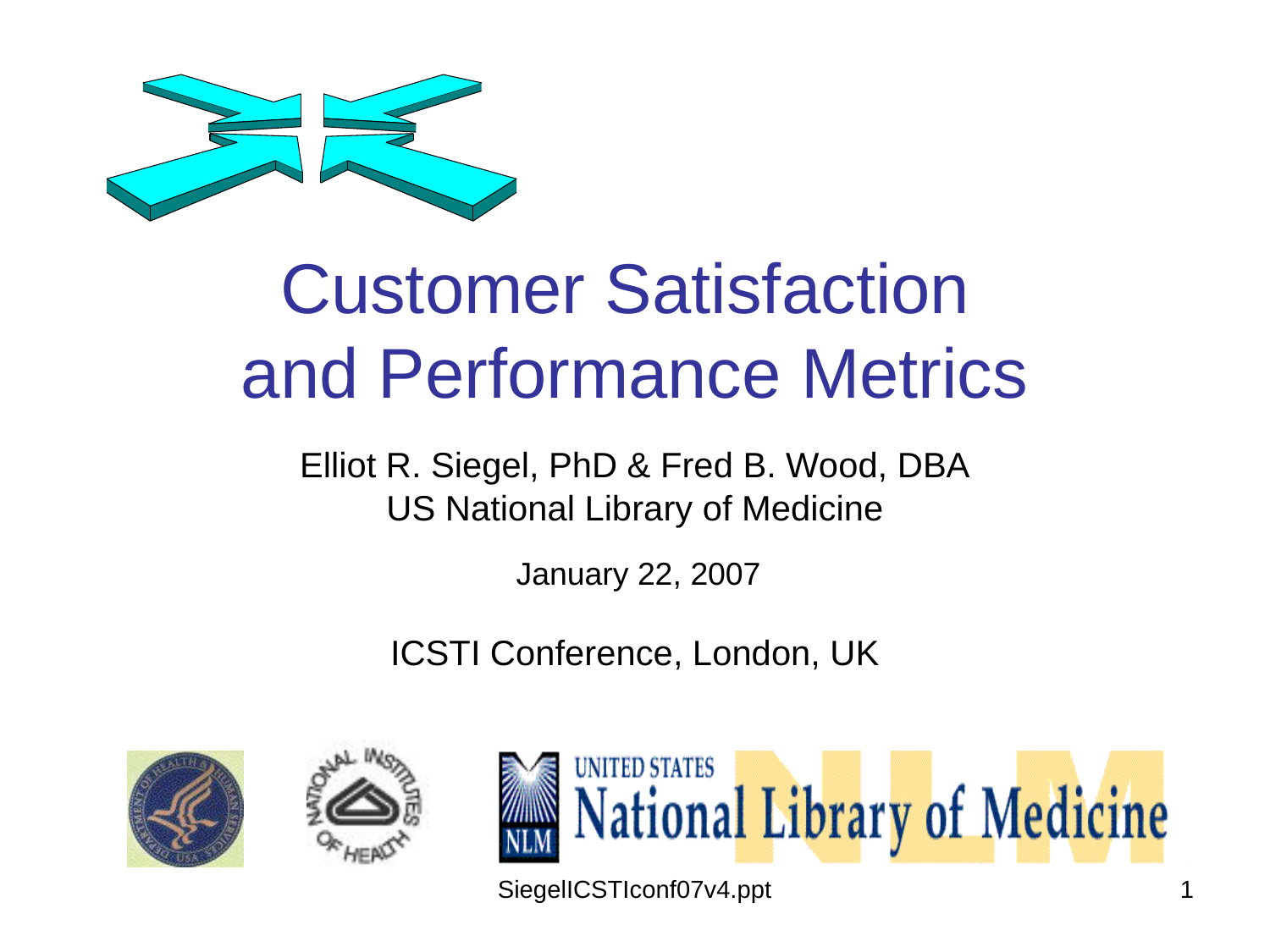

# Customer Satisfaction and Performance Metrics
Elliot R. Siegel, PhD & Fred B. Wood, DBA
US National Library of Medicine
 January 22, 2007
ICSTI Conference, London, UK
SiegelICSTIconf07v4.ppt
1

## Slide 2
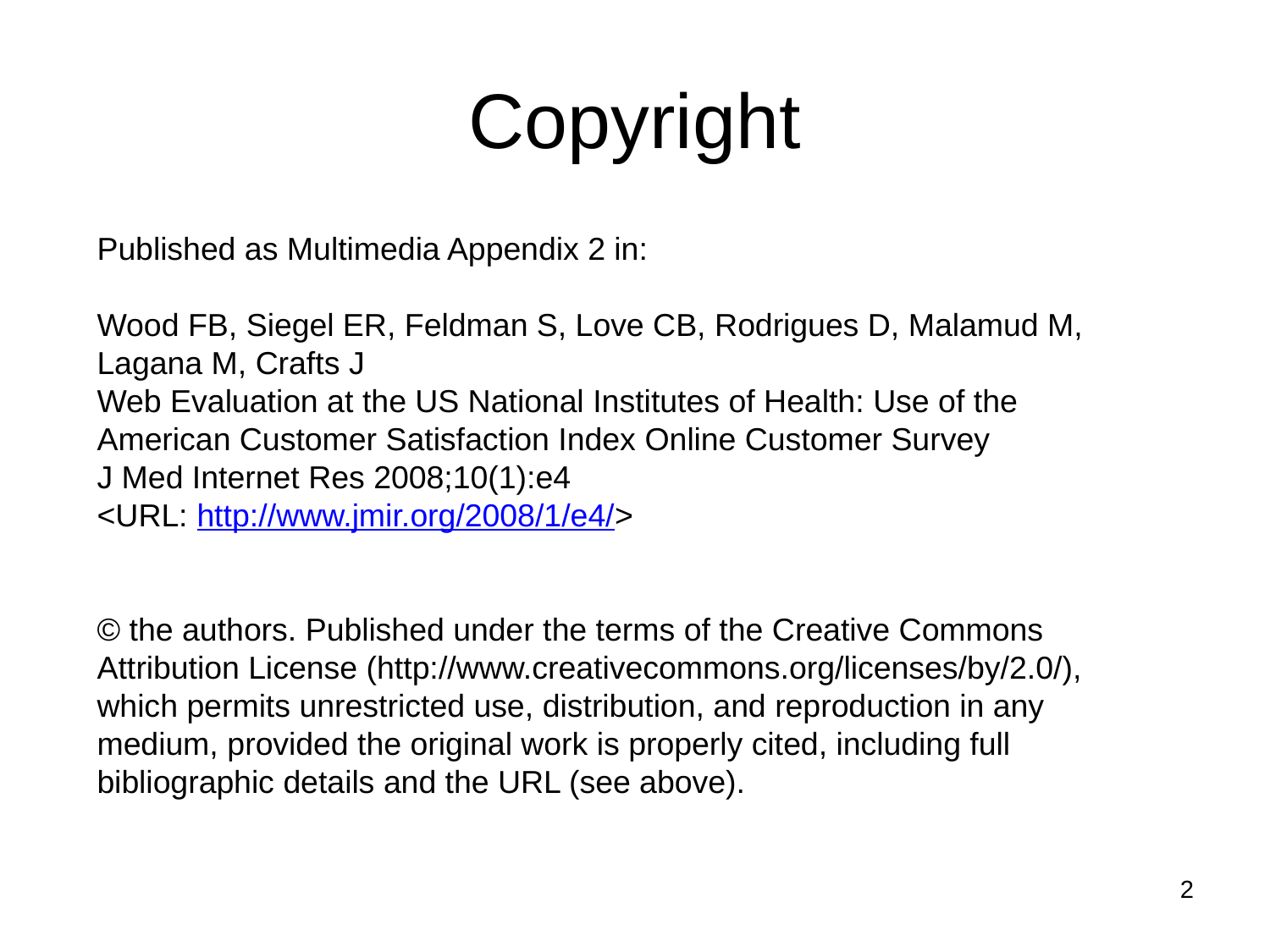

# Copyright
Published as Multimedia Appendix 2 in:
Wood FB, Siegel ER, Feldman S, Love CB, Rodrigues D, Malamud M, Lagana M, Crafts J
Web Evaluation at the US National Institutes of Health: Use of the American Customer Satisfaction Index Online Customer Survey
J Med Internet Res 2008;10(1):e4
<URL: http://www.jmir.org/2008/1/e4/>
© the authors. Published under the terms of the Creative Commons Attribution License (http://www.creativecommons.org/licenses/by/2.0/), which permits unrestricted use, distribution, and reproduction in any medium, provided the original work is properly cited, including full bibliographic details and the URL (see above).
2

## Slide 3
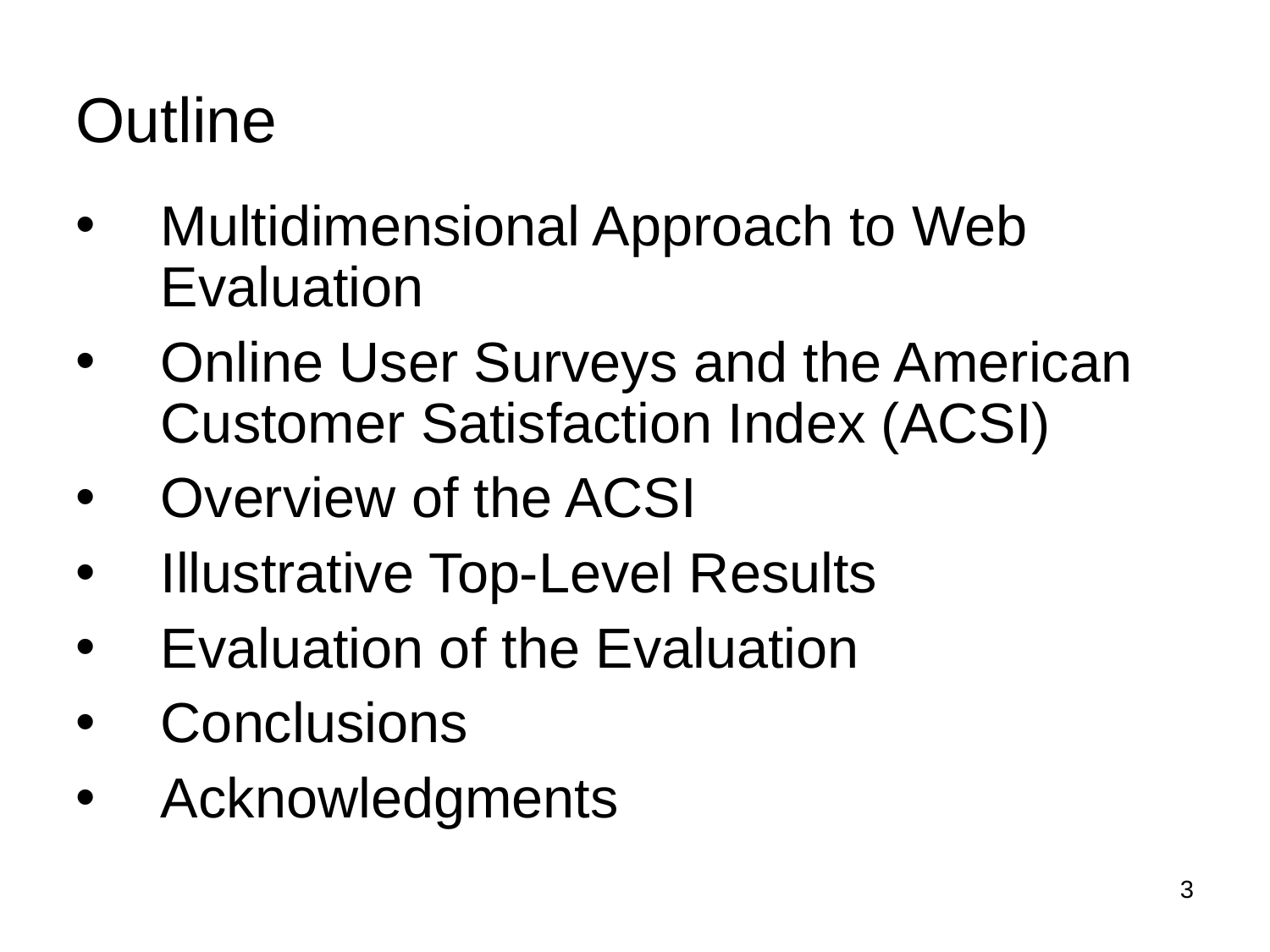

# Outline
Multidimensional Approach to Web Evaluation
Online User Surveys and the American Customer Satisfaction Index (ACSI)
Overview of the ACSI
Illustrative Top-Level Results
Evaluation of the Evaluation
Conclusions
Acknowledgments
3

## Slide 4
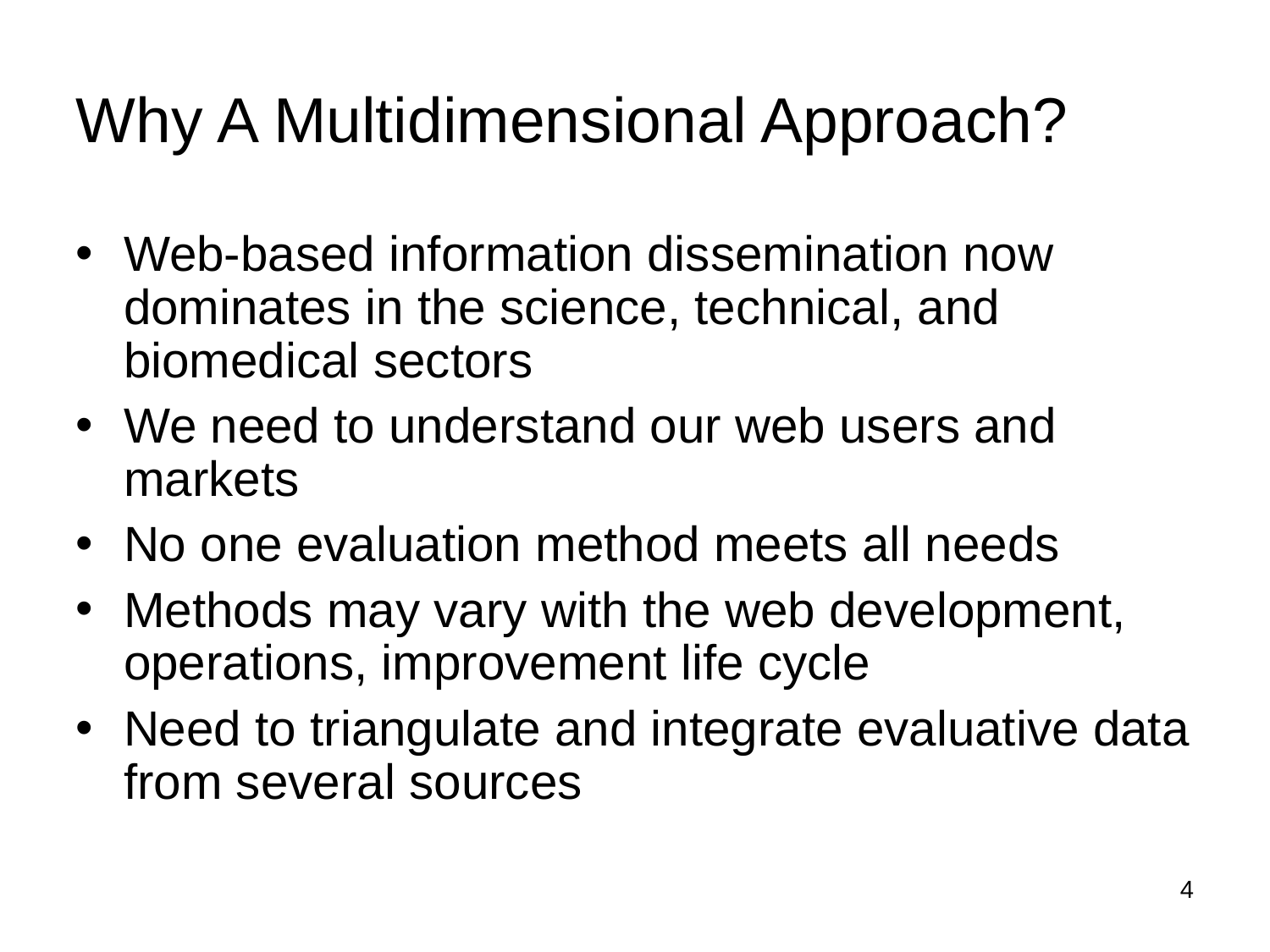

# Why A Multidimensional Approach?
Web-based information dissemination now dominates in the science, technical, and biomedical sectors
We need to understand our web users and markets
No one evaluation method meets all needs
Methods may vary with the web development, operations, improvement life cycle
Need to triangulate and integrate evaluative data from several sources
4

## Slide 5
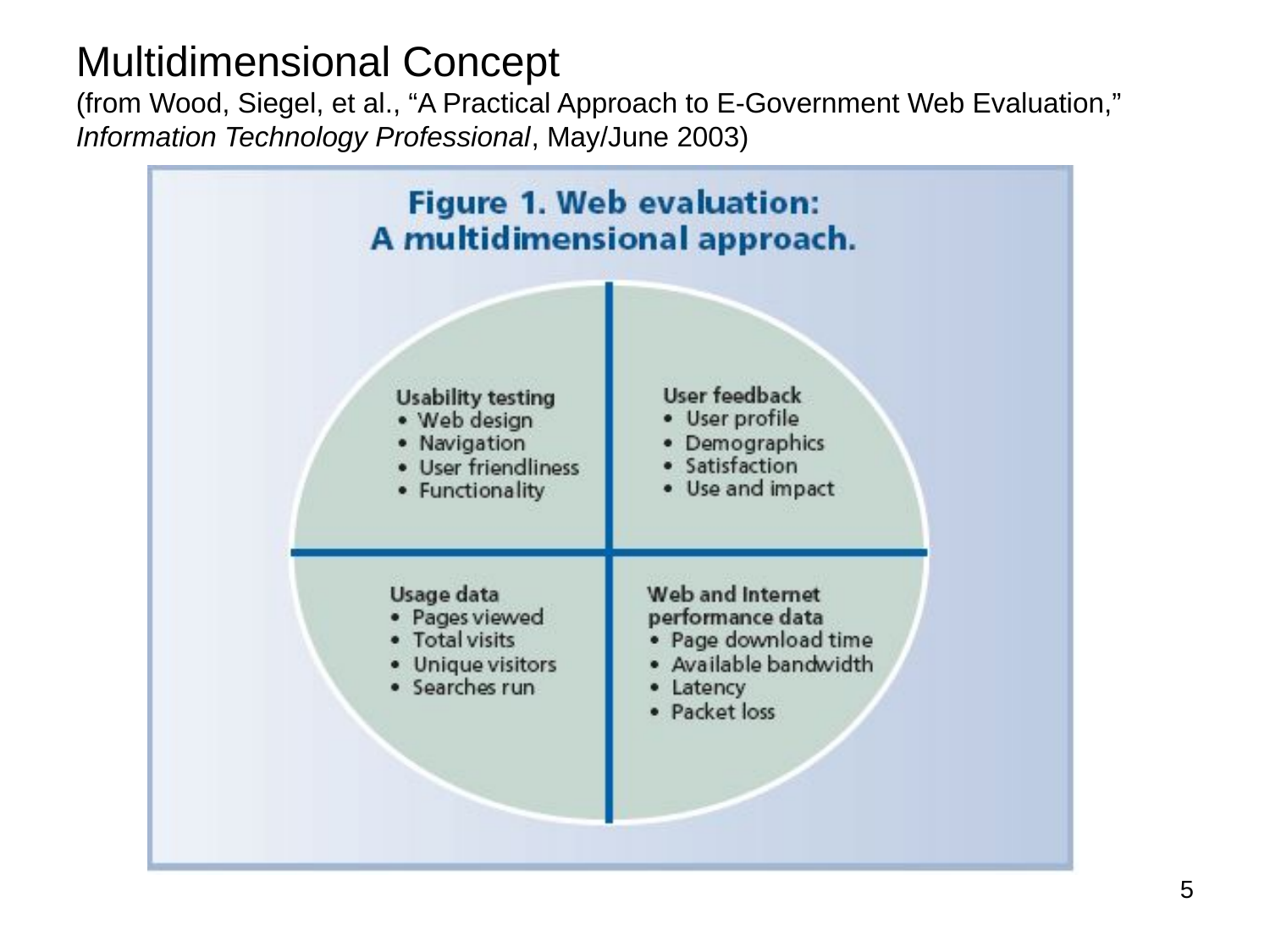

# Multidimensional Concept (from Wood, Siegel, et al., “A Practical Approach to E-Government Web Evaluation,” Information Technology Professional, May/June 2003)
5

## Slide 6
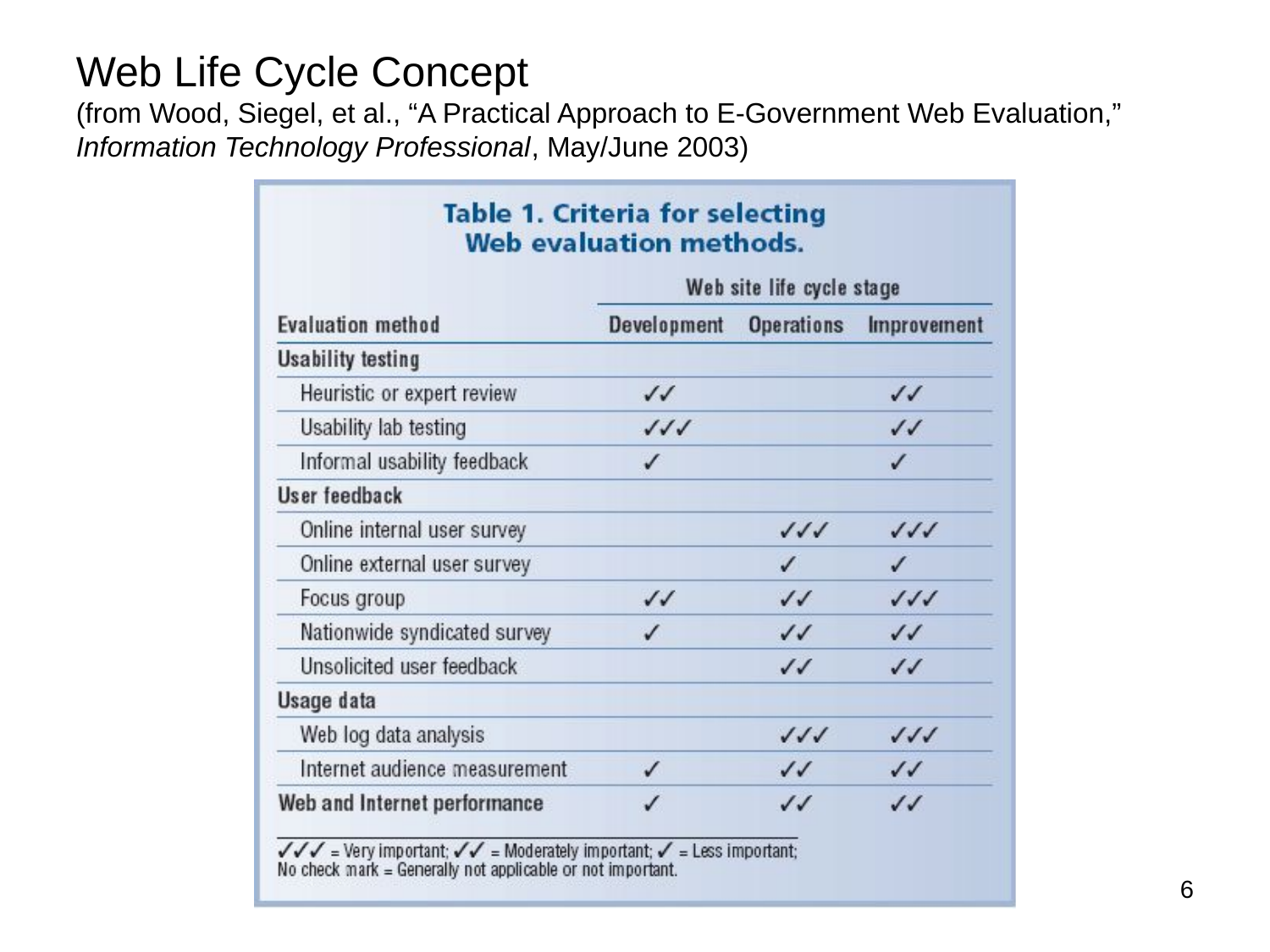

# Web Life Cycle Concept (from Wood, Siegel, et al., “A Practical Approach to E-Government Web Evaluation,” Information Technology Professional, May/June 2003)
6

## Slide 7
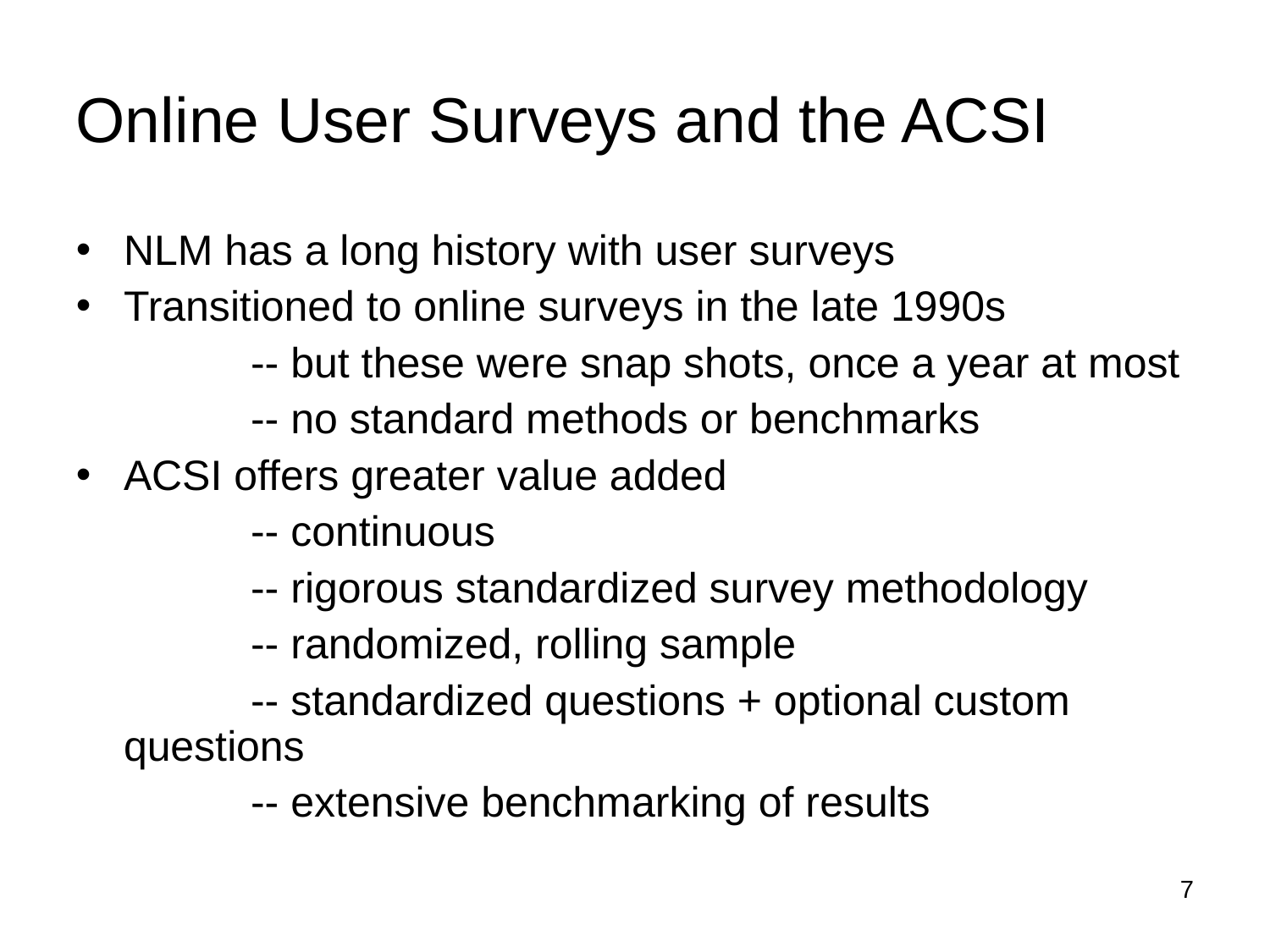

# Online User Surveys and the ACSI
NLM has a long history with user surveys
Transitioned to online surveys in the late 1990s
	-- but these were snap shots, once a year at most
	-- no standard methods or benchmarks
ACSI offers greater value added
	-- continuous
	-- rigorous standardized survey methodology
	-- randomized, rolling sample
	-- standardized questions + optional custom questions
	-- extensive benchmarking of results
7

## Slide 8
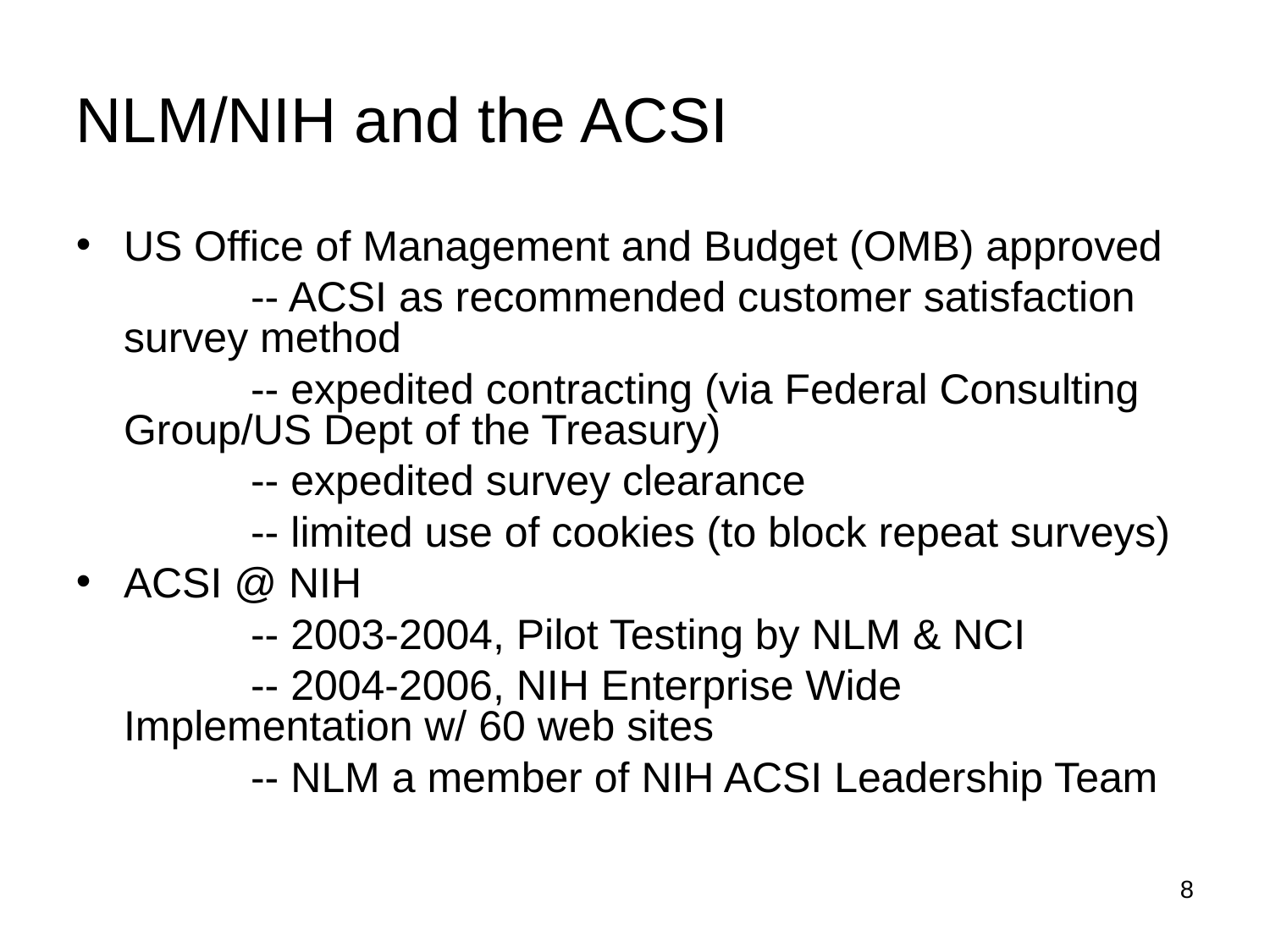

# NLM/NIH and the ACSI
US Office of Management and Budget (OMB) approved
	-- ACSI as recommended customer satisfaction survey method
	-- expedited contracting (via Federal Consulting Group/US Dept of the Treasury)
	-- expedited survey clearance
	-- limited use of cookies (to block repeat surveys)
ACSI @ NIH
	-- 2003-2004, Pilot Testing by NLM & NCI
	-- 2004-2006, NIH Enterprise Wide Implementation w/ 60 web sites
	-- NLM a member of NIH ACSI Leadership Team
8

## Slide 9
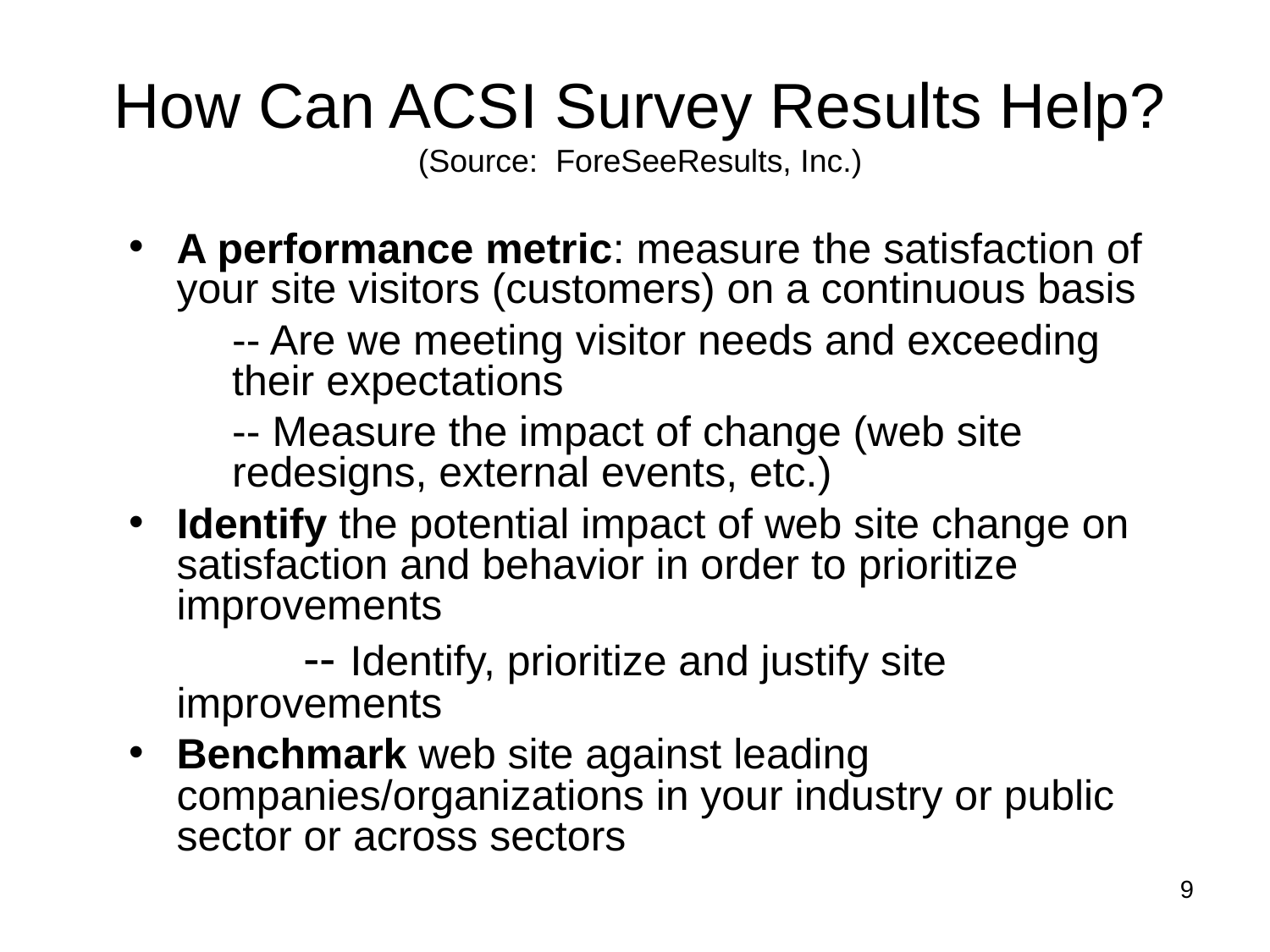

# How Can ACSI Survey Results Help? (Source: ForeSeeResults, Inc.)
A performance metric: measure the satisfaction of your site visitors (customers) on a continuous basis
-- Are we meeting visitor needs and exceeding their expectations
-- Measure the impact of change (web site redesigns, external events, etc.)
Identify the potential impact of web site change on satisfaction and behavior in order to prioritize improvements
	-- Identify, prioritize and justify site improvements
Benchmark web site against leading companies/organizations in your industry or public sector or across sectors
9

## Slide 10
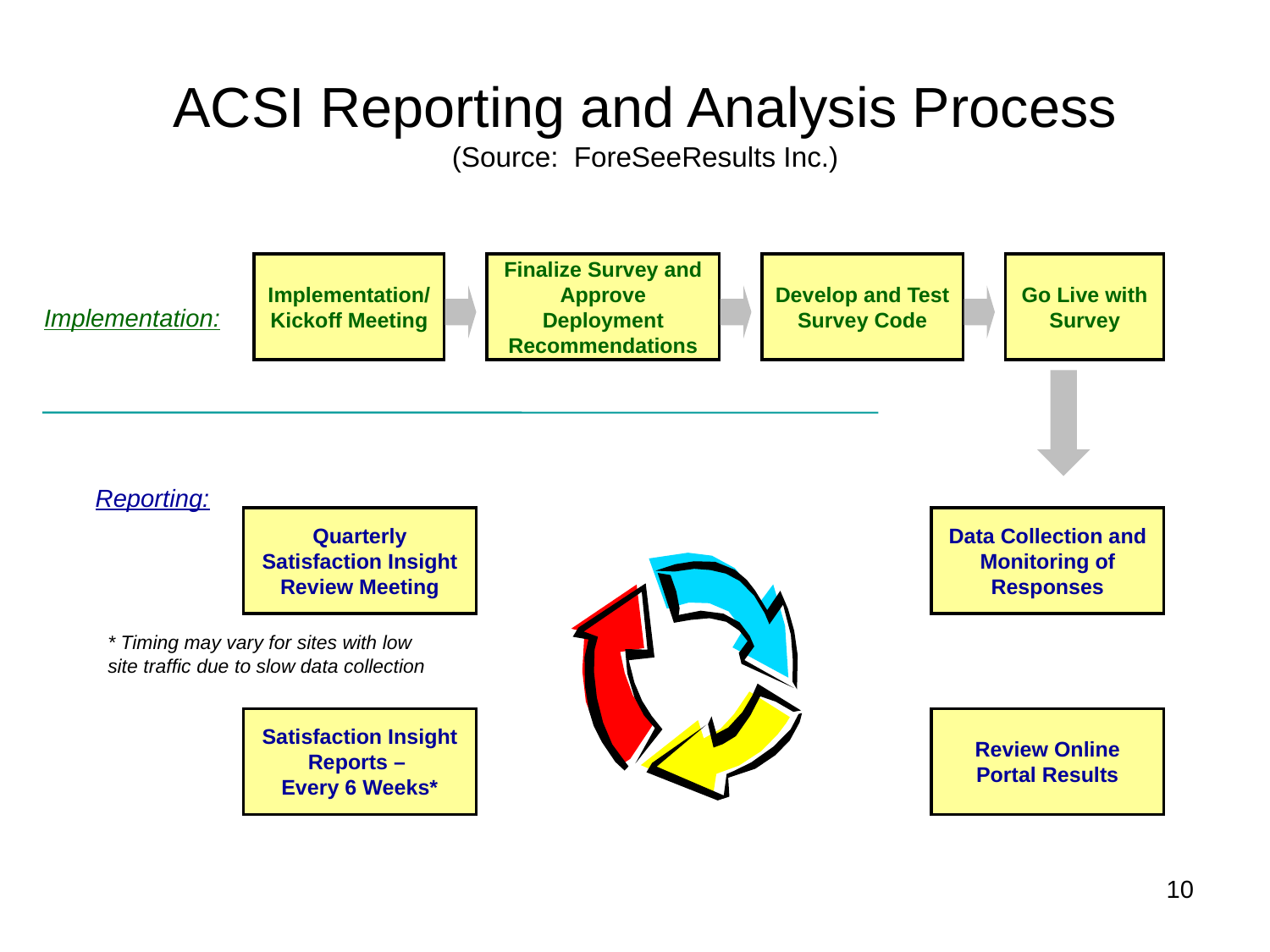

# ACSI Reporting and Analysis Process (Source: ForeSeeResults Inc.)
Implementation/ Kickoff Meeting
Finalize Survey and Approve Deployment Recommendations
Develop and Test Survey Code
Go Live with Survey
Implementation:
Reporting:
Quarterly Satisfaction Insight Review Meeting
Data Collection and Monitoring of Responses
* Timing may vary for sites with low site traffic due to slow data collection
Satisfaction Insight Reports –
Every 6 Weeks*
Review Online Portal Results
10

## Slide 11
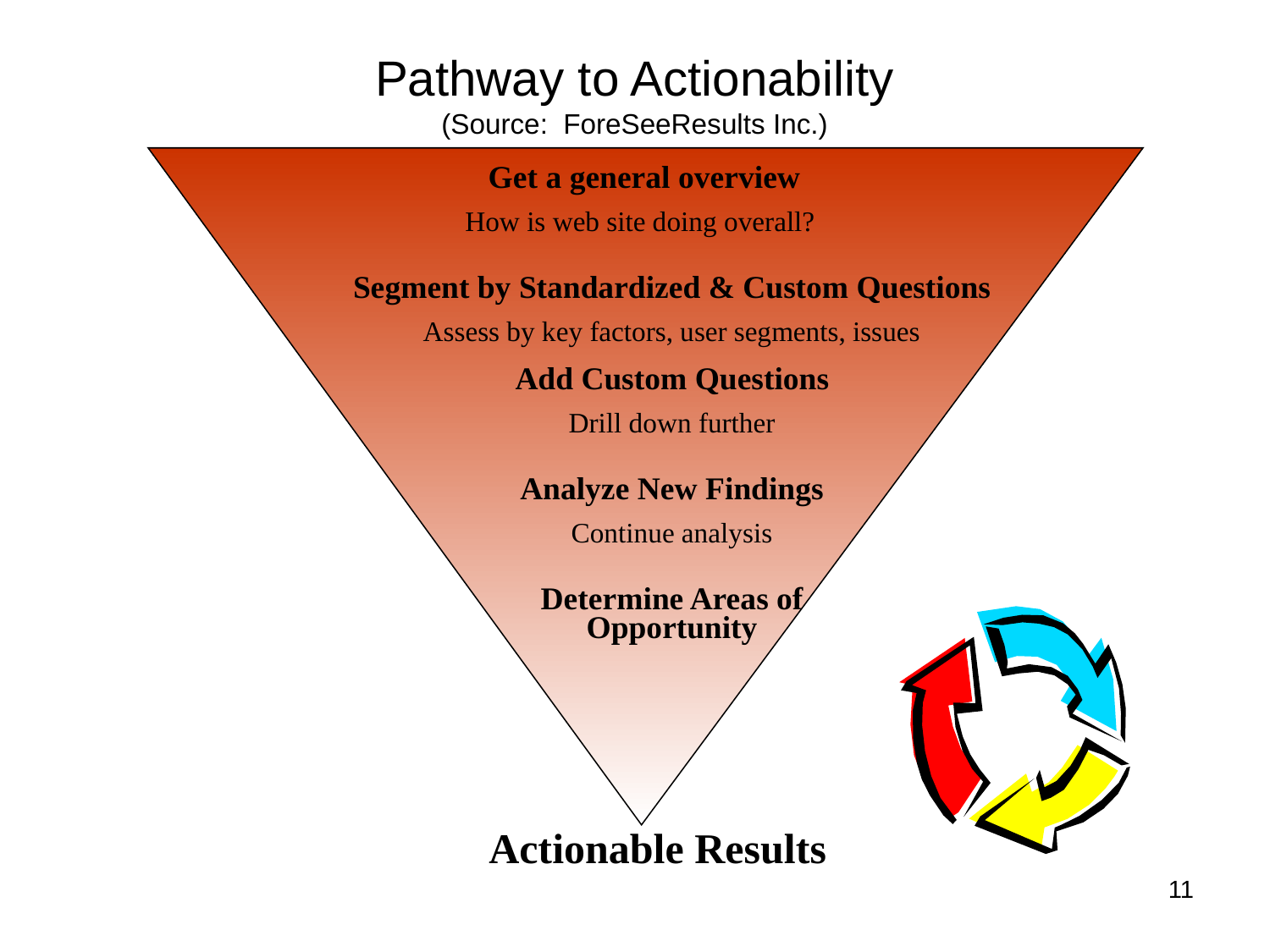

# Pathway to Actionability (Source: ForeSeeResults Inc.)
 Get a general overview
How is web site doing overall?
Segment by Standardized & Custom Questions
Assess by key factors, user segments, issues
Add Custom Questions
Drill down further
Analyze New Findings
Continue analysis
Determine Areas of
Opportunity
Actionable Results
11

## Slide 12
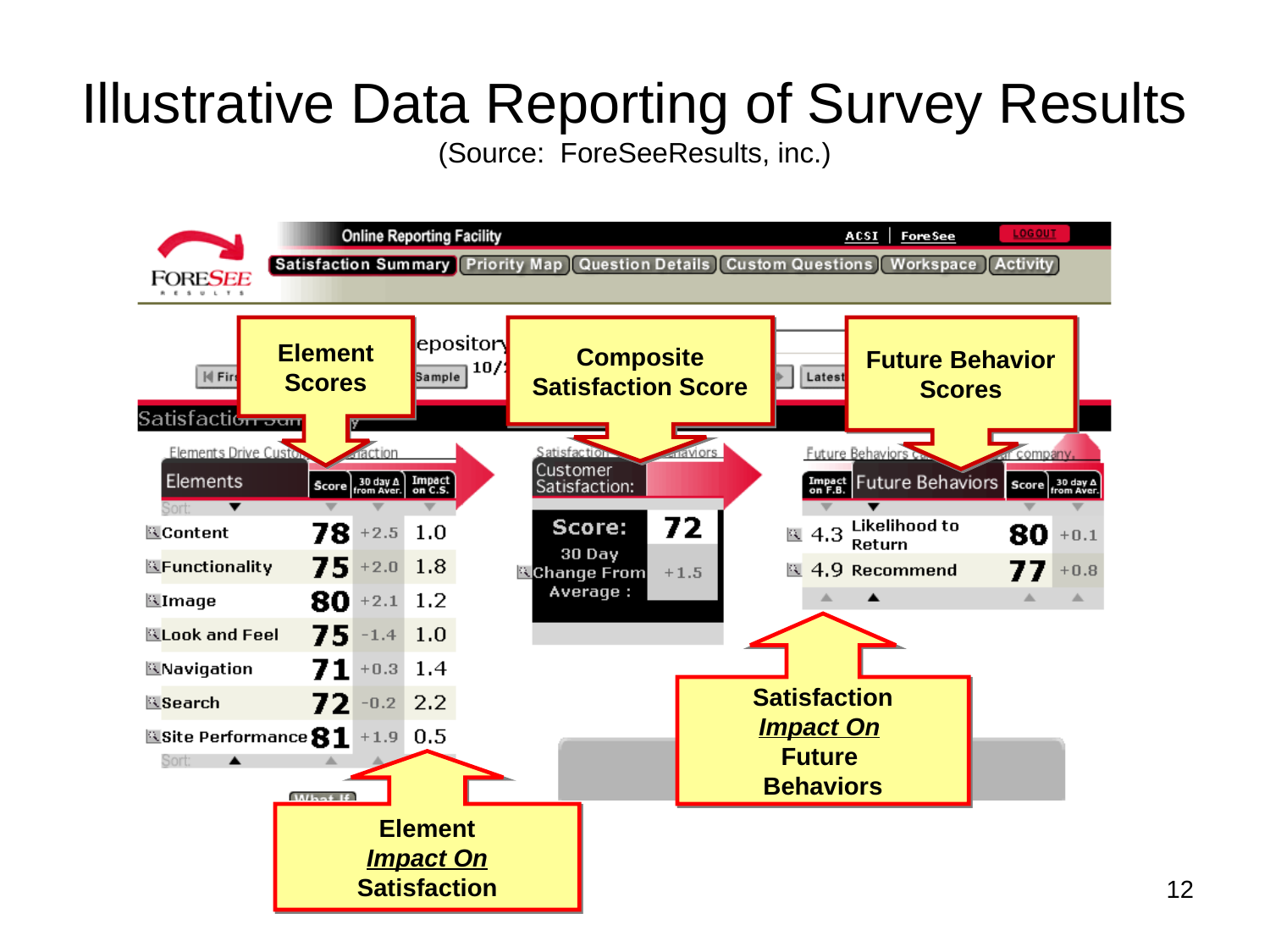

# Illustrative Data Reporting of Survey Results (Source: ForeSeeResults, inc.)
Element Scores
Composite Satisfaction Score
Future Behavior Scores
Satisfaction
Impact On
Future
Behaviors
Element
Impact On
Satisfaction
12

## Slide 13
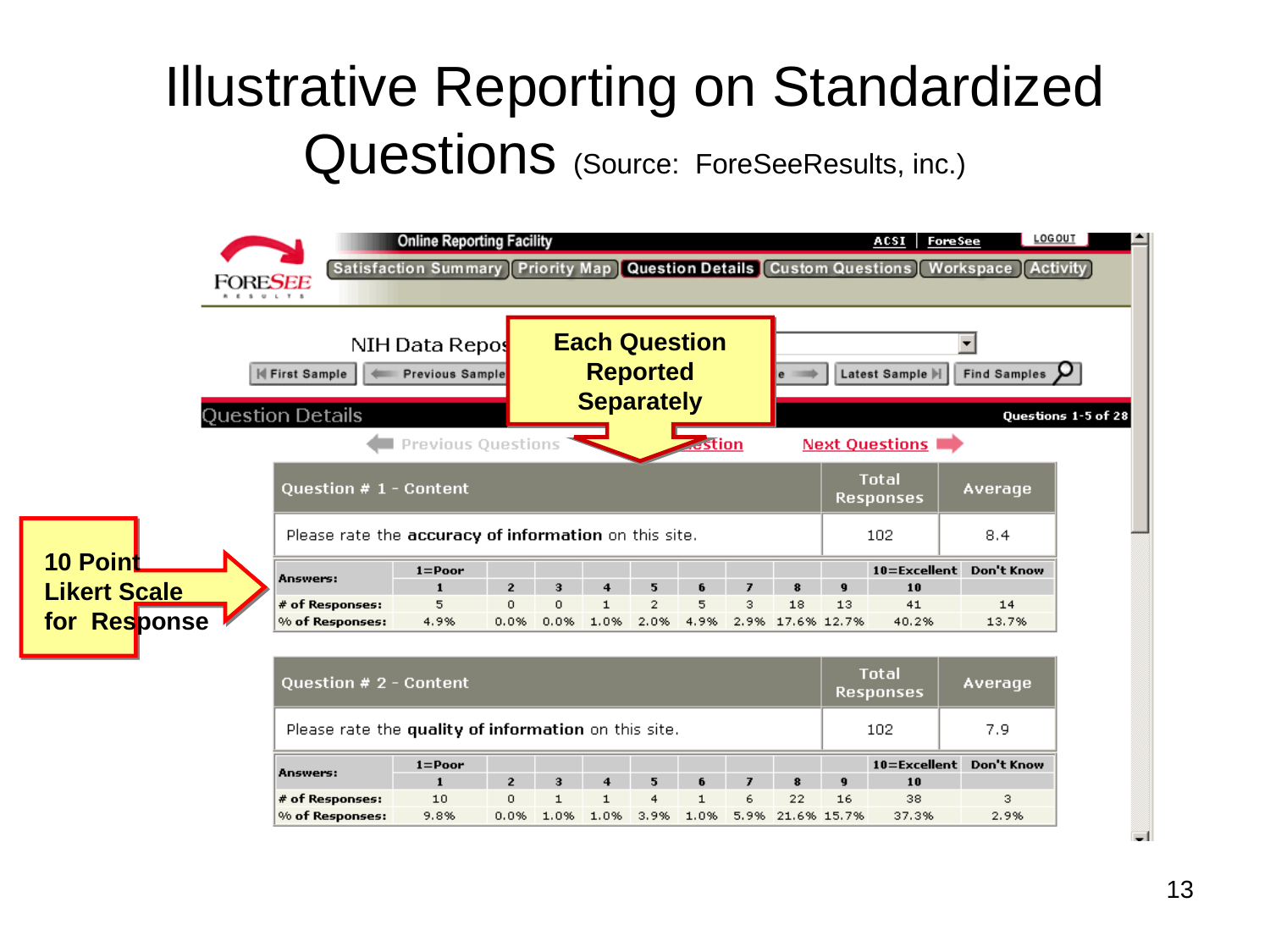

# Illustrative Reporting on Standardized Questions (Source: ForeSeeResults, inc.)
Each Question Reported Separately
10 Point Likert Scale for Response
13

## Slide 14
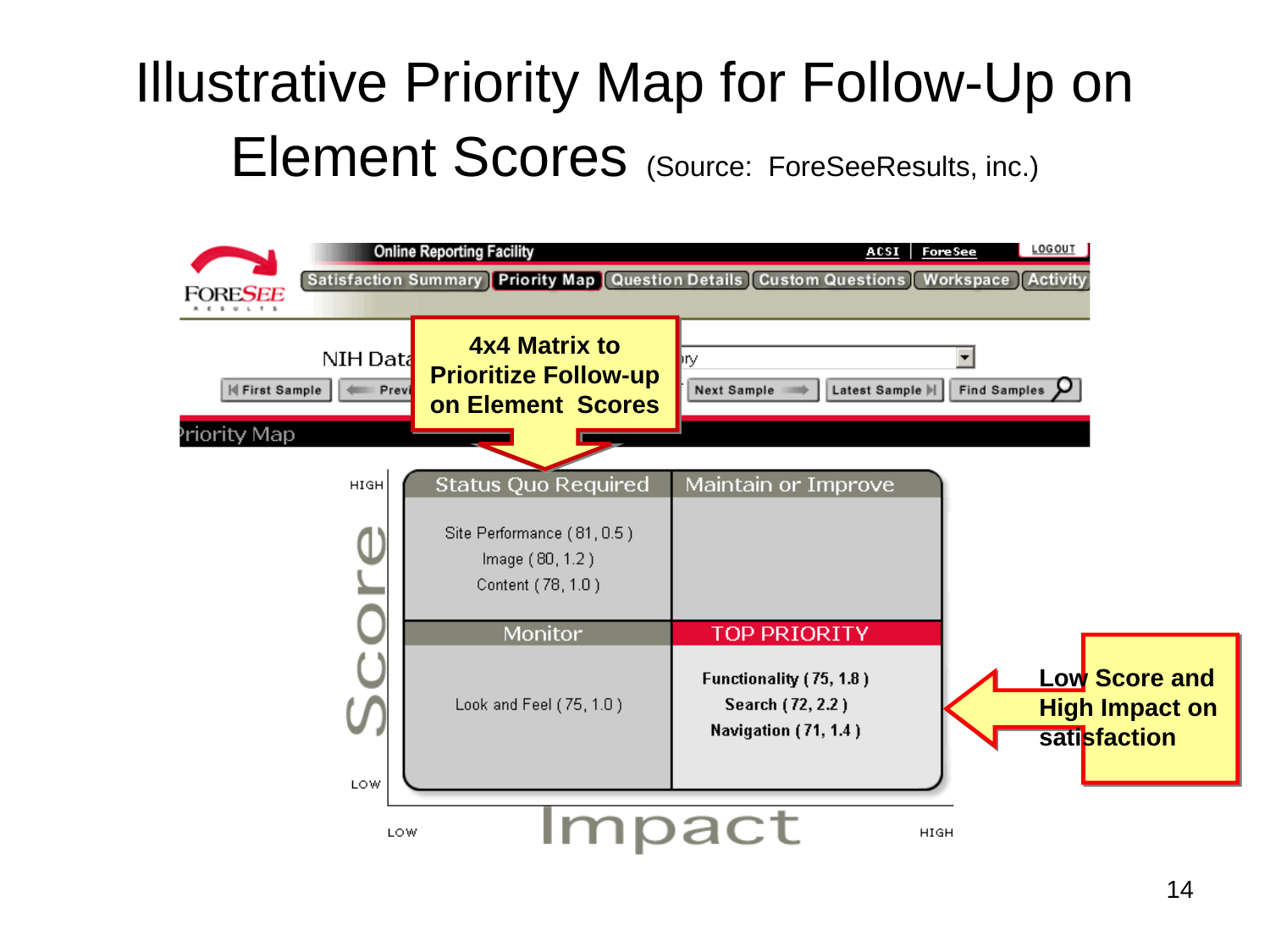

# Illustrative Priority Map for Follow-Up on Element Scores (Source: ForeSeeResults, inc.)
4x4 Matrix to Prioritize Follow-up on Element Scores
Low Score and
High Impact on satisfaction
14

## Slide 15
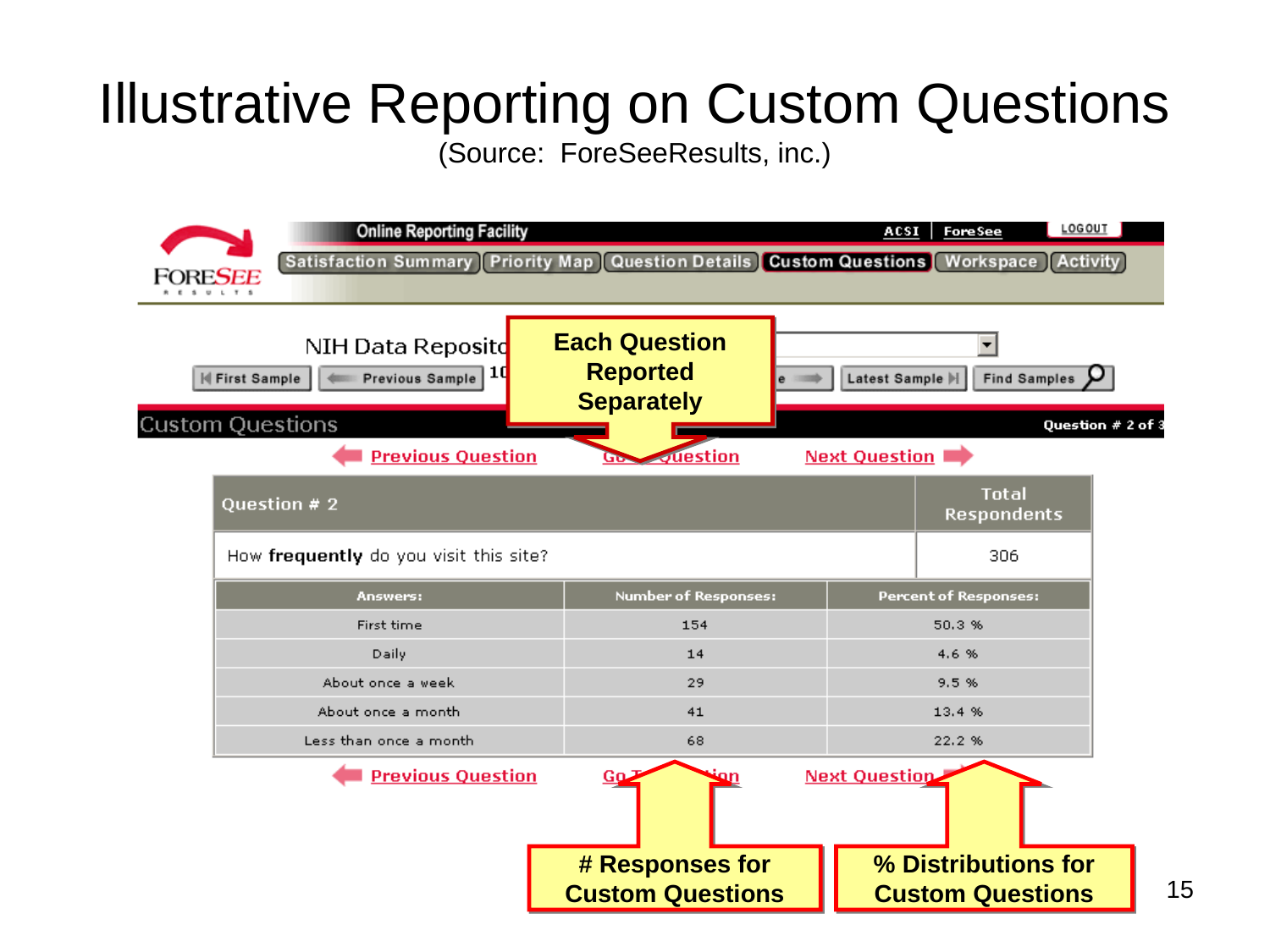

# Illustrative Reporting on Custom Questions (Source: ForeSeeResults, inc.)
Each Question Reported Separately
# Responses for Custom Questions
% Distributions for Custom Questions
15

## Slide 16
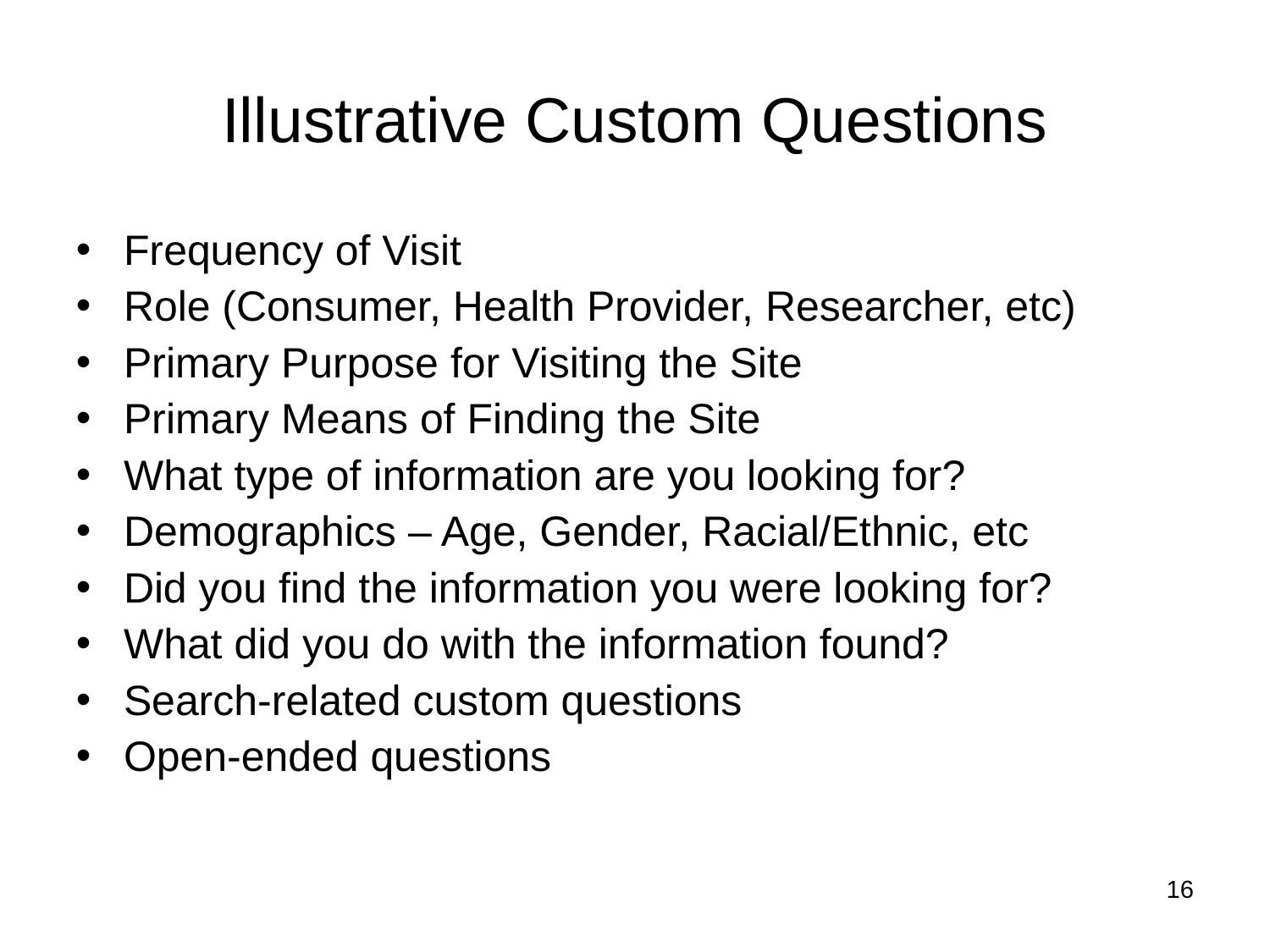

# Illustrative Custom Questions
Frequency of Visit
Role (Consumer, Health Provider, Researcher, etc)
Primary Purpose for Visiting the Site
Primary Means of Finding the Site
What type of information are you looking for?
Demographics – Age, Gender, Racial/Ethnic, etc
Did you find the information you were looking for?
What did you do with the information found?
Search-related custom questions
Open-ended questions
16

## Slide 17
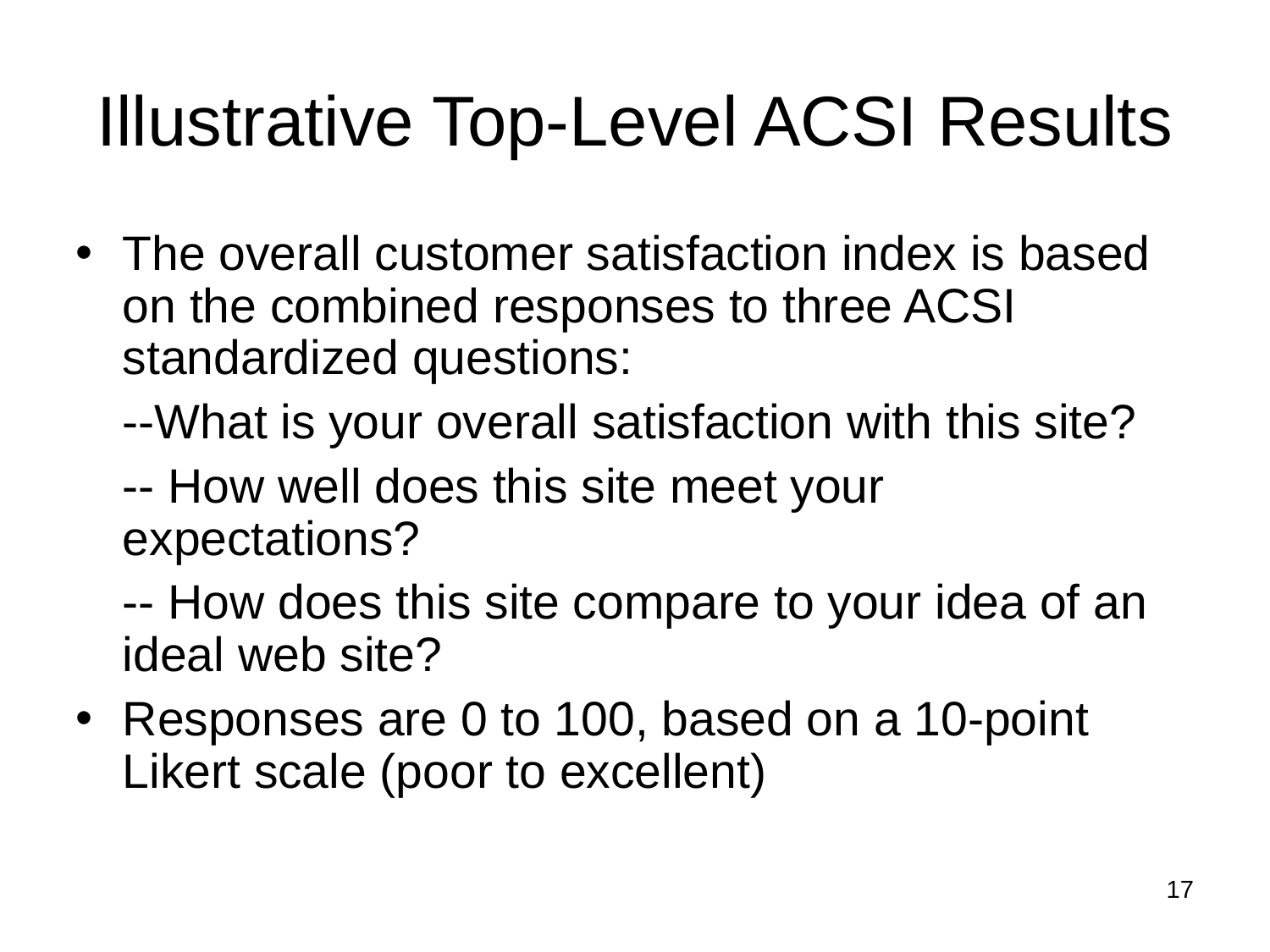

# Illustrative Top-Level ACSI Results
The overall customer satisfaction index is based on the combined responses to three ACSI standardized questions:
	--What is your overall satisfaction with this site?
	-- How well does this site meet your expectations?
	-- How does this site compare to your idea of an ideal web site?
Responses are 0 to 100, based on a 10-point Likert scale (poor to excellent)
17

## Slide 18
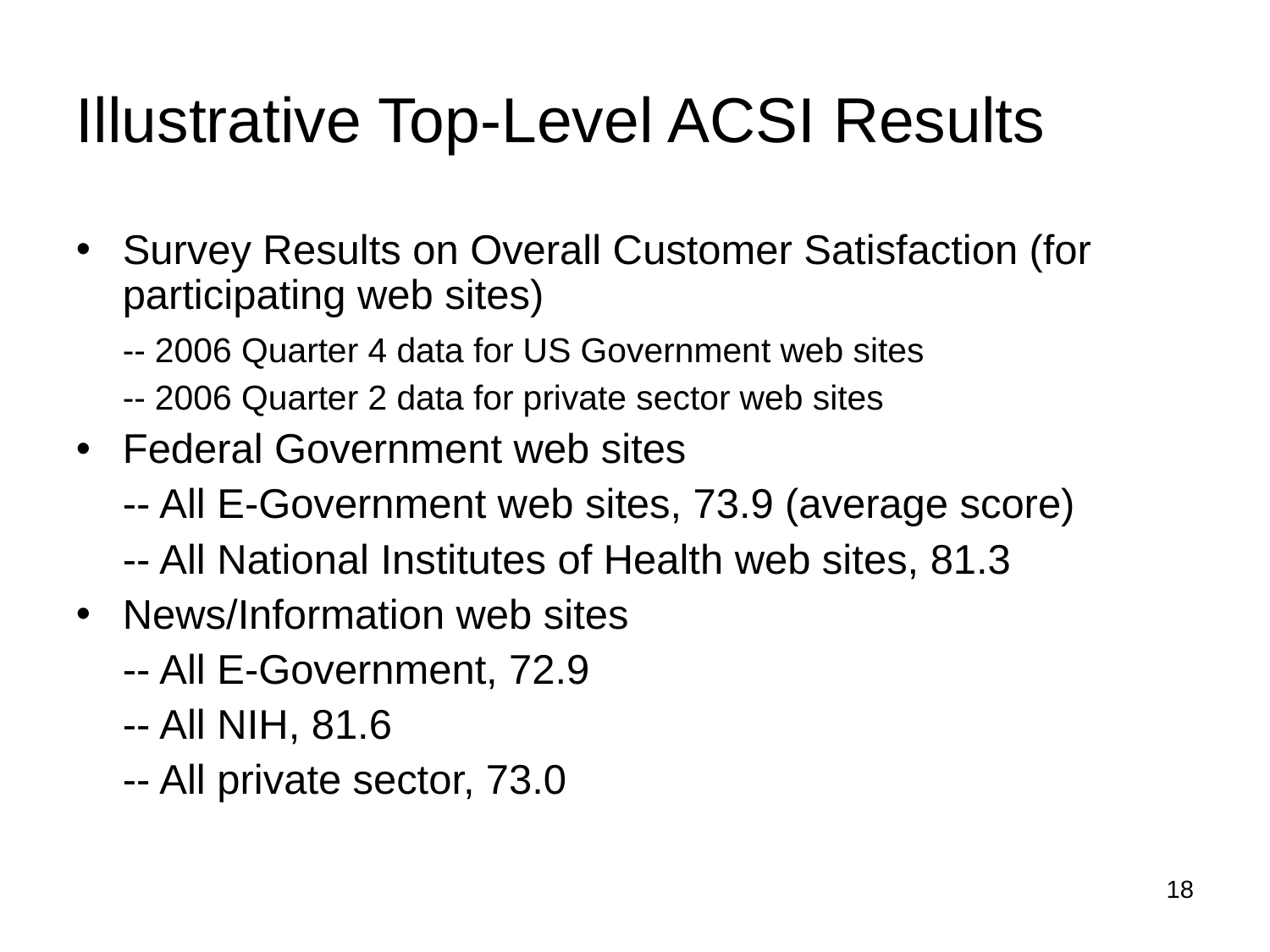

# Illustrative Top-Level ACSI Results
Survey Results on Overall Customer Satisfaction (for participating web sites)
	-- 2006 Quarter 4 data for US Government web sites
	-- 2006 Quarter 2 data for private sector web sites
Federal Government web sites
	-- All E-Government web sites, 73.9 (average score)
	-- All National Institutes of Health web sites, 81.3
News/Information web sites
	-- All E-Government, 72.9
	-- All NIH, 81.6
	-- All private sector, 73.0
18

## Slide 19
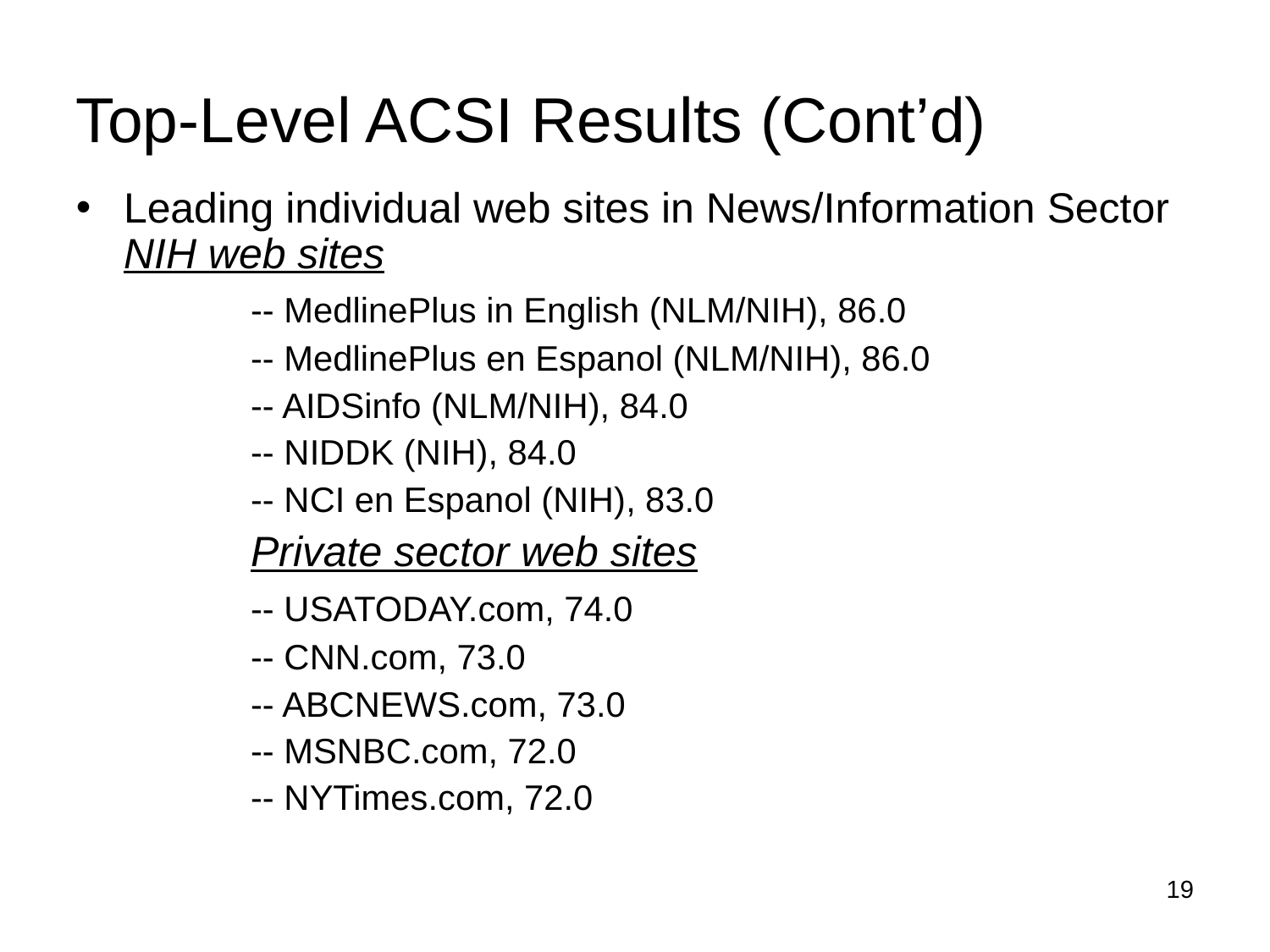

# Top-Level ACSI Results (Cont’d)
Leading individual web sites in News/Information Sector NIH web sites
	-- MedlinePlus in English (NLM/NIH), 86.0
	-- MedlinePlus en Espanol (NLM/NIH), 86.0
	-- AIDSinfo (NLM/NIH), 84.0
	-- NIDDK (NIH), 84.0
	-- NCI en Espanol (NIH), 83.0
	Private sector web sites
	-- USATODAY.com, 74.0
	-- CNN.com, 73.0
	-- ABCNEWS.com, 73.0
	-- MSNBC.com, 72.0
	-- NYTimes.com, 72.0
19

## Slide 20
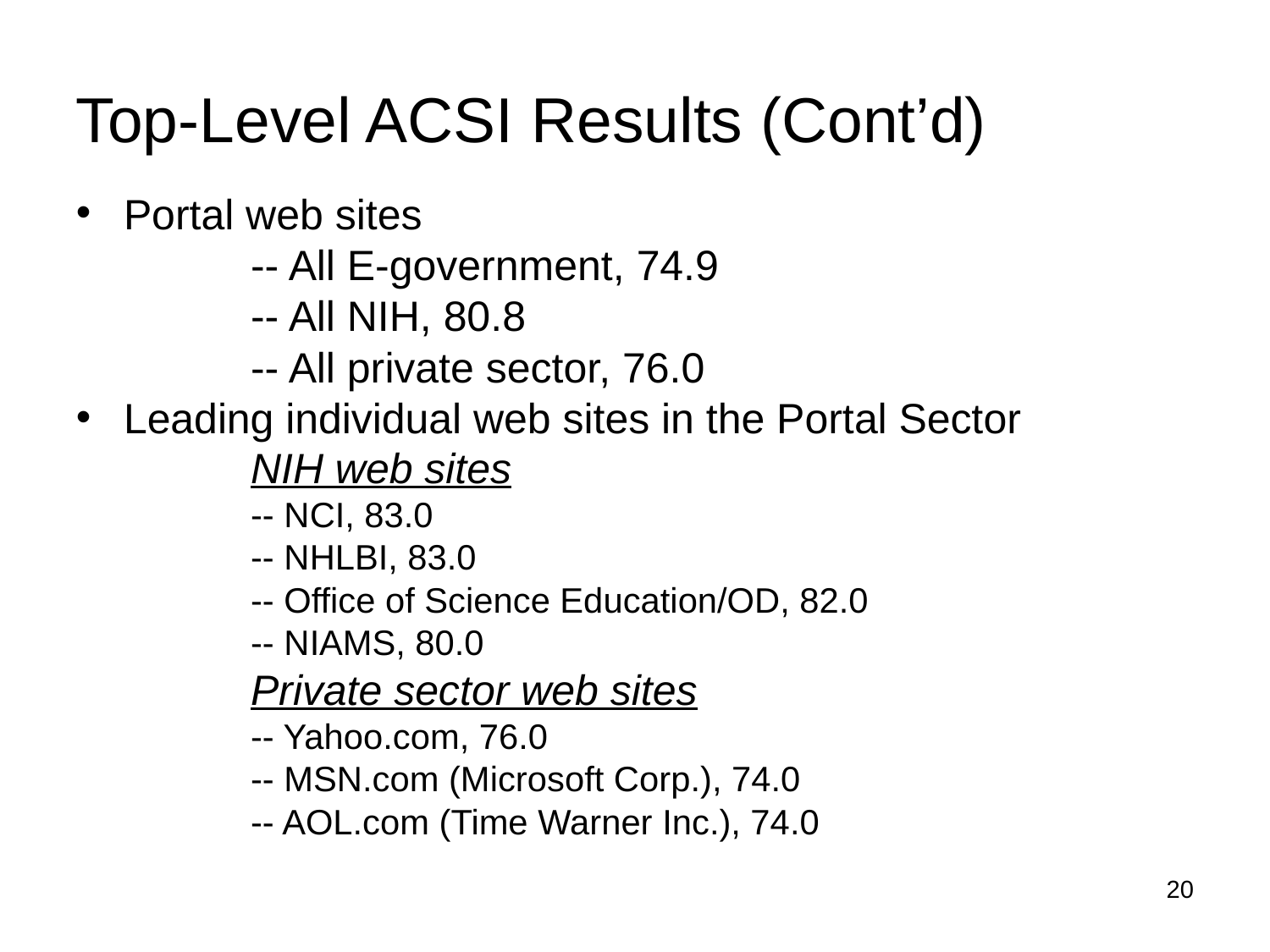

# Top-Level ACSI Results (Cont’d)
Portal web sites
	-- All E-government, 74.9
	-- All NIH, 80.8
	-- All private sector, 76.0
Leading individual web sites in the Portal Sector
	NIH web sites
	-- NCI, 83.0
	-- NHLBI, 83.0
	-- Office of Science Education/OD, 82.0
	-- NIAMS, 80.0
	Private sector web sites
	-- Yahoo.com, 76.0
	-- MSN.com (Microsoft Corp.), 74.0
	-- AOL.com (Time Warner Inc.), 74.0
20

## Slide 21
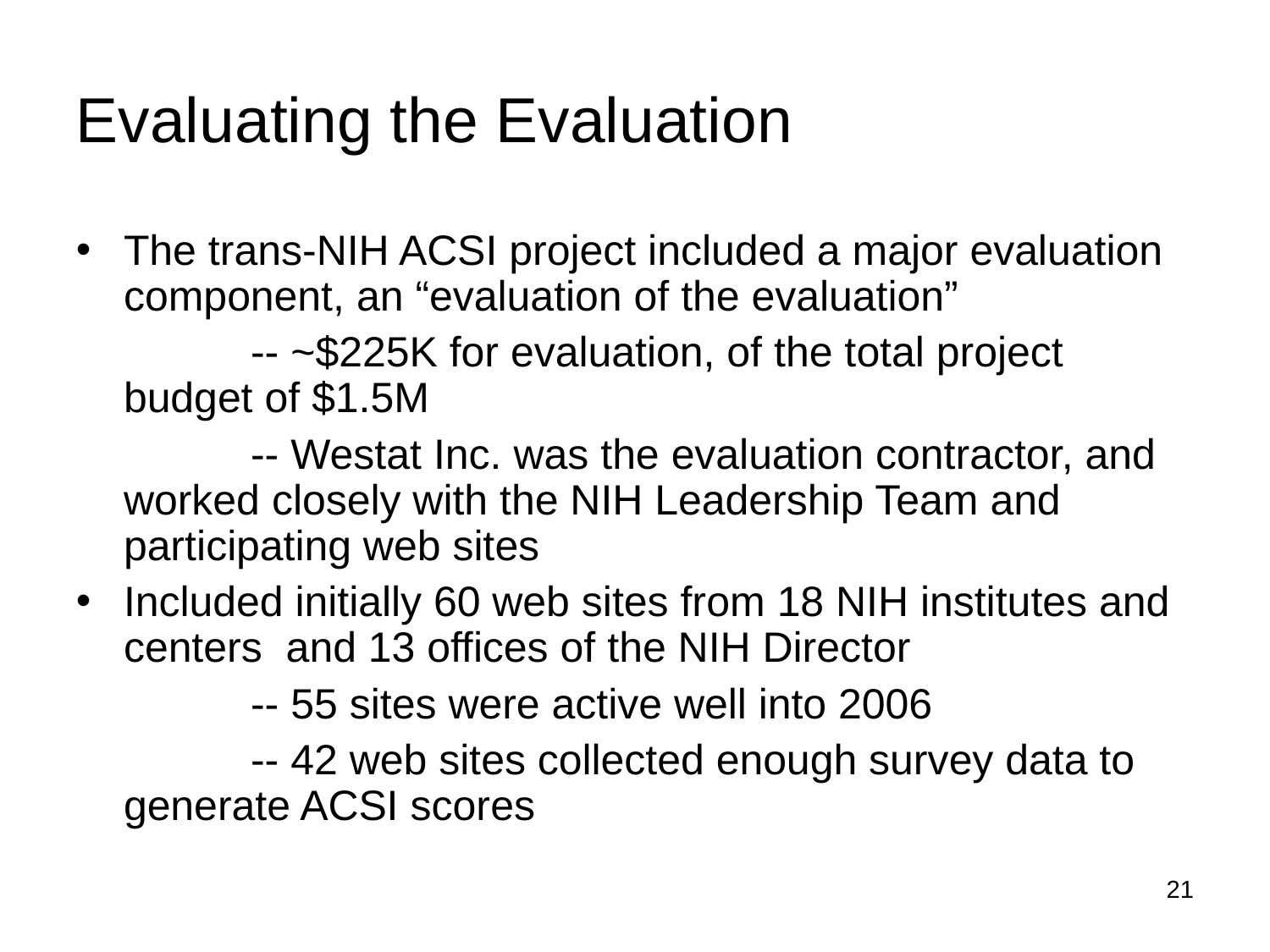

# Evaluating the Evaluation
The trans-NIH ACSI project included a major evaluation component, an “evaluation of the evaluation”
	-- ~$225K for evaluation, of the total project budget of $1.5M
	-- Westat Inc. was the evaluation contractor, and worked closely with the NIH Leadership Team and participating web sites
Included initially 60 web sites from 18 NIH institutes and centers and 13 offices of the NIH Director
	-- 55 sites were active well into 2006
	-- 42 web sites collected enough survey data to generate ACSI scores
21

## Slide 22
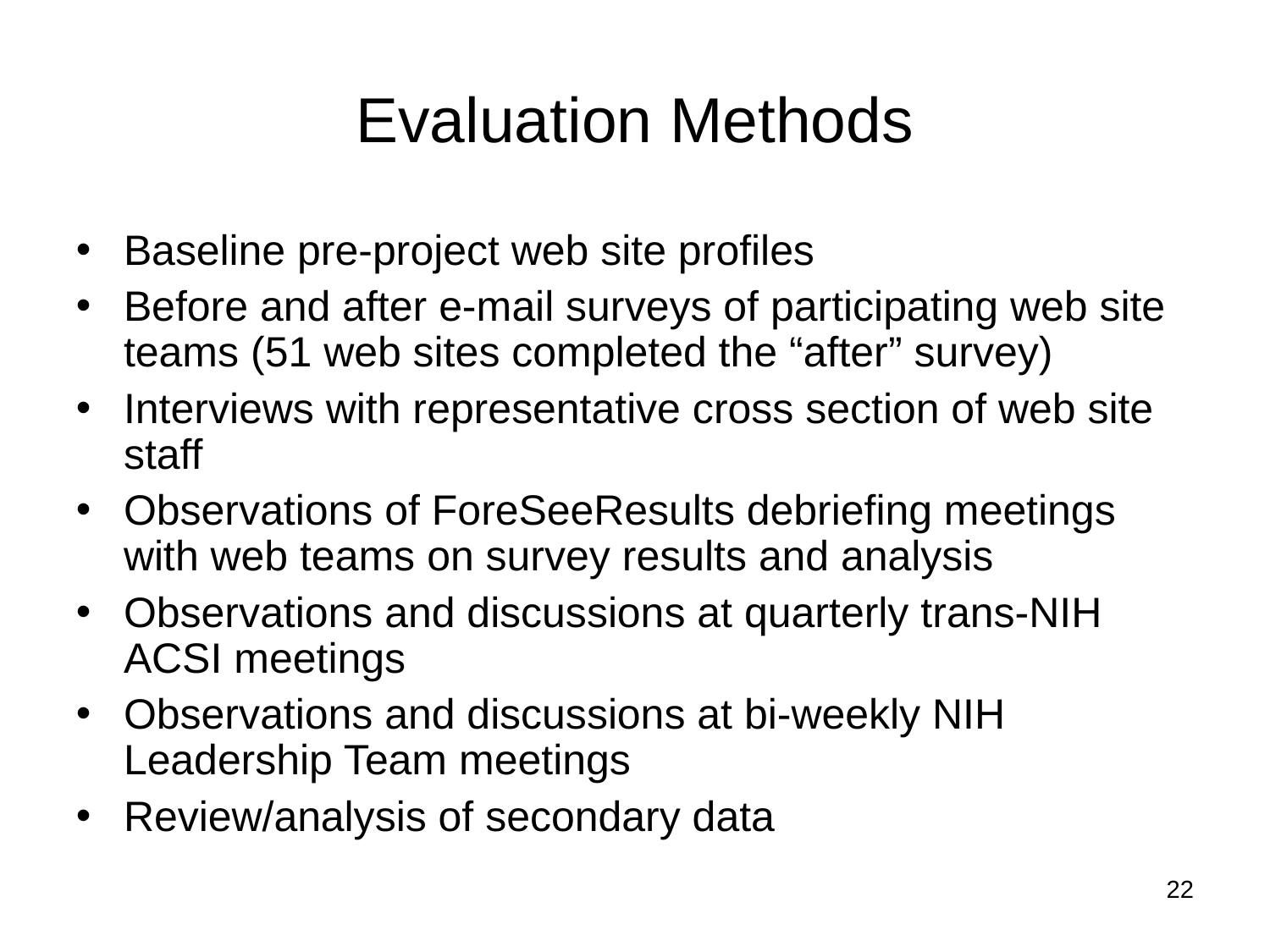

# Evaluation Methods
Baseline pre-project web site profiles
Before and after e-mail surveys of participating web site teams (51 web sites completed the “after” survey)
Interviews with representative cross section of web site staff
Observations of ForeSeeResults debriefing meetings with web teams on survey results and analysis
Observations and discussions at quarterly trans-NIH ACSI meetings
Observations and discussions at bi-weekly NIH Leadership Team meetings
Review/analysis of secondary data
22

## Slide 23
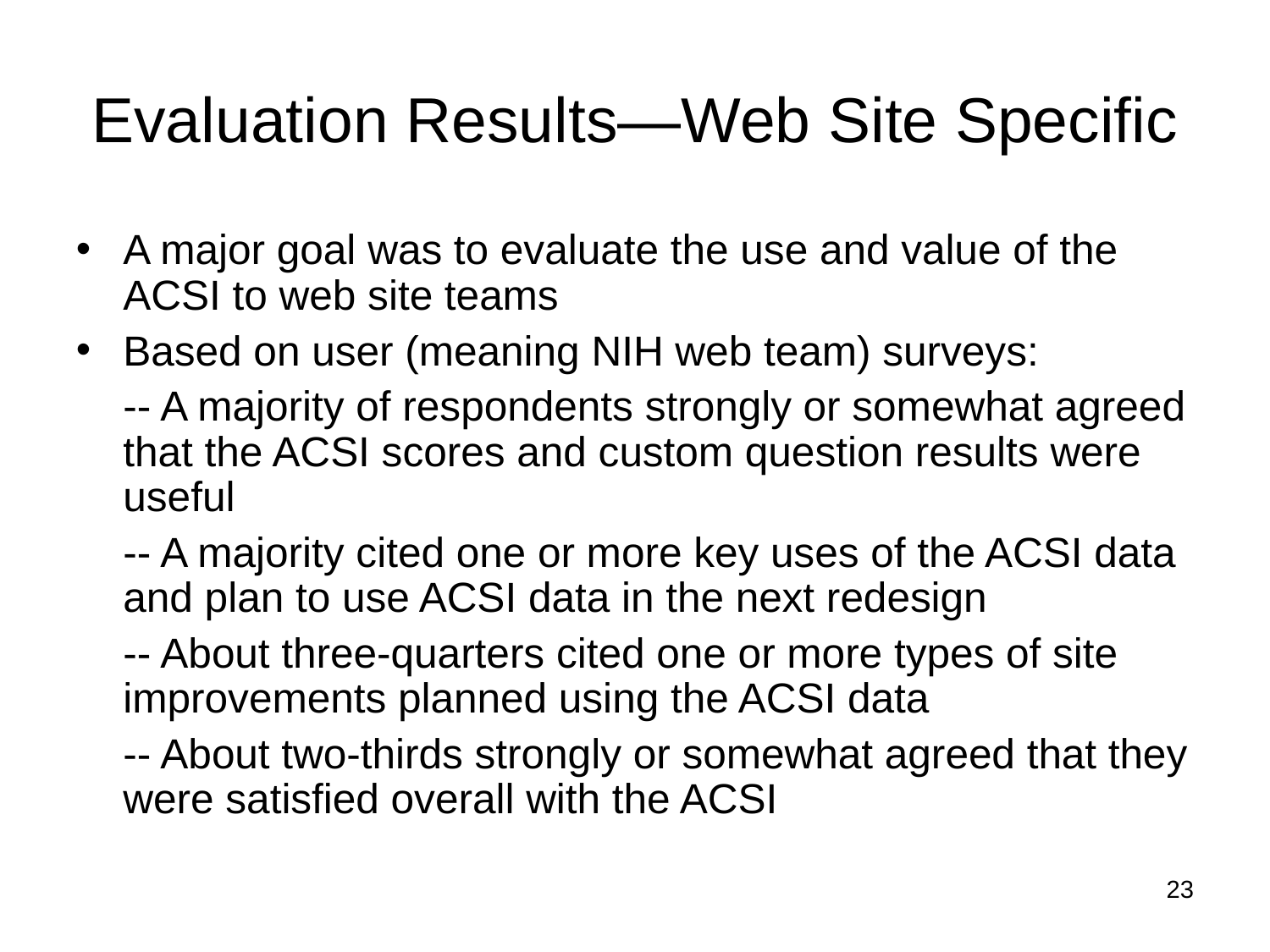

# Evaluation Results—Web Site Specific
A major goal was to evaluate the use and value of the ACSI to web site teams
Based on user (meaning NIH web team) surveys:
	-- A majority of respondents strongly or somewhat agreed that the ACSI scores and custom question results were useful
	-- A majority cited one or more key uses of the ACSI data and plan to use ACSI data in the next redesign
	-- About three-quarters cited one or more types of site improvements planned using the ACSI data
	-- About two-thirds strongly or somewhat agreed that they were satisfied overall with the ACSI
23

## Slide 24
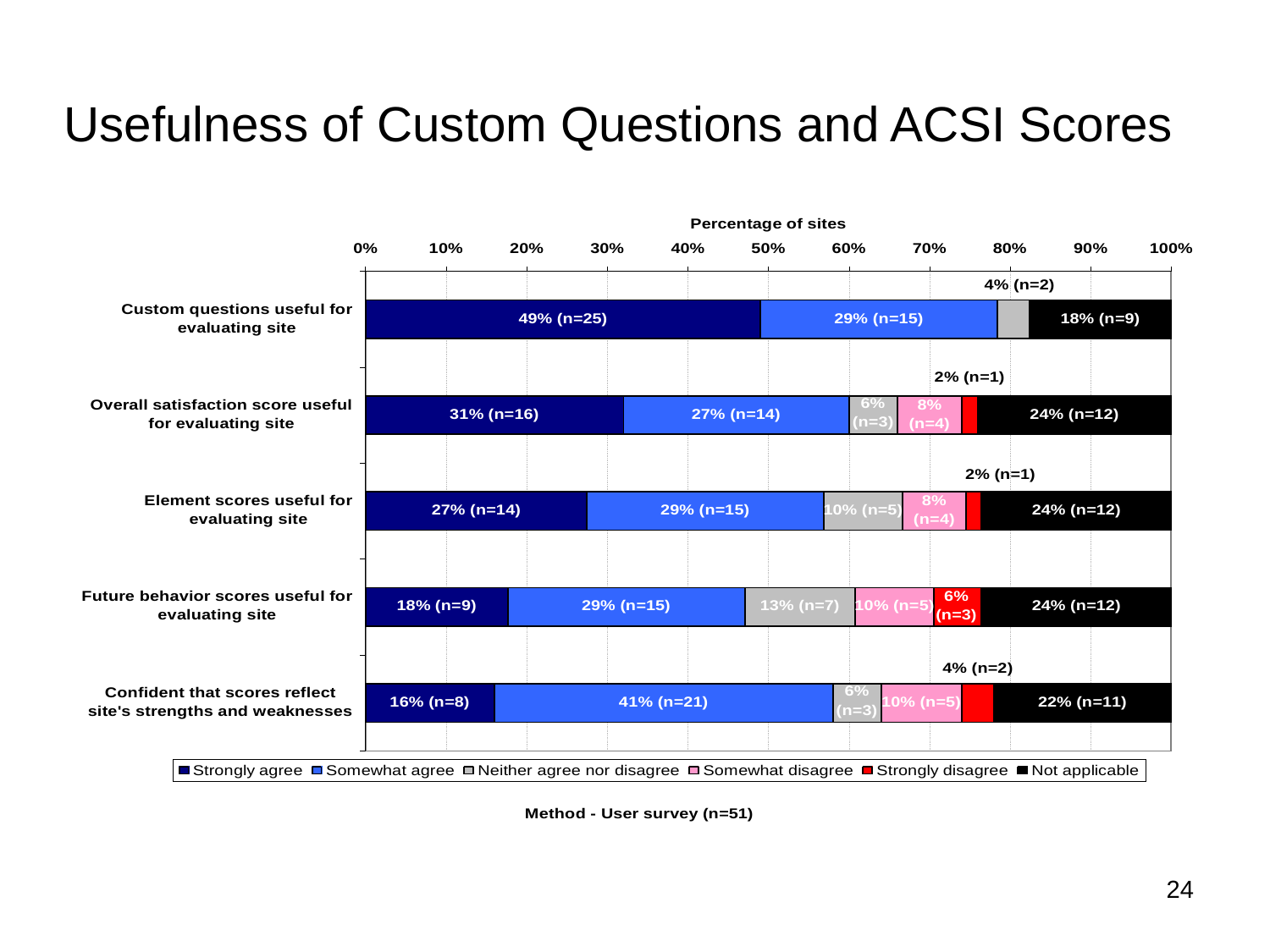

# Usefulness of Custom Questions and ACSI Scores
24

## Slide 25
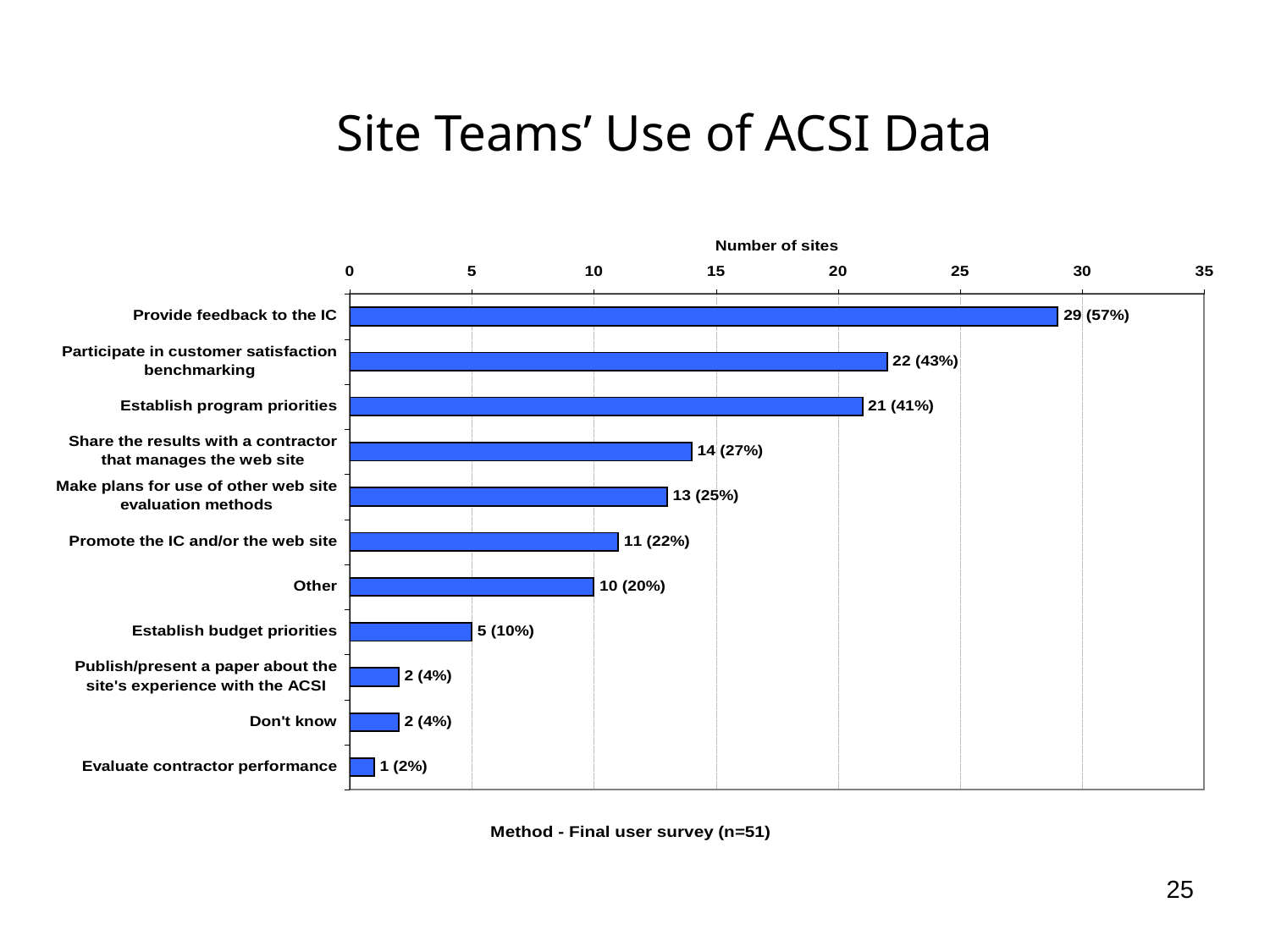

Site Teams’ Use of ACSI Data
25

## Slide 26
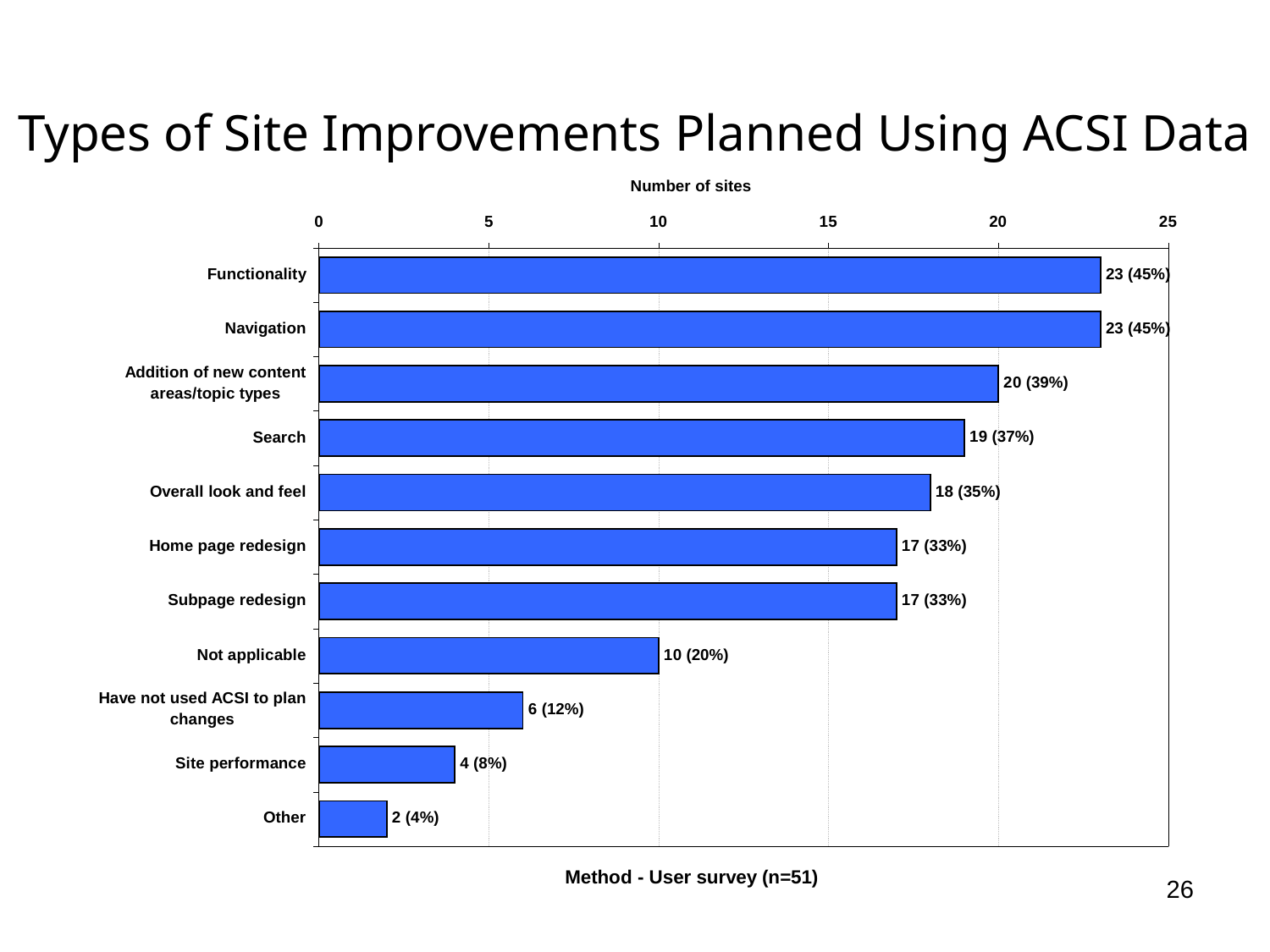

Types of Site Improvements Planned Using ACSI Data
26

## Slide 27
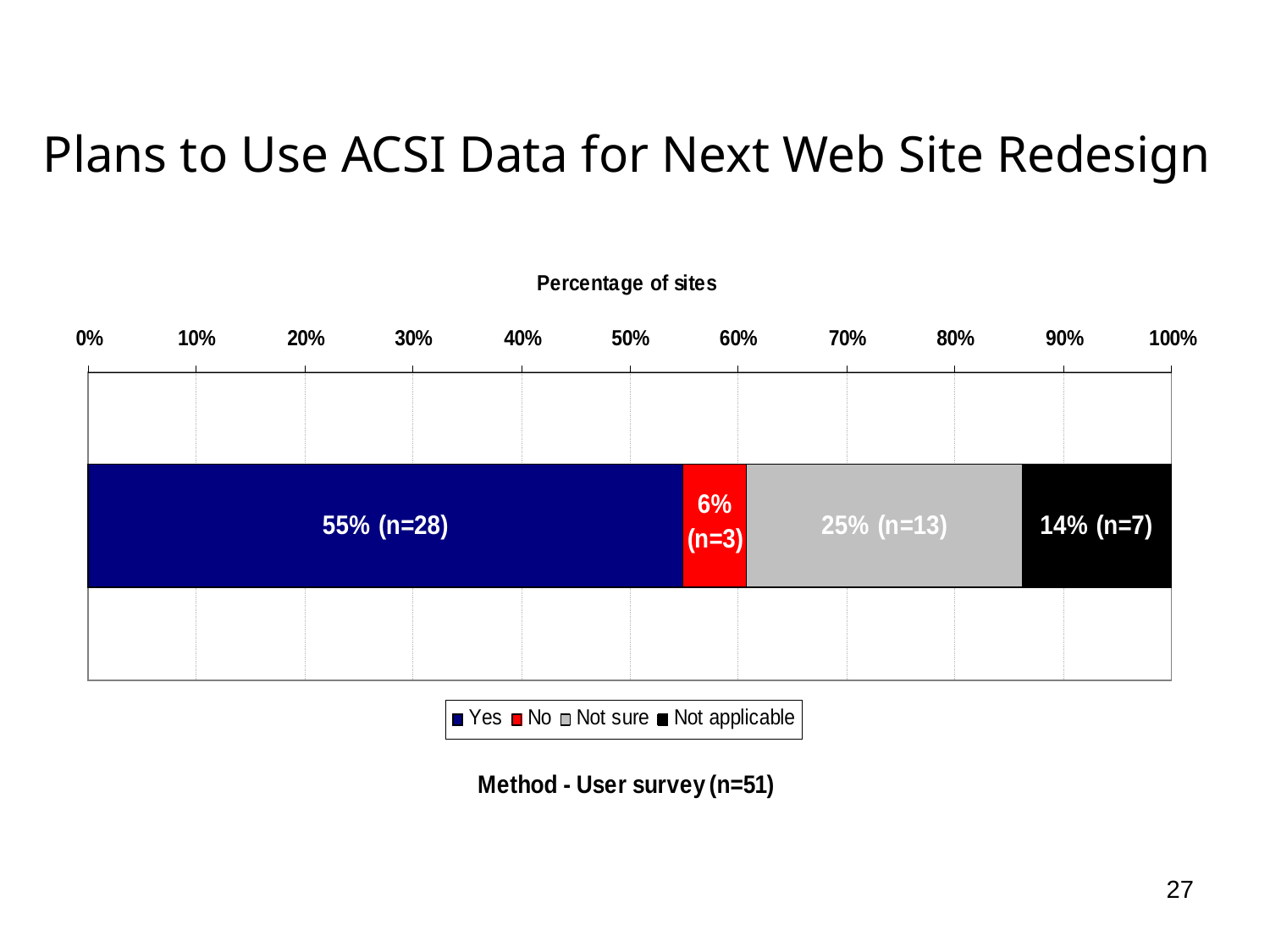

Plans to Use ACSI Data for Next Web Site Redesign
27

## Slide 28
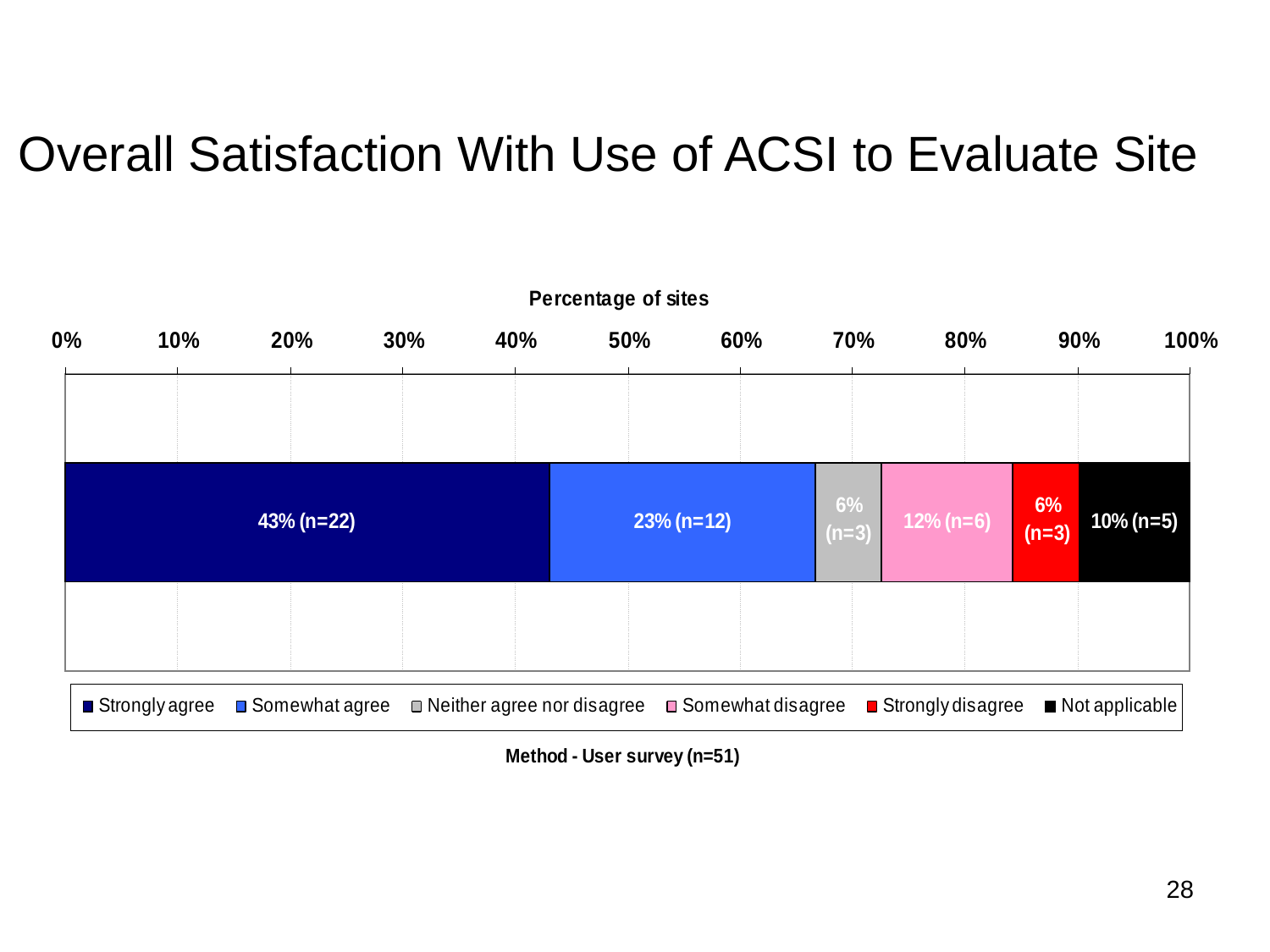

# Overall Satisfaction With Use of ACSI to Evaluate Site
28

## Slide 29
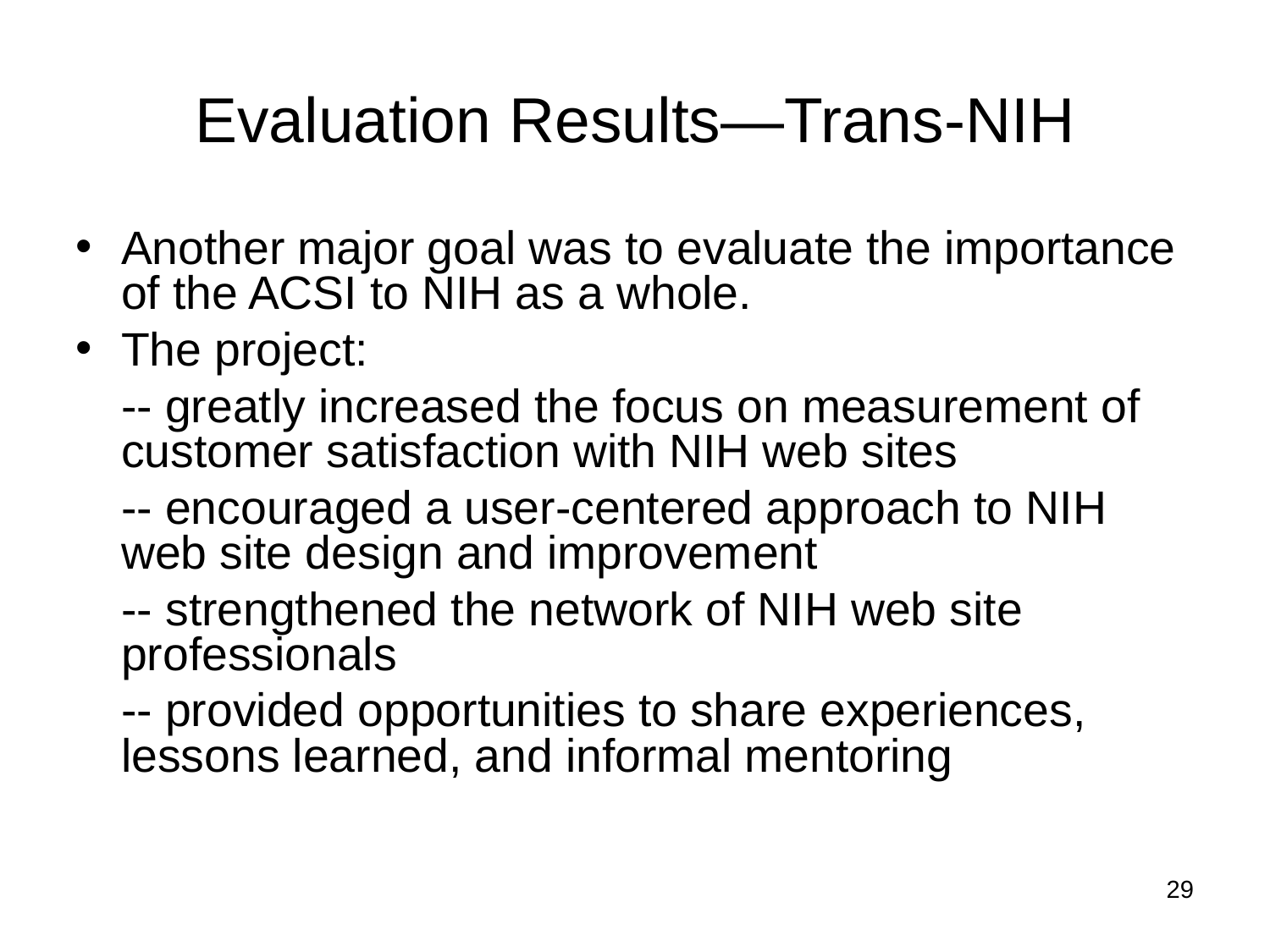

# Evaluation Results—Trans-NIH
Another major goal was to evaluate the importance of the ACSI to NIH as a whole.
The project:
	-- greatly increased the focus on measurement of customer satisfaction with NIH web sites
	-- encouraged a user-centered approach to NIH web site design and improvement
	-- strengthened the network of NIH web site professionals
	-- provided opportunities to share experiences, lessons learned, and informal mentoring
29

## Slide 30
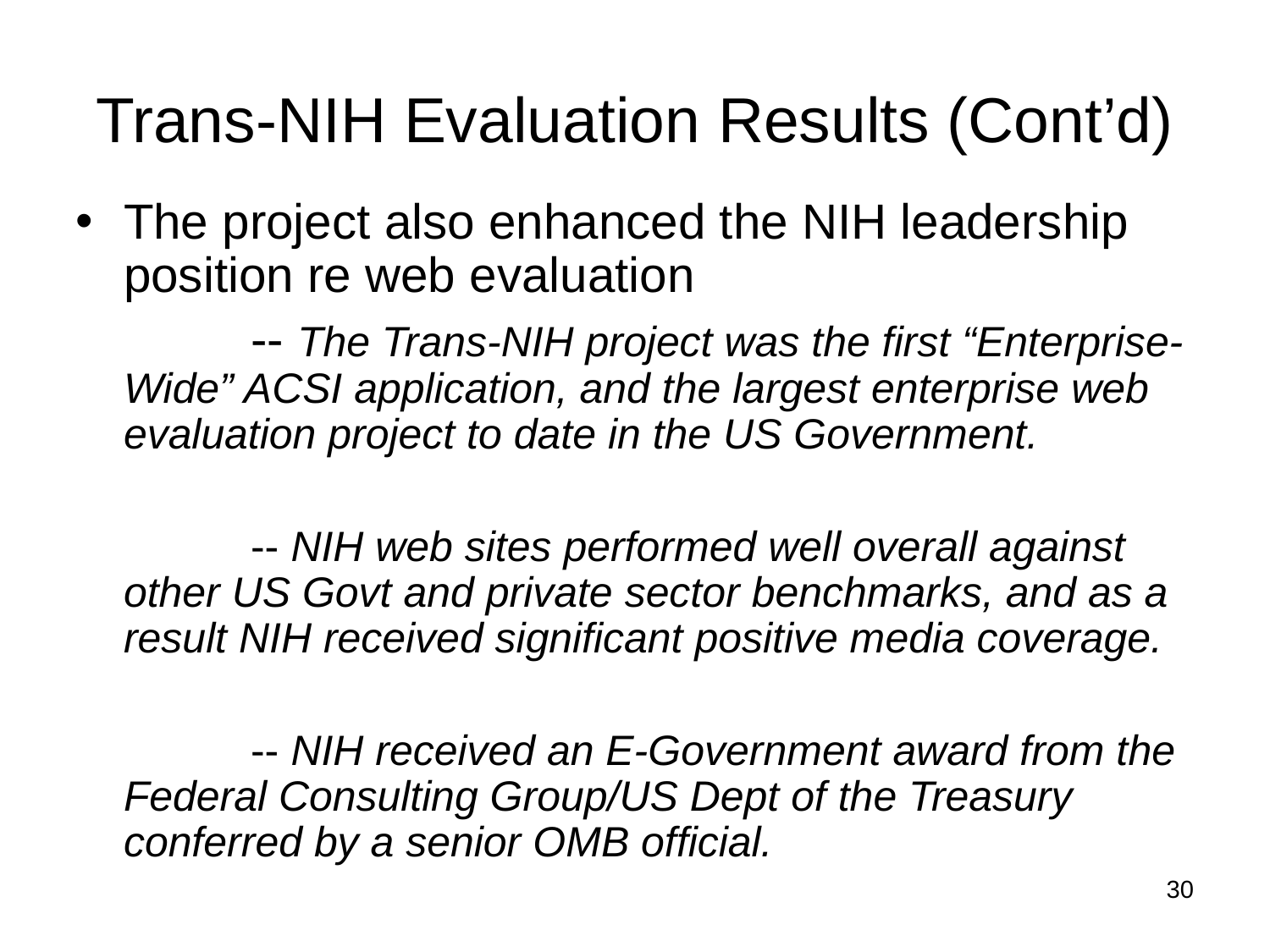

# Trans-NIH Evaluation Results (Cont’d)
The project also enhanced the NIH leadership position re web evaluation
	-- The Trans-NIH project was the first “Enterprise-Wide” ACSI application, and the largest enterprise web evaluation project to date in the US Government.
	-- NIH web sites performed well overall against other US Govt and private sector benchmarks, and as a result NIH received significant positive media coverage.
	-- NIH received an E-Government award from the Federal Consulting Group/US Dept of the Treasury conferred by a senior OMB official.
30

## Slide 31
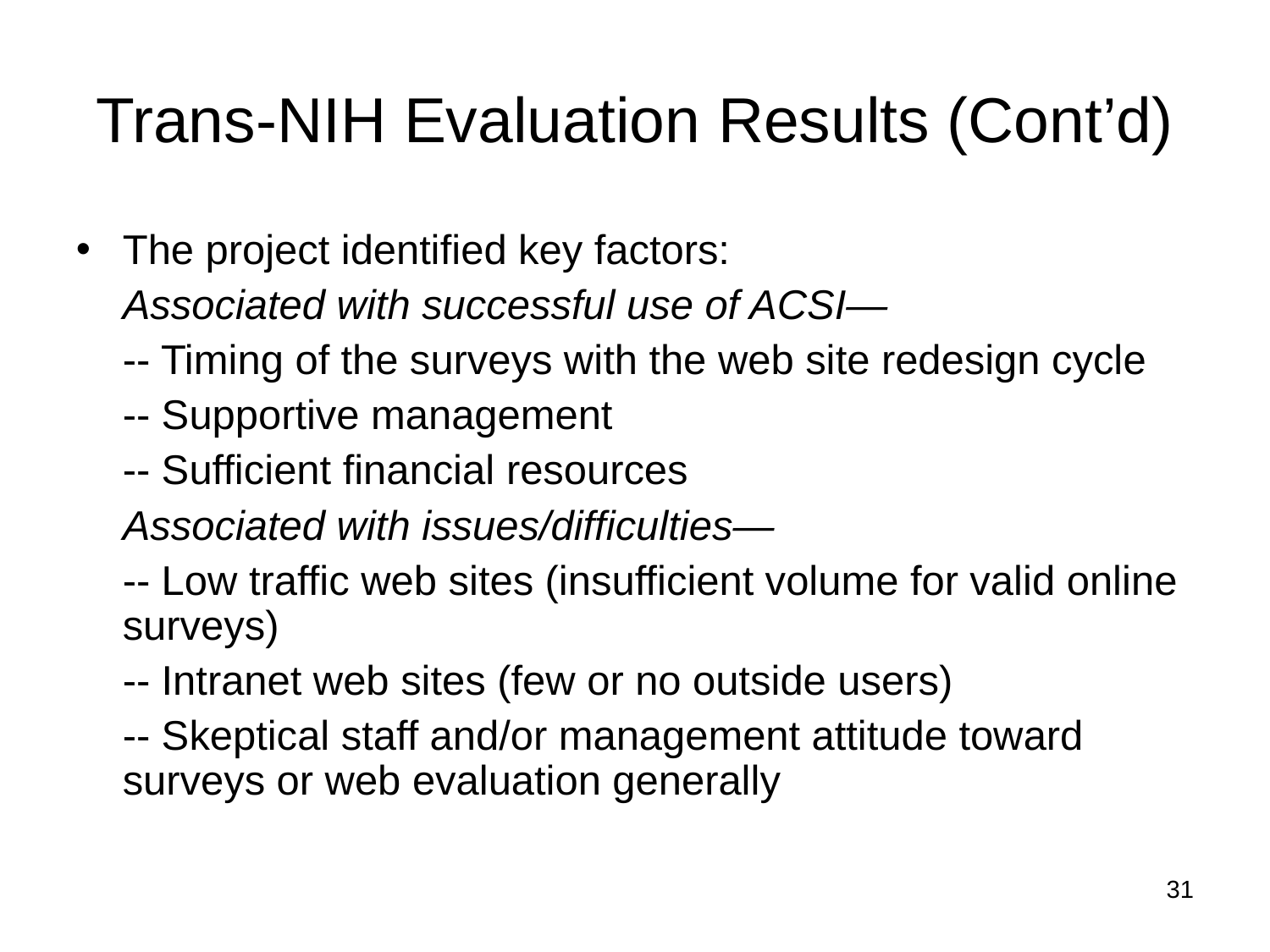

# Trans-NIH Evaluation Results (Cont’d)
The project identified key factors:
	Associated with successful use of ACSI—
	-- Timing of the surveys with the web site redesign cycle
	-- Supportive management
	-- Sufficient financial resources
	Associated with issues/difficulties—
	-- Low traffic web sites (insufficient volume for valid online surveys)
	-- Intranet web sites (few or no outside users)
	-- Skeptical staff and/or management attitude toward surveys or web evaluation generally
31

## Slide 32
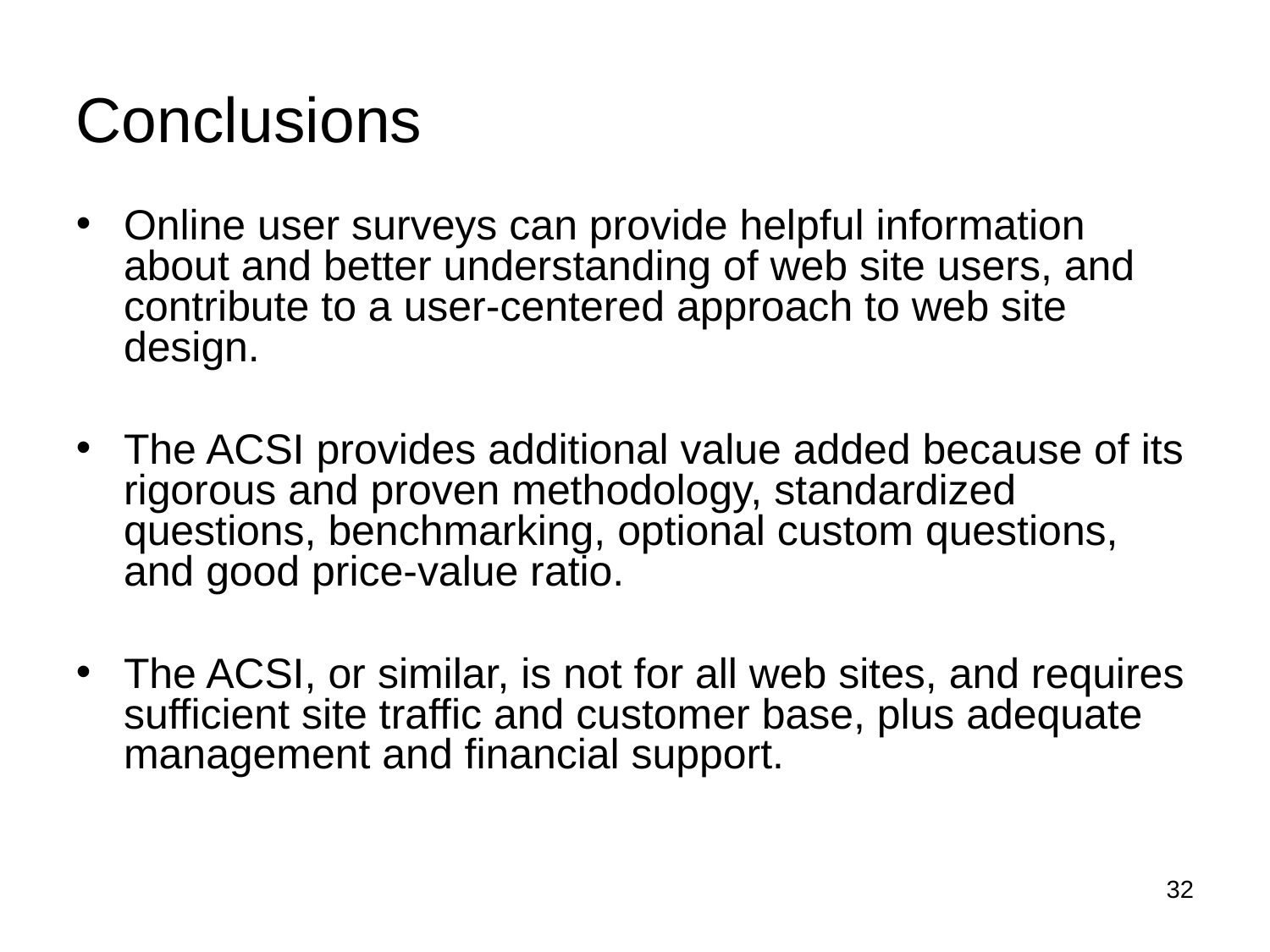

# Conclusions
Online user surveys can provide helpful information about and better understanding of web site users, and contribute to a user-centered approach to web site design.
The ACSI provides additional value added because of its rigorous and proven methodology, standardized questions, benchmarking, optional custom questions, and good price-value ratio.
The ACSI, or similar, is not for all web sites, and requires sufficient site traffic and customer base, plus adequate management and financial support.
32

## Slide 33
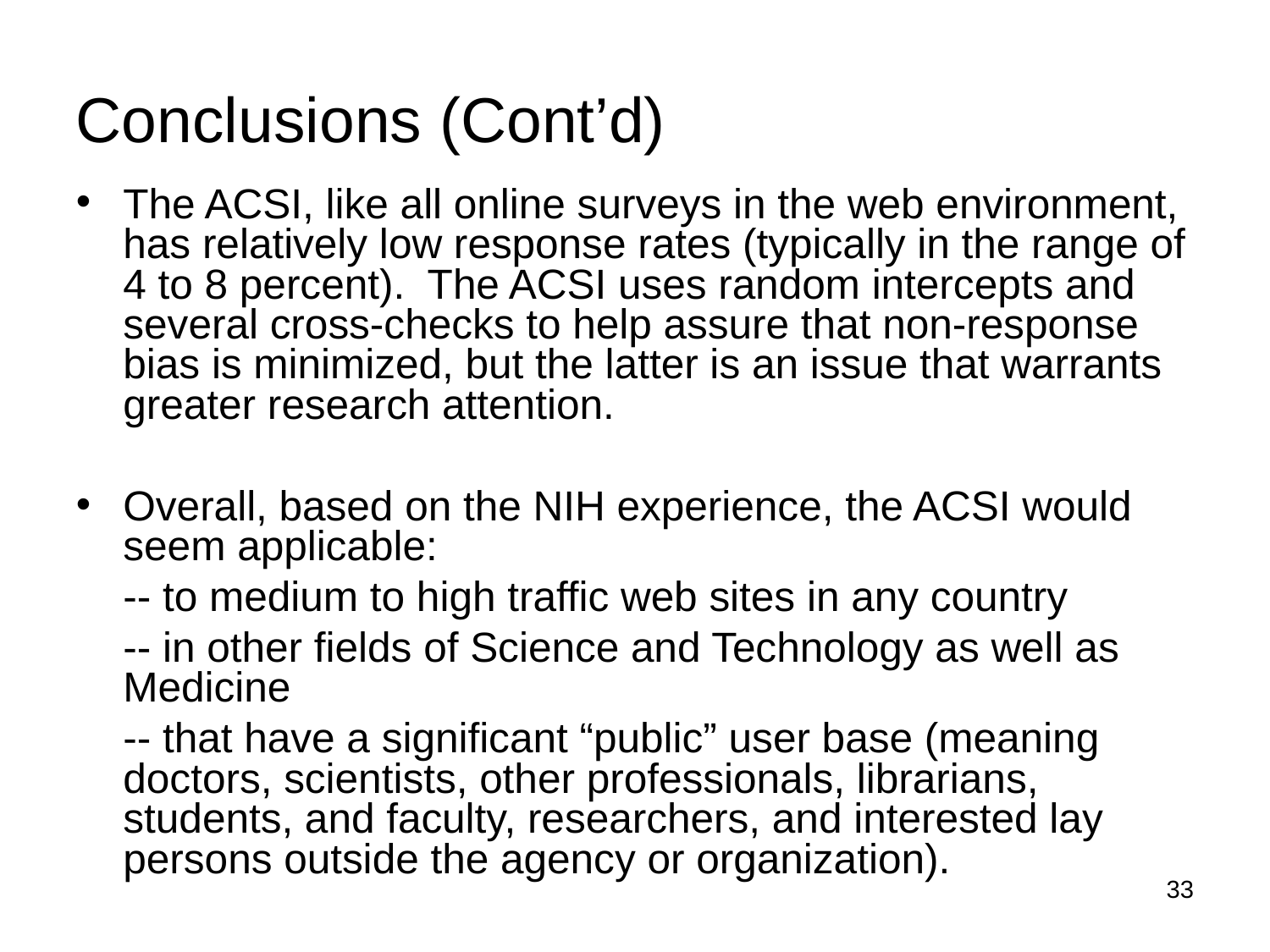

# Conclusions (Cont’d)
The ACSI, like all online surveys in the web environment, has relatively low response rates (typically in the range of 4 to 8 percent). The ACSI uses random intercepts and several cross-checks to help assure that non-response bias is minimized, but the latter is an issue that warrants greater research attention.
Overall, based on the NIH experience, the ACSI would seem applicable:
	-- to medium to high traffic web sites in any country
	-- in other fields of Science and Technology as well as Medicine
	-- that have a significant “public” user base (meaning doctors, scientists, other professionals, librarians, students, and faculty, researchers, and interested lay persons outside the agency or organization).
33

## Slide 34
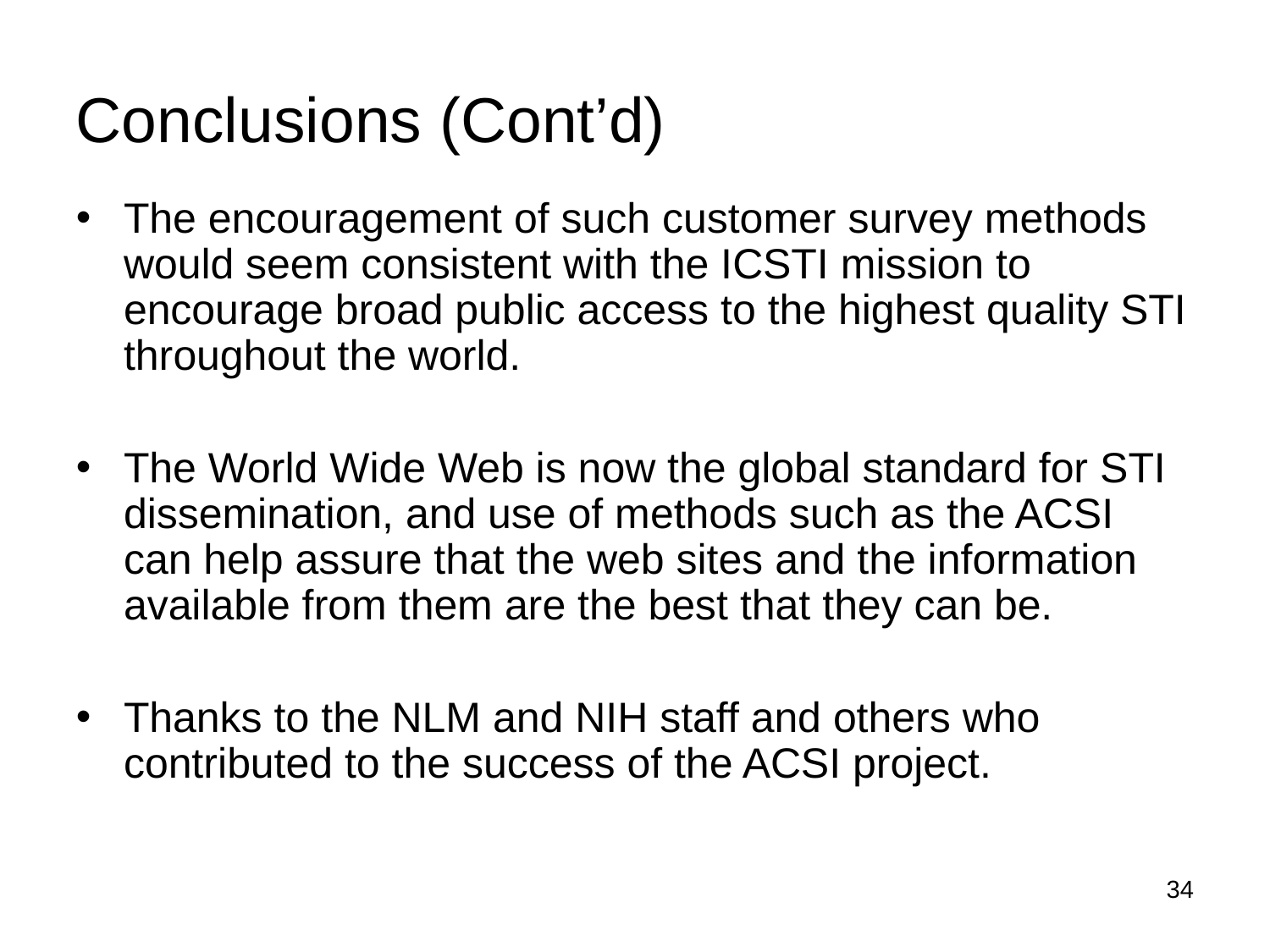

# Conclusions (Cont’d)
The encouragement of such customer survey methods would seem consistent with the ICSTI mission to encourage broad public access to the highest quality STI throughout the world.
The World Wide Web is now the global standard for STI dissemination, and use of methods such as the ACSI can help assure that the web sites and the information available from them are the best that they can be.
Thanks to the NLM and NIH staff and others who contributed to the success of the ACSI project.
34

## Slide 35
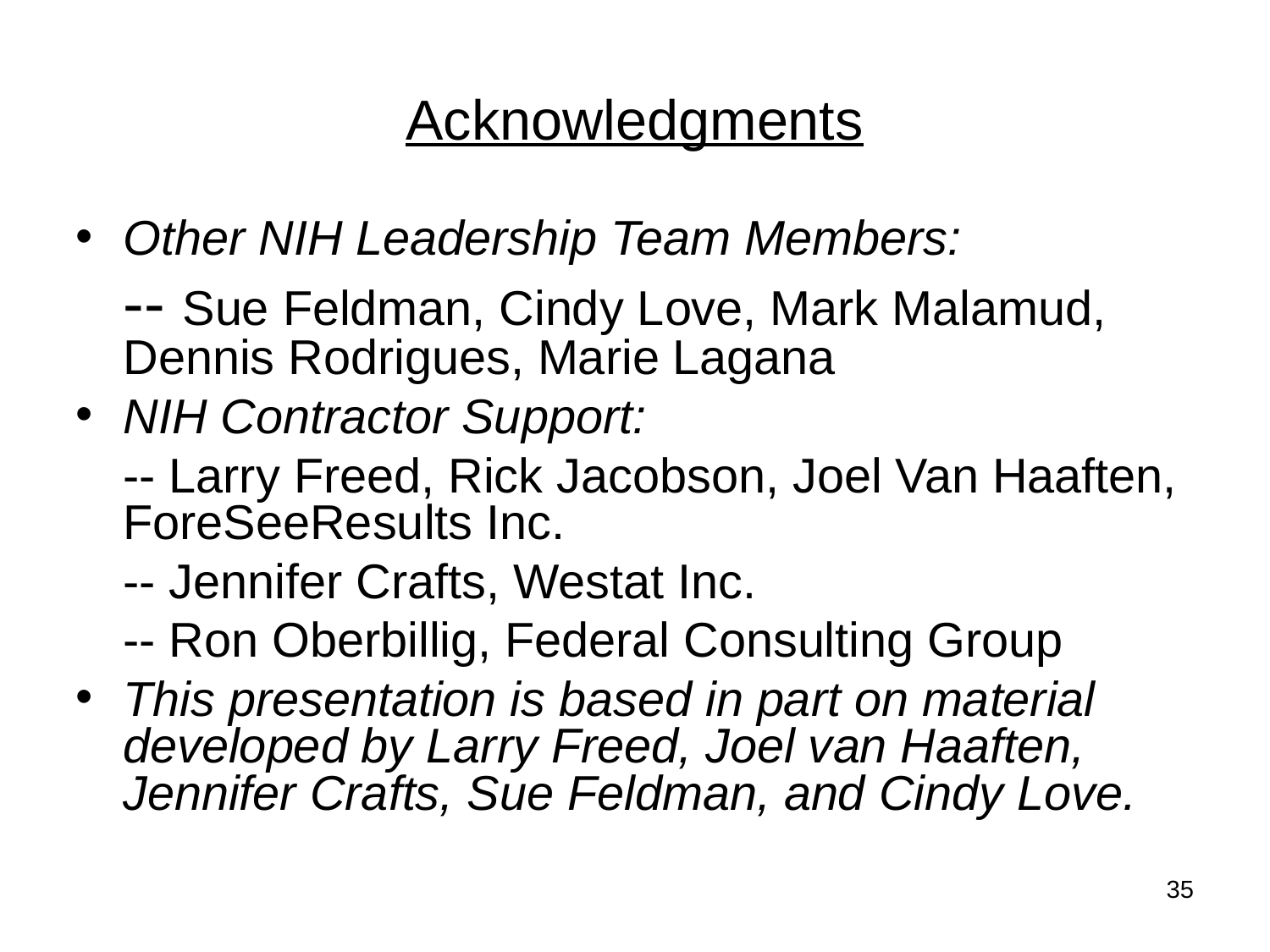

# Acknowledgments
Other NIH Leadership Team Members:
	-- Sue Feldman, Cindy Love, Mark Malamud, Dennis Rodrigues, Marie Lagana
NIH Contractor Support:
	-- Larry Freed, Rick Jacobson, Joel Van Haaften, ForeSeeResults Inc.
	-- Jennifer Crafts, Westat Inc.
	-- Ron Oberbillig, Federal Consulting Group
This presentation is based in part on material developed by Larry Freed, Joel van Haaften, Jennifer Crafts, Sue Feldman, and Cindy Love.
35

## Slide 36
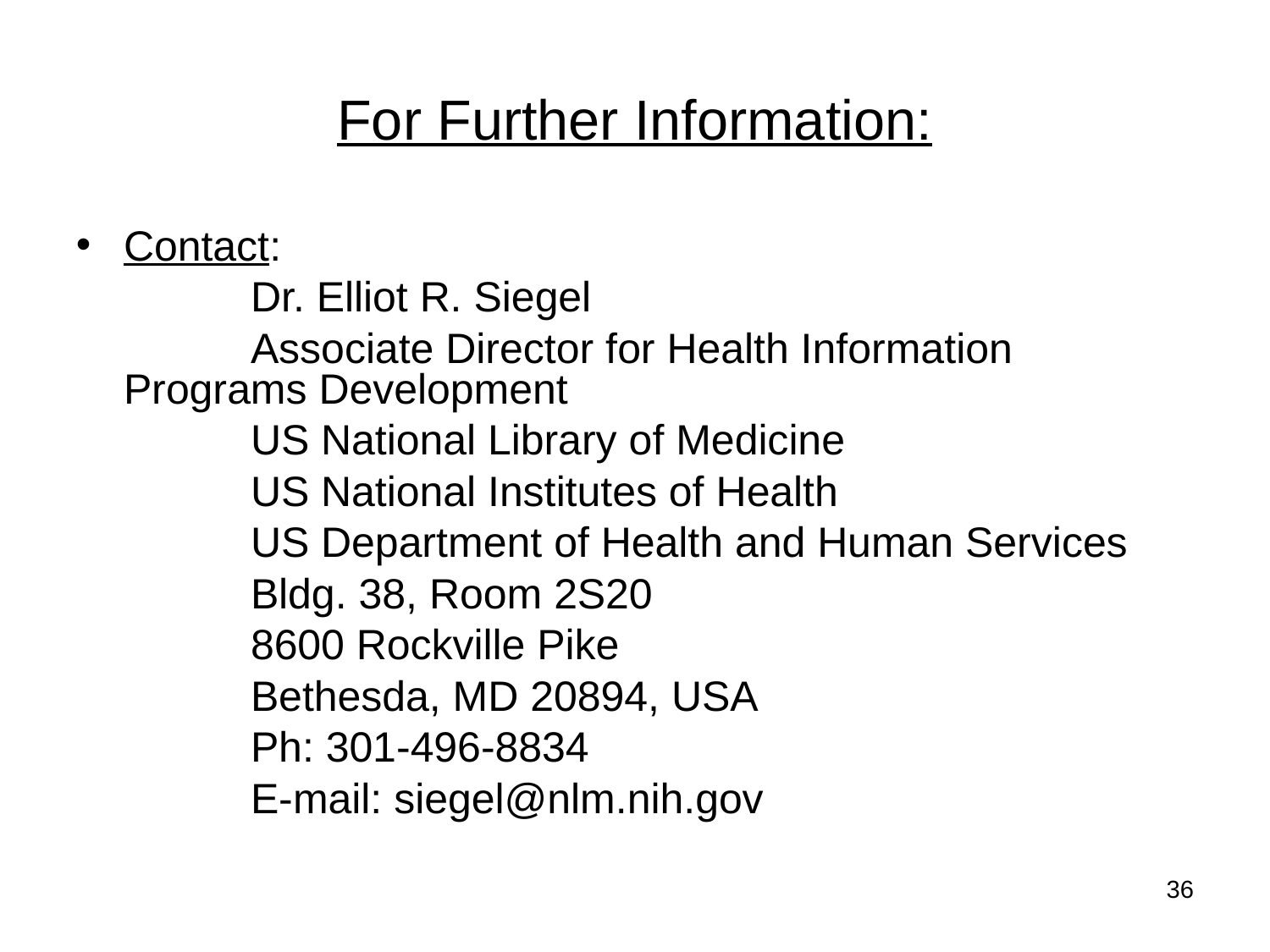

# For Further Information:
Contact:
	Dr. Elliot R. Siegel
	Associate Director for Health Information Programs Development
	US National Library of Medicine
	US National Institutes of Health
	US Department of Health and Human Services
	Bldg. 38, Room 2S20
	8600 Rockville Pike
	Bethesda, MD 20894, USA
	Ph: 301-496-8834
	E-mail: siegel@nlm.nih.gov
36
