# Supplementary material for: Web Evaluation at the US National Institutes of Health: Use of the American Customer Satisfaction Index Online Customer Survey
Source: J Med Internet Res. 2008 Feb 15;10(1):e4. doi: 10.2196/jmir.944 (PMC2483849; doi:10.2196/jmir.944)
Supplement: Supplementary file 3 [file jmir_v10i1e4_app3.pdf]

Multimedia Appendix 3
Wood FB, Siegel ER, Feldman S, Love CB, Rodrigues D, Malamud M, Lagana M, Crafts J
Web Evaluation at the US National Institutes of Health: Use of the American Customer Satisfaction Index Online Customer Survey
J Med Internet Res 2008;10(1):e4
<URL: http://www.jmir.org/2008/1/e4/>

American Customer Satisfaction Index E-Government Satisfaction Index Quarterly Reports, With Commentary and Analysis, by Larry Freed, ForeSee Results Inc.

Freed, L. (2003). American Customer Satisfaction Index E-Government Satisfaction Index September 15, 2003 Commentary and Analysis. (This was the first in the series of reports on e-government customer satisfaction.) http://www.fcg.gov/pdf/e-gov_comm_092003.pdf
Archived at: http://www.webcitation.org/5ODsndOtY

Freed, L. (2003). American Customer Satisfaction Index E-Government Satisfaction Index December 15, 2003 Commentary and Analysis. http://www.fcg.gov/pdf/e-gov_comm_122003.pdf.
Archived at: http://www.webcitation.org/5O6kzzerV

Freed, L. (2004). American Customer Satisfaction Index E-Government Satisfaction Index March 15, 2004 Commentary and Analysis. http://www.fcg.gov/pdf/e-gov_comm_032004.pdf.
Archived at: http://www.webcitation.org/5O6kzzerL

Freed, L. (2004). American Customer Satisfaction Index E-Government Satisfaction Index June 21, 2004 Commentary and Analysis. http://www.fcg.gov/pdf/e-gov_comm_062004.pdf.
Archived at: http://www.webcitation.org/5O6kzzerC

Freed, L. (2004). American Customer Satisfaction Index E-Government Satisfaction Index September 21, 2004 Commentary and Analysis. http://www.fcg.gov/pdf/e-gov_comm_092004.pdf.
Archived at: http://www.webcitation.org/5O6kzzer3

Freed, L. (2004). American Customer Satisfaction Index E-Government Satisfaction Index December 14, 2004 Commentary and Analysis. http://www.fcg.gov/pdf/e-gov_comm_122004.pdf.
Archived at: http://www.webcitation.org/5O6kzzeqs

Freed, L. (2005). American Customer Satisfaction Index E-Government Satisfaction Index December 15, 2005 Commentary and Analysis. http://www.fcg.gov/pdf/e-gov_comm_122005.pdf.
Archived at: http://www.webcitation.org/5O6kzzeqF

Freed, L. (2005). American Customer Satisfaction Index E-Government Satisfaction Index September 20, 2005 Commentary and Analysis. http://www.fcg.gov/pdf/e-gov_comm_092005.pdf. Archived at: http://www.webcitation.org/5O6kzzeqP

Freed, L. (2005). American Customer Satisfaction Index E-Government Satisfaction Index June 14, 2005 Commentary and Analysis. http://www.fcg.gov/pdf/e-gov_comm_062005.pdf. Archived at: http://www.webcitation.org/5O6kzzeqY

Freed, L. (2005). American Customer Satisfaction Index E-Government Satisfaction Index March 15, 2005 Commentary and Analysis. http://www.fcg.gov/pdf/e-gov_comm_0305.pdf. Archived at: http://www.webcitation.org/5O6kzzeqi

Freed, L. (2006). American Customer Satisfaction Index E-Government Satisfaction Index December 15, 2006 Commentary and Analysis. http://www.fcg.gov/documents/acsi-results-12-2006.pdf. Archived at: http://www.webcitation.org/5O6kzzepa

Freed, L. (2006). American Customer Satisfaction Index E-Government Satisfaction Index September 19, 2006 Commentary and Analysis. http://www.fcg.gov/documents/acsi-results-09-2006.pdf. Archived at: http://www.webcitation.org/5O6kzzepm

Freed, L. (2006). American Customer Satisfaction Index E-Government Satisfaction Index June 20, 2006 Commentary and Analysis. http://www.fcg.gov/documents/acsi-results-06-2006.pdf. Archived at: http://www.webcitation.org/5O6kzzepw

Freed, L. (2006). American Customer Satisfaction Index E-Government Satisfaction Index March 27, 2006 Commentary and Analysis. http://www.fcg.gov/documents/foresee_results-03-2006.pdf. Archived at: http://www.webcitation.org/5O9cAinua

Freed, L. (2007). American Customer Satisfaction Index E-Government Satisfaction Index March 20, 2007 Commentary and Analysis. http://www.fcg.gov/documents/acsi-results-03-2007.pdf. Archived at: http://www.webcitation.org/5O6kzzep9
