# Supplementary material for: Web Evaluation at the US National Institutes of Health: Use of the American Customer Satisfaction Index Online Customer Survey
Source: J Med Internet Res. 2008 Feb 15;10(1):e4. doi: 10.2196/jmir.944 (PMC2483849; doi:10.2196/jmir.944)
Supplement: Supplementary file 4 [file jmir_v10i1e4_app4.ppt]

## Slide 1
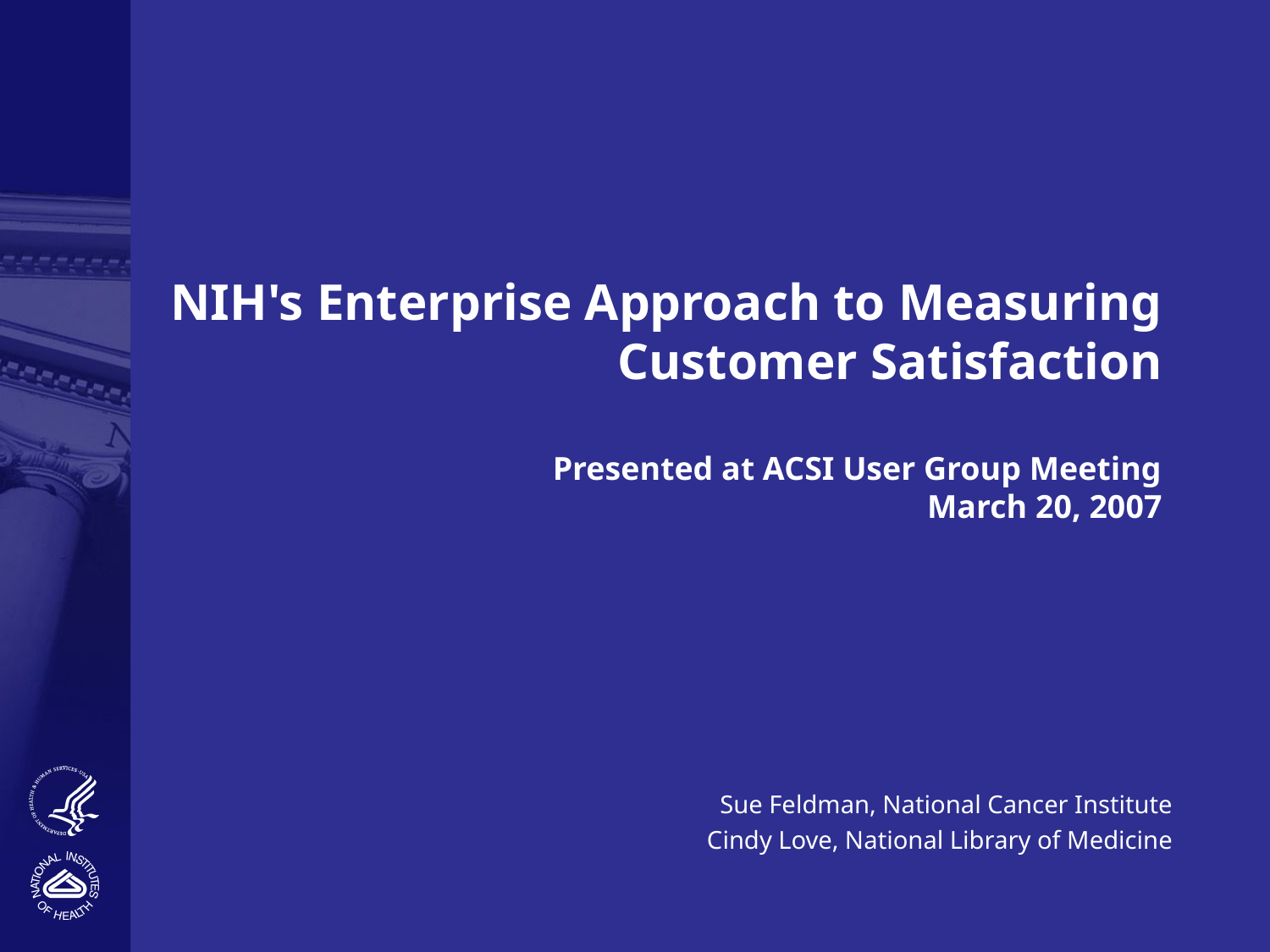

# NIH's Enterprise Approach to Measuring Customer Satisfaction Presented at ACSI User Group Meeting March 20, 2007
Sue Feldman, National Cancer Institute
Cindy Love, National Library of Medicine

## Slide 2
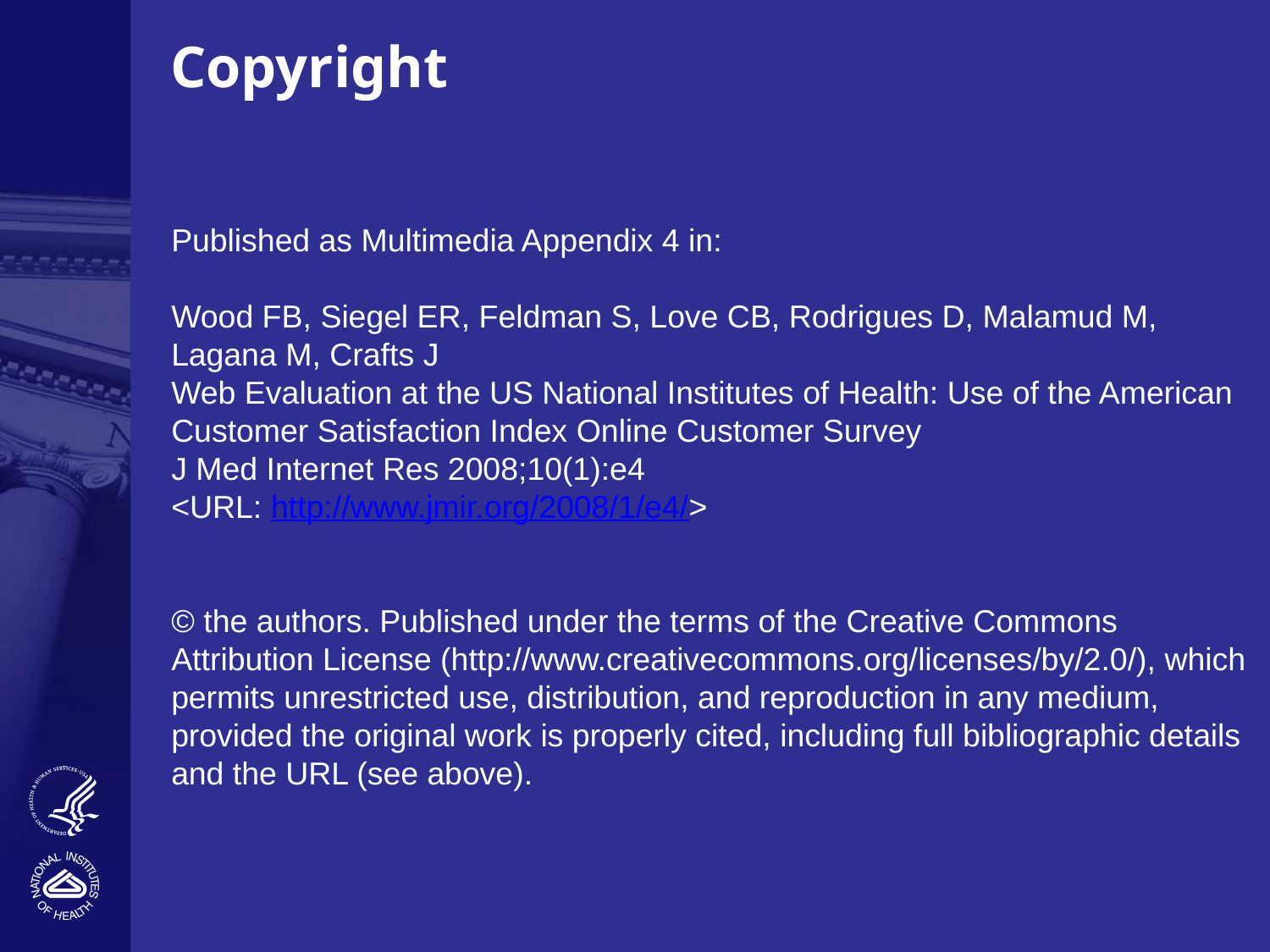

# Copyright
Published as Multimedia Appendix 4 in:
Wood FB, Siegel ER, Feldman S, Love CB, Rodrigues D, Malamud M, Lagana M, Crafts J
Web Evaluation at the US National Institutes of Health: Use of the American Customer Satisfaction Index Online Customer Survey
J Med Internet Res 2008;10(1):e4
<URL: http://www.jmir.org/2008/1/e4/>
© the authors. Published under the terms of the Creative Commons Attribution License (http://www.creativecommons.org/licenses/by/2.0/), which permits unrestricted use, distribution, and reproduction in any medium, provided the original work is properly cited, including full bibliographic details and the URL (see above).

## Slide 3
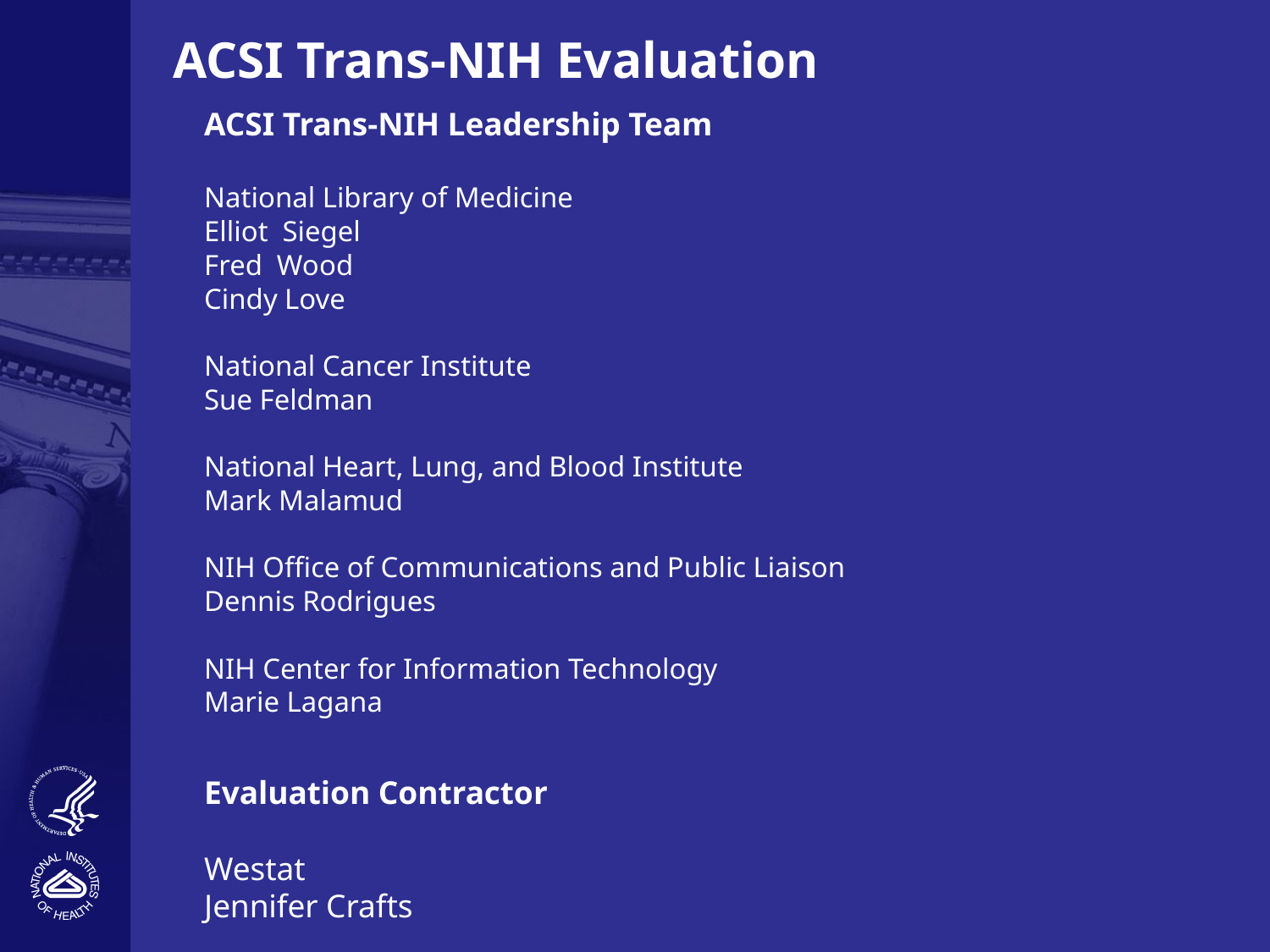

# ACSI Trans-NIH Evaluation
ACSI Trans-NIH Leadership Team
	National Library of Medicine
		Elliot Siegel
		Fred Wood
		Cindy Love
	National Cancer Institute
		Sue Feldman
	National Heart, Lung, and Blood Institute
		Mark Malamud
	NIH Office of Communications and Public Liaison
		Dennis Rodrigues
	NIH Center for Information Technology
		Marie Lagana
Evaluation Contractor
	Westat
		Jennifer Crafts

## Slide 4
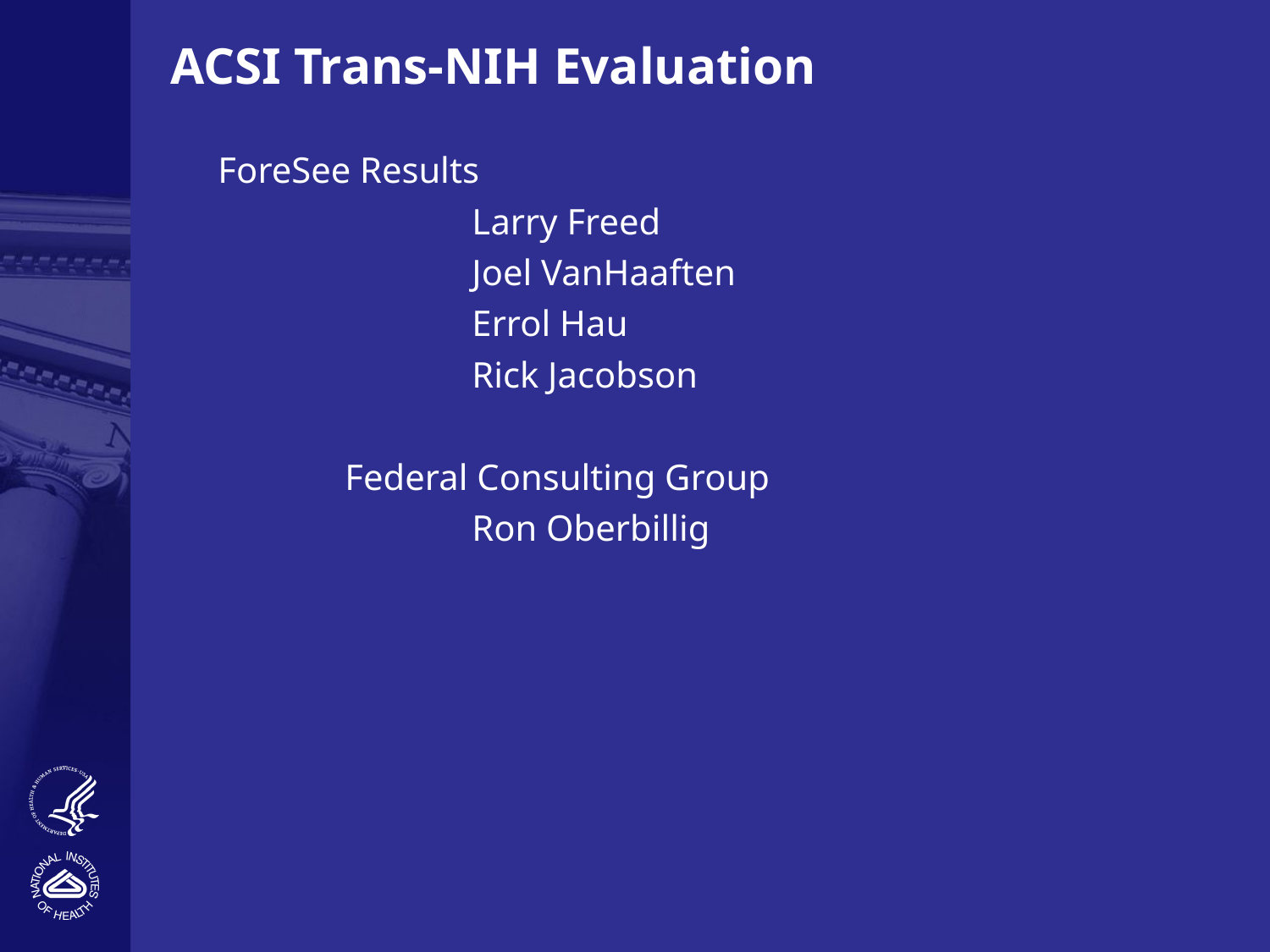

# ACSI Trans-NIH Evaluation
ForeSee Results
		Larry Freed
		Joel VanHaaften
		Errol Hau
		Rick Jacobson
	Federal Consulting Group
		Ron Oberbillig

## Slide 5
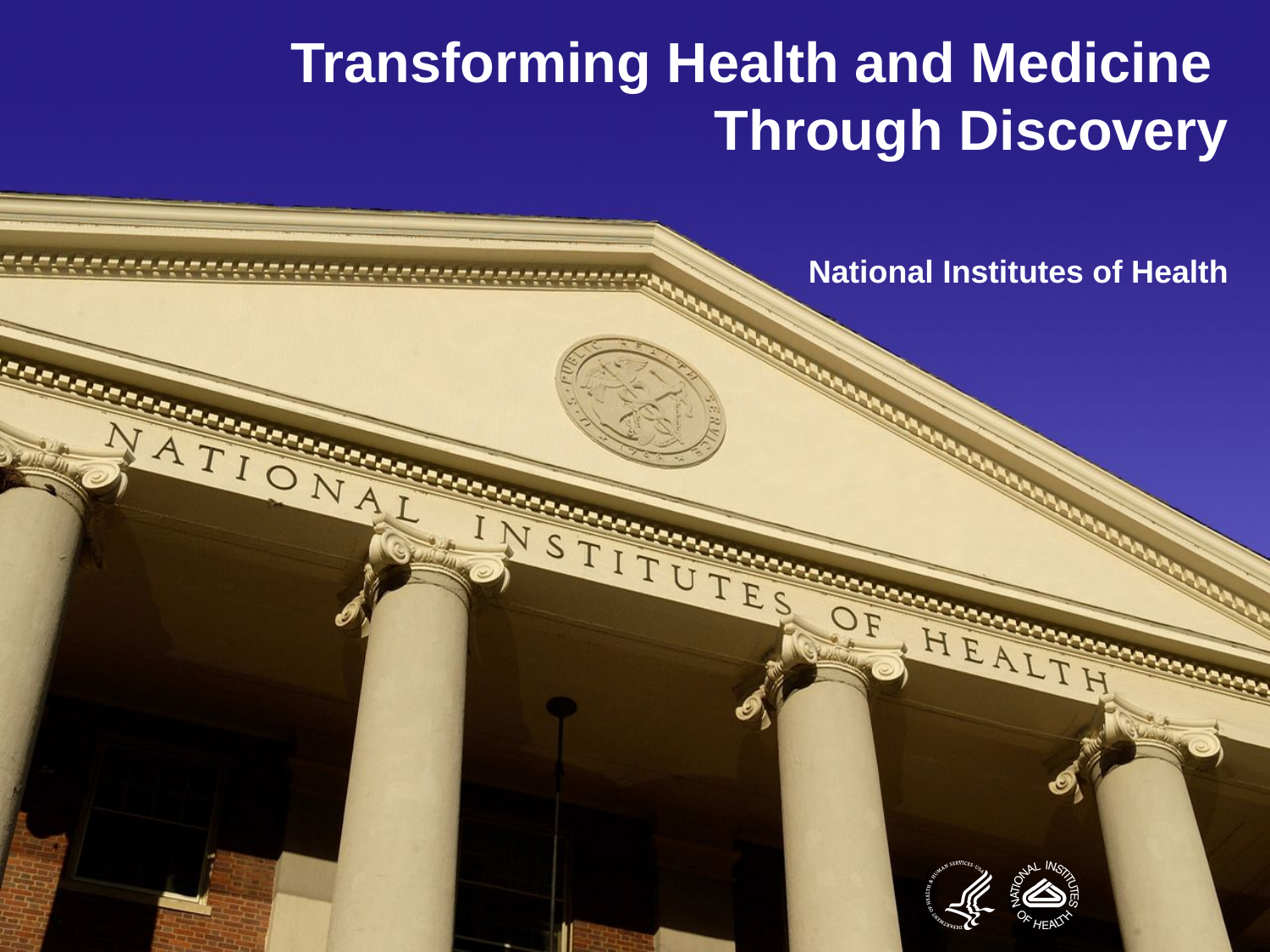

Transforming Health and Medicine
Through Discovery
National Institutes of Health

## Slide 6
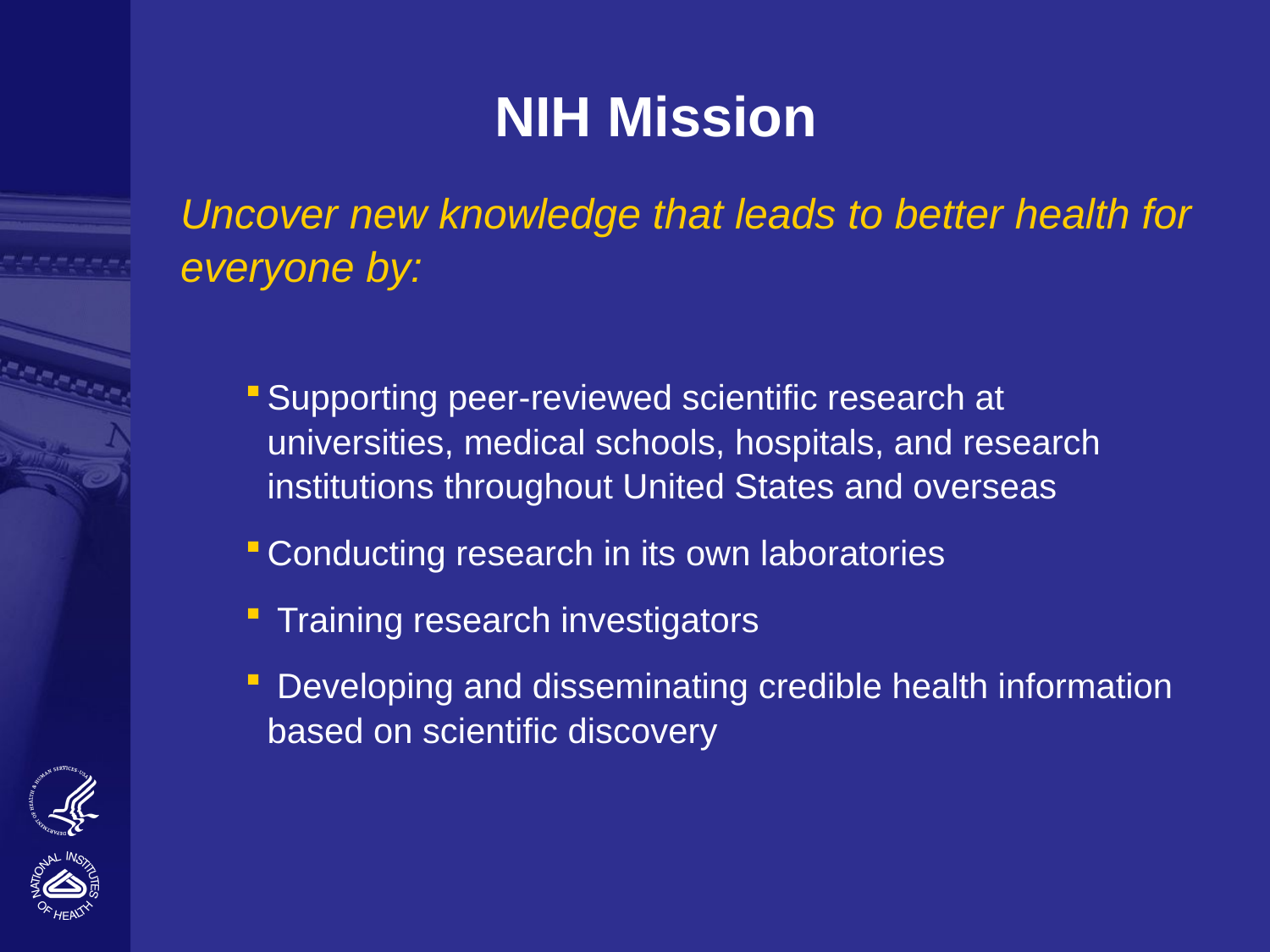

# NIH Mission
Uncover new knowledge that leads to better health for everyone by:
Supporting peer-reviewed scientific research at universities, medical schools, hospitals, and research institutions throughout United States and overseas
Conducting research in its own laboratories
 Training research investigators
 Developing and disseminating credible health information based on scientific discovery

## Slide 7
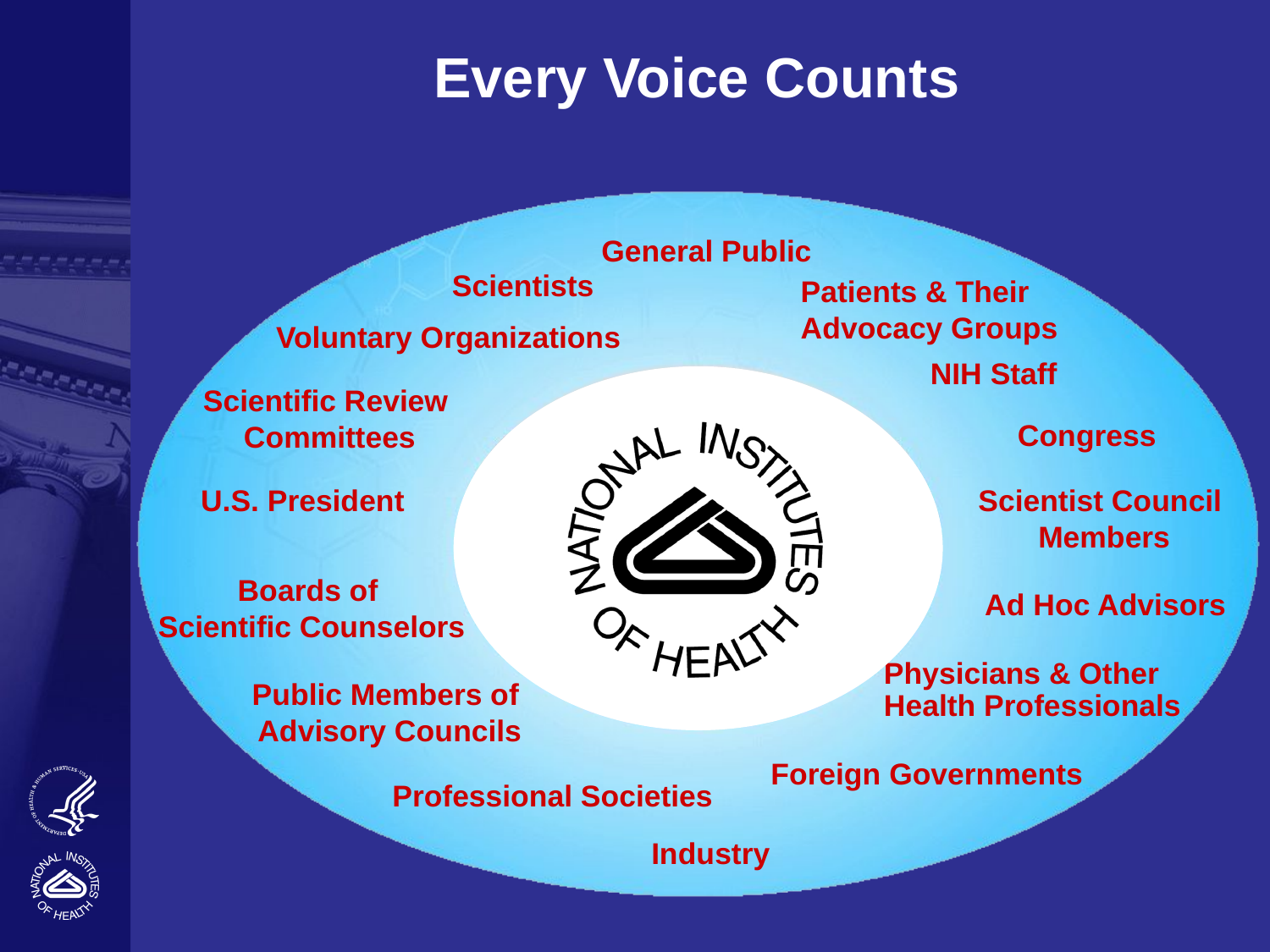

General Public
Scientists
Patients & Their
Advocacy Groups
Voluntary Organizations
NIH Staff
Scientific Review
Committees
Congress
U.S. President
Scientist Council
Members
Boards of
Scientific Counselors
Ad Hoc Advisors
Physicians & Other
Health Professionals
Public Members of
Advisory Councils
Foreign Governments
Professional Societies
Industry
Every Voice Counts

## Slide 8
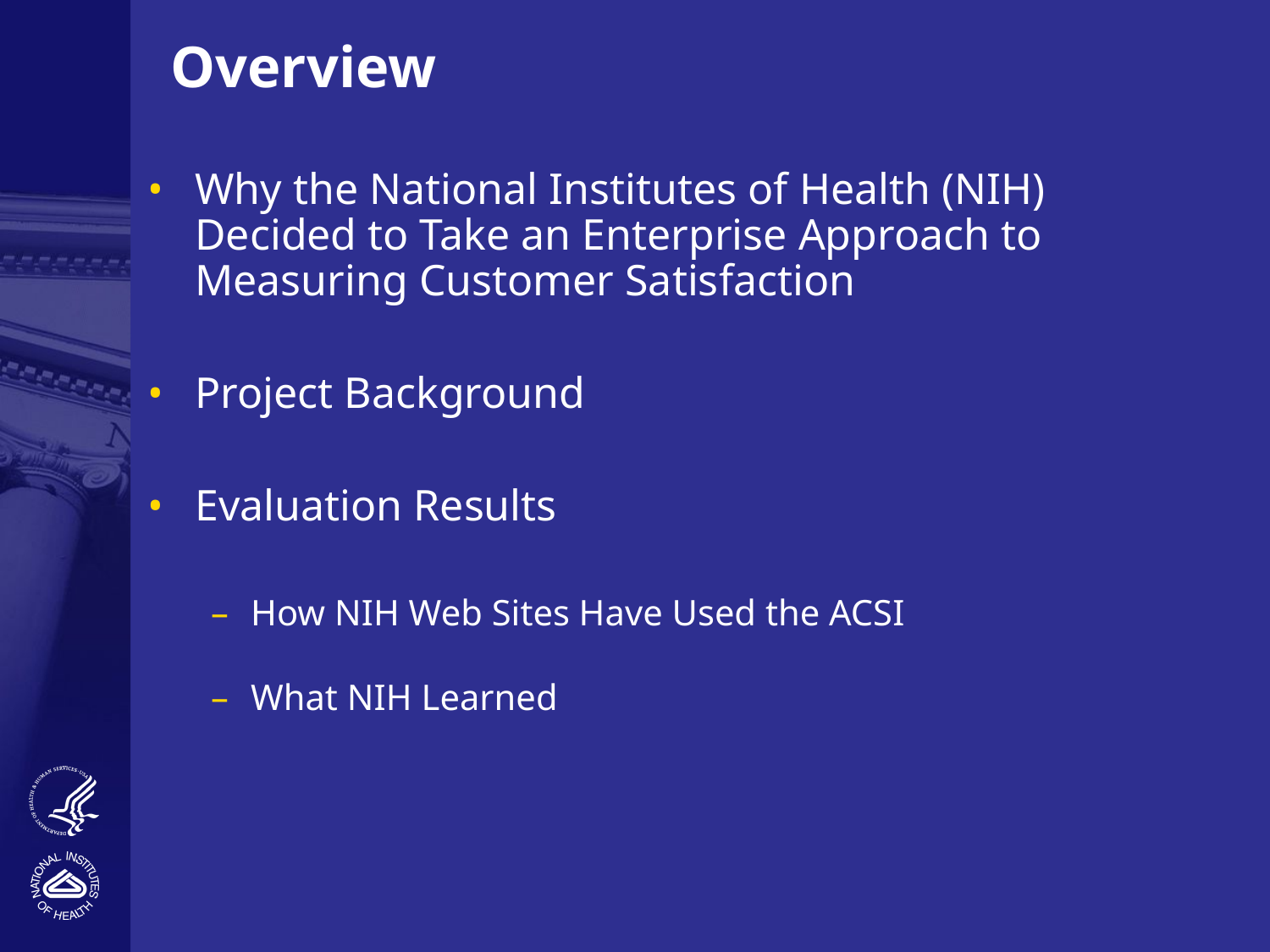

# Overview
Why the National Institutes of Health (NIH) Decided to Take an Enterprise Approach to Measuring Customer Satisfaction
Project Background
Evaluation Results
How NIH Web Sites Have Used the ACSI
What NIH Learned

## Slide 9
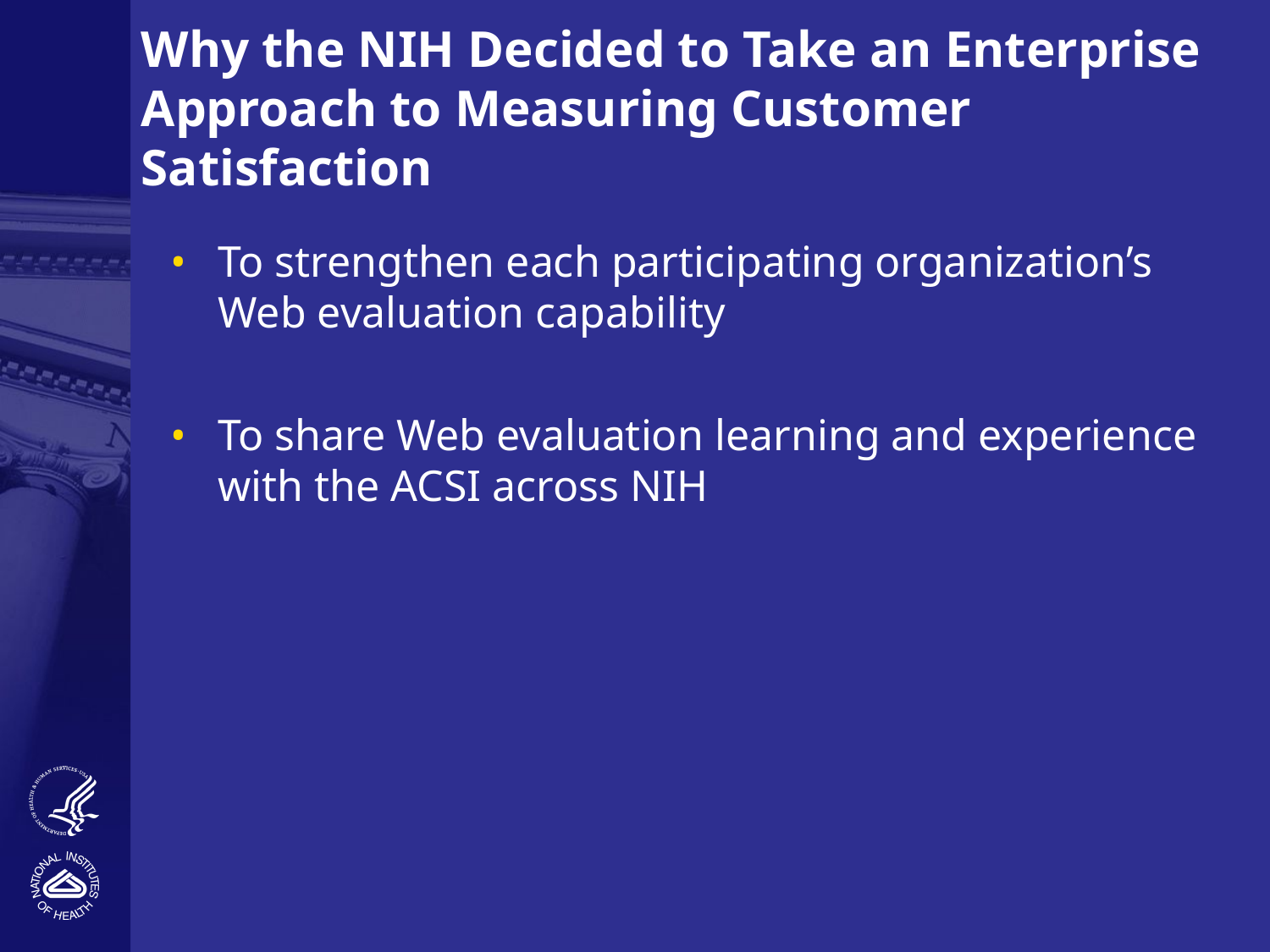

Why the NIH Decided to Take an Enterprise Approach to Measuring Customer Satisfaction
# To strengthen each participating organization’s Web evaluation capability
To share Web evaluation learning and experience with the ACSI across NIH

## Slide 10
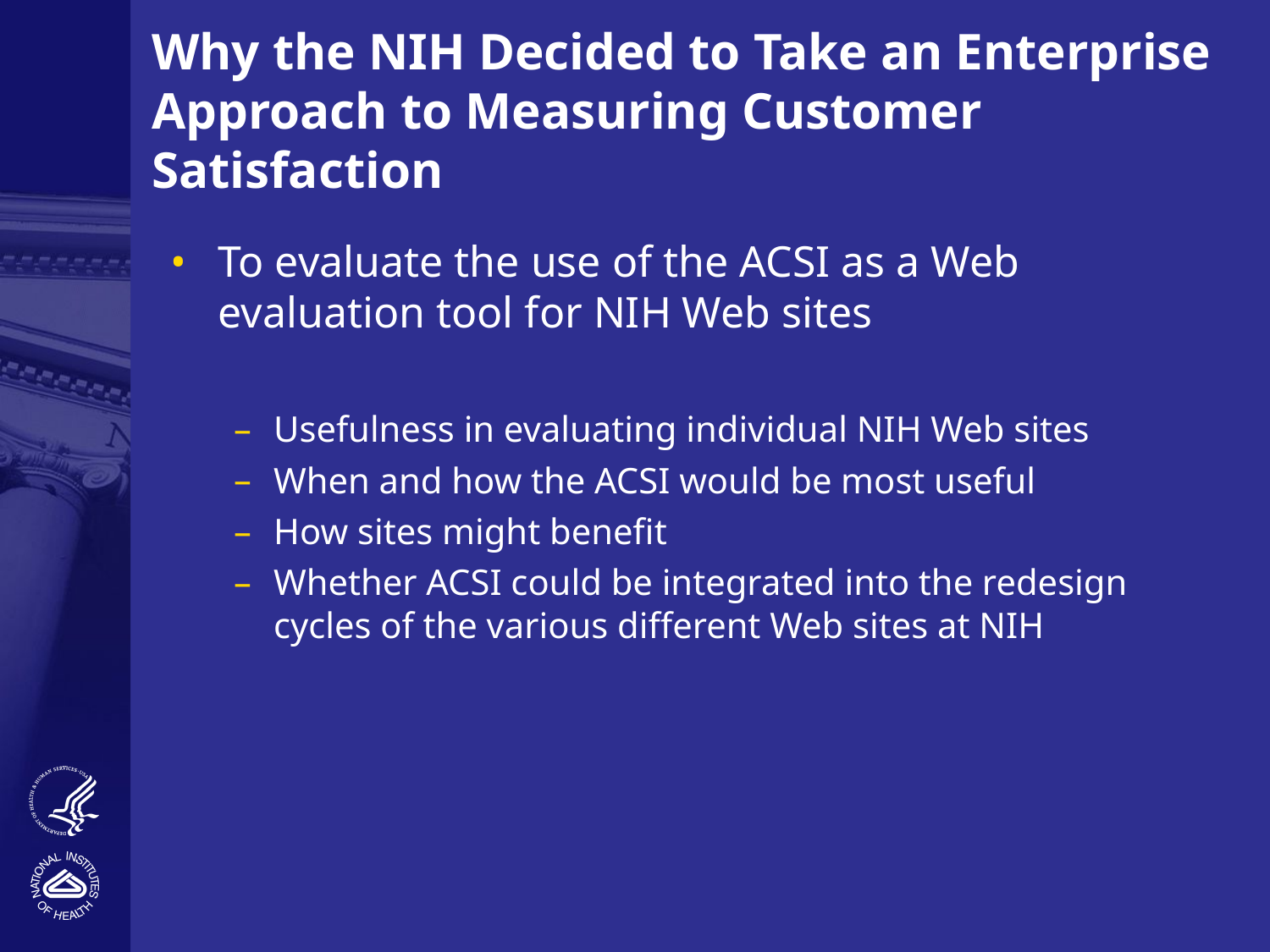

# Why the NIH Decided to Take an Enterprise Approach to Measuring Customer Satisfaction
To evaluate the use of the ACSI as a Web evaluation tool for NIH Web sites
Usefulness in evaluating individual NIH Web sites
When and how the ACSI would be most useful
How sites might benefit
Whether ACSI could be integrated into the redesign cycles of the various different Web sites at NIH

## Slide 11
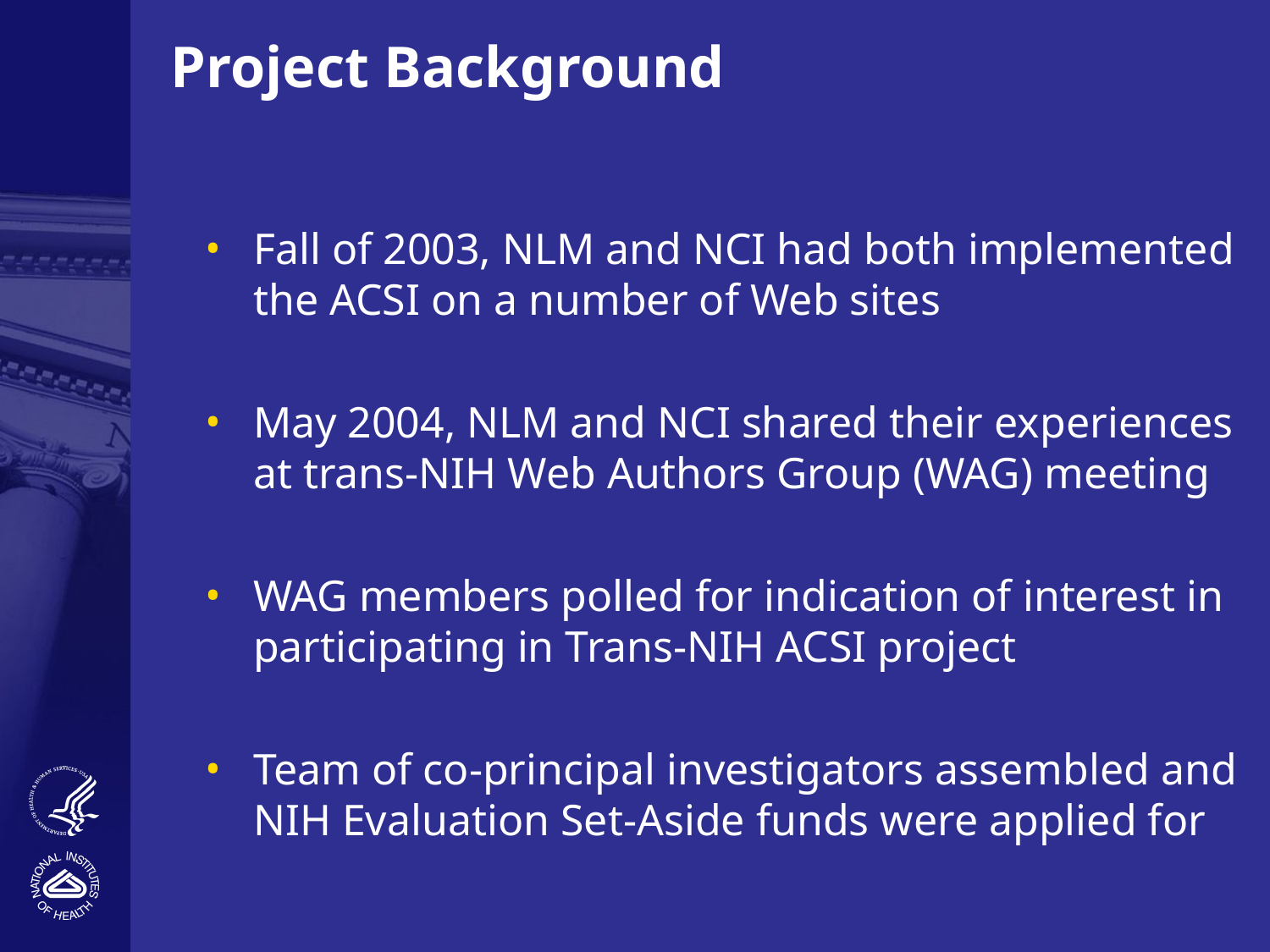

# Project Background
Fall of 2003, NLM and NCI had both implemented the ACSI on a number of Web sites
May 2004, NLM and NCI shared their experiences at trans-NIH Web Authors Group (WAG) meeting
WAG members polled for indication of interest in participating in Trans-NIH ACSI project
Team of co-principal investigators assembled and NIH Evaluation Set-Aside funds were applied for

## Slide 12
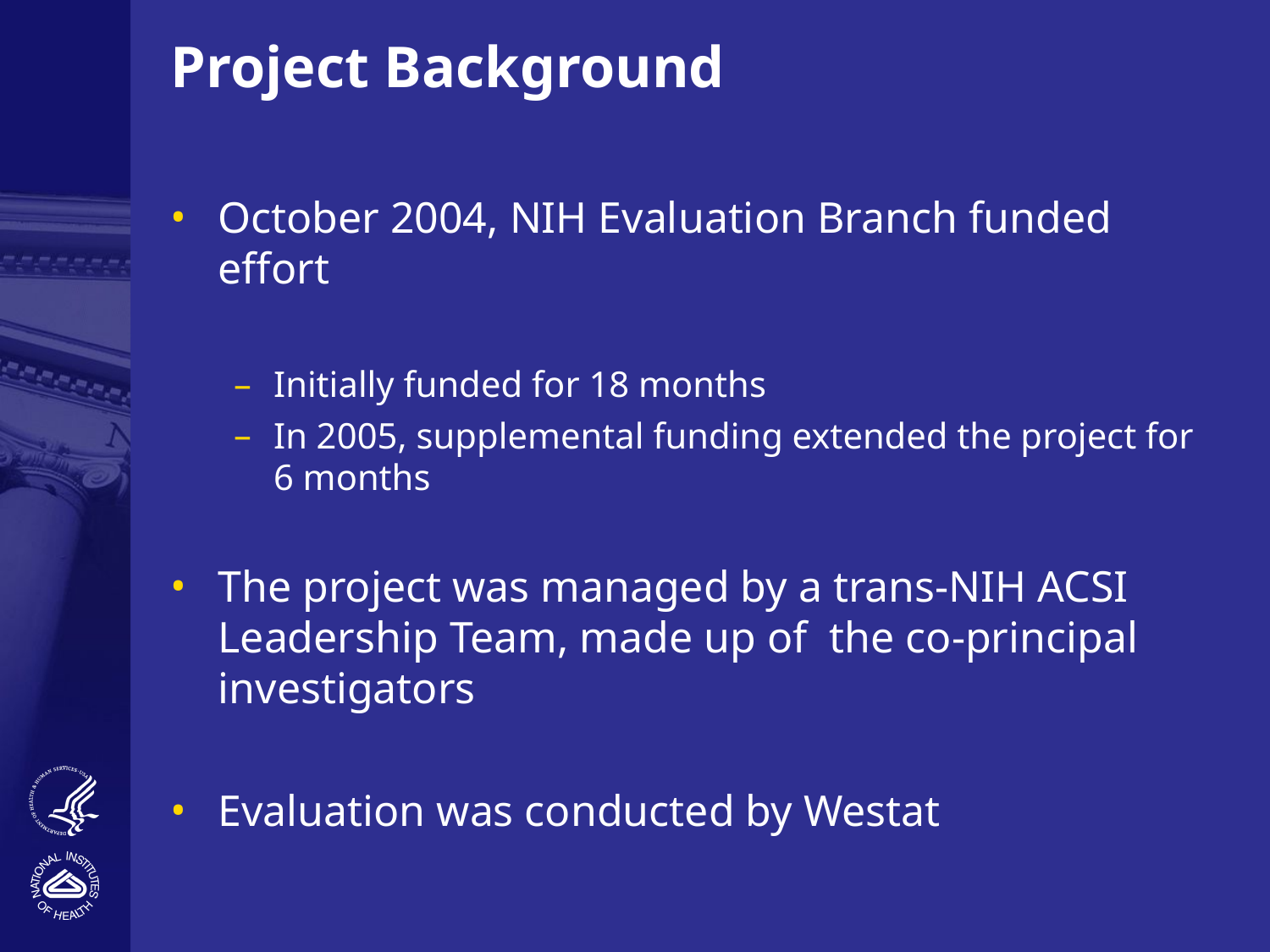

# Project Background
October 2004, NIH Evaluation Branch funded effort
Initially funded for 18 months
In 2005, supplemental funding extended the project for 6 months
The project was managed by a trans-NIH ACSI Leadership Team, made up of the co-principal investigators
Evaluation was conducted by Westat

## Slide 13
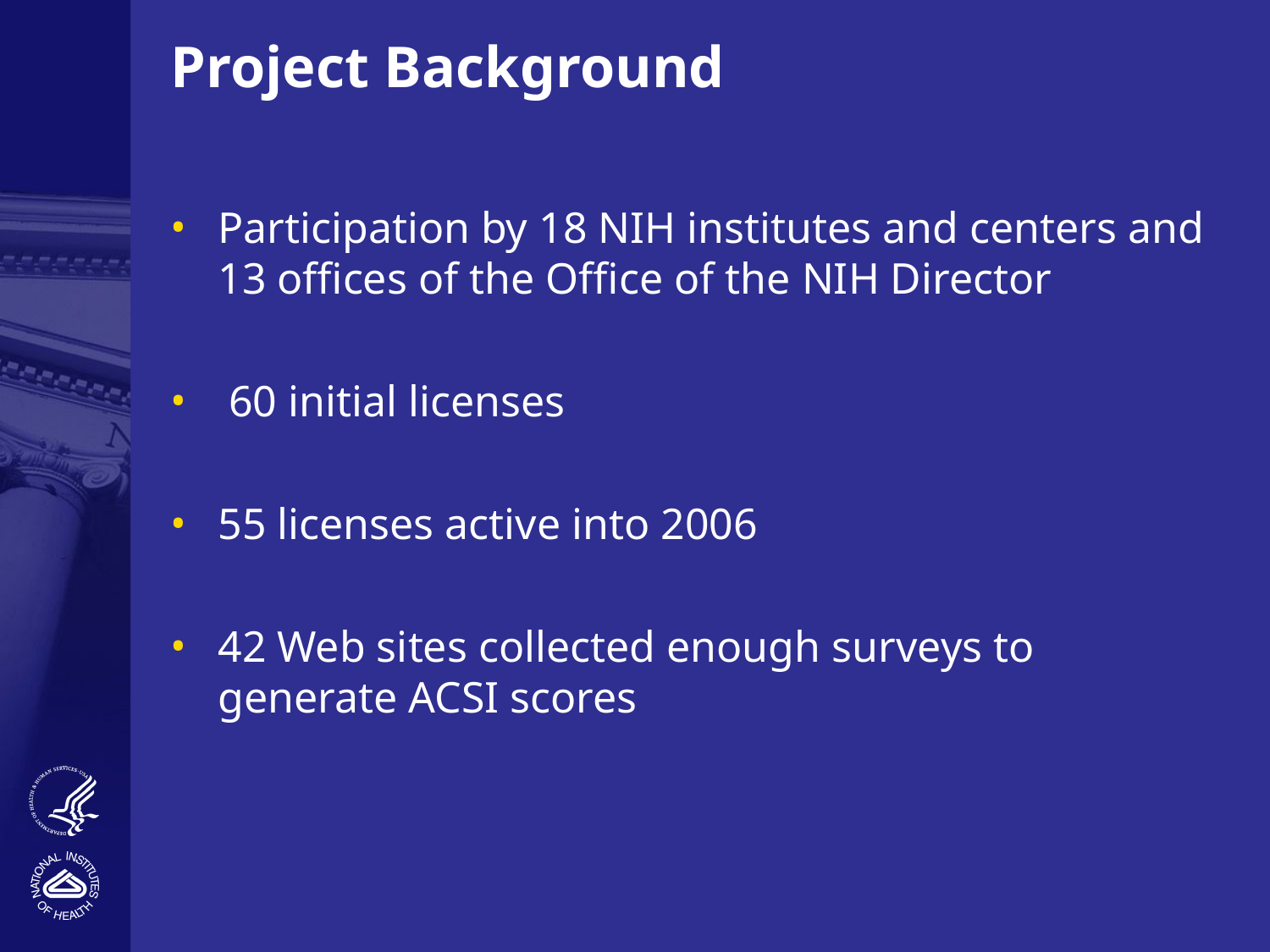

# Project Background
Participation by 18 NIH institutes and centers and 13 offices of the Office of the NIH Director
 60 initial licenses
55 licenses active into 2006
42 Web sites collected enough surveys to generate ACSI scores

## Slide 14
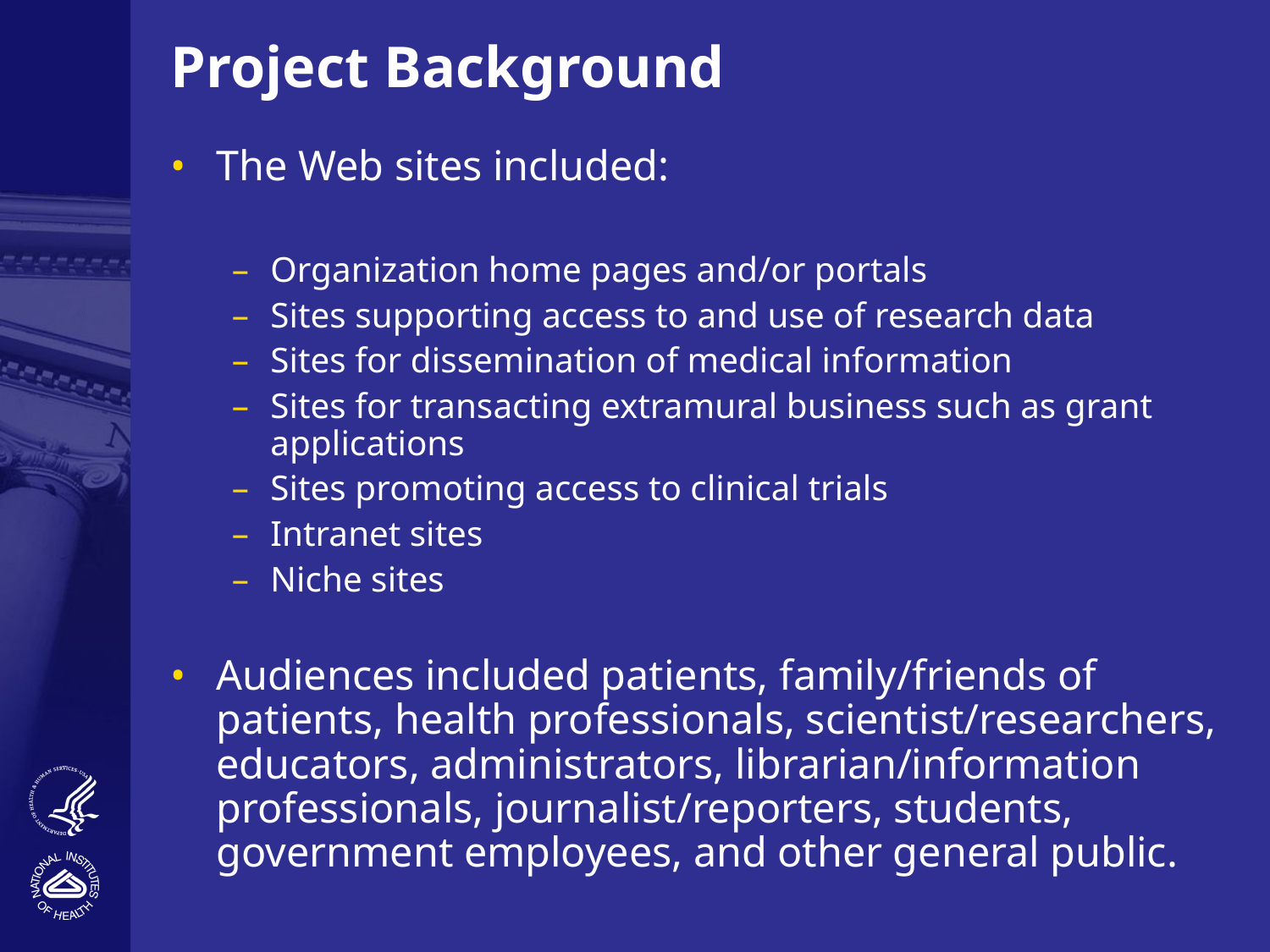

# Project Background
The Web sites included:
Organization home pages and/or portals
Sites supporting access to and use of research data
Sites for dissemination of medical information
Sites for transacting extramural business such as grant applications
Sites promoting access to clinical trials
Intranet sites
Niche sites
Audiences included patients, family/friends of patients, health professionals, scientist/researchers, educators, administrators, librarian/information professionals, journalist/reporters, students, government employees, and other general public.

## Slide 15
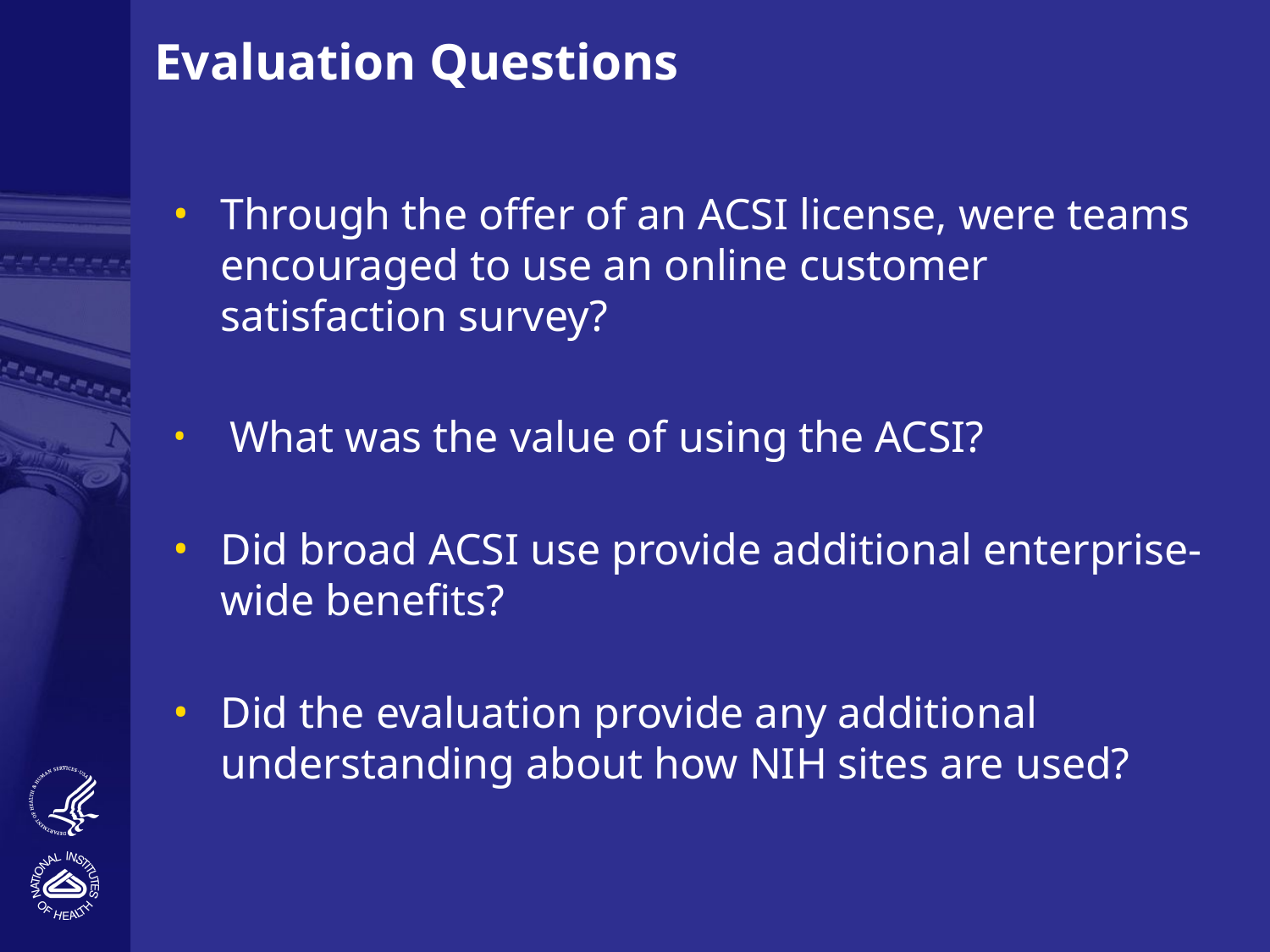

# Evaluation Questions
Through the offer of an ACSI license, were teams encouraged to use an online customer satisfaction survey?
 What was the value of using the ACSI?
Did broad ACSI use provide additional enterprise-wide benefits?
Did the evaluation provide any additional understanding about how NIH sites are used?

## Slide 16
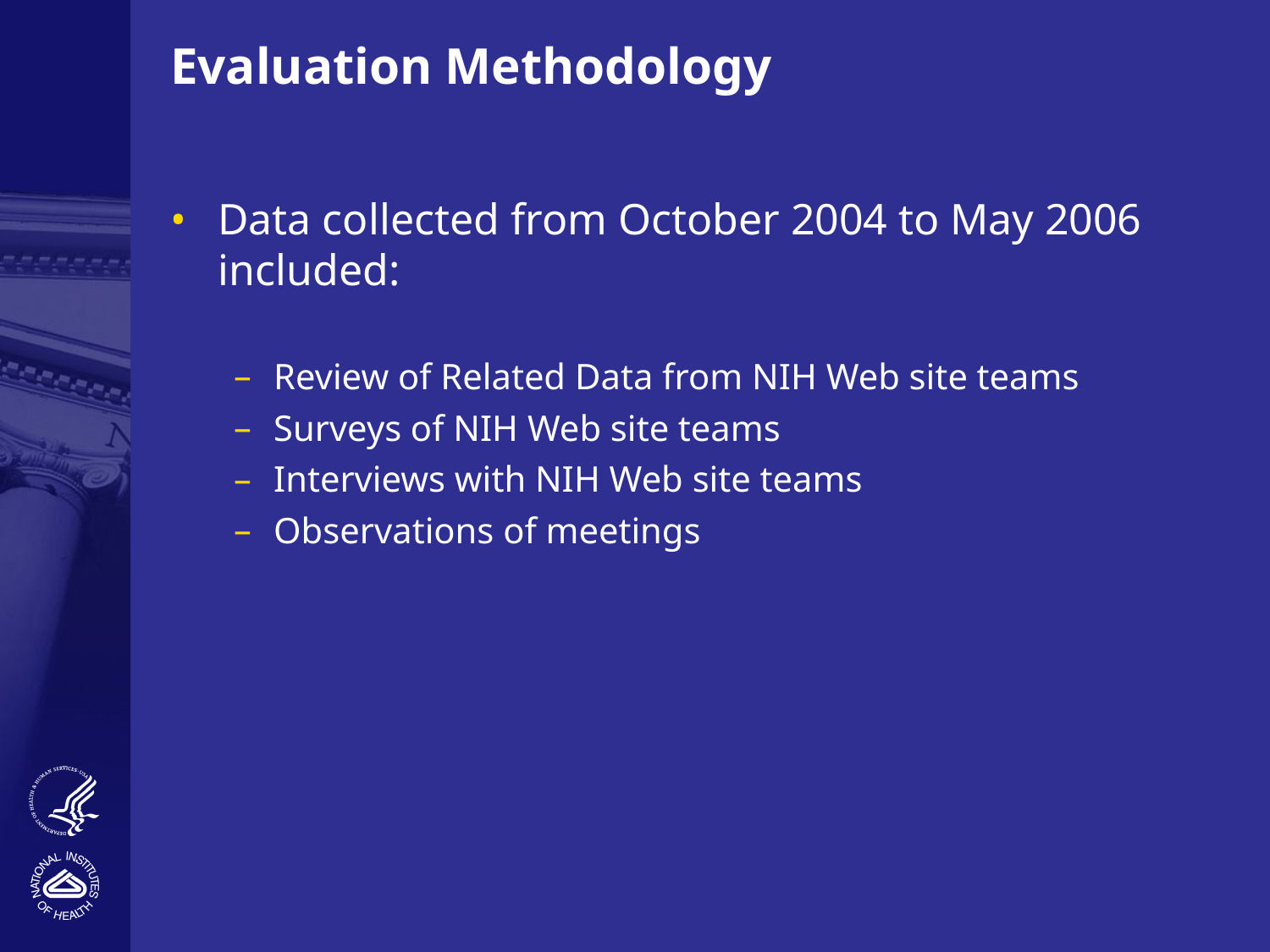

# Evaluation Methodology
Data collected from October 2004 to May 2006 included:
Review of Related Data from NIH Web site teams
Surveys of NIH Web site teams
Interviews with NIH Web site teams
Observations of meetings

## Slide 17
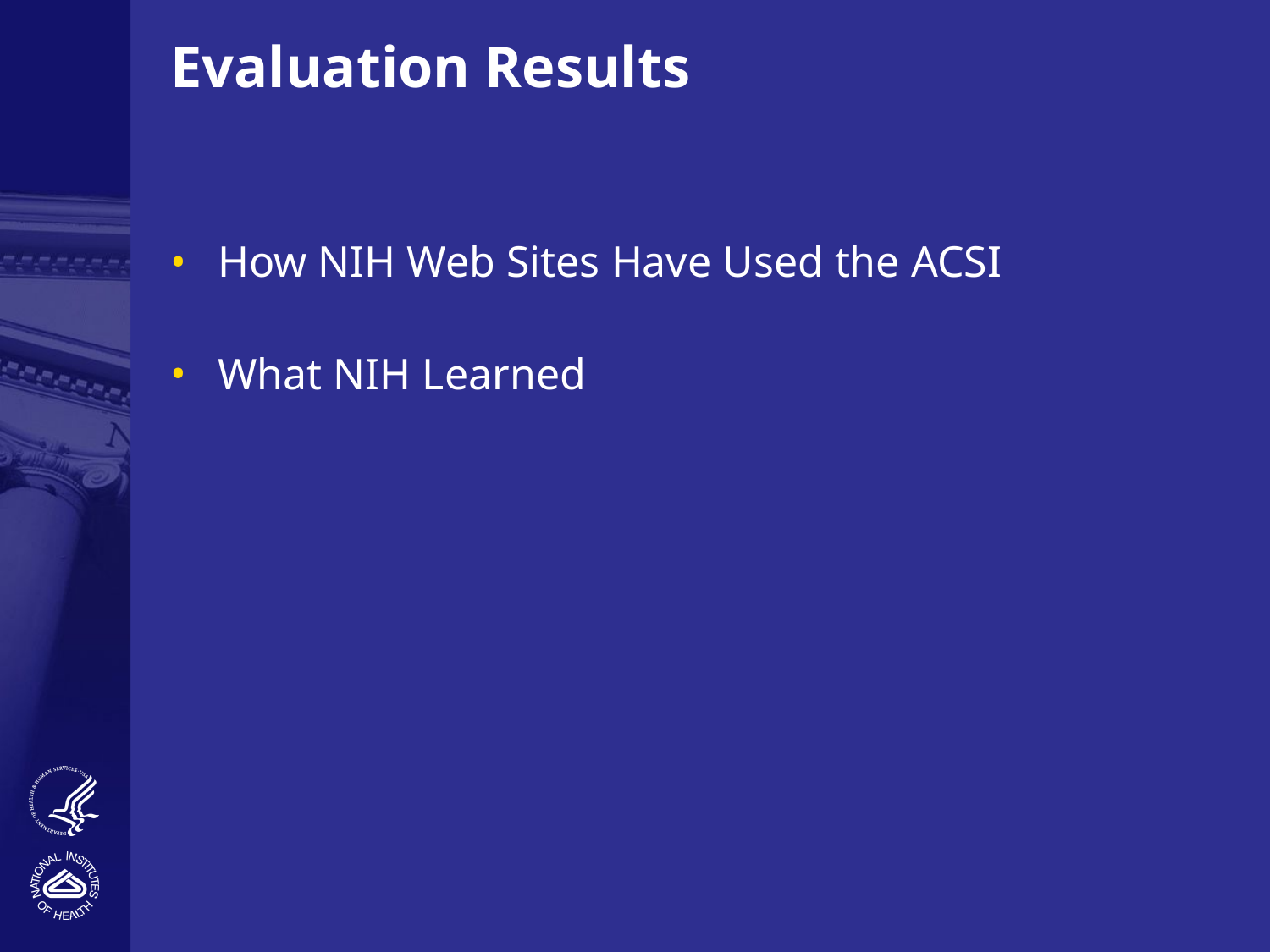

# Evaluation Results
How NIH Web Sites Have Used the ACSI
What NIH Learned

## Slide 18
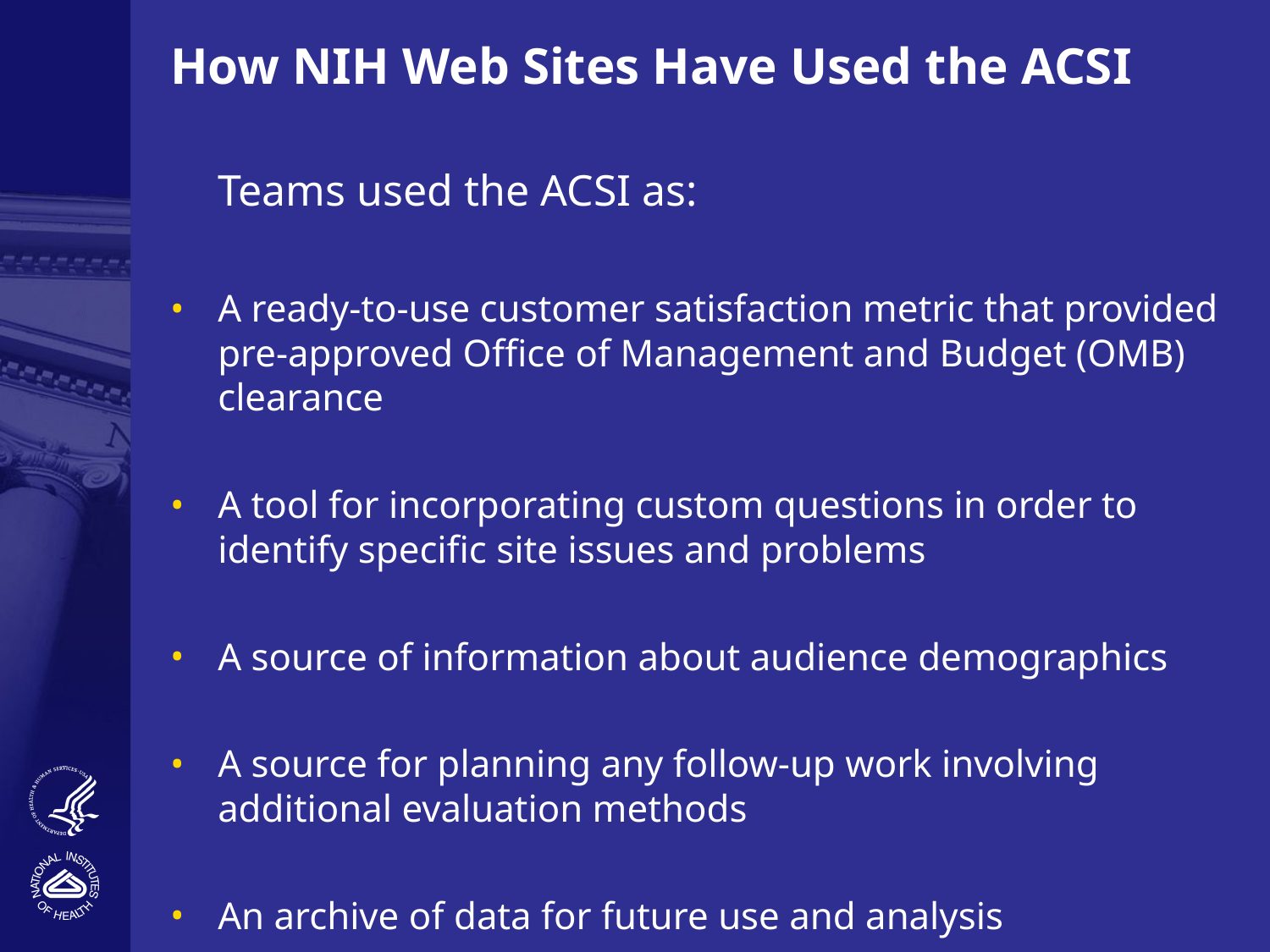

# How NIH Web Sites Have Used the ACSI
Teams used the ACSI as:
A ready-to-use customer satisfaction metric that provided pre-approved Office of Management and Budget (OMB) clearance
A tool for incorporating custom questions in order to identify specific site issues and problems
A source of information about audience demographics
A source for planning any follow-up work involving additional evaluation methods
An archive of data for future use and analysis

## Slide 19
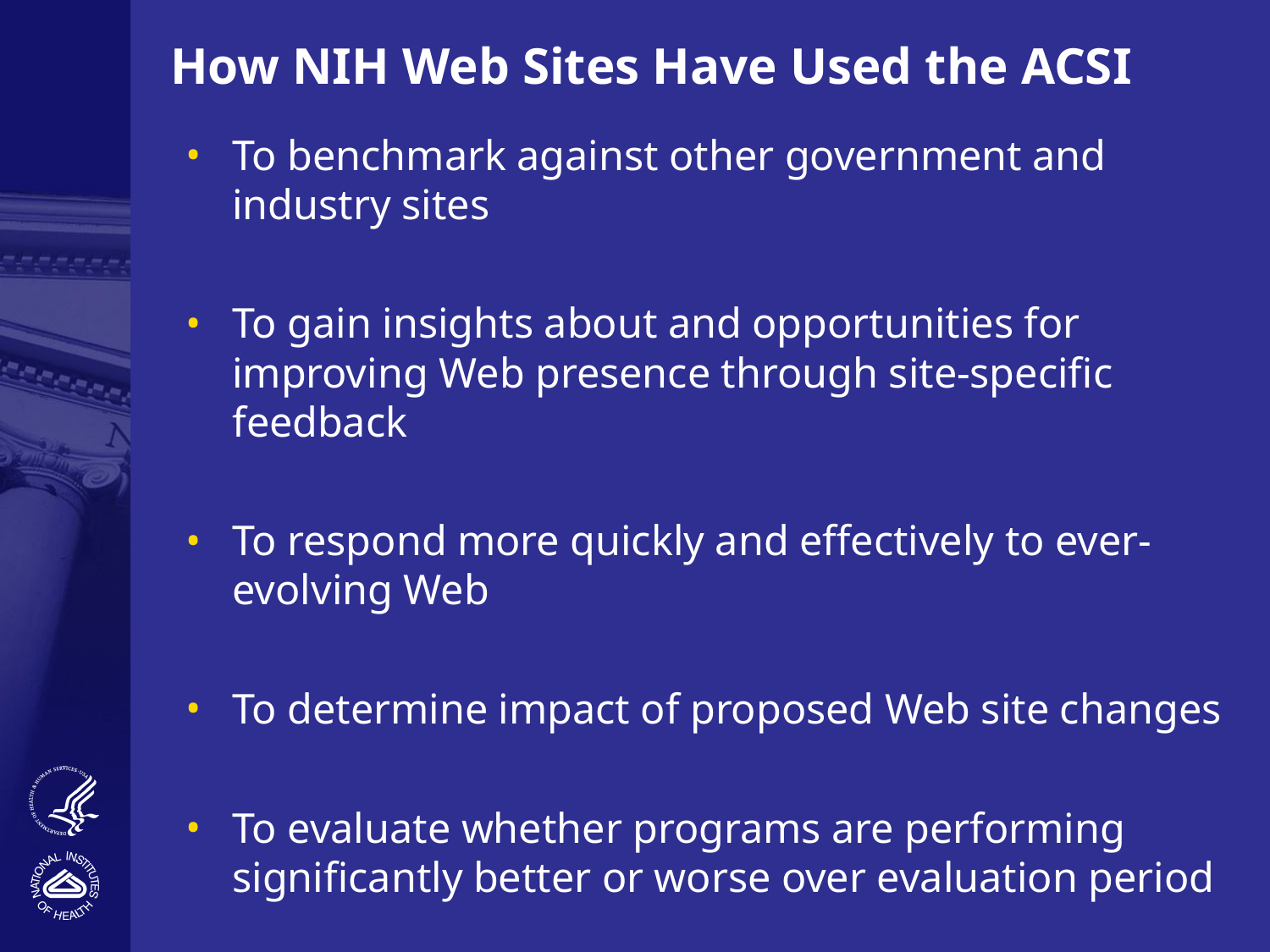

# How NIH Web Sites Have Used the ACSI
To benchmark against other government and industry sites
To gain insights about and opportunities for improving Web presence through site-specific feedback
To respond more quickly and effectively to ever-evolving Web
To determine impact of proposed Web site changes
To evaluate whether programs are performing significantly better or worse over evaluation period

## Slide 20
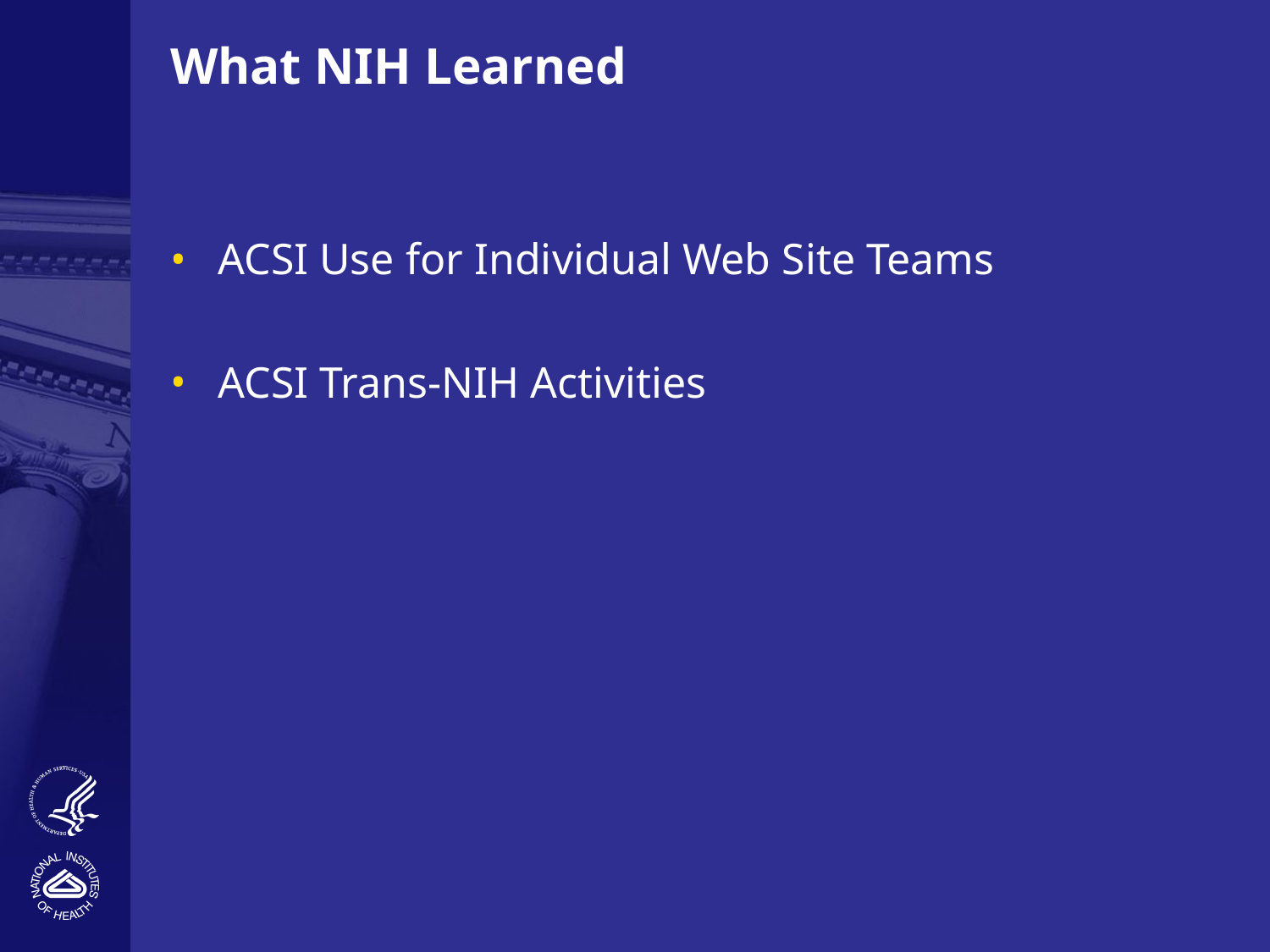

# What NIH Learned
ACSI Use for Individual Web Site Teams
ACSI Trans-NIH Activities

## Slide 21
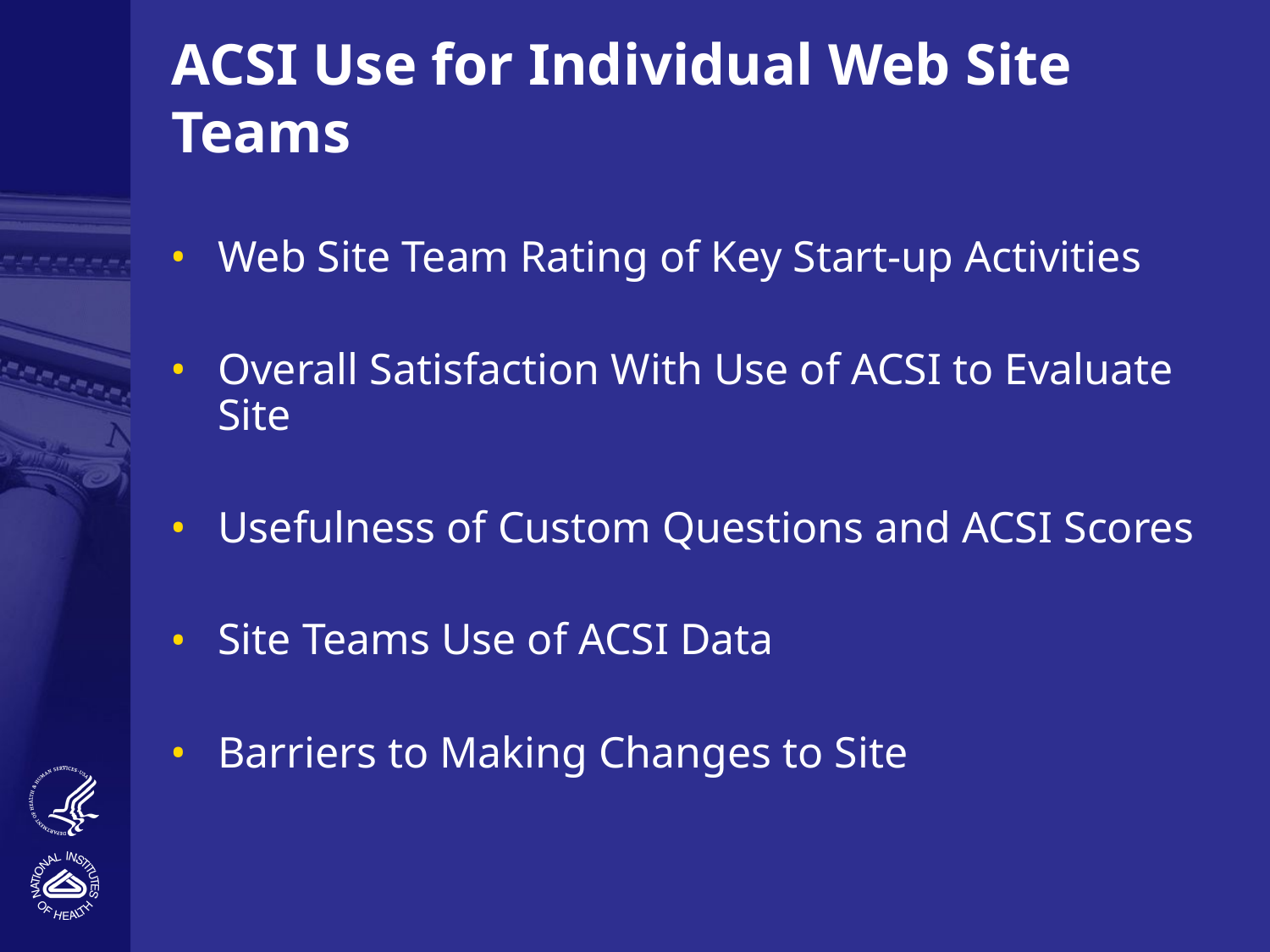

# ACSI Use for Individual Web Site Teams
Web Site Team Rating of Key Start-up Activities
Overall Satisfaction With Use of ACSI to Evaluate Site
Usefulness of Custom Questions and ACSI Scores
Site Teams Use of ACSI Data
Barriers to Making Changes to Site

## Slide 22
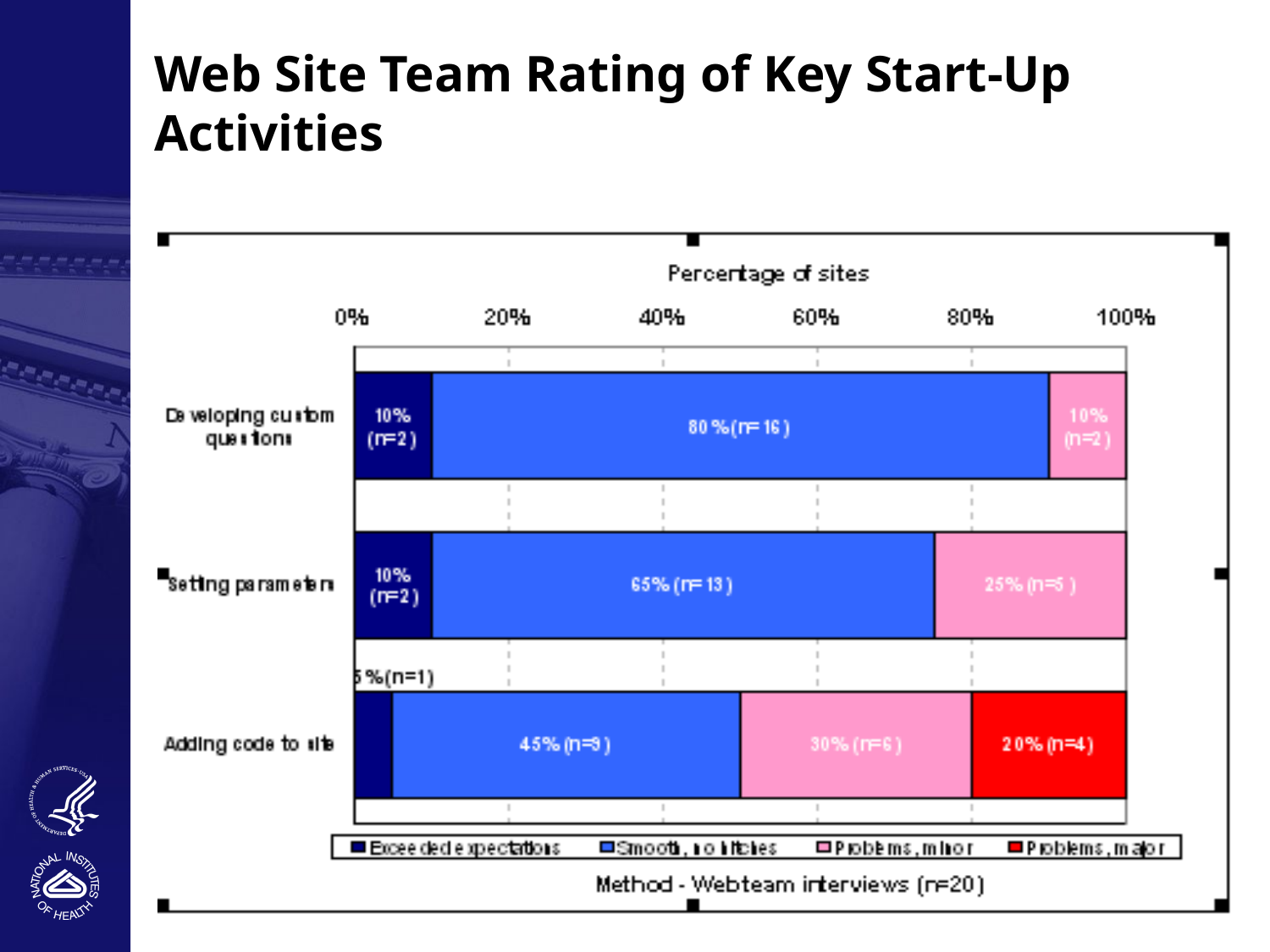

# Web Site Team Rating of Key Start-Up Activities

## Slide 23
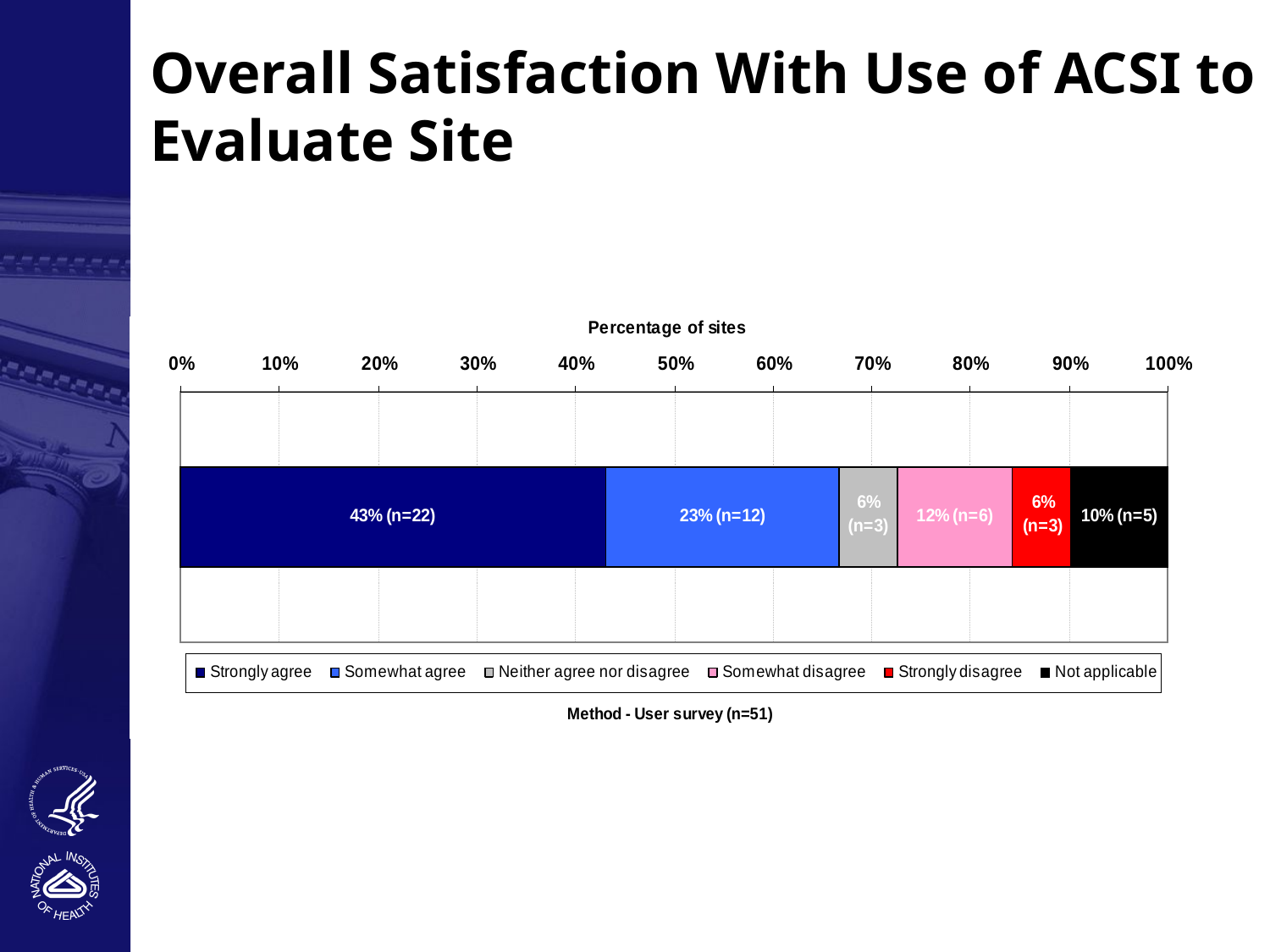

# Overall Satisfaction With Use of ACSI to Evaluate Site

## Slide 24
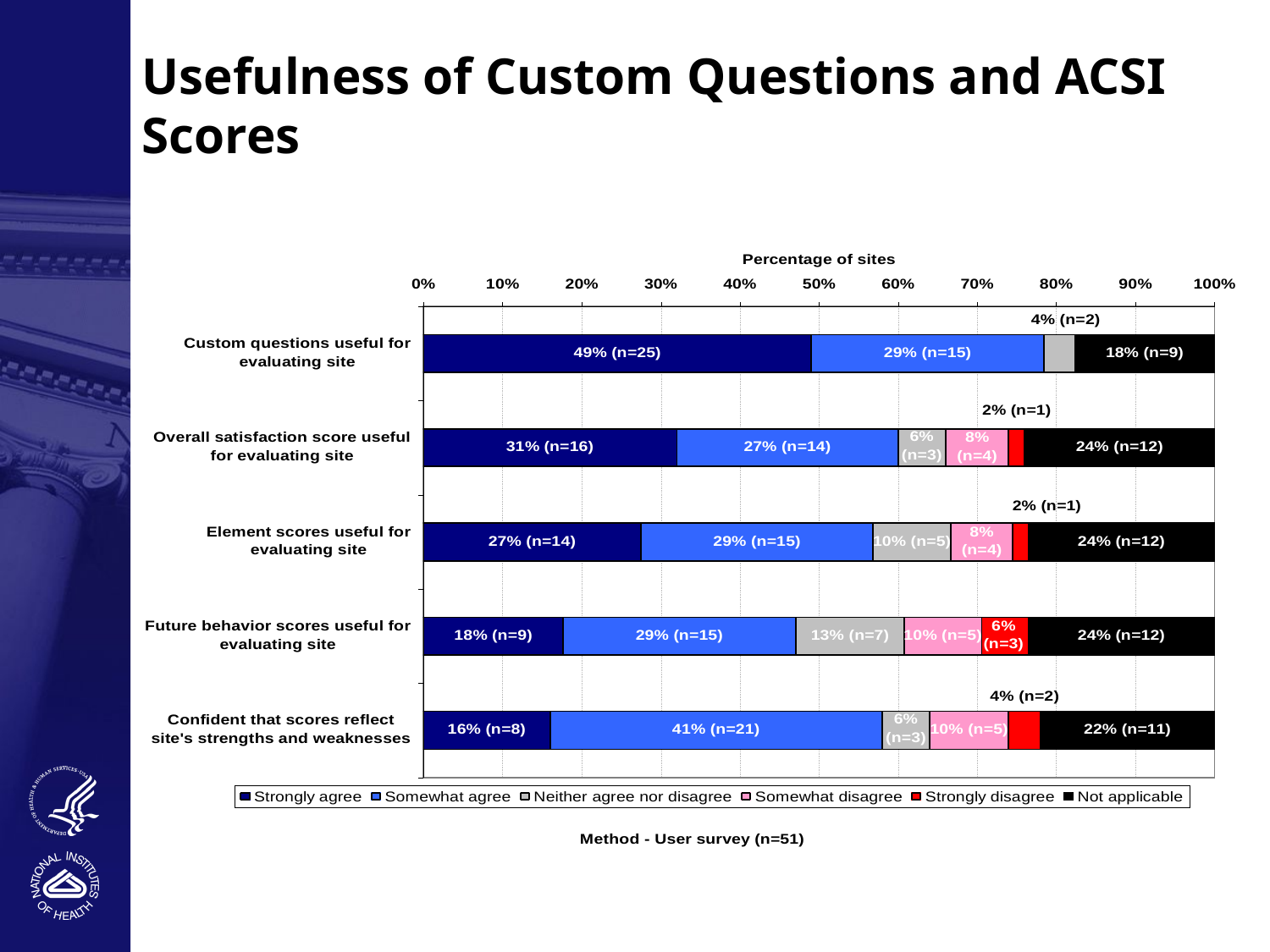

# Usefulness of Custom Questions and ACSI Scores

## Slide 25
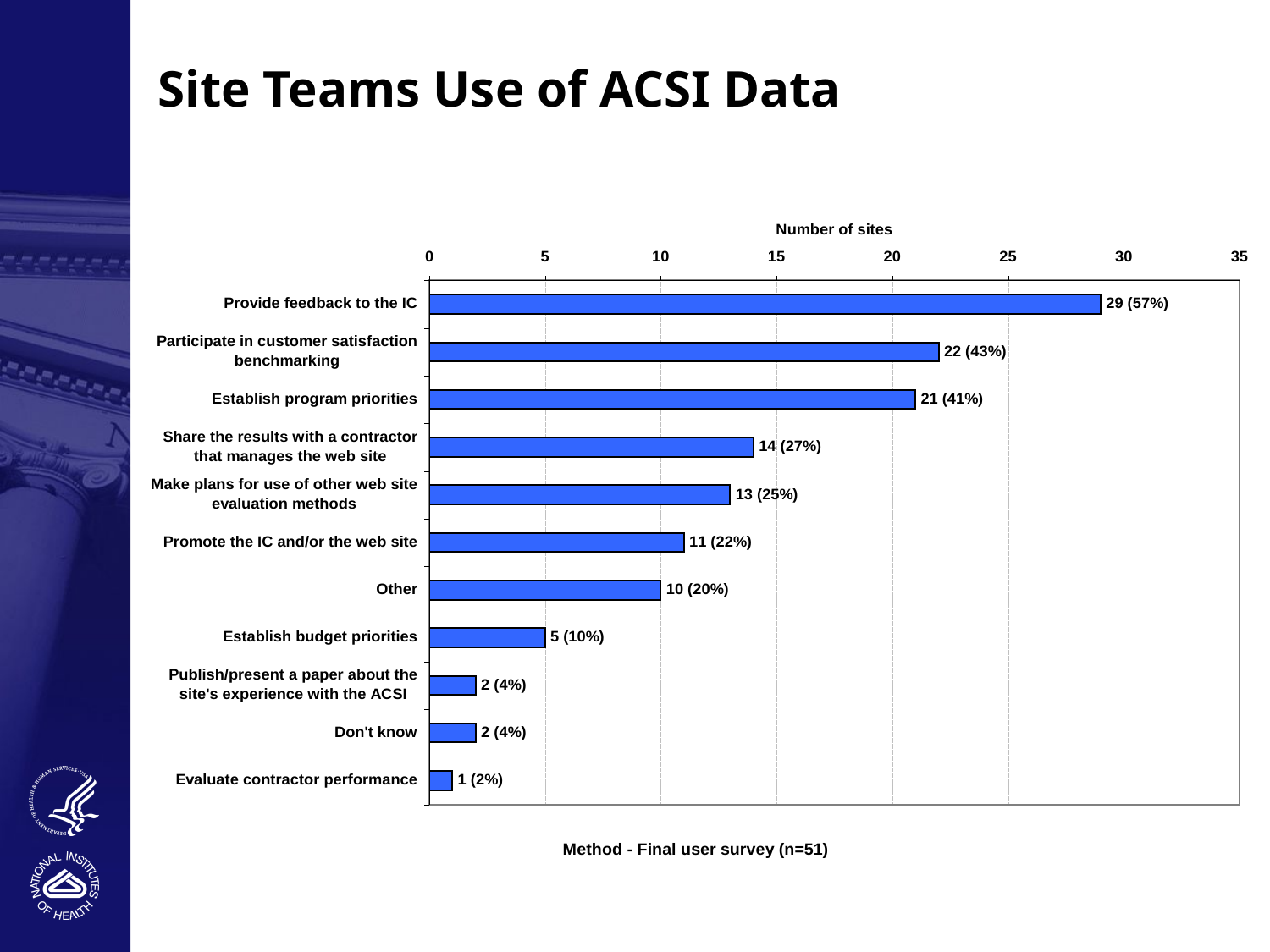

Site Teams Use of ACSI Data

## Slide 26
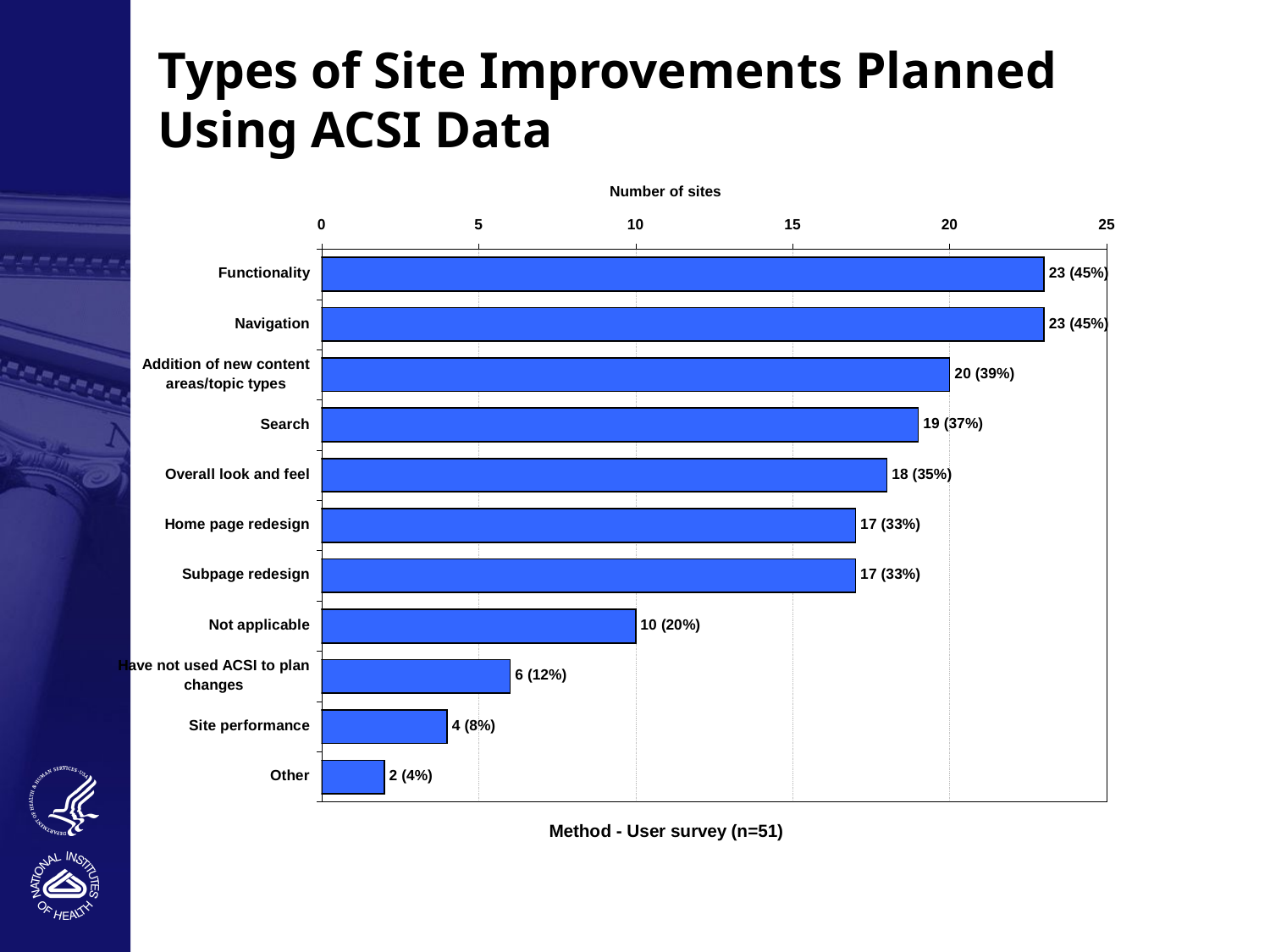

Types of Site Improvements Planned
Using ACSI Data

## Slide 27
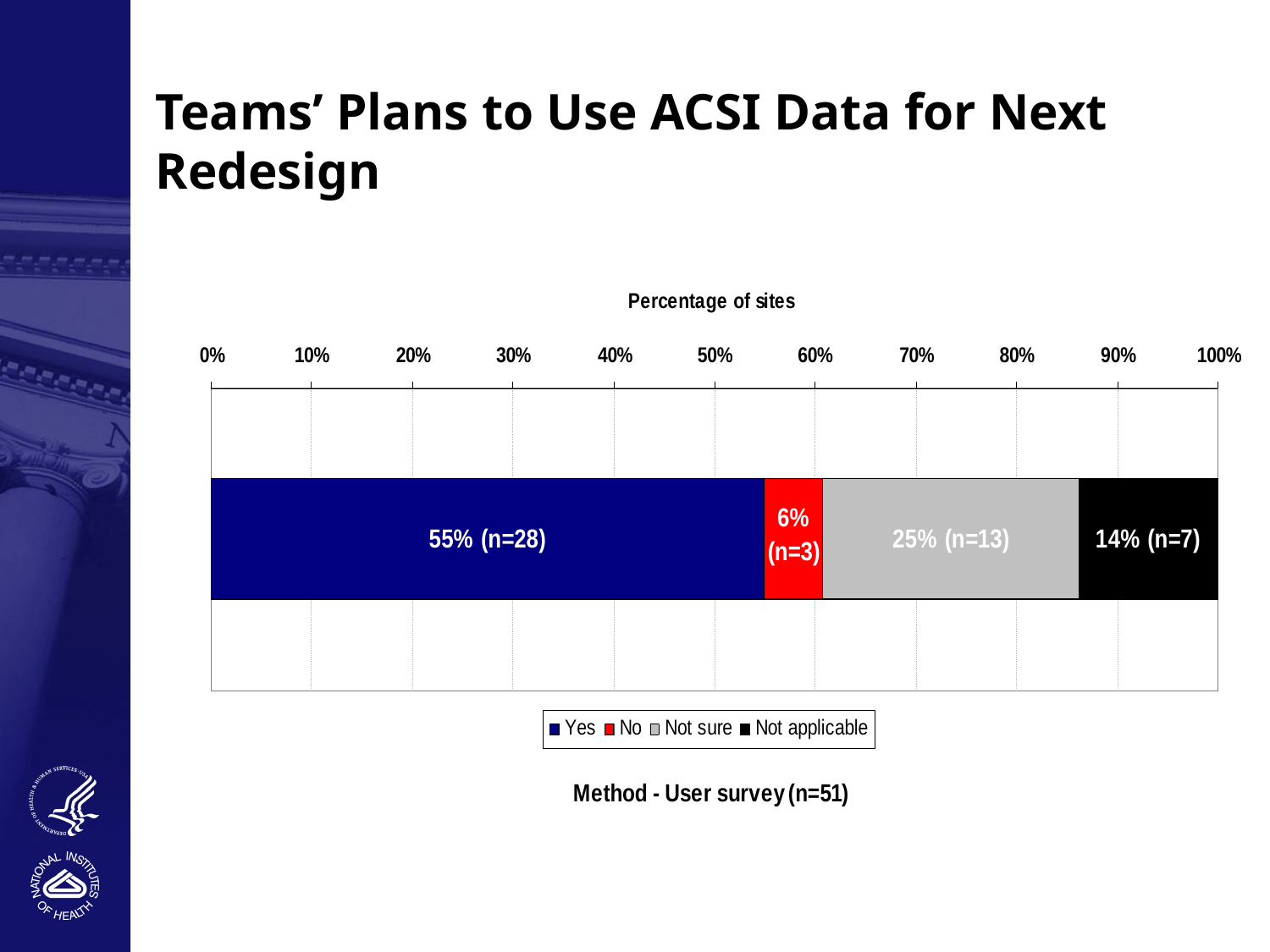

Teams’ Plans to Use ACSI Data for Next Redesign

## Slide 28
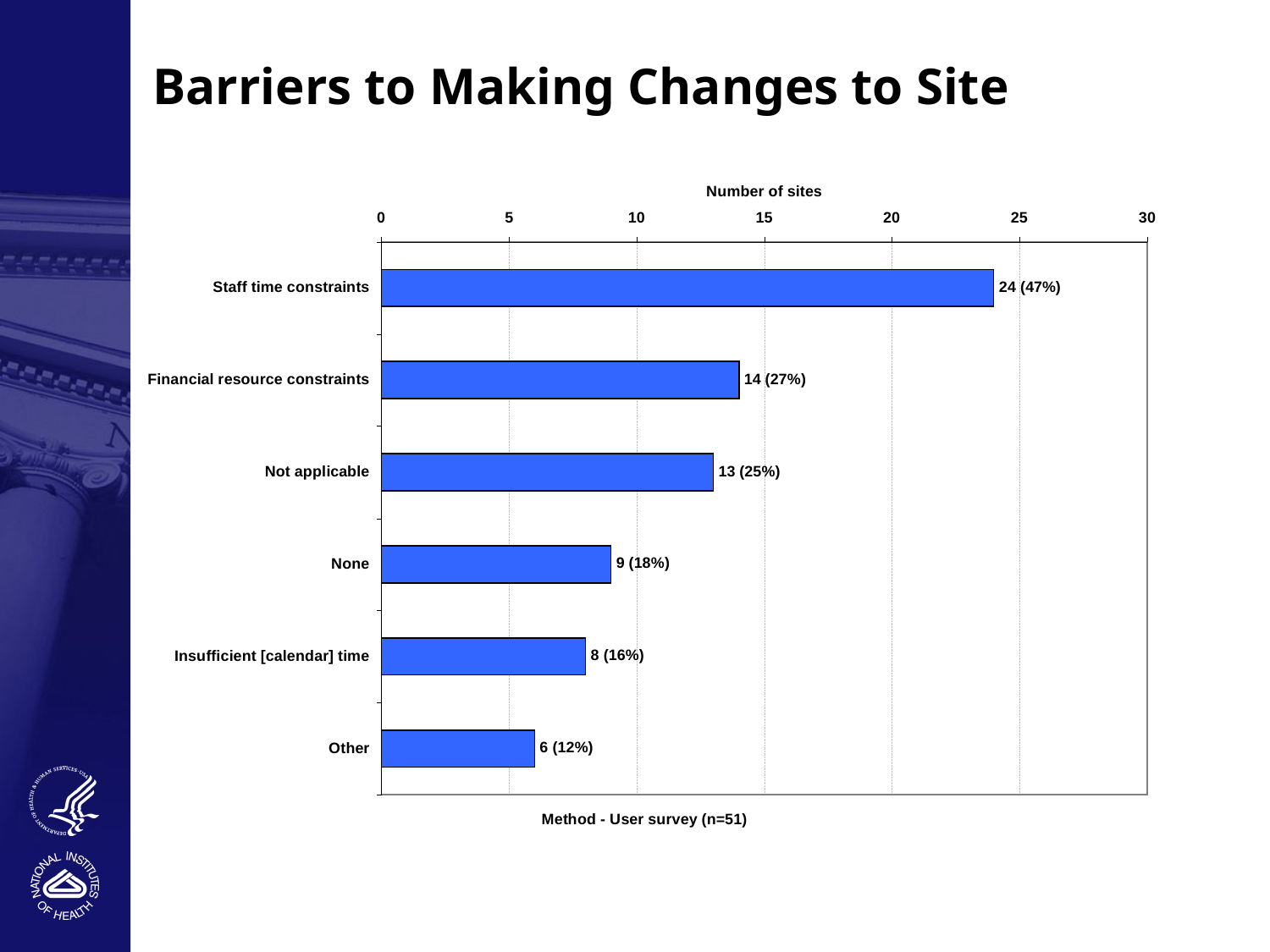

Barriers to Making Changes to Site

## Slide 29
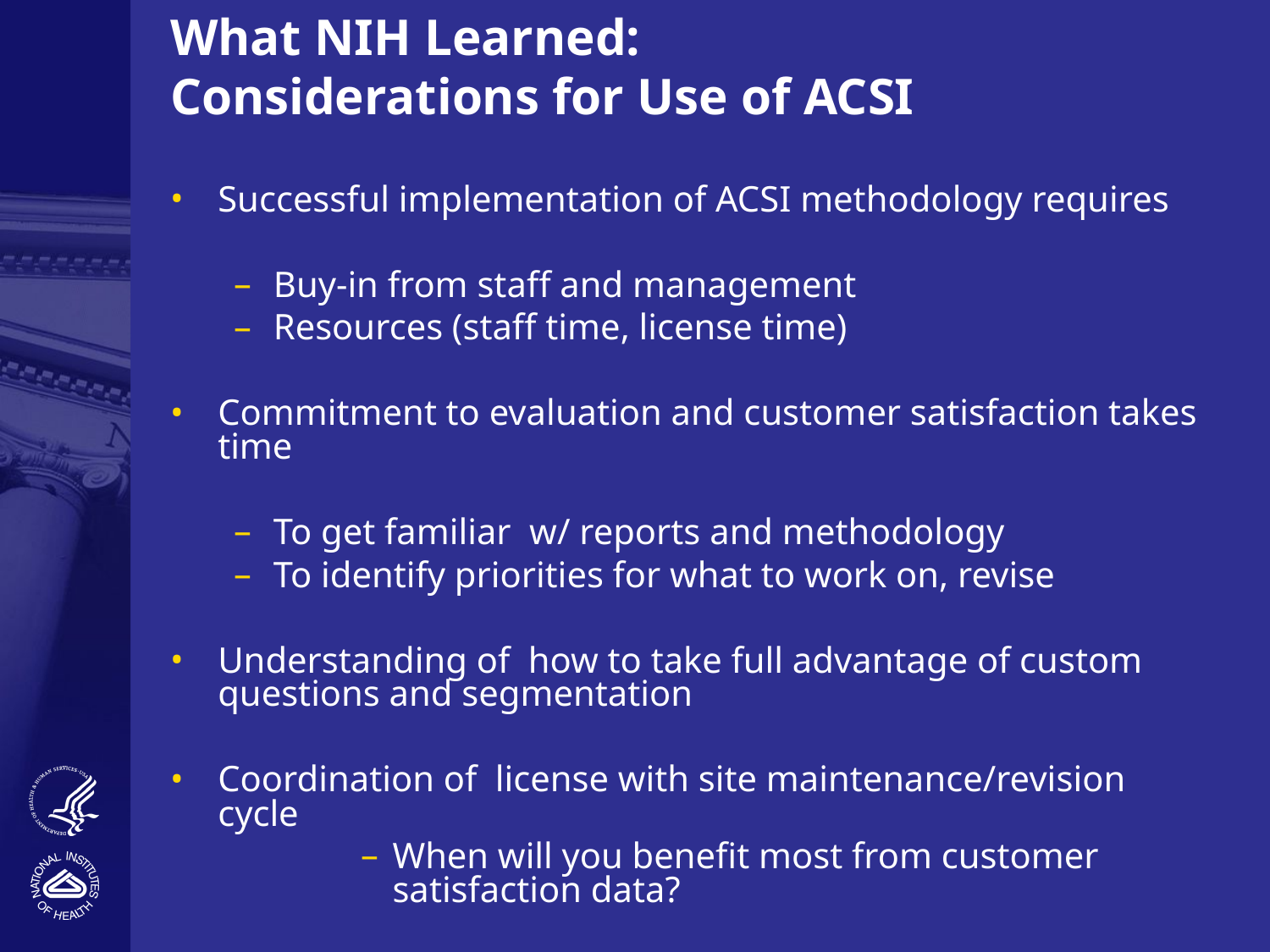

# What NIH Learned: Considerations for Use of ACSI
Successful implementation of ACSI methodology requires
Buy-in from staff and management
Resources (staff time, license time)
Commitment to evaluation and customer satisfaction takes time
To get familiar w/ reports and methodology
To identify priorities for what to work on, revise
Understanding of how to take full advantage of custom questions and segmentation
Coordination of license with site maintenance/revision cycle
When will you benefit most from customer satisfaction data?

## Slide 30
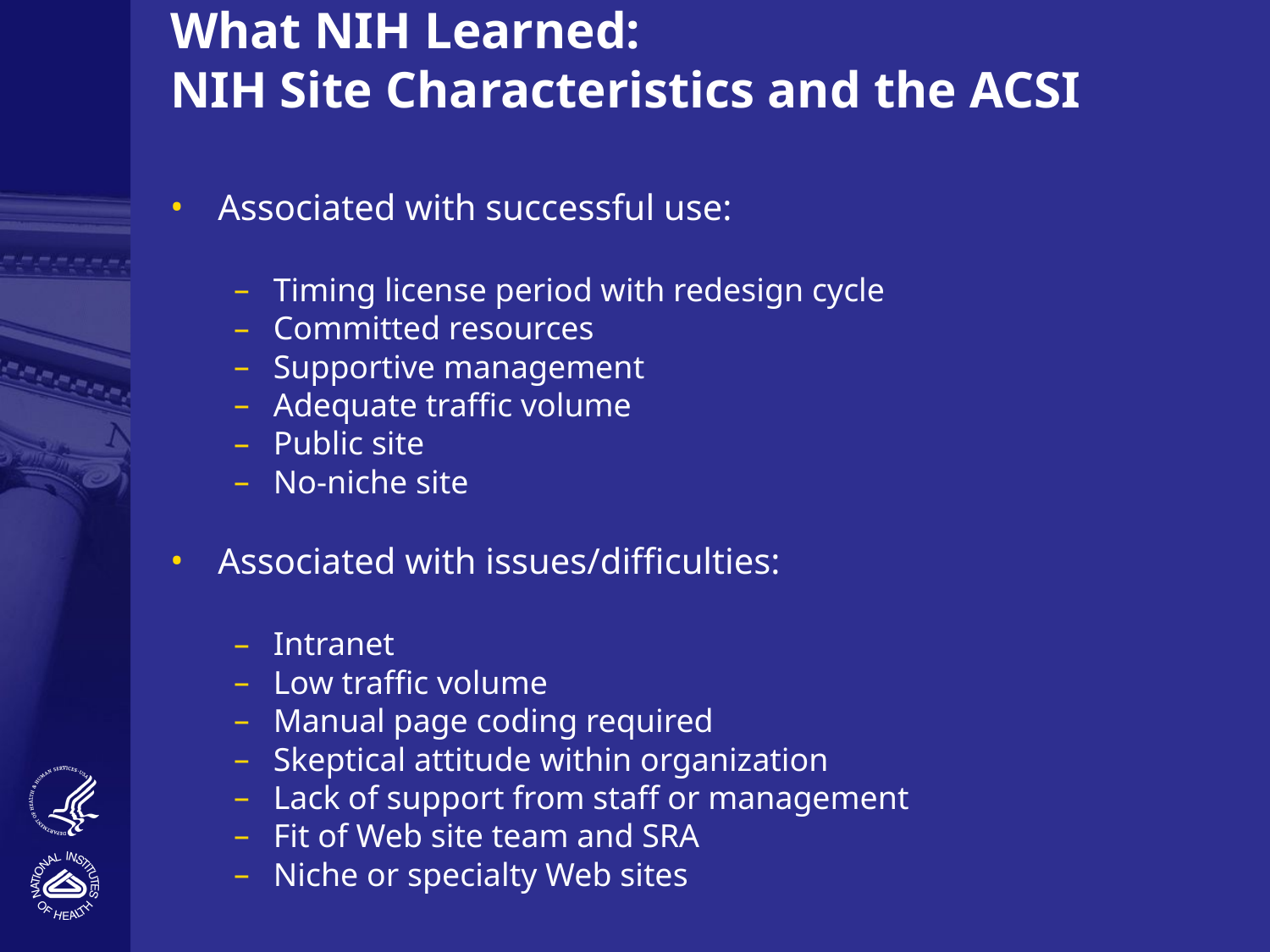

# What NIH Learned: NIH Site Characteristics and the ACSI
Associated with successful use:
Timing license period with redesign cycle
Committed resources
Supportive management
Adequate traffic volume
Public site
No-niche site
Associated with issues/difficulties:
Intranet
Low traffic volume
Manual page coding required
Skeptical attitude within organization
Lack of support from staff or management
Fit of Web site team and SRA
Niche or specialty Web sites

## Slide 31
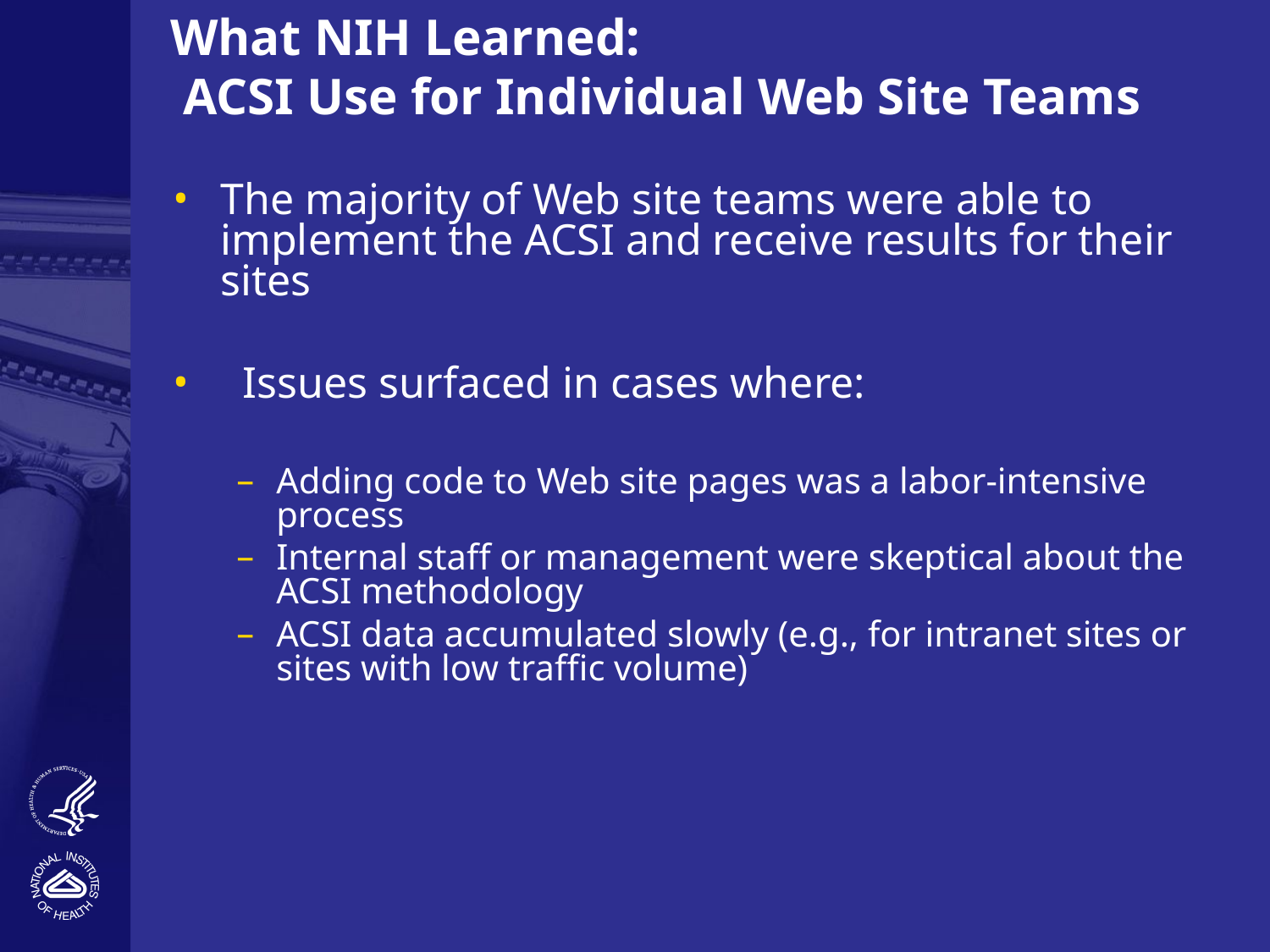

# What NIH Learned: ACSI Use for Individual Web Site Teams
The majority of Web site teams were able to implement the ACSI and receive results for their sites
 Issues surfaced in cases where:
Adding code to Web site pages was a labor-intensive process
Internal staff or management were skeptical about the ACSI methodology
ACSI data accumulated slowly (e.g., for intranet sites or sites with low traffic volume)

## Slide 32
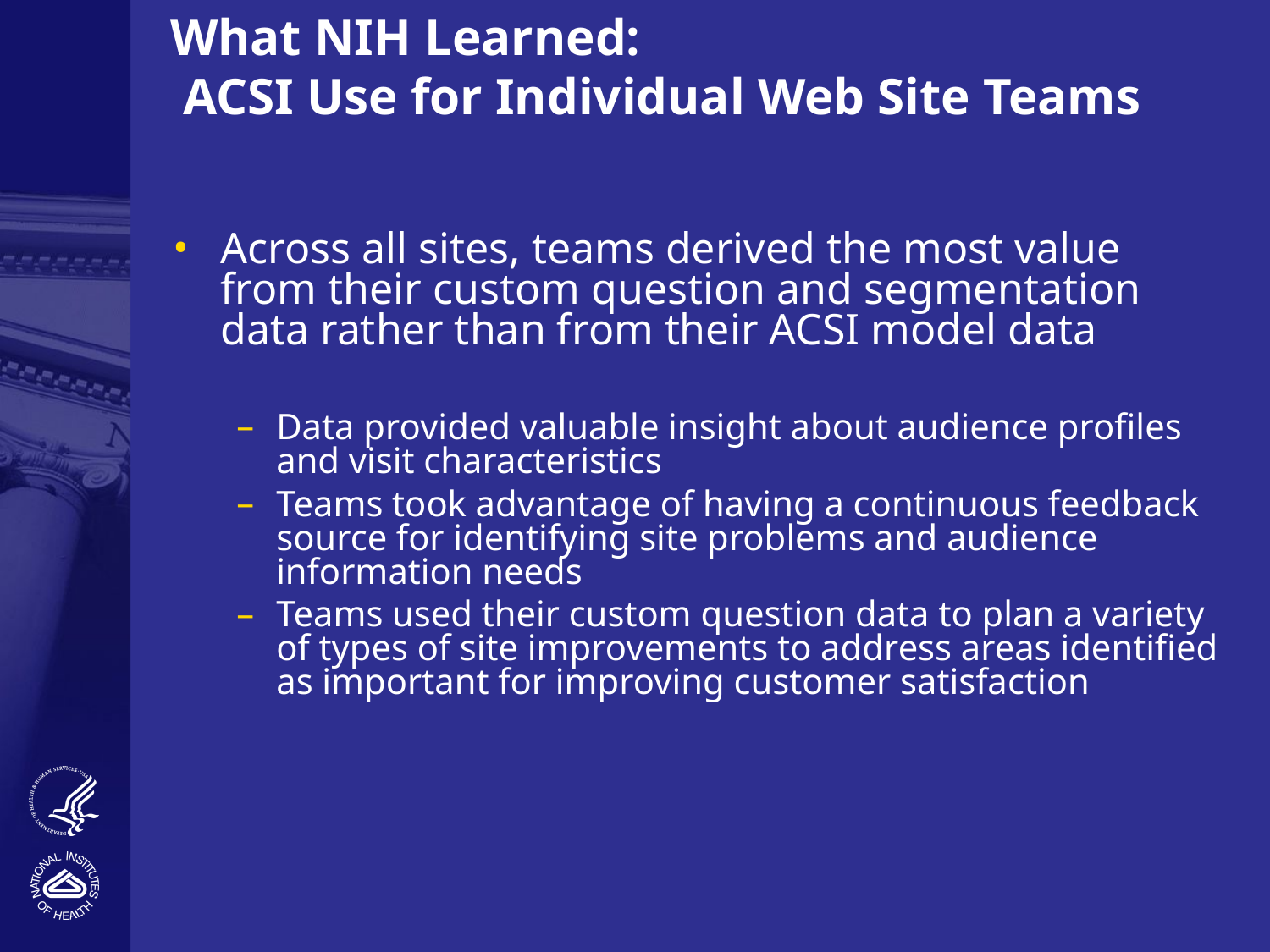

# What NIH Learned: ACSI Use for Individual Web Site Teams
Across all sites, teams derived the most value from their custom question and segmentation data rather than from their ACSI model data
Data provided valuable insight about audience profiles and visit characteristics
Teams took advantage of having a continuous feedback source for identifying site problems and audience information needs
Teams used their custom question data to plan a variety of types of site improvements to address areas identified as important for improving customer satisfaction

## Slide 33
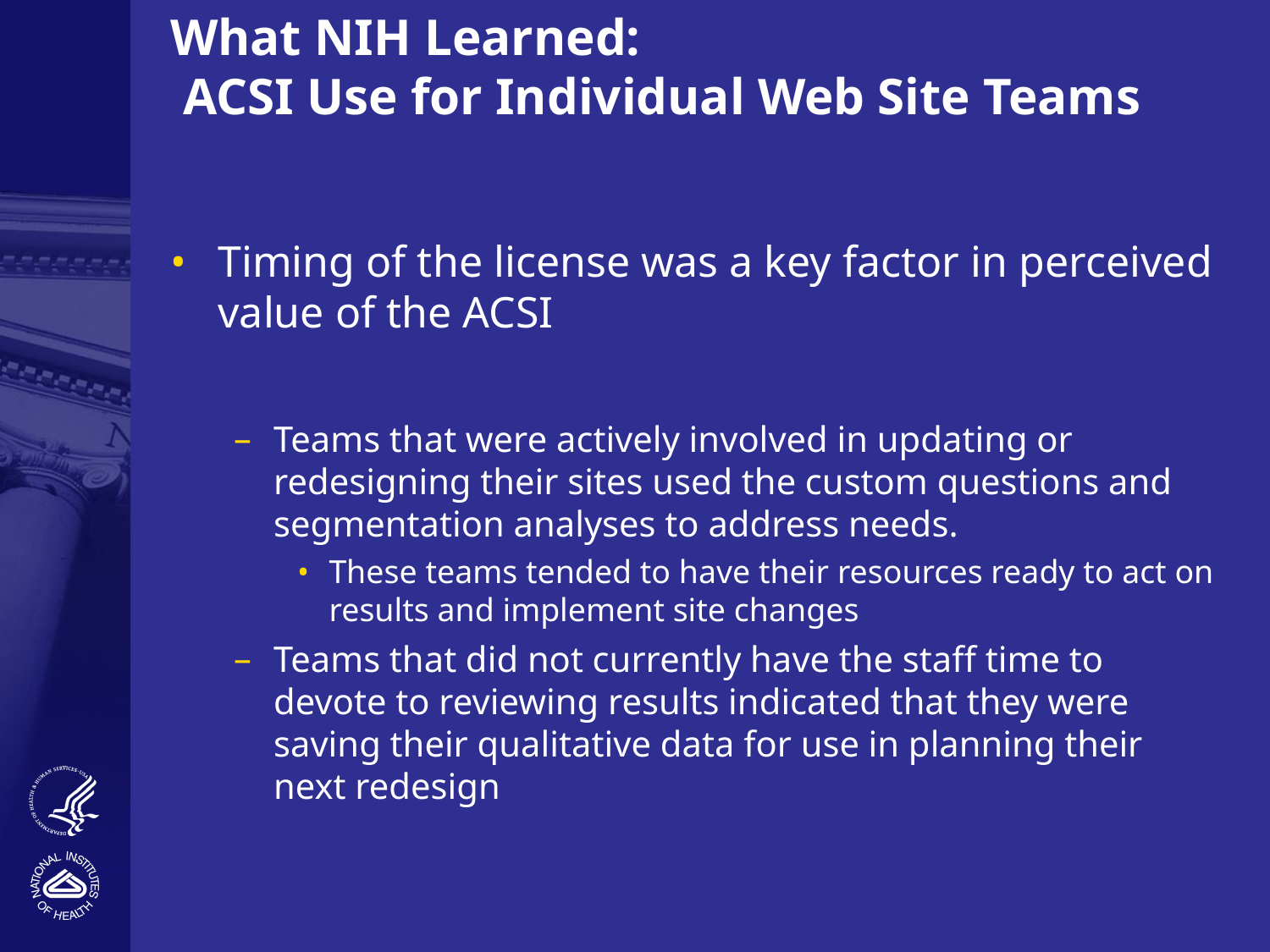

# What NIH Learned: ACSI Use for Individual Web Site Teams
Timing of the license was a key factor in perceived value of the ACSI
Teams that were actively involved in updating or redesigning their sites used the custom questions and segmentation analyses to address needs.
These teams tended to have their resources ready to act on results and implement site changes
Teams that did not currently have the staff time to devote to reviewing results indicated that they were saving their qualitative data for use in planning their next redesign

## Slide 34
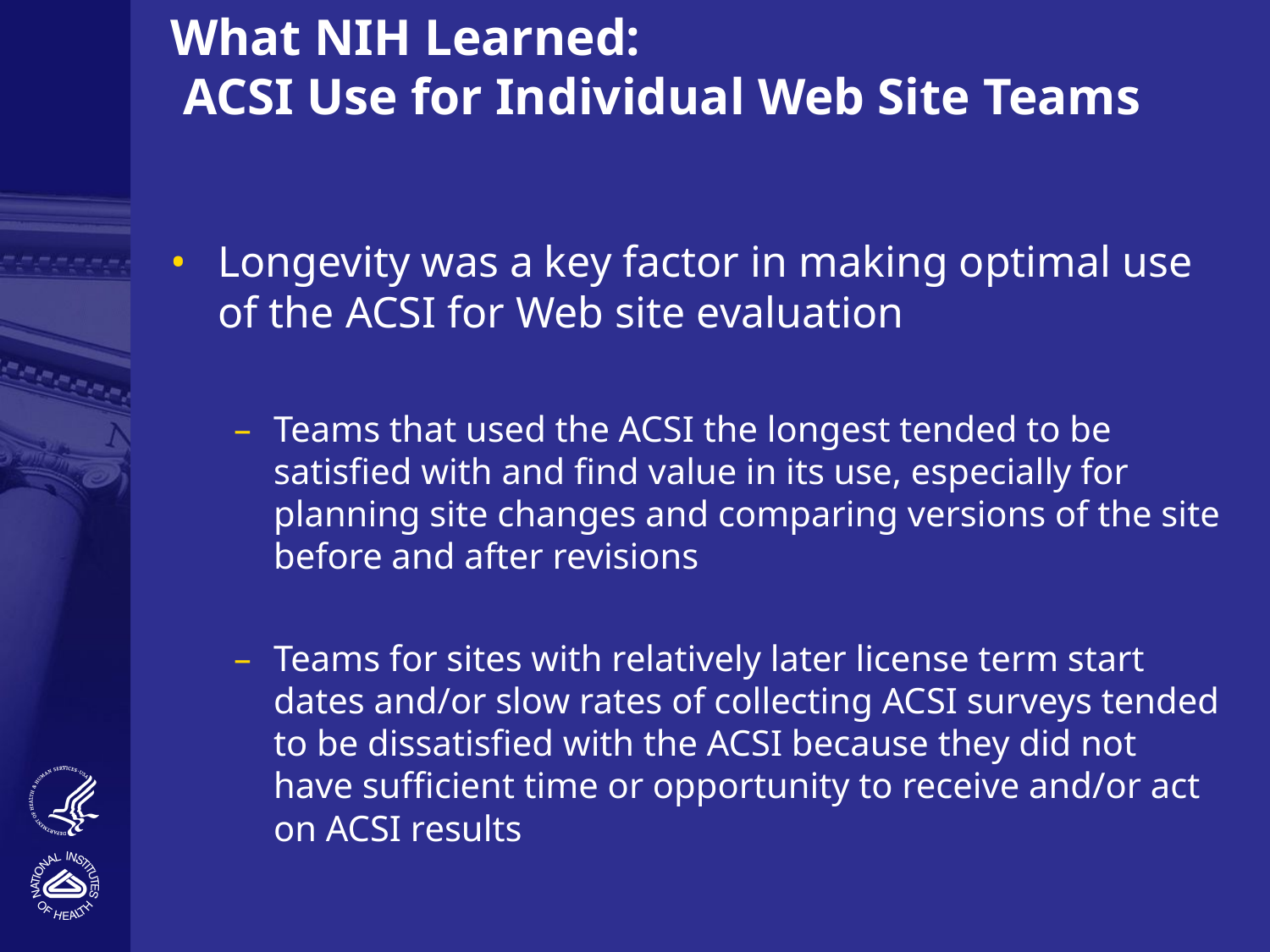

# What NIH Learned: ACSI Use for Individual Web Site Teams
Longevity was a key factor in making optimal use of the ACSI for Web site evaluation
Teams that used the ACSI the longest tended to be satisfied with and find value in its use, especially for planning site changes and comparing versions of the site before and after revisions
Teams for sites with relatively later license term start dates and/or slow rates of collecting ACSI surveys tended to be dissatisfied with the ACSI because they did not have sufficient time or opportunity to receive and/or act on ACSI results

## Slide 35
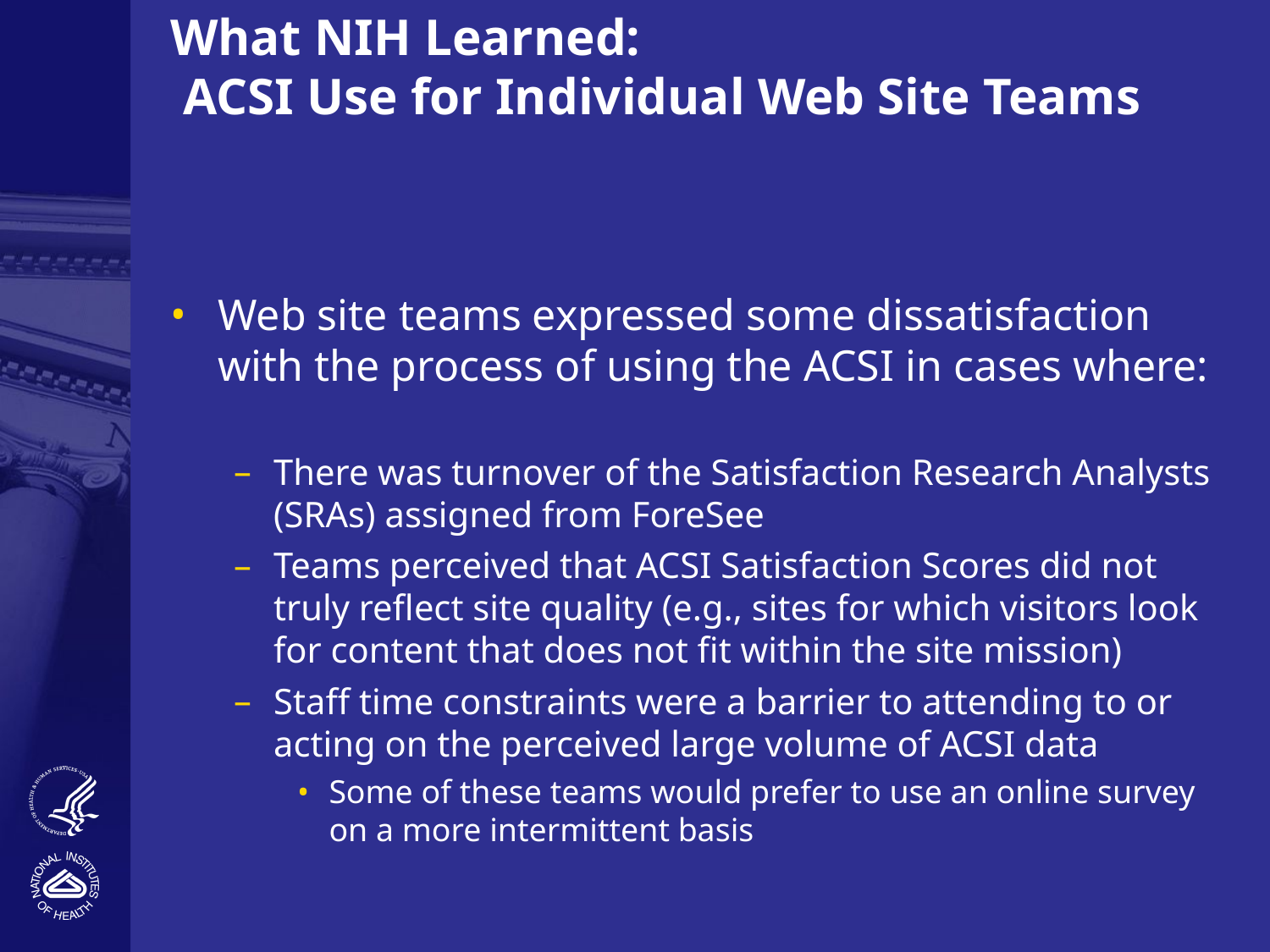

# What NIH Learned: ACSI Use for Individual Web Site Teams
Web site teams expressed some dissatisfaction with the process of using the ACSI in cases where:
There was turnover of the Satisfaction Research Analysts (SRAs) assigned from ForeSee
Teams perceived that ACSI Satisfaction Scores did not truly reflect site quality (e.g., sites for which visitors look for content that does not fit within the site mission)
Staff time constraints were a barrier to attending to or acting on the perceived large volume of ACSI data
Some of these teams would prefer to use an online survey on a more intermittent basis

## Slide 36
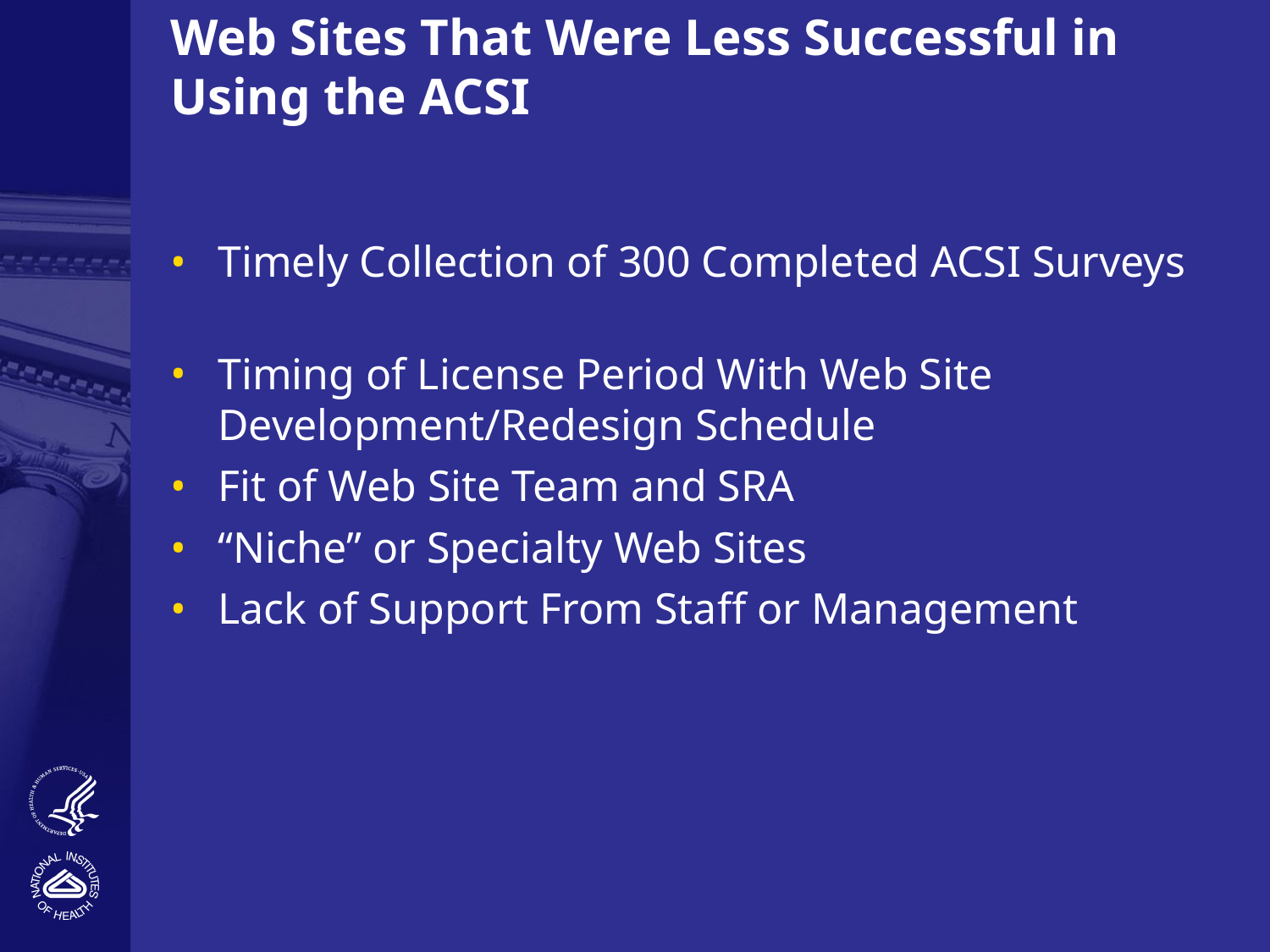

# Web Sites That Were Less Successful in Using the ACSI
Timely Collection of 300 Completed ACSI Surveys
Timing of License Period With Web Site Development/Redesign Schedule
Fit of Web Site Team and SRA
“Niche” or Specialty Web Sites
Lack of Support From Staff or Management

## Slide 37
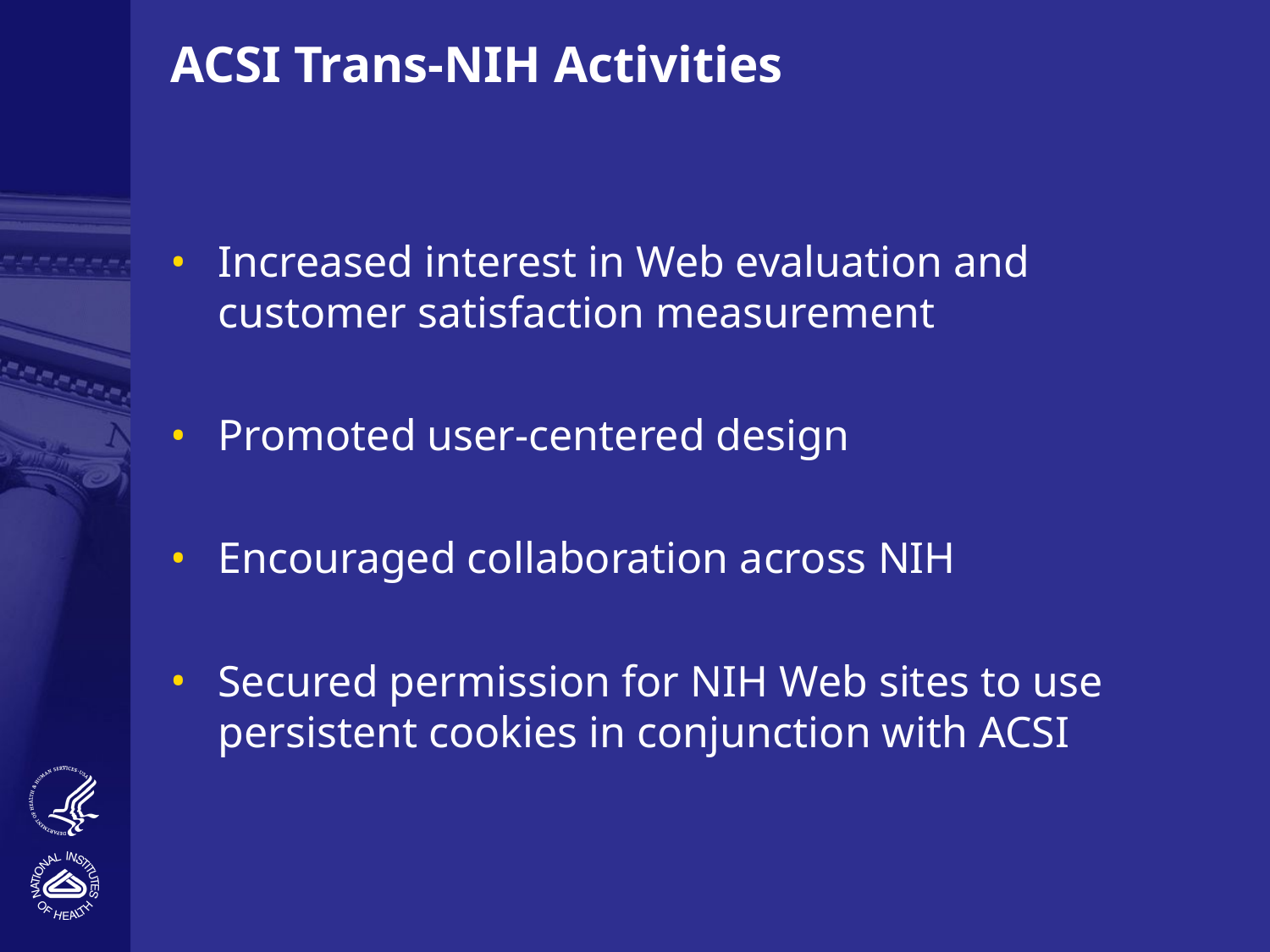

# ACSI Trans-NIH Activities
Increased interest in Web evaluation and customer satisfaction measurement
Promoted user-centered design
Encouraged collaboration across NIH
Secured permission for NIH Web sites to use persistent cookies in conjunction with ACSI

## Slide 38
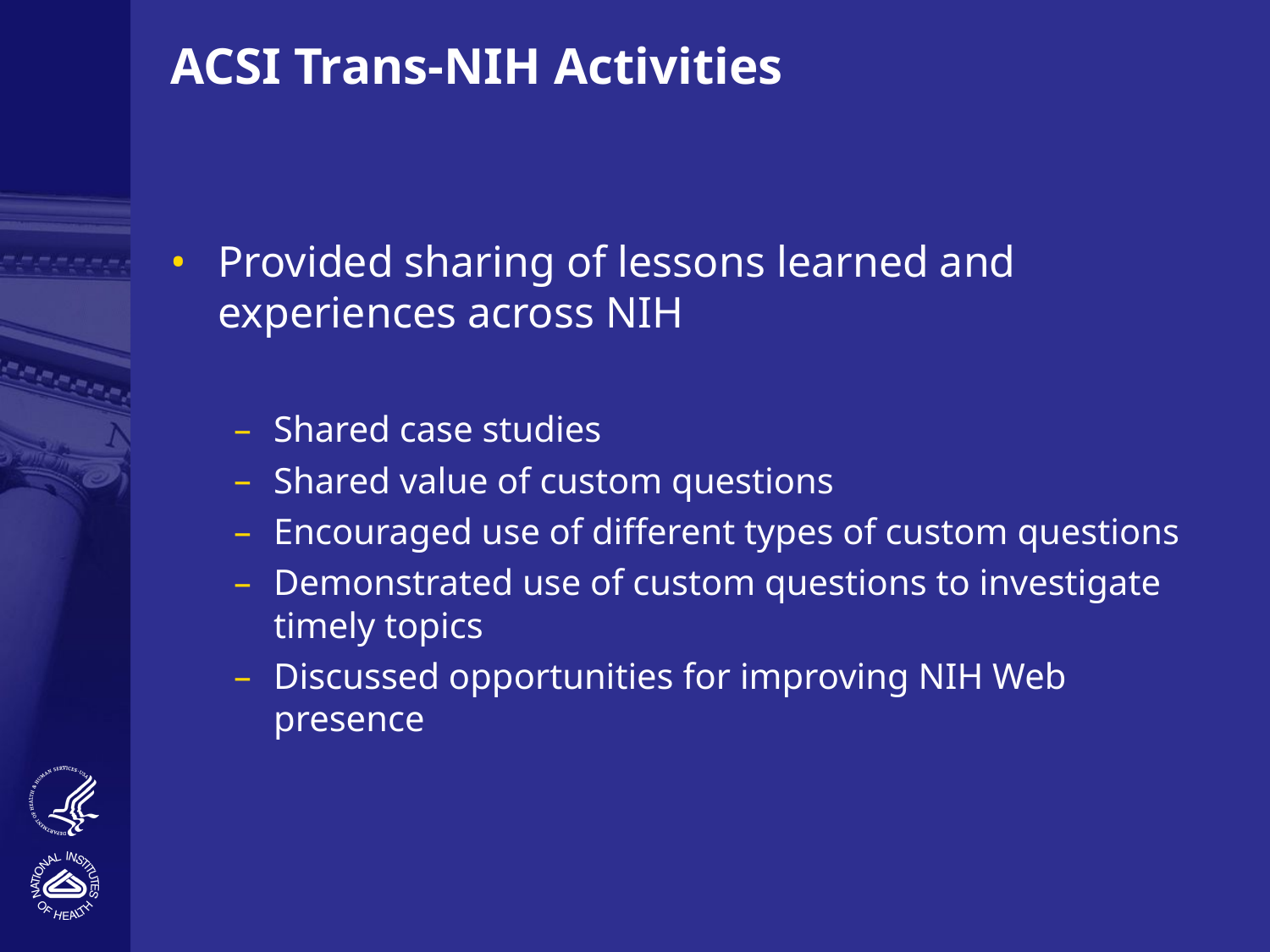

# ACSI Trans-NIH Activities
Provided sharing of lessons learned and experiences across NIH
Shared case studies
Shared value of custom questions
Encouraged use of different types of custom questions
Demonstrated use of custom questions to investigate timely topics
Discussed opportunities for improving NIH Web presence

## Slide 39
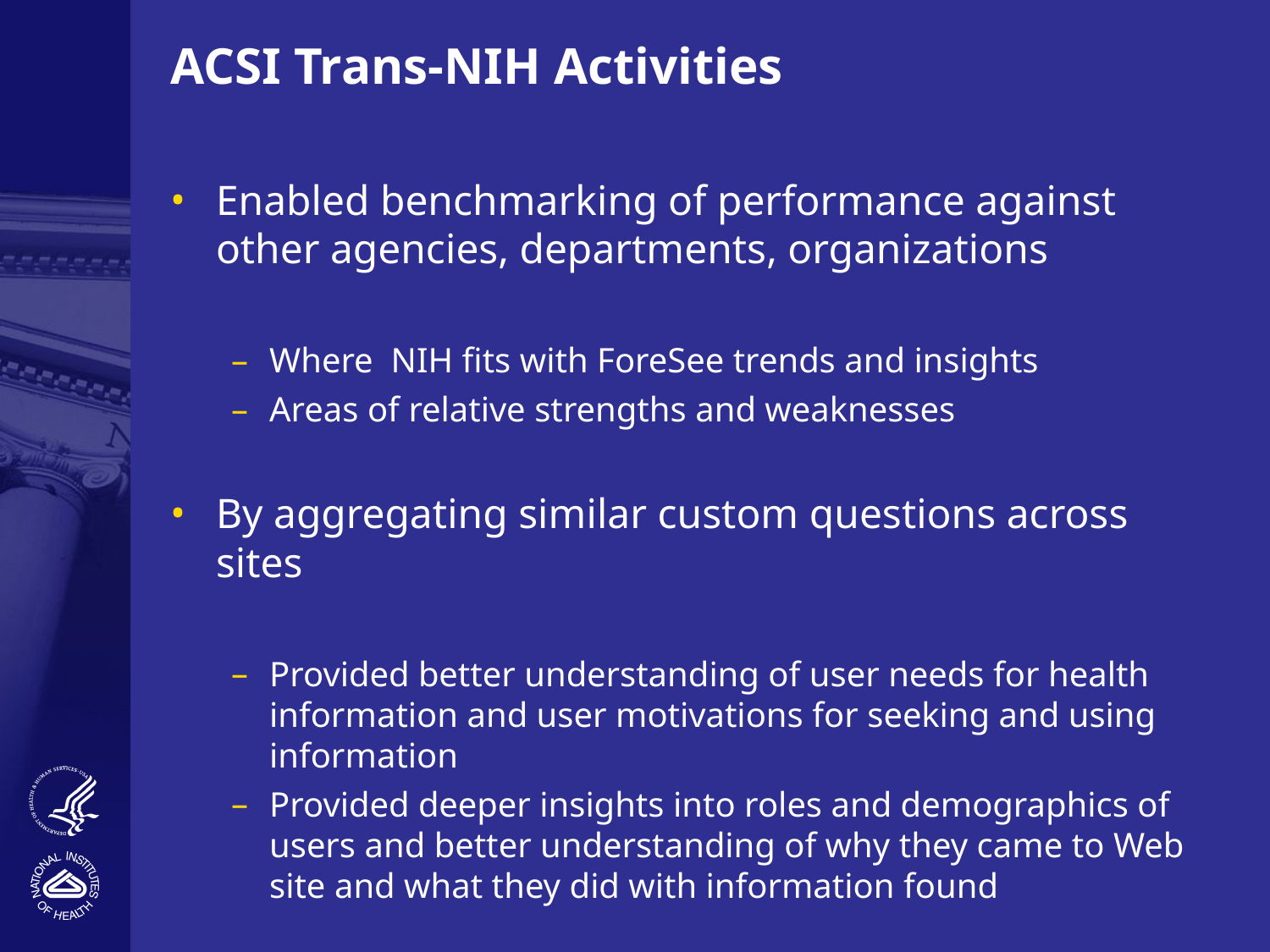

# ACSI Trans-NIH Activities
Enabled benchmarking of performance against other agencies, departments, organizations
Where NIH fits with ForeSee trends and insights
Areas of relative strengths and weaknesses
By aggregating similar custom questions across sites
Provided better understanding of user needs for health information and user motivations for seeking and using information
Provided deeper insights into roles and demographics of users and better understanding of why they came to Web site and what they did with information found

## Slide 40
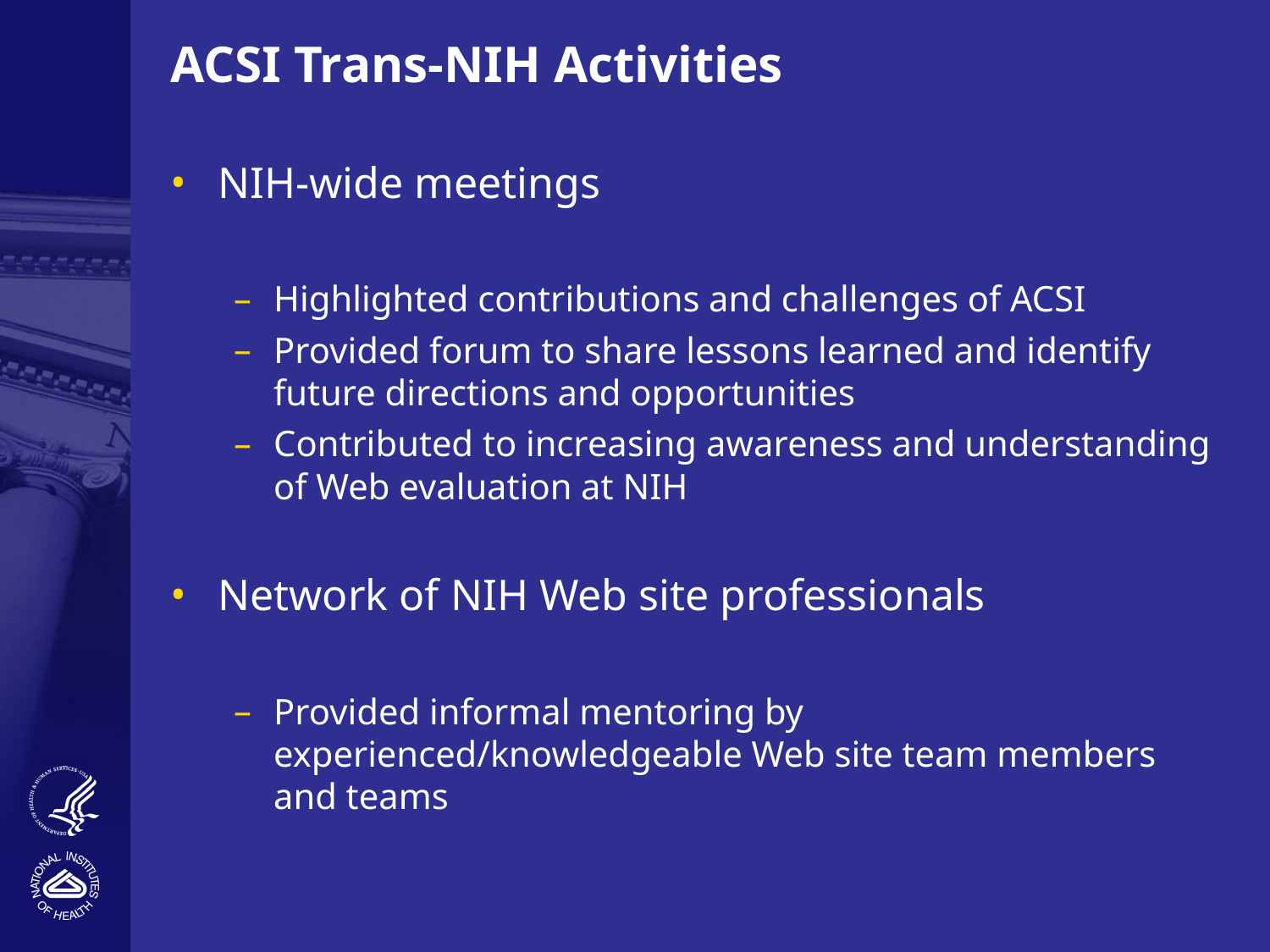

# ACSI Trans-NIH Activities
NIH-wide meetings
Highlighted contributions and challenges of ACSI
Provided forum to share lessons learned and identify future directions and opportunities
Contributed to increasing awareness and understanding of Web evaluation at NIH
Network of NIH Web site professionals
Provided informal mentoring by experienced/knowledgeable Web site team members and teams

## Slide 41
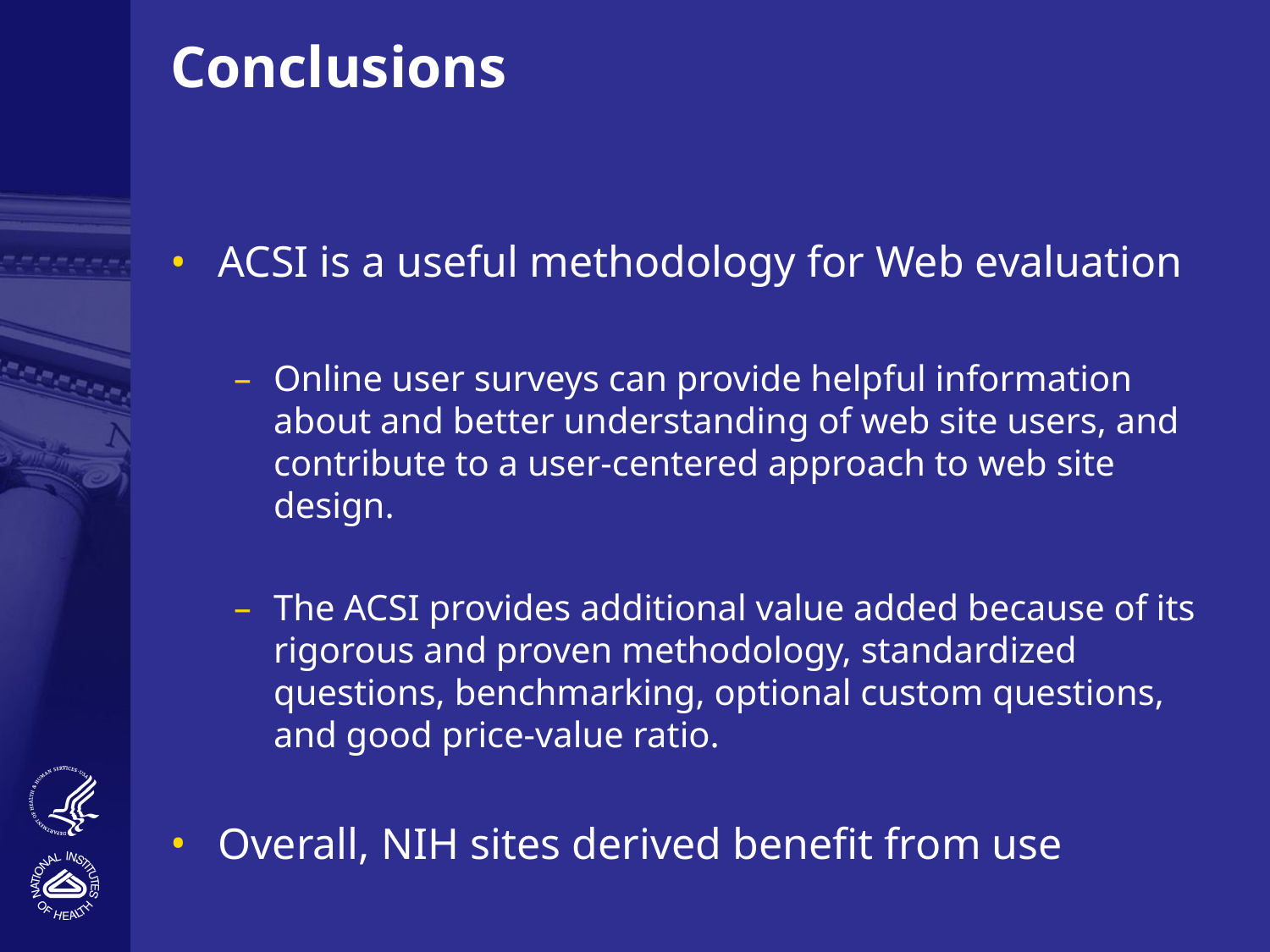

# Conclusions
ACSI is a useful methodology for Web evaluation
Online user surveys can provide helpful information about and better understanding of web site users, and contribute to a user-centered approach to web site design.
The ACSI provides additional value added because of its rigorous and proven methodology, standardized questions, benchmarking, optional custom questions, and good price-value ratio.
Overall, NIH sites derived benefit from use

## Slide 42
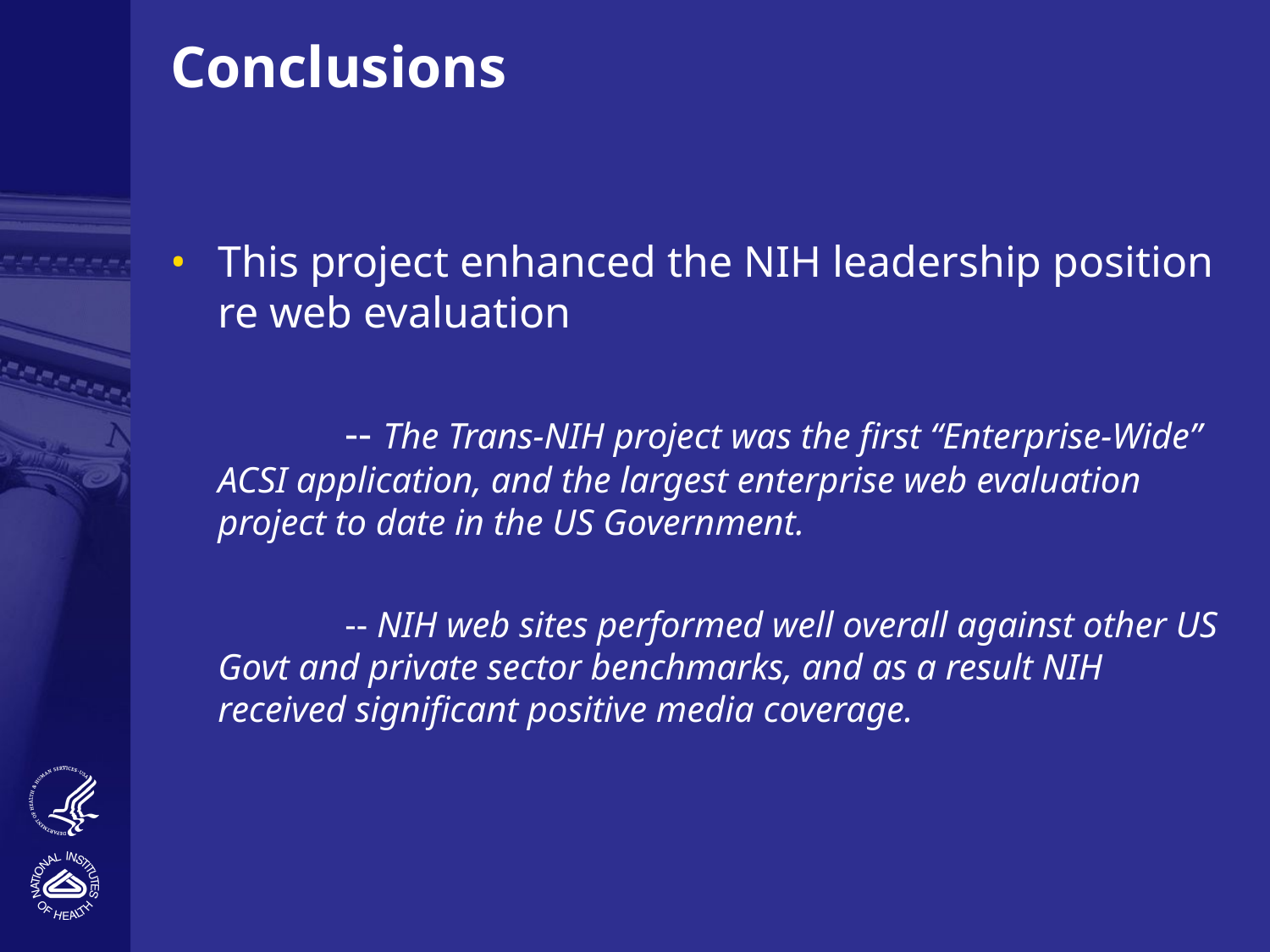

# Conclusions
This project enhanced the NIH leadership position re web evaluation
	-- The Trans-NIH project was the first “Enterprise-Wide” ACSI application, and the largest enterprise web evaluation project to date in the US Government.
	-- NIH web sites performed well overall against other US Govt and private sector benchmarks, and as a result NIH received significant positive media coverage.

## Slide 43
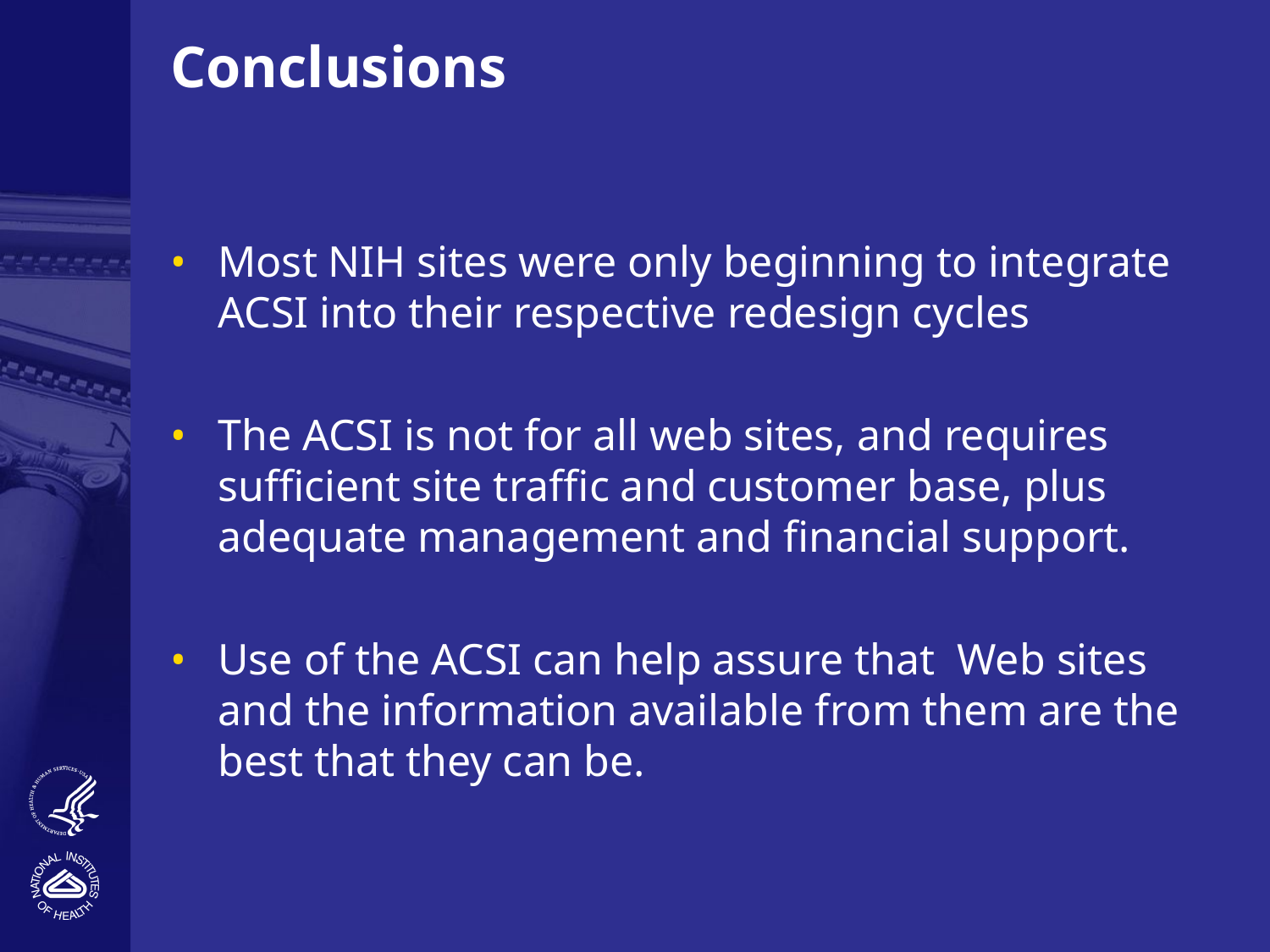

# Conclusions
Most NIH sites were only beginning to integrate ACSI into their respective redesign cycles
The ACSI is not for all web sites, and requires sufficient site traffic and customer base, plus adequate management and financial support.
Use of the ACSI can help assure that Web sites and the information available from them are the best that they can be.

## Slide 44
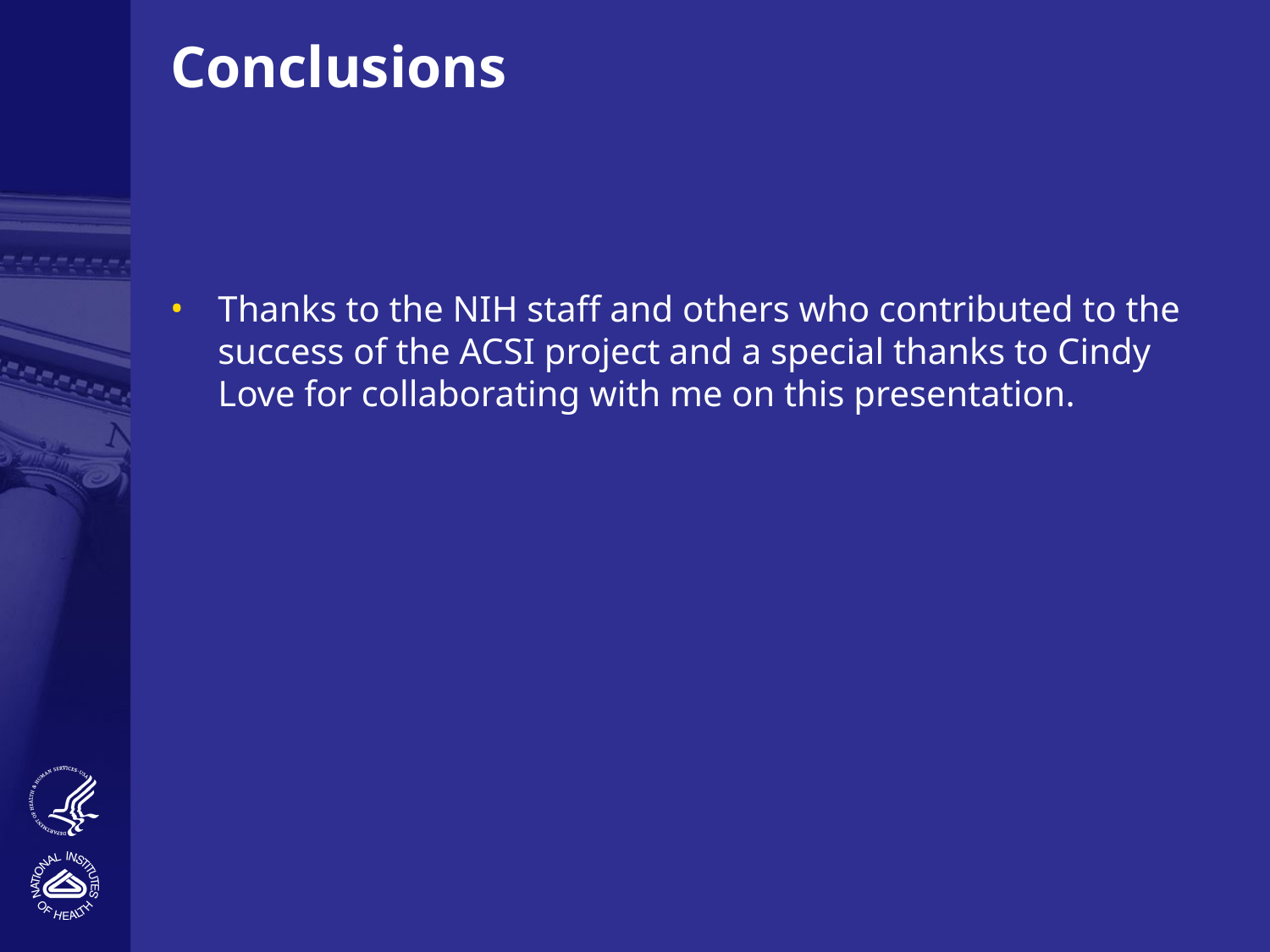

# Conclusions
Thanks to the NIH staff and others who contributed to the success of the ACSI project and a special thanks to Cindy Love for collaborating with me on this presentation.

## Slide 45
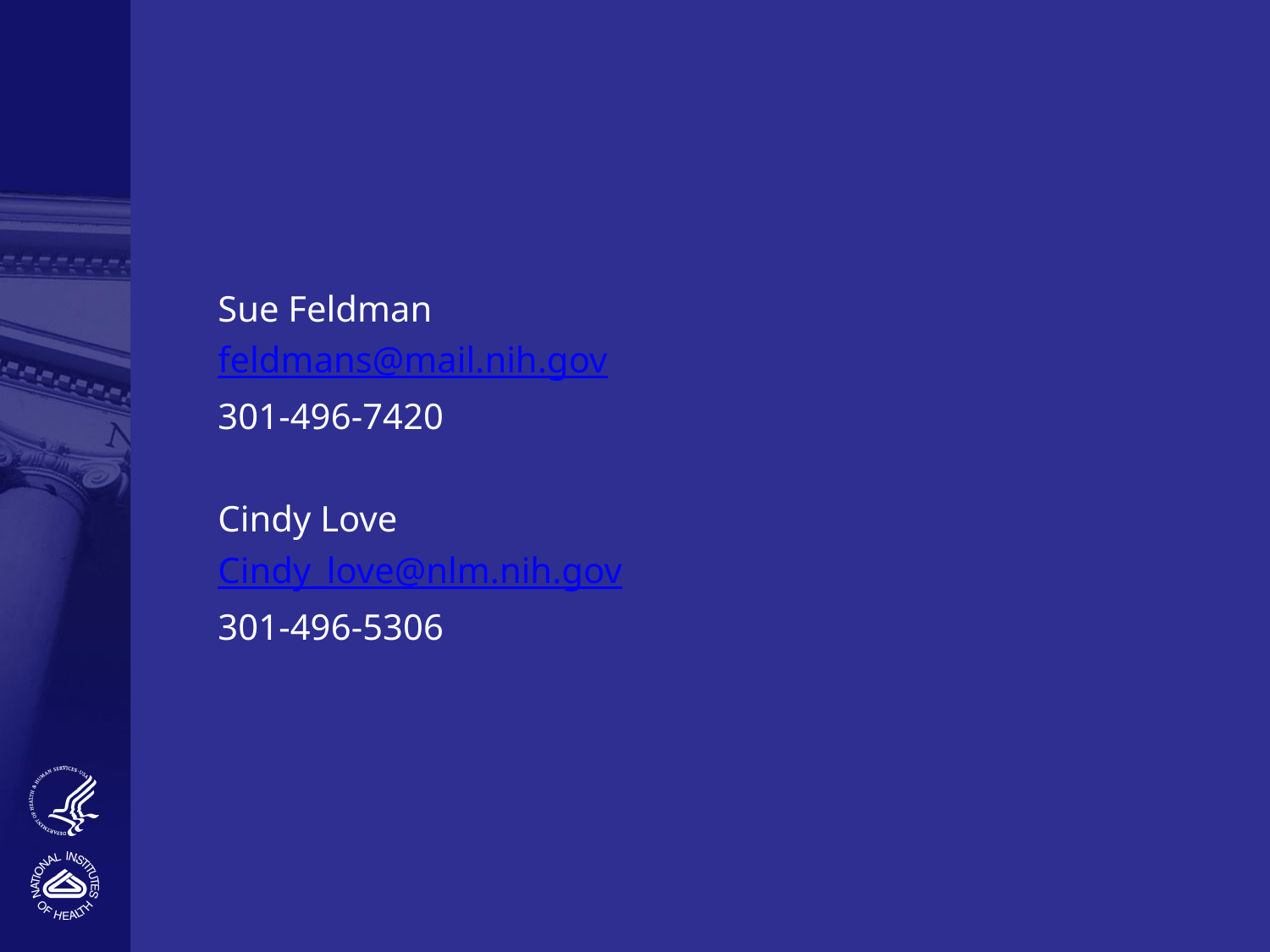

# Sue Feldman
feldmans@mail.nih.gov
301-496-7420
Cindy Love
Cindy_love@nlm.nih.gov
301-496-5306

## Slide 46
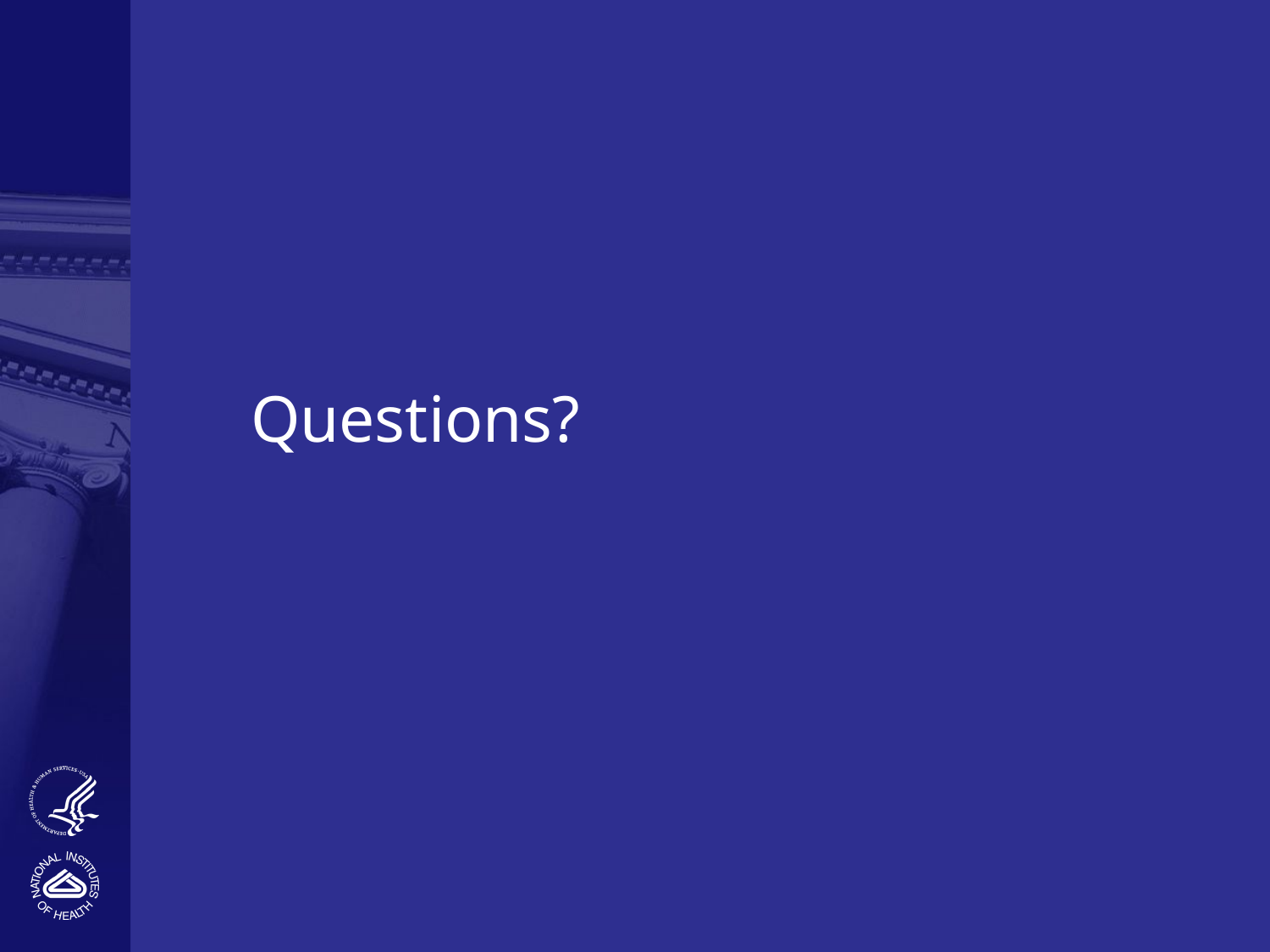

# Questions?
