# Supplementary material for: Web Evaluation at the US National Institutes of Health: Use of the American Customer Satisfaction Index Online Customer Survey
Source: J Med Internet Res. 2008 Feb 15;10(1):e4. doi: 10.2196/jmir.944 (PMC2483849; doi:10.2196/jmir.944)
Supplement: Supplementary file 5 [file jmir_v10i1e4_app5.ppt]

## Slide 1
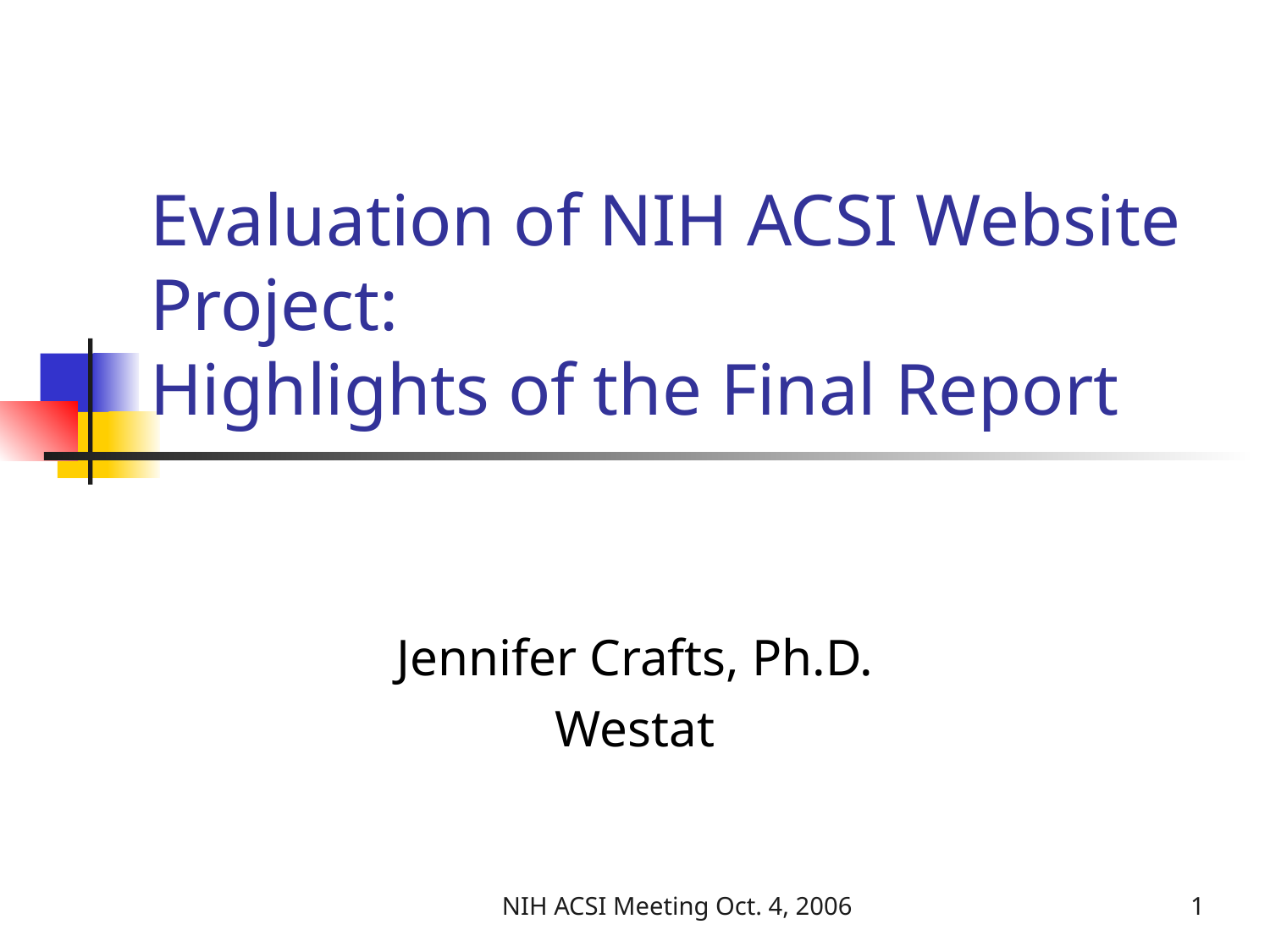

# Evaluation of NIH ACSI Website Project: Highlights of the Final Report
Jennifer Crafts, Ph.D.
Westat
NIH ACSI Meeting Oct. 4, 2006
1

## Slide 2
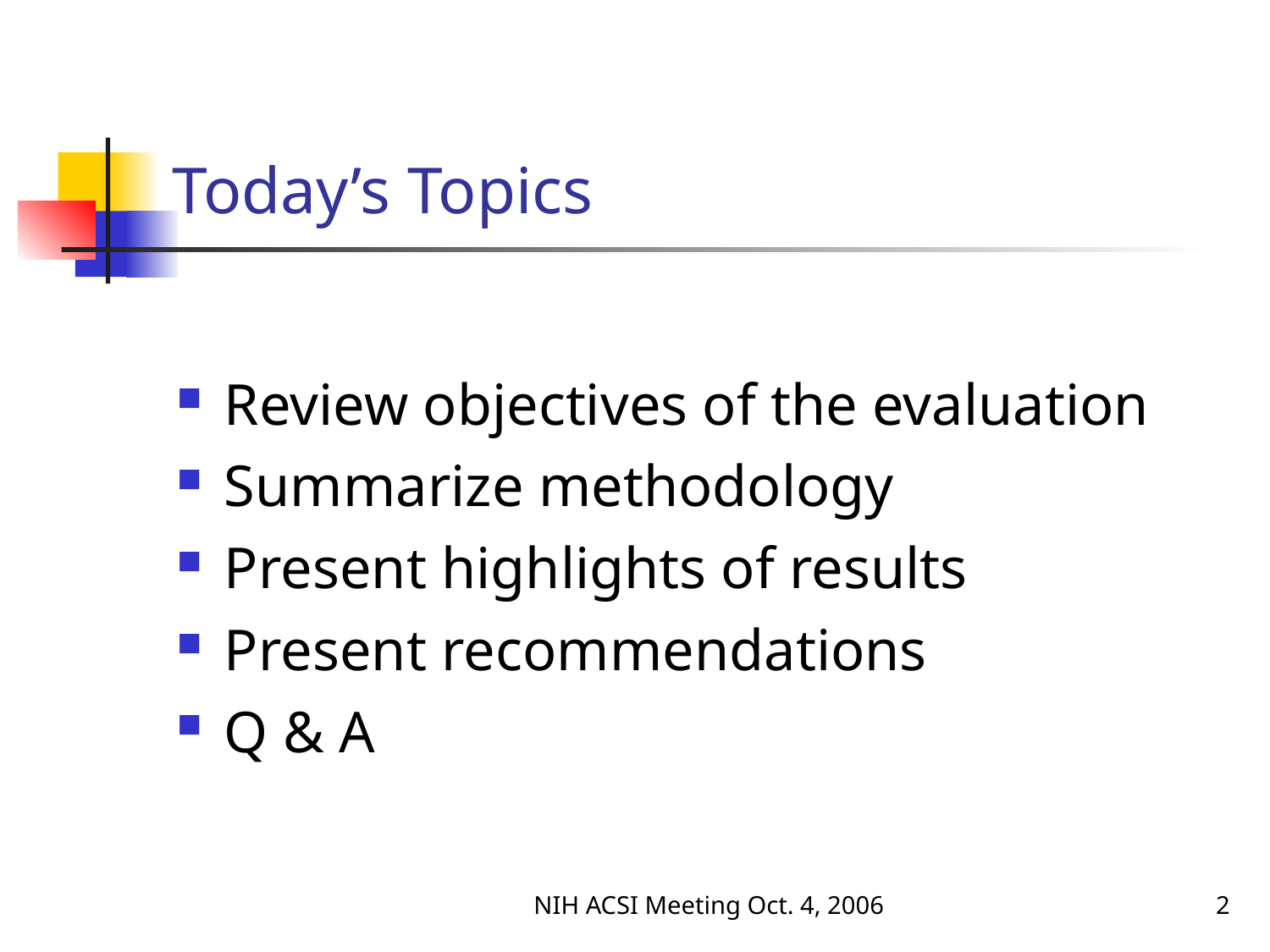

# Today’s Topics
Review objectives of the evaluation
Summarize methodology
Present highlights of results
Present recommendations
Q & A
NIH ACSI Meeting Oct. 4, 2006
2

## Slide 3
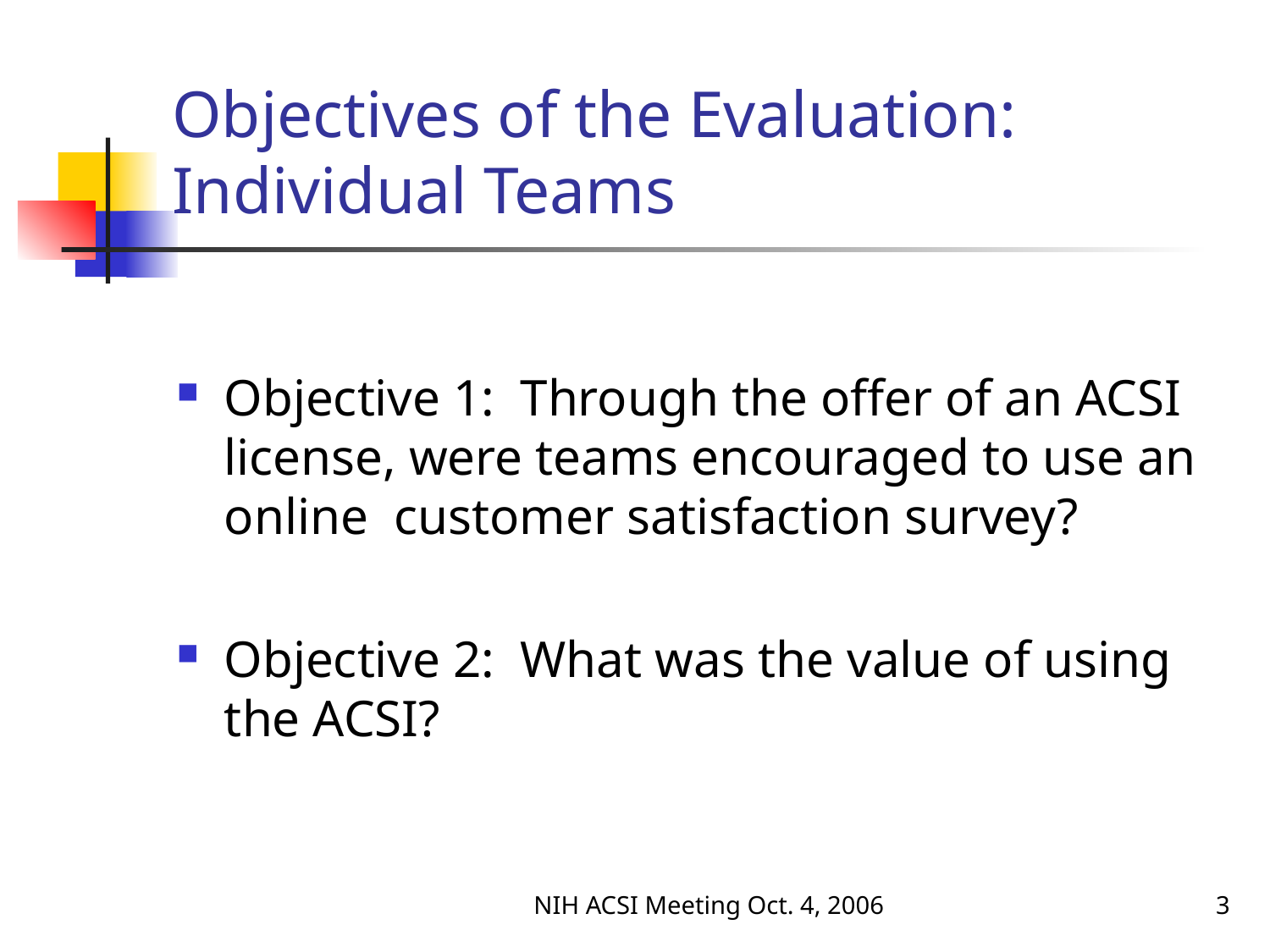

# Objectives of the Evaluation: Individual Teams
Objective 1: Through the offer of an ACSI license, were teams encouraged to use an online customer satisfaction survey?
Objective 2: What was the value of using the ACSI?
NIH ACSI Meeting Oct. 4, 2006
3

## Slide 4
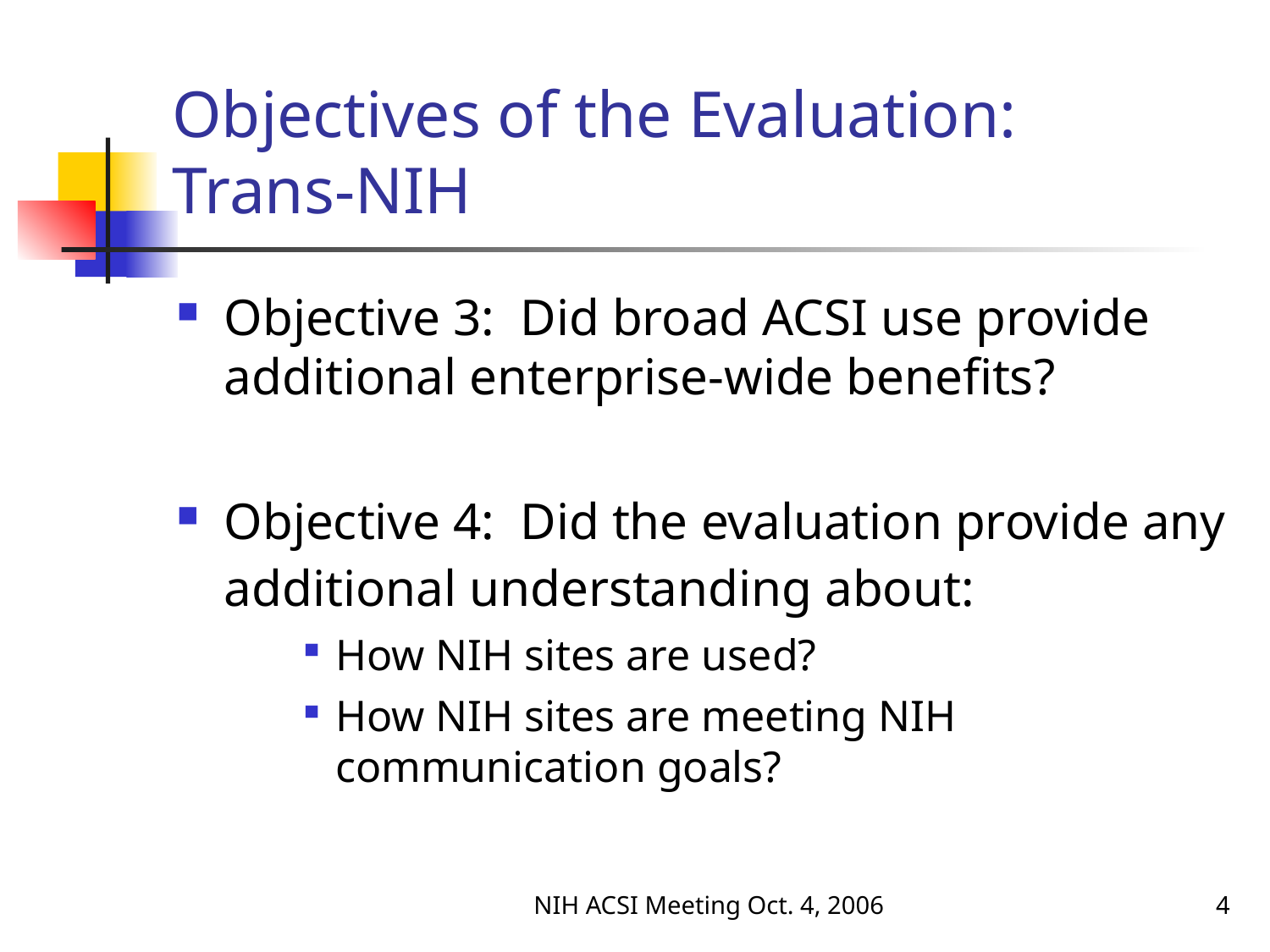

# Objectives of the Evaluation: Trans-NIH
Objective 3: Did broad ACSI use provide additional enterprise-wide benefits?
Objective 4: Did the evaluation provide any additional understanding about:
How NIH sites are used?
How NIH sites are meeting NIH communication goals?
NIH ACSI Meeting Oct. 4, 2006
4

## Slide 5
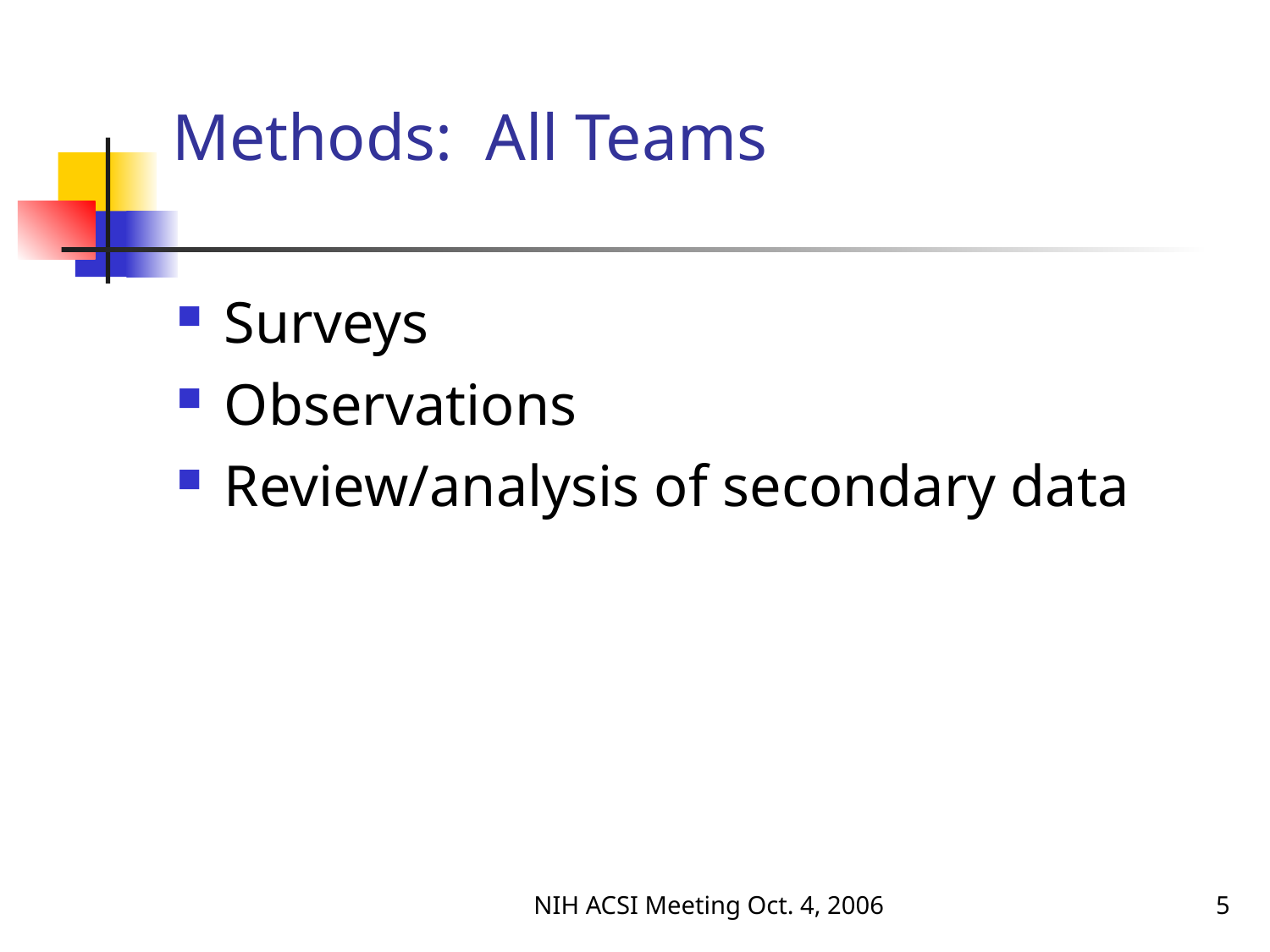

# Methods: All Teams
Surveys
Observations
Review/analysis of secondary data
NIH ACSI Meeting Oct. 4, 2006
5

## Slide 6
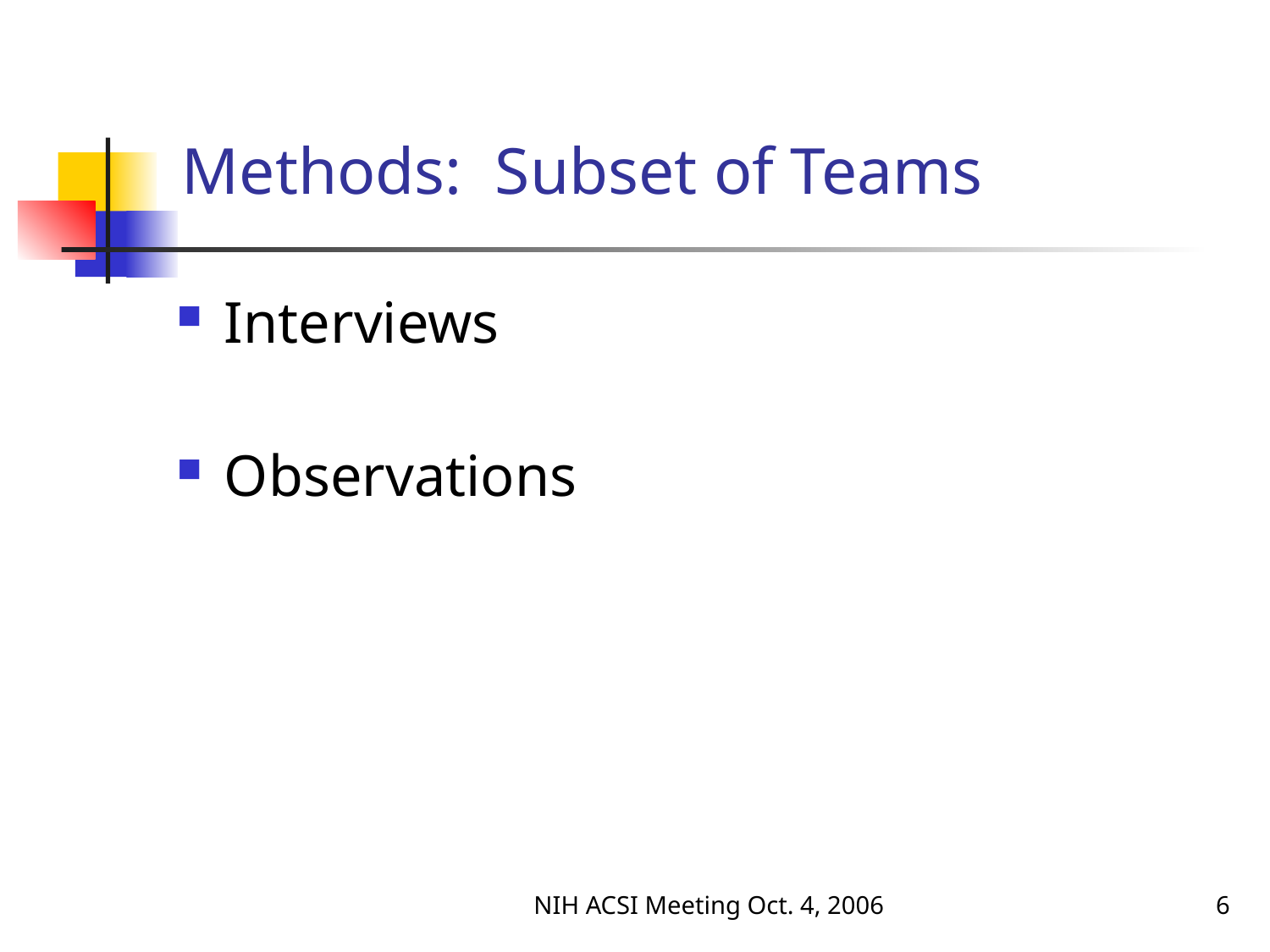

# Methods: Subset of Teams
Interviews
Observations
NIH ACSI Meeting Oct. 4, 2006
6

## Slide 7
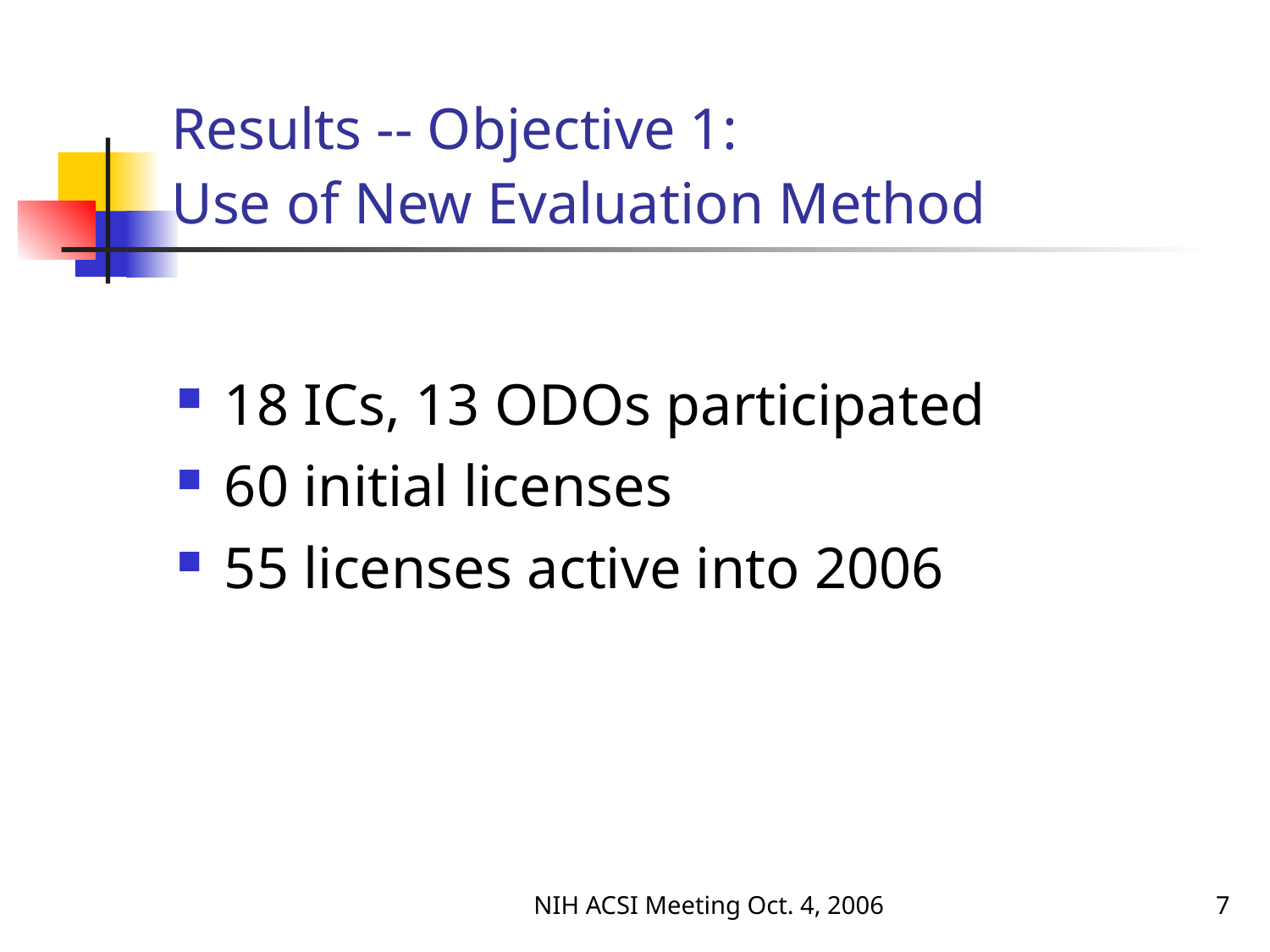

# Results -- Objective 1: Use of New Evaluation Method
18 ICs, 13 ODOs participated
60 initial licenses
55 licenses active into 2006
NIH ACSI Meeting Oct. 4, 2006
7

## Slide 8
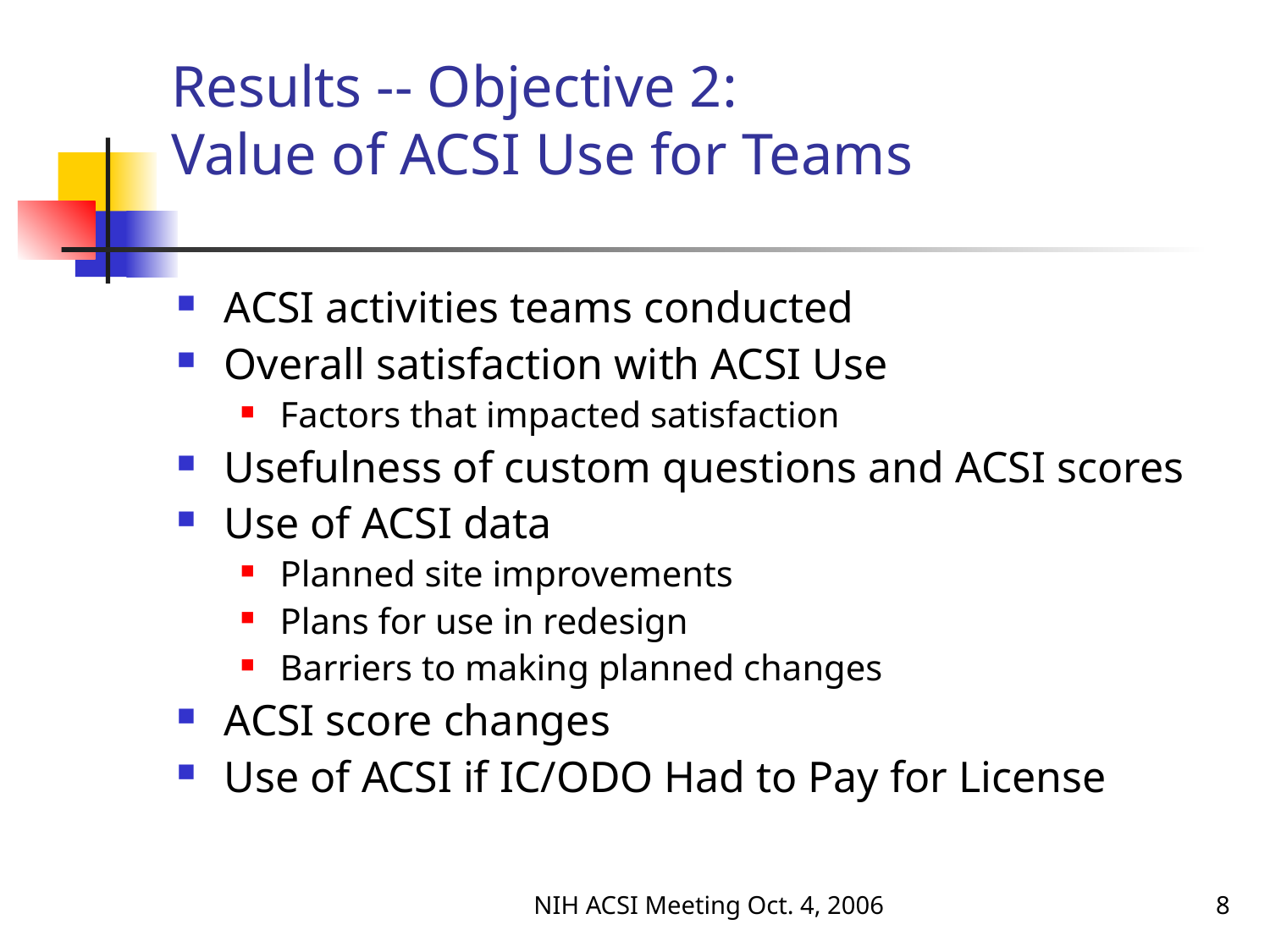

# Results -- Objective 2: Value of ACSI Use for Teams
ACSI activities teams conducted
Overall satisfaction with ACSI Use
Factors that impacted satisfaction
Usefulness of custom questions and ACSI scores
Use of ACSI data
Planned site improvements
Plans for use in redesign
Barriers to making planned changes
ACSI score changes
Use of ACSI if IC/ODO Had to Pay for License
NIH ACSI Meeting Oct. 4, 2006
8

## Slide 9
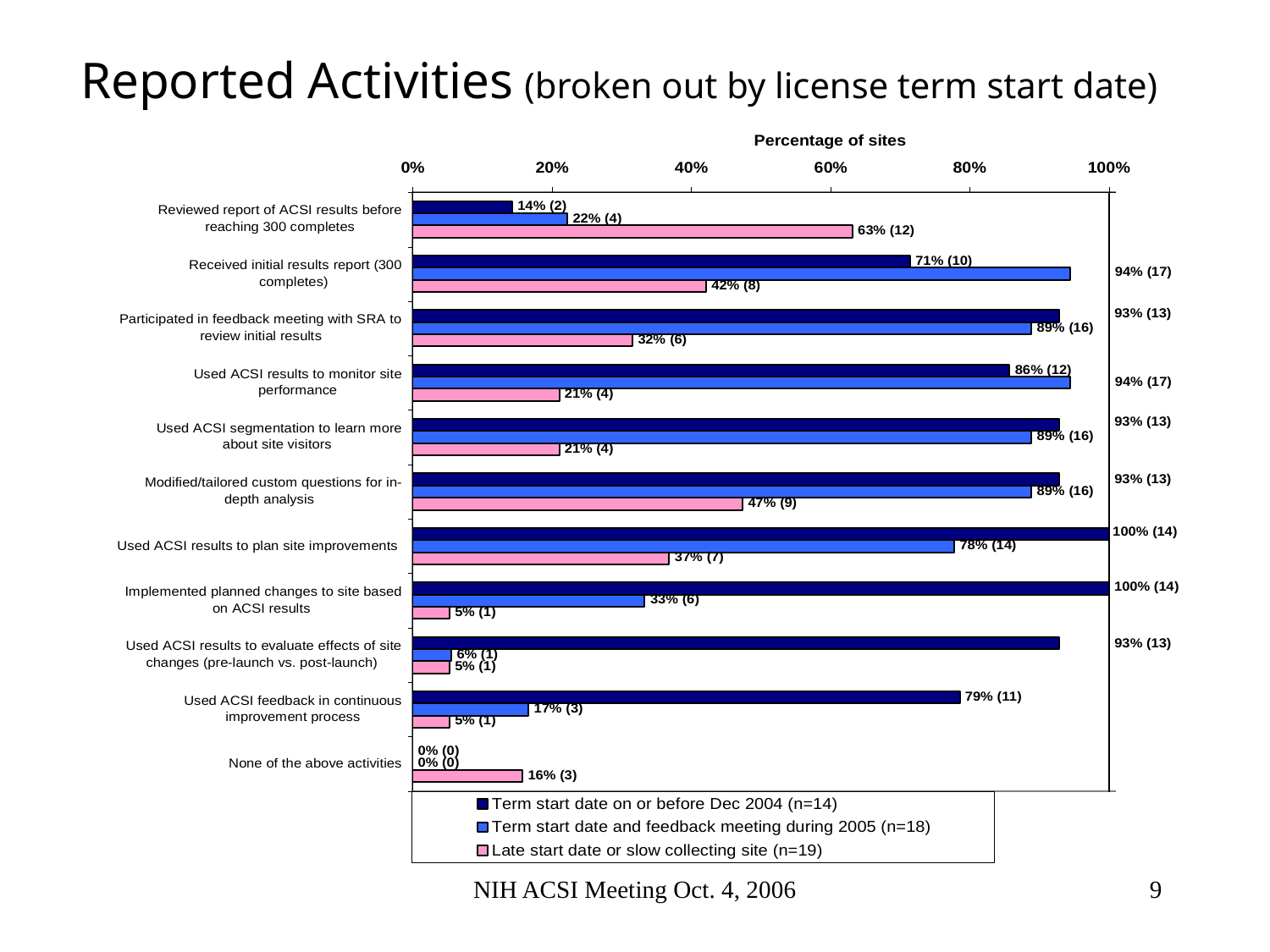

Reported Activities (broken out by license term start date)
NIH ACSI Meeting Oct. 4, 2006
9

## Slide 10
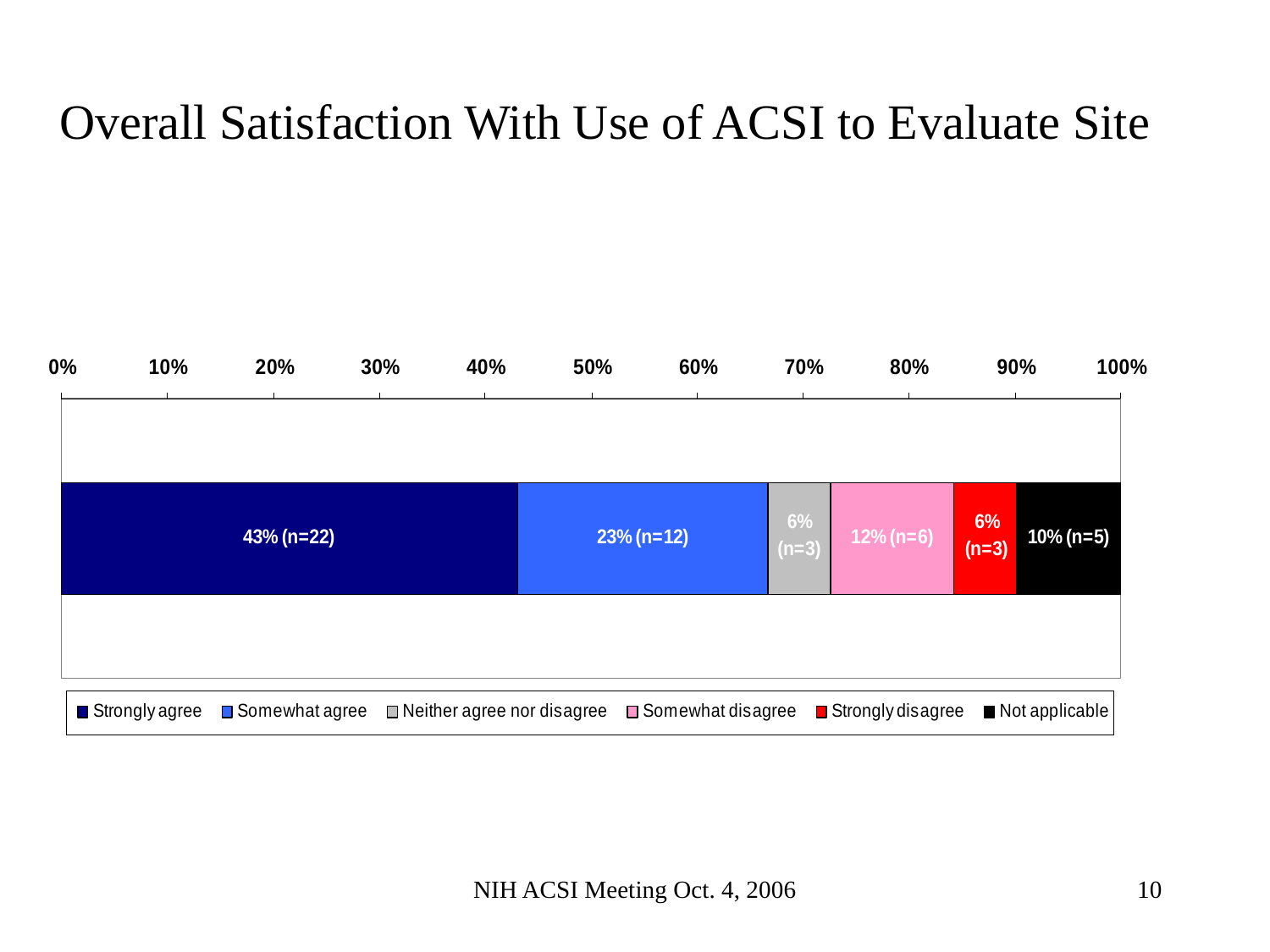

# Overall Satisfaction With Use of ACSI to Evaluate Site
NIH ACSI Meeting Oct. 4, 2006
10

## Slide 11
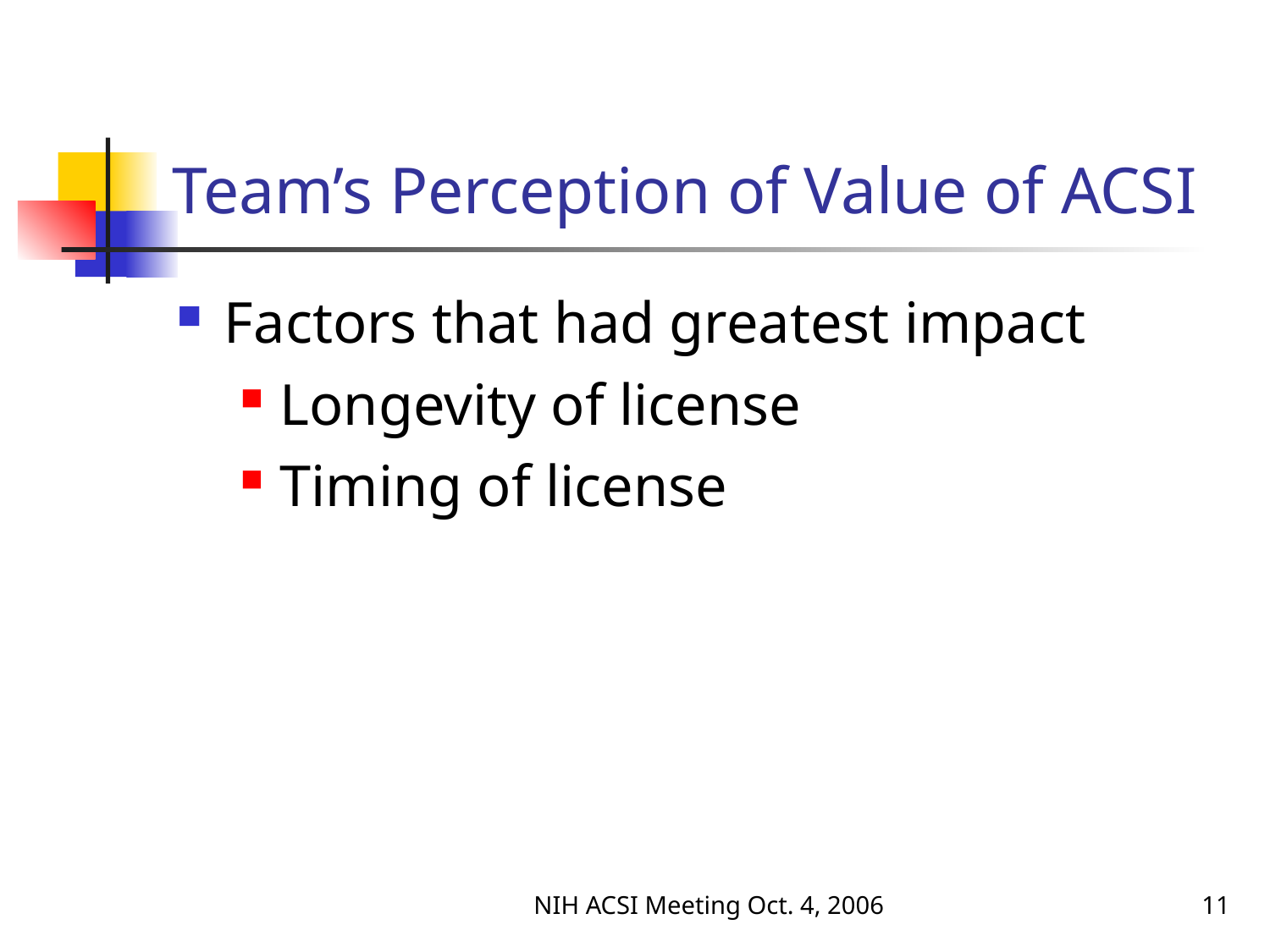

# Team’s Perception of Value of ACSI
Factors that had greatest impact
Longevity of license
Timing of license
NIH ACSI Meeting Oct. 4, 2006
11

## Slide 12
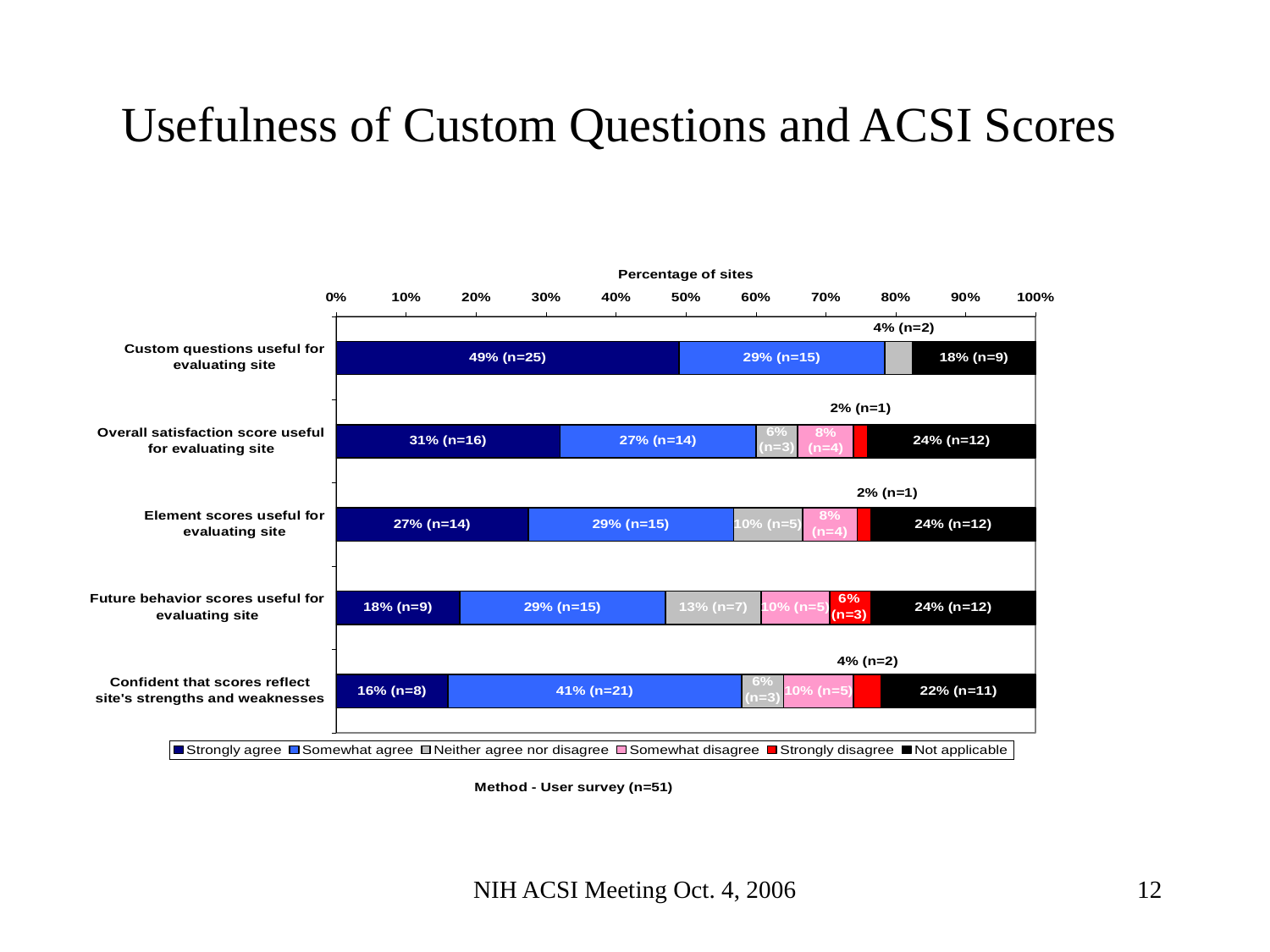

# Usefulness of Custom Questions and ACSI Scores
NIH ACSI Meeting Oct. 4, 2006
12

## Slide 13
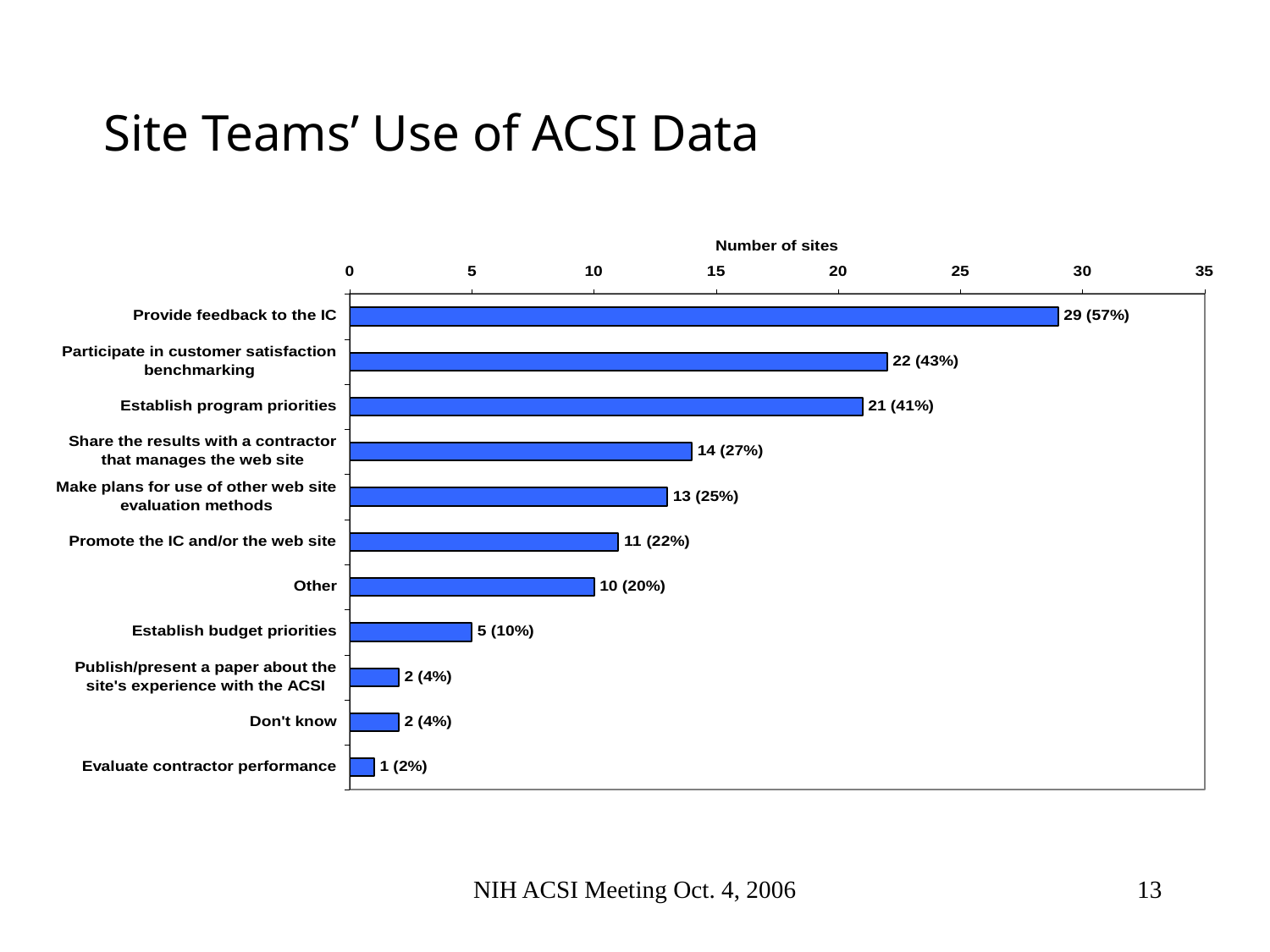

Site Teams’ Use of ACSI Data
NIH ACSI Meeting Oct. 4, 2006
13

## Slide 14
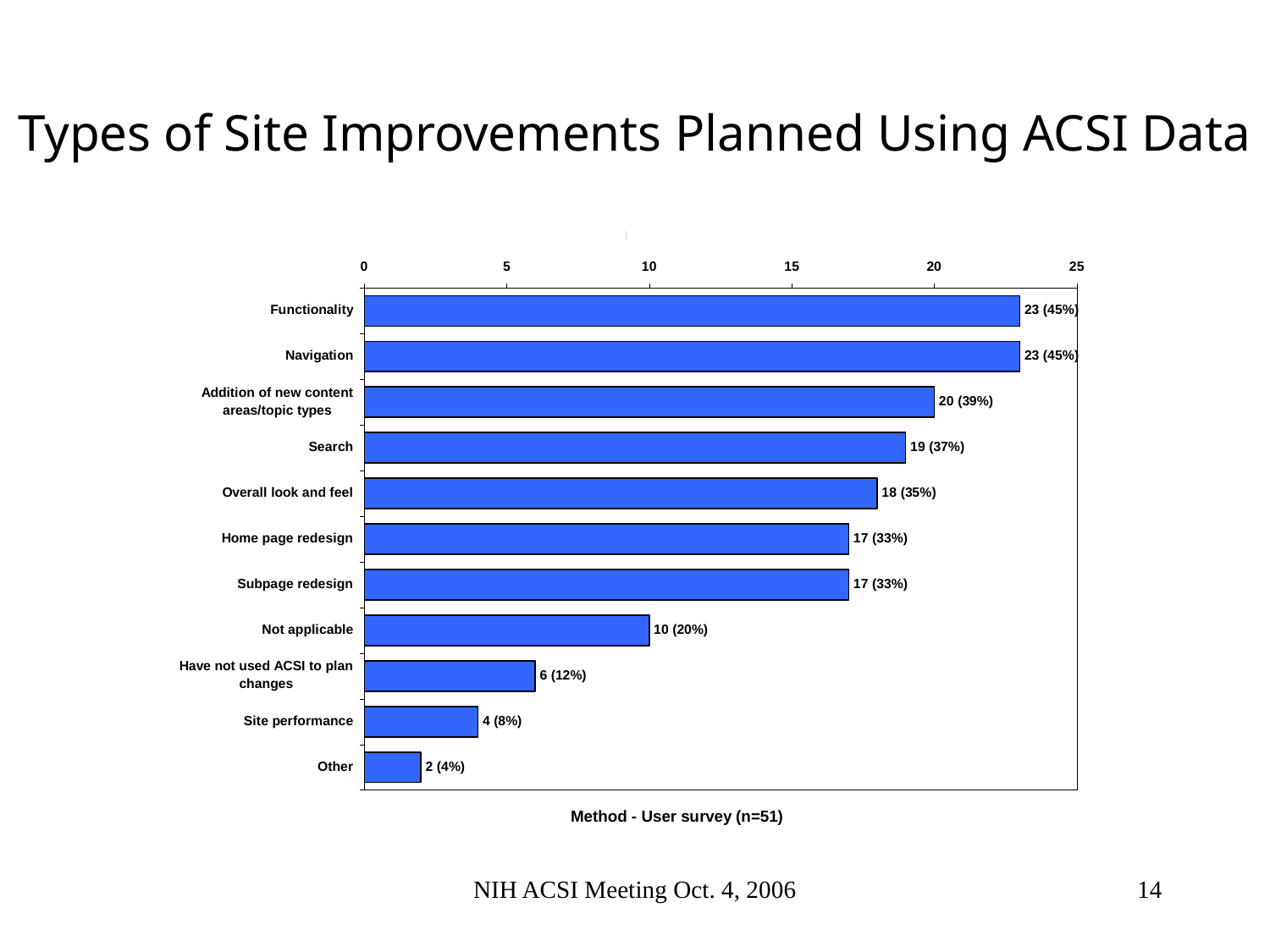

Types of Site Improvements Planned Using ACSI Data
NIH ACSI Meeting Oct. 4, 2006
14

## Slide 15
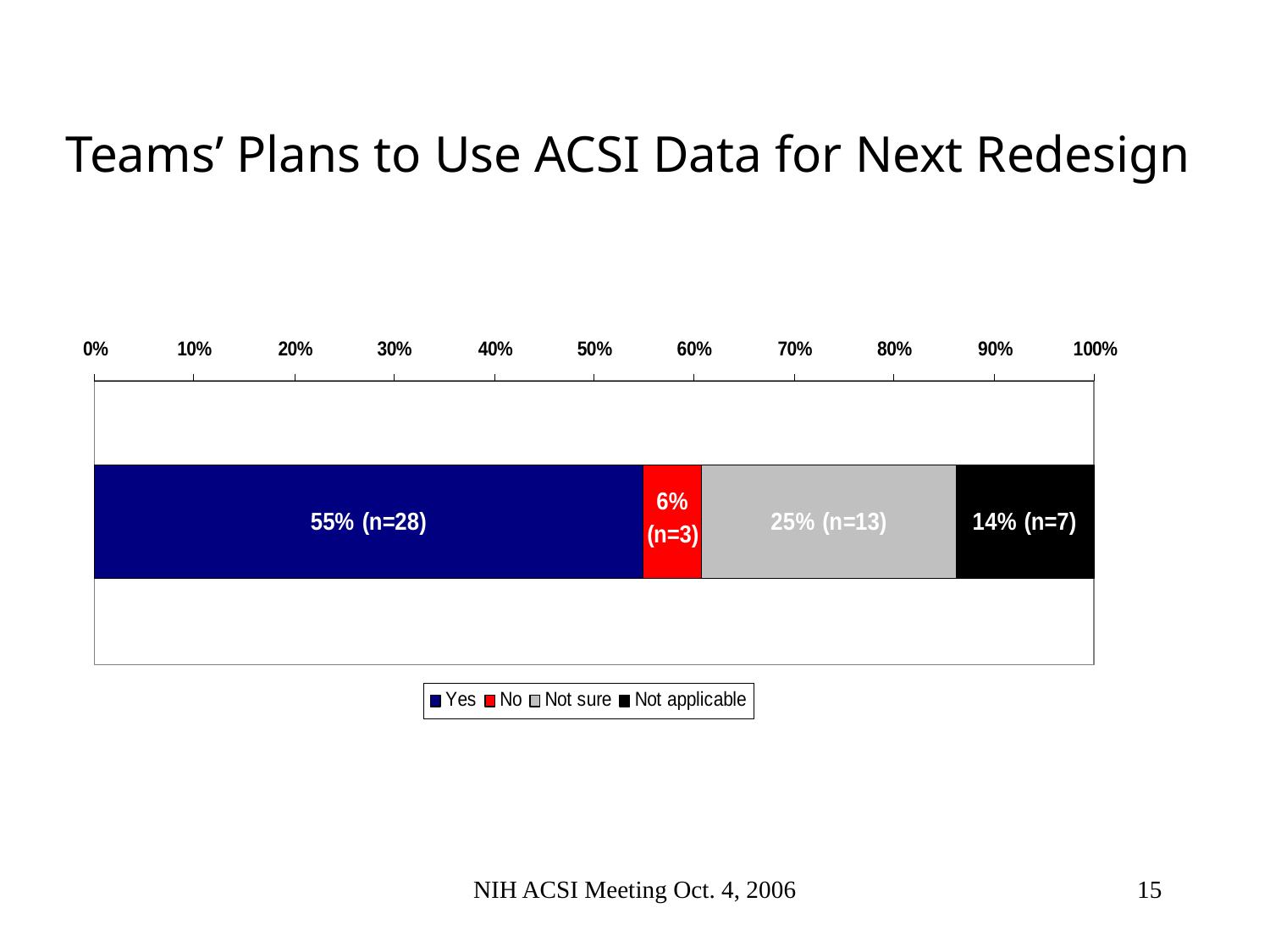

Teams’ Plans to Use ACSI Data for Next Redesign
NIH ACSI Meeting Oct. 4, 2006
15

## Slide 16
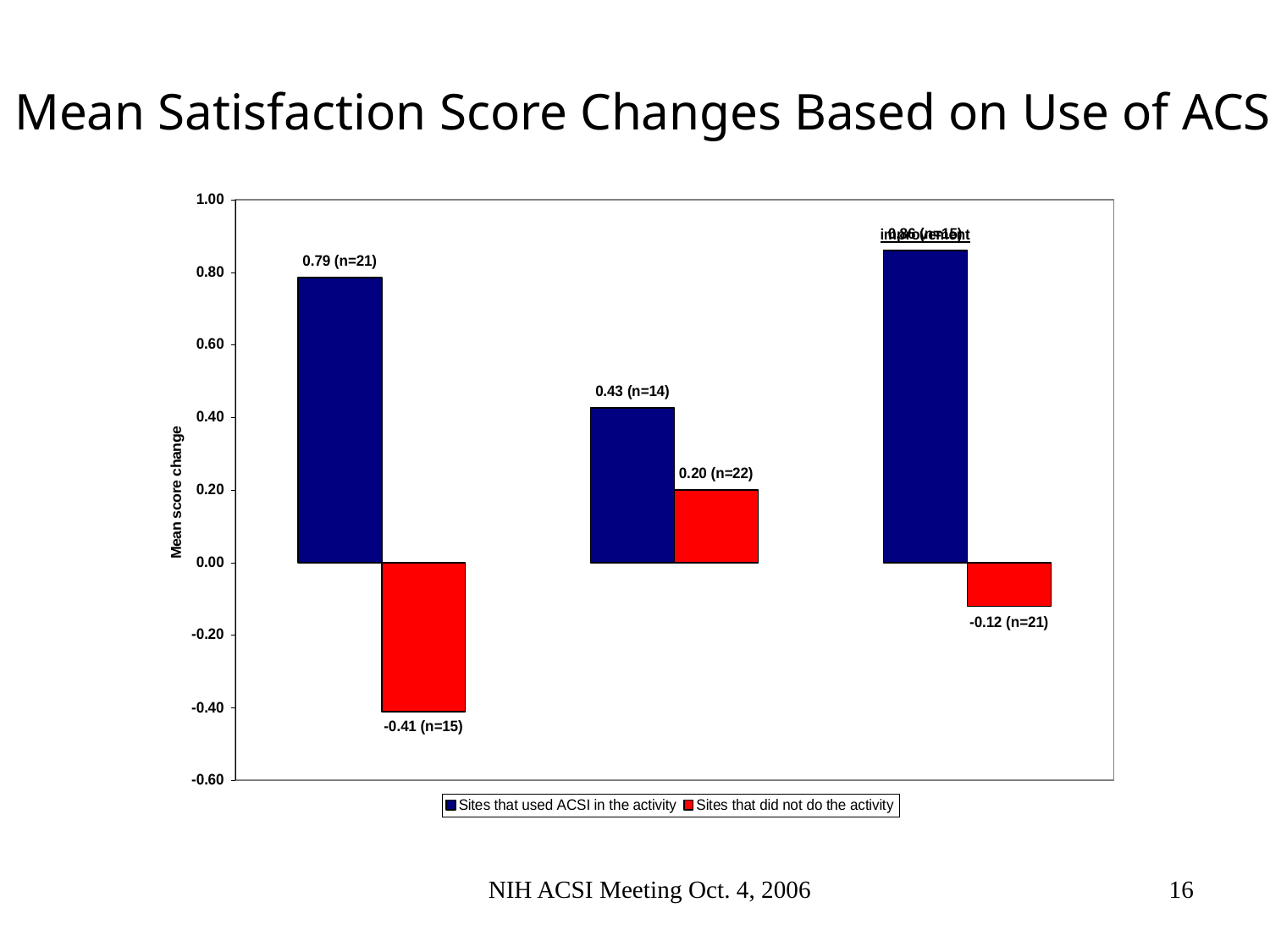

Mean Satisfaction Score Changes Based on Use of ACSI
NIH ACSI Meeting Oct. 4, 2006
16

## Slide 17
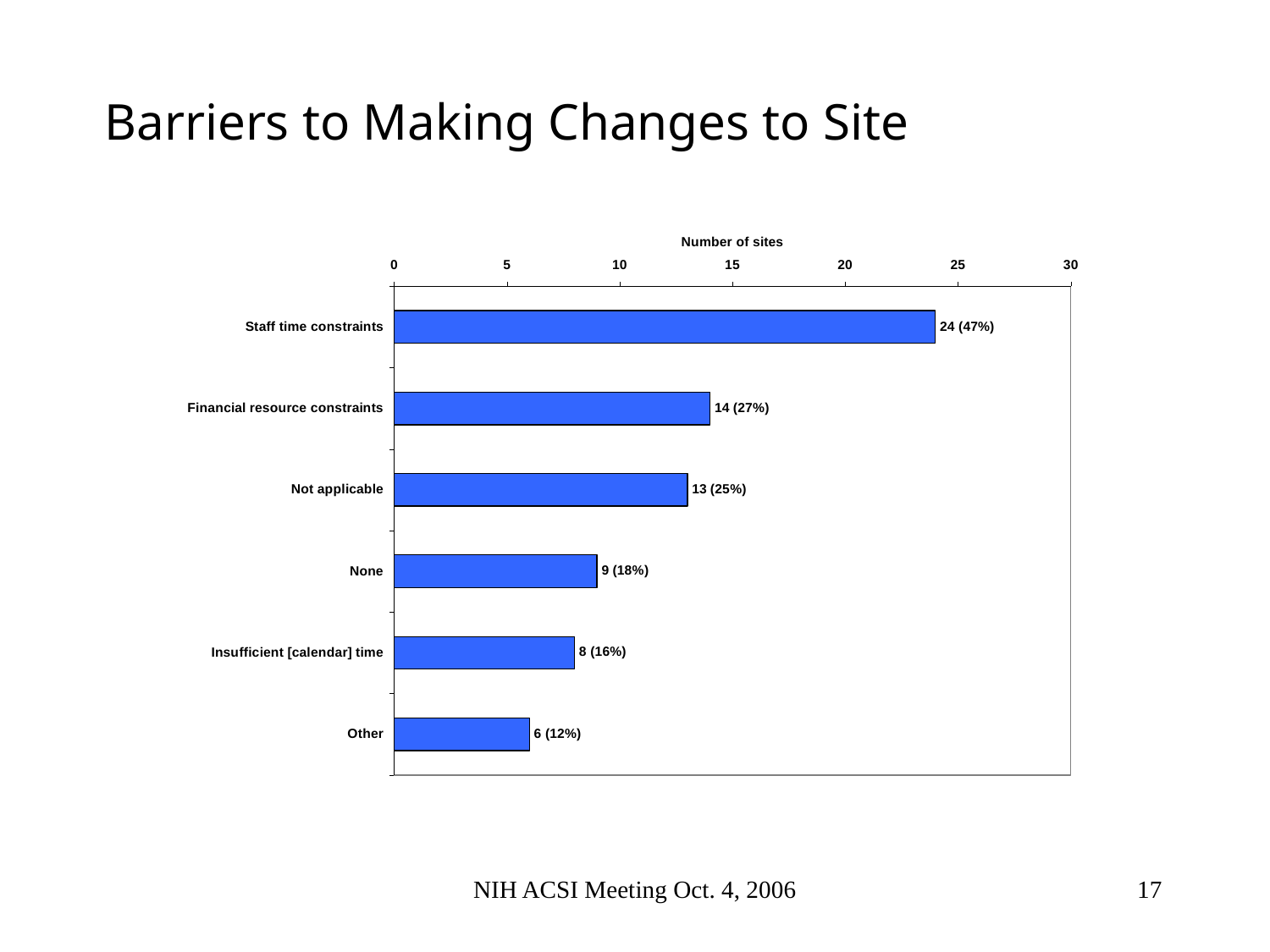

Barriers to Making Changes to Site
NIH ACSI Meeting Oct. 4, 2006
17

## Slide 18
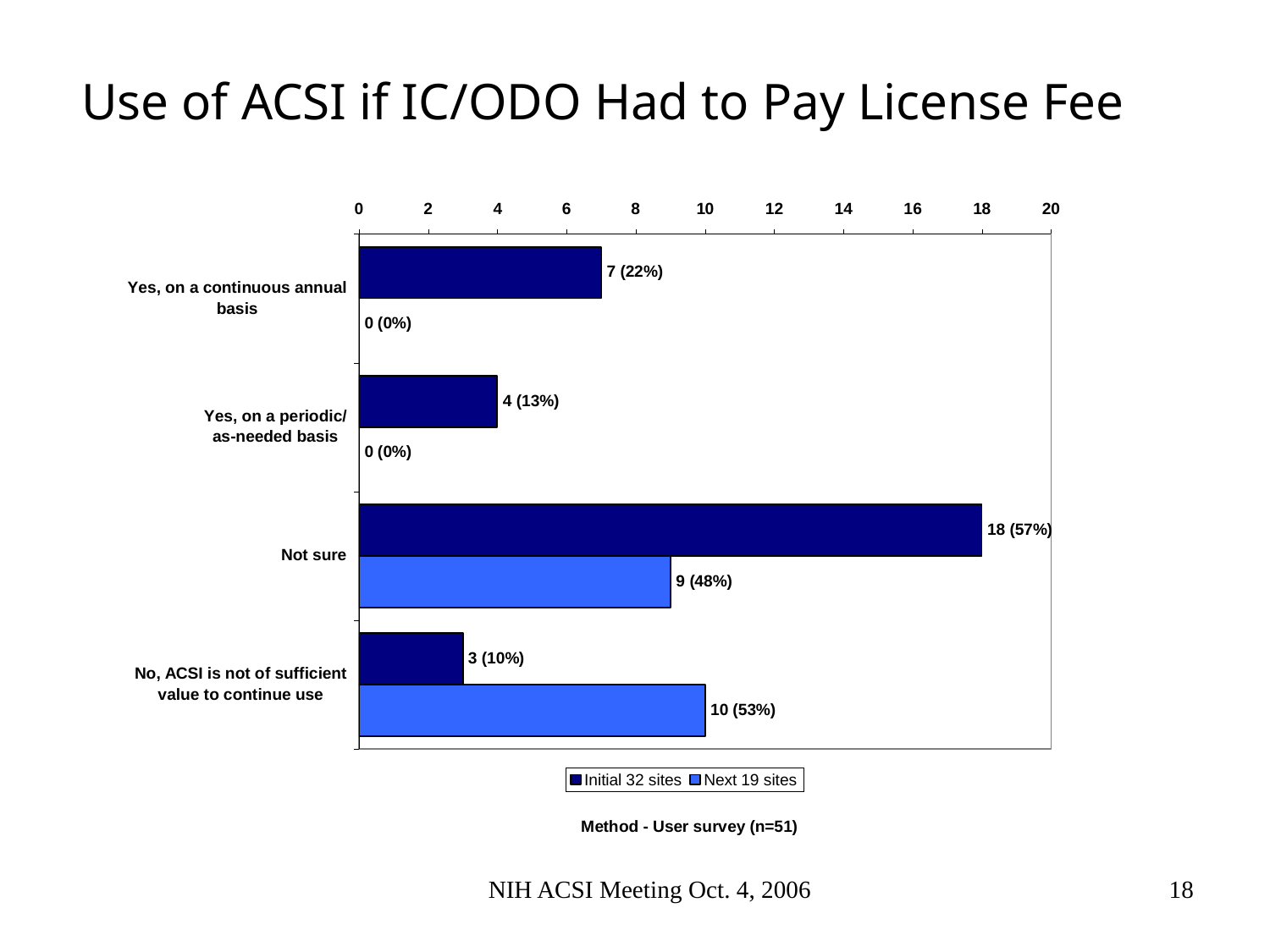

Use of ACSI if IC/ODO Had to Pay License Fee
NIH ACSI Meeting Oct. 4, 2006
18

## Slide 19
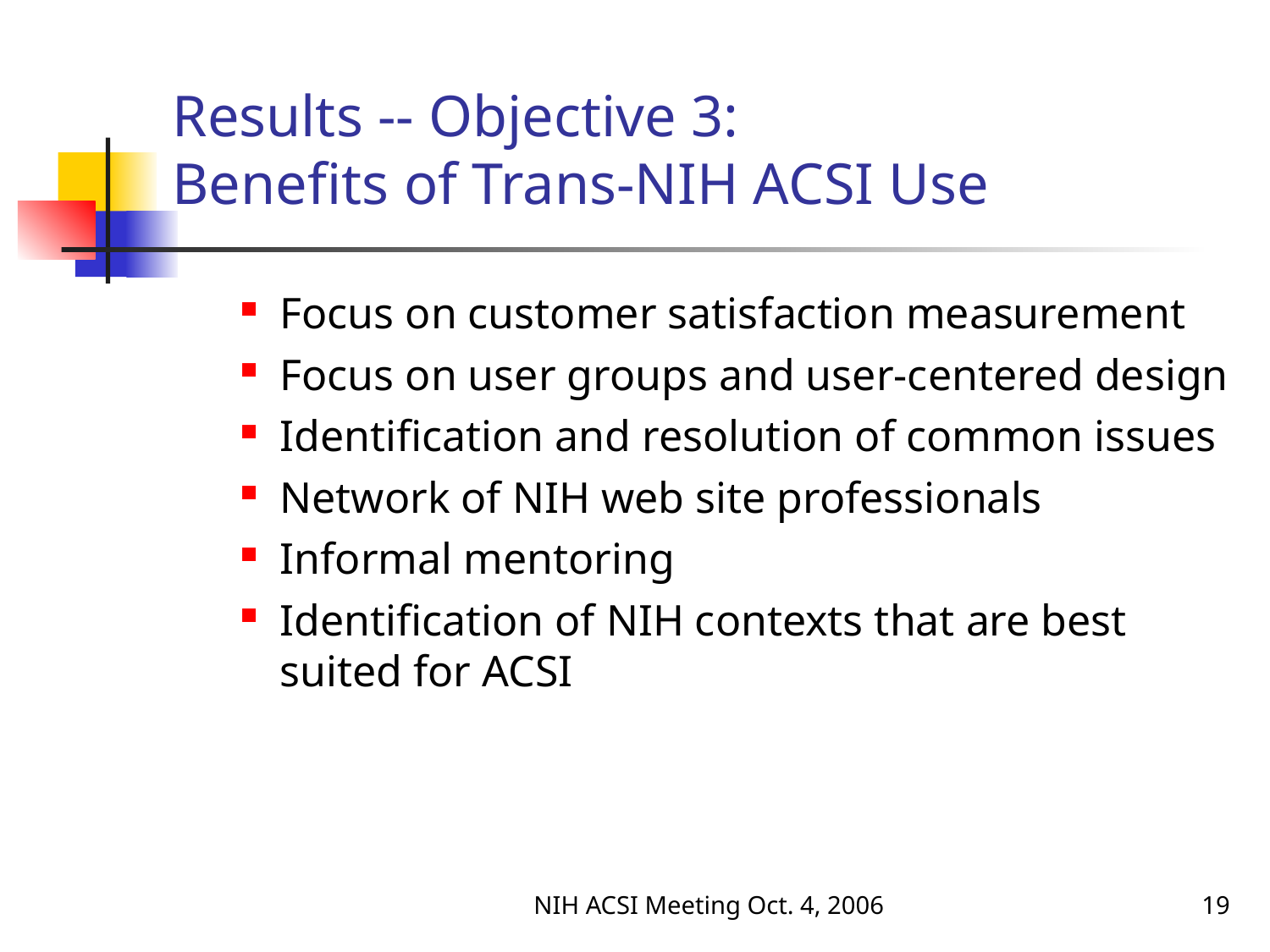

# Results -- Objective 3: Benefits of Trans-NIH ACSI Use
Focus on customer satisfaction measurement
Focus on user groups and user-centered design
Identification and resolution of common issues
Network of NIH web site professionals
Informal mentoring
Identification of NIH contexts that are best suited for ACSI
NIH ACSI Meeting Oct. 4, 2006
19

## Slide 20
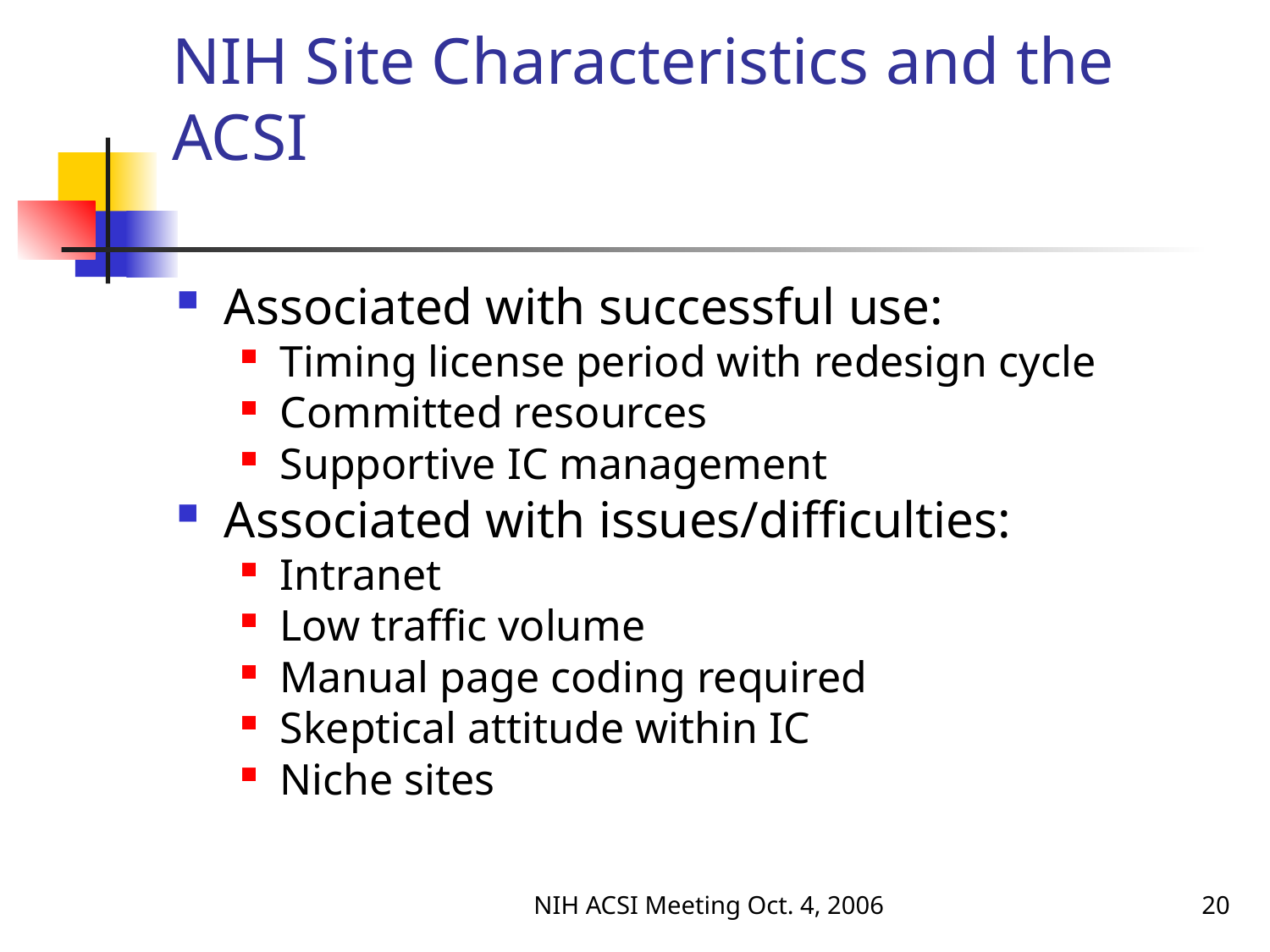

# NIH Site Characteristics and the ACSI
Associated with successful use:
Timing license period with redesign cycle
Committed resources
Supportive IC management
Associated with issues/difficulties:
Intranet
Low traffic volume
Manual page coding required
Skeptical attitude within IC
Niche sites
NIH ACSI Meeting Oct. 4, 2006
20

## Slide 21
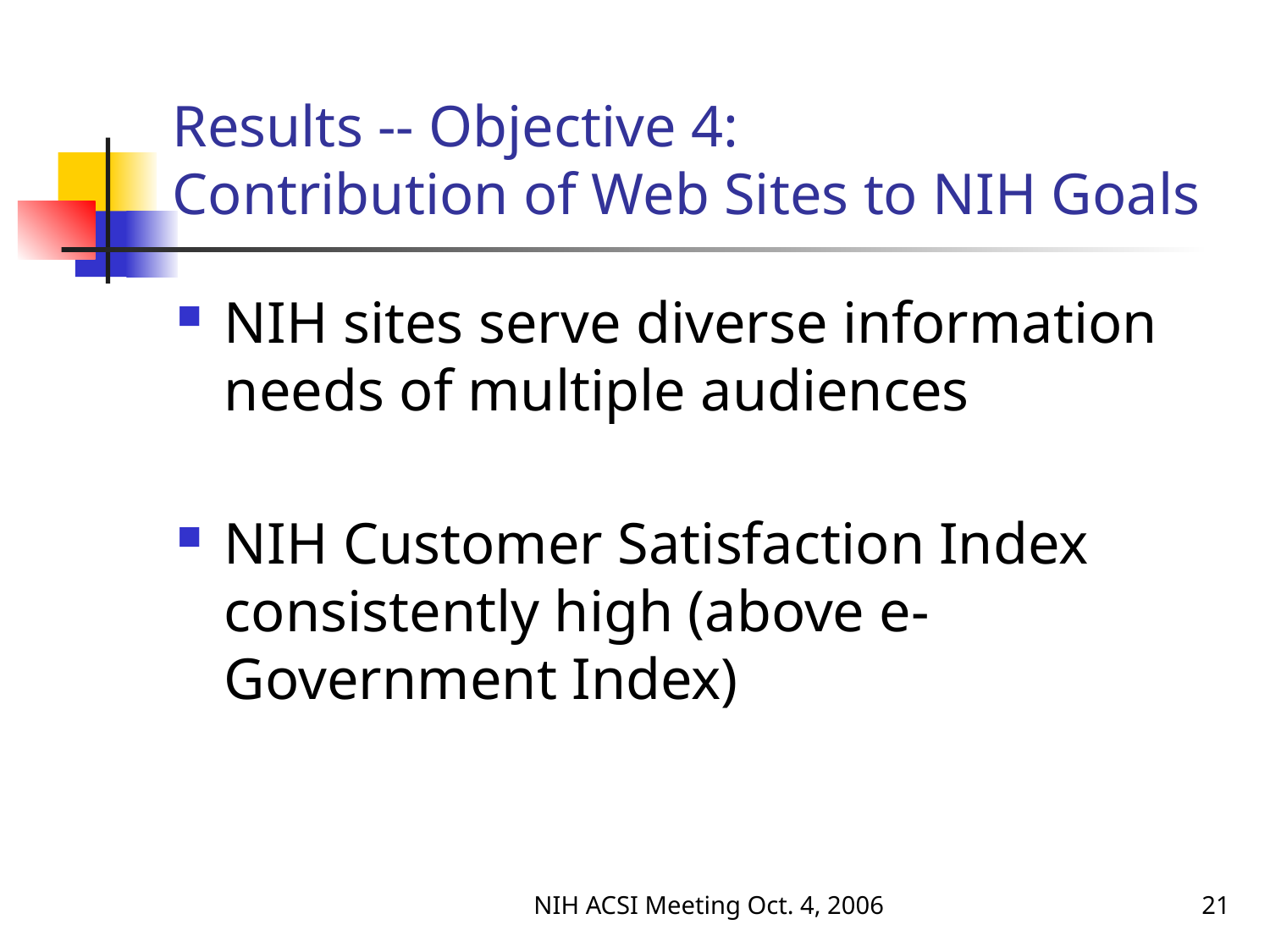

# Results -- Objective 4: Contribution of Web Sites to NIH Goals
NIH sites serve diverse information needs of multiple audiences
NIH Customer Satisfaction Index consistently high (above e-Government Index)
NIH ACSI Meeting Oct. 4, 2006
21

## Slide 22
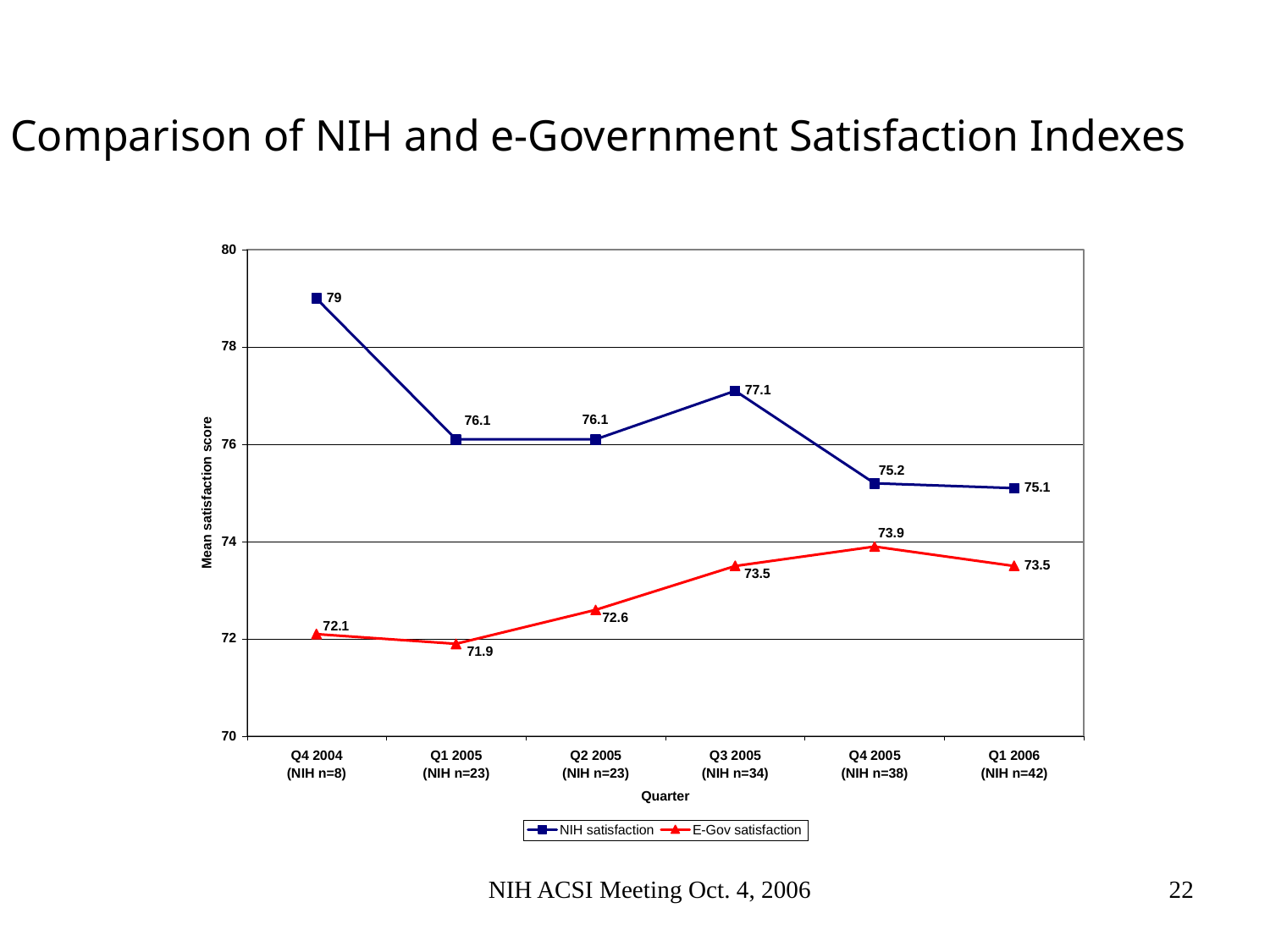

Comparison of NIH and e-Government Satisfaction Indexes
NIH ACSI Meeting Oct. 4, 2006
22

## Slide 23
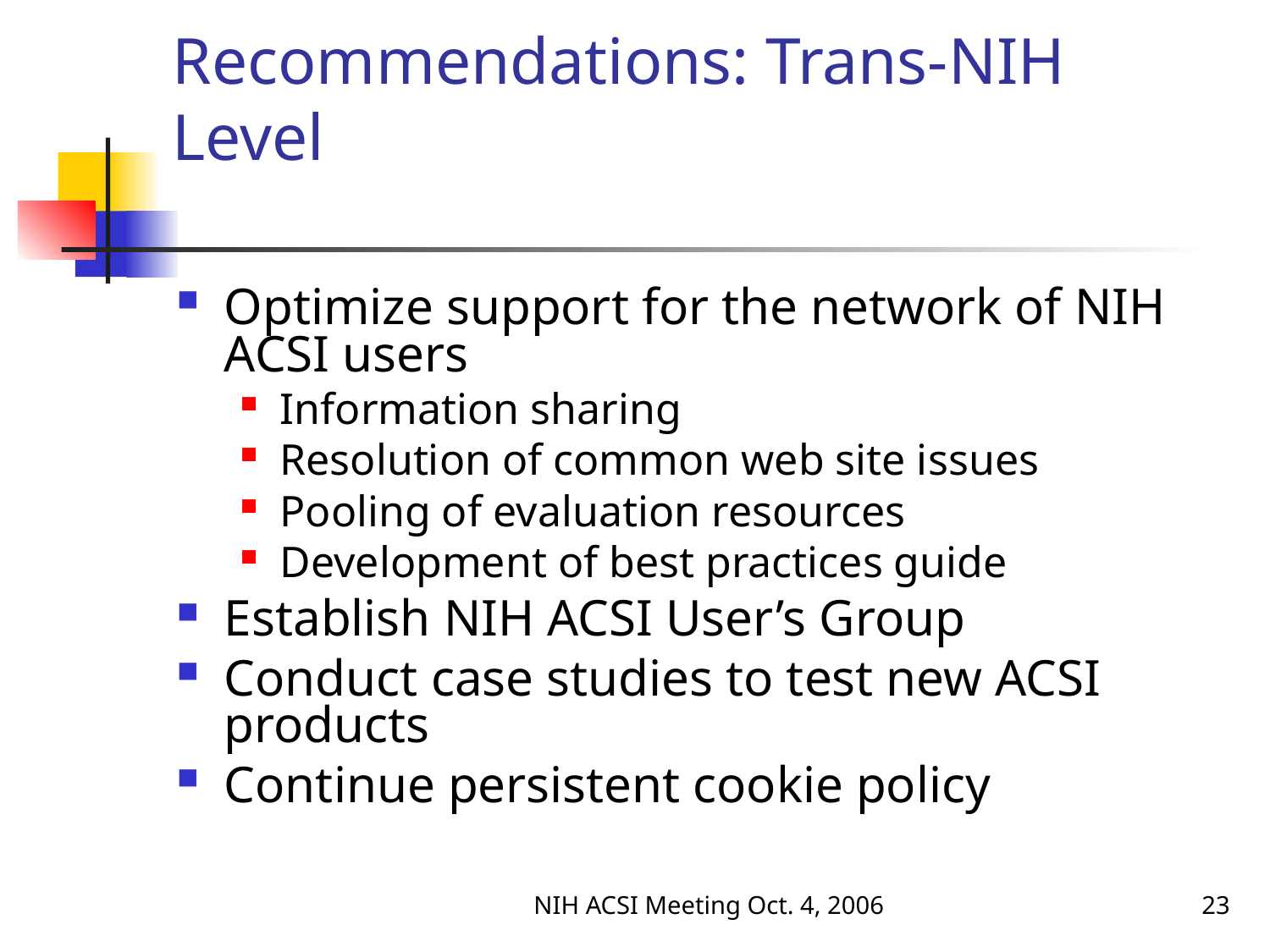

# Recommendations: Trans-NIH Level
Optimize support for the network of NIH ACSI users
Information sharing
Resolution of common web site issues
Pooling of evaluation resources
Development of best practices guide
Establish NIH ACSI User’s Group
Conduct case studies to test new ACSI products
Continue persistent cookie policy
NIH ACSI Meeting Oct. 4, 2006
23

## Slide 24
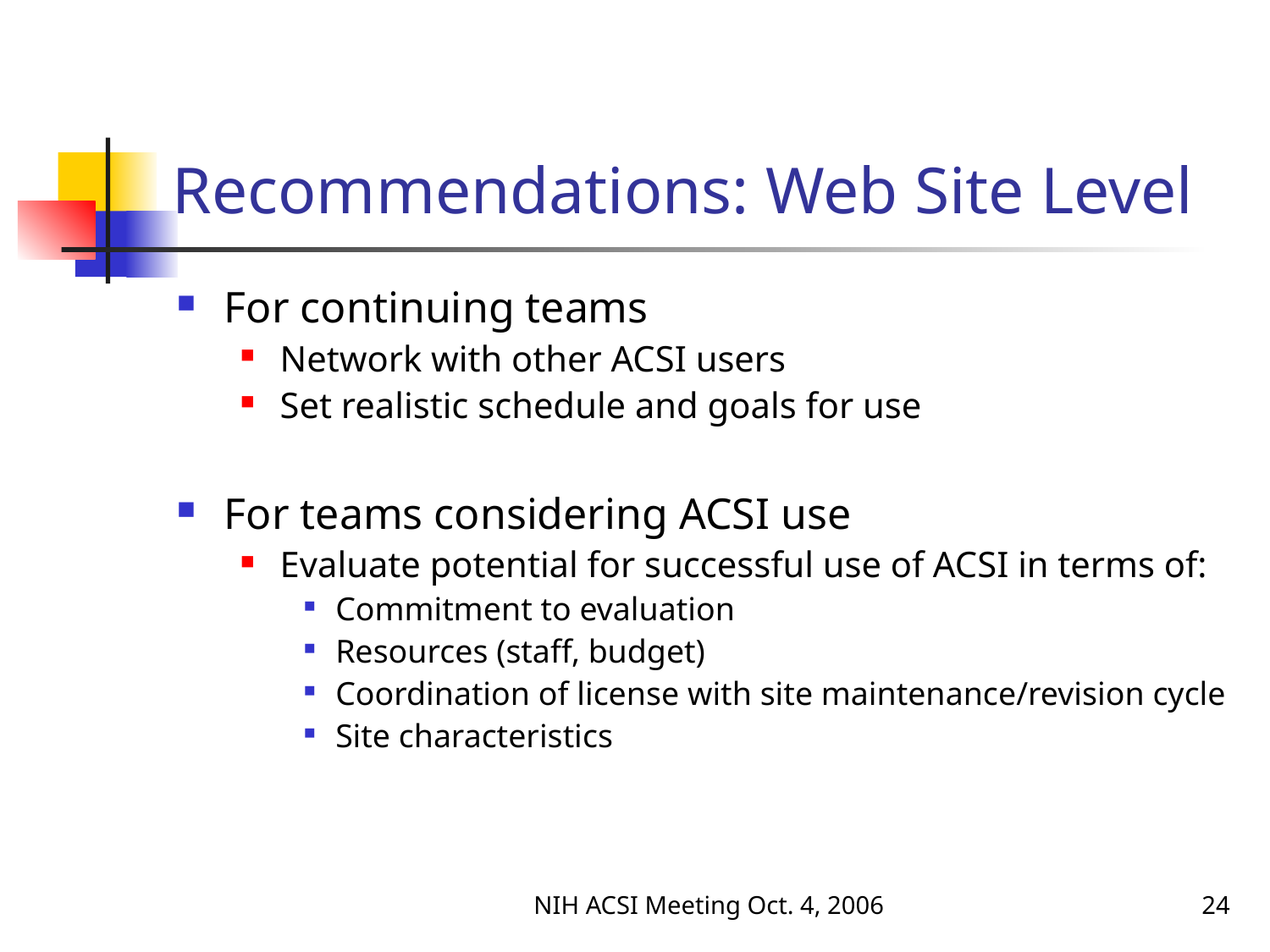

# Recommendations: Web Site Level
For continuing teams
Network with other ACSI users
Set realistic schedule and goals for use
For teams considering ACSI use
Evaluate potential for successful use of ACSI in terms of:
Commitment to evaluation
Resources (staff, budget)
Coordination of license with site maintenance/revision cycle
Site characteristics
NIH ACSI Meeting Oct. 4, 2006
24

## Slide 25
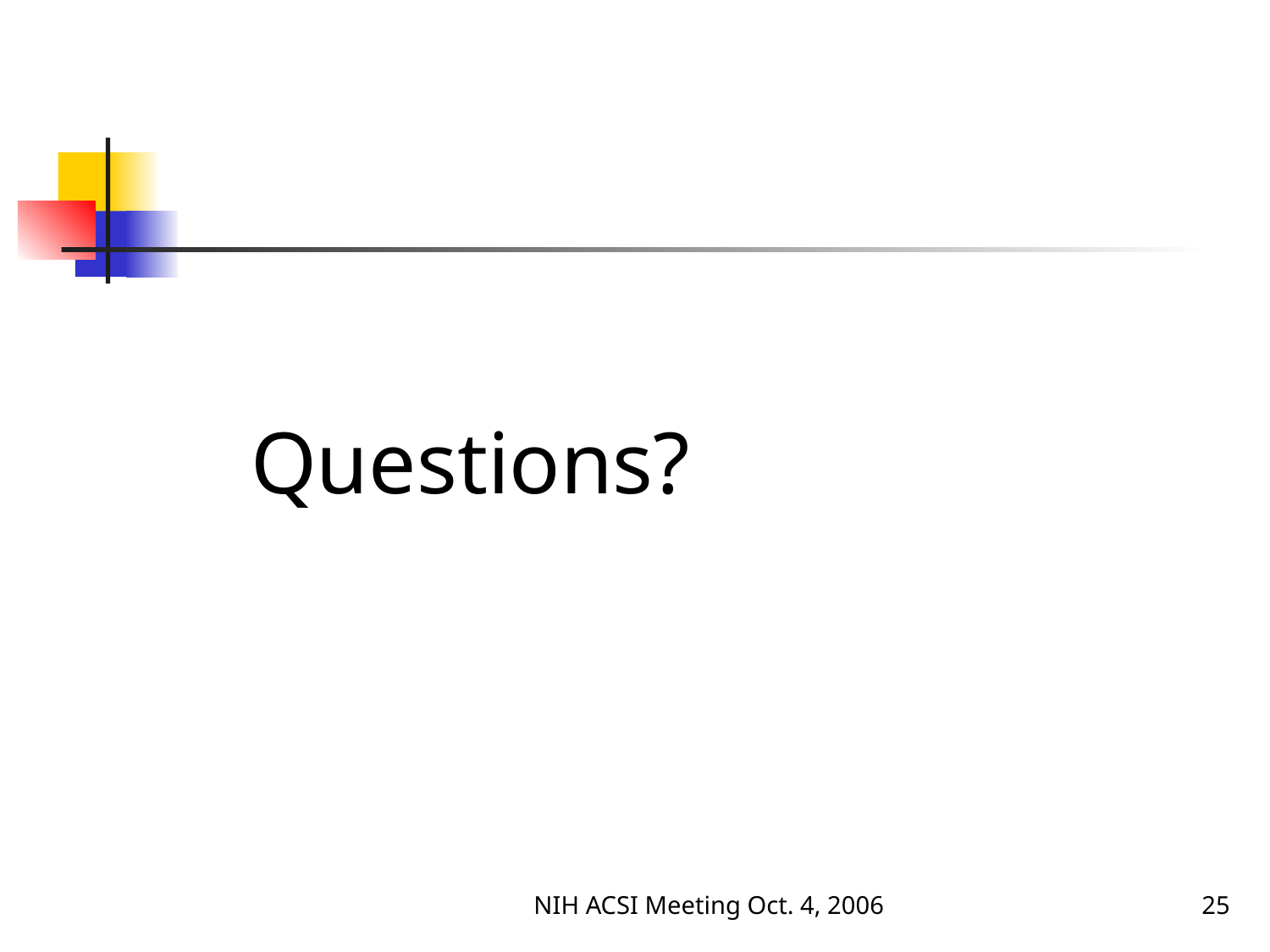

# Questions?
NIH ACSI Meeting Oct. 4, 2006
25
